# A Bovine Lymphosarcoma Cell Line Infected with *Theileria annulata* Exhibits an Irreversible Reconfiguration of Host Cell Gene Expression

**DOI:** 10.1371/journal.pone.0066833

**Published:** 2013-06-26

**Authors:** Jane H. Kinnaird, William Weir, Zeeshan Durrani, Sreerekha S. Pillai, Margaret Baird, Brian R. Shiels

**Affiliations:** 1 Institute of Infection, Immunity and Inflammation, College of Medical, Veterinary and Life Sciences, University of Glasgow, Glasgow, United Kingdom; 2 Institute of Cell Biology, School of Biological Sciences, University of Edinburgh, Edinburgh, United Kingdom; Institut national de la santé et de la recherche médicale – Institut Cochin, France

## Abstract

*Theileria annulata*, an intracellular parasite of bovine lymphoid cells, induces substantial phenotypic alterations to its host cell including continuous proliferation, cytoskeletal changes and resistance to apoptosis. While parasite induced modulation of host cell signal transduction pathways and NFκB activation are established, there remains considerable speculation on the complexities of the parasite directed control mechanisms that govern these radical changes to the host cell. Our objectives in this study were to provide a comprehensive analysis of the global changes to host cell gene expression with emphasis on those that result from direct intervention by the parasite. By using comparative microarray analysis of an uninfected bovine cell line and its *Theileria* infected counterpart, in conjunction with use of the specific parasitacidal agent, buparvaquone, we have identified a large number of host cell gene expression changes that result from parasite infection. Our results indicate that the viable parasite can irreversibly modify the transformed phenotype of a bovine cell line. Fifty percent of genes with altered expression failed to show a reversible response to parasite death, a possible contributing factor to initiation of host cell apoptosis. The genes that did show an early predicted response to loss of parasite viability highlighted a sub-group of genes that are likely to be under direct control by parasite infection. Network and pathway analysis demonstrated that this sub-group is significantly enriched for genes involved in regulation of chromatin modification and gene expression. The results provide evidence that the *Theileria* parasite has the regulatory capacity to generate widespread change to host cell gene expression in a complex and largely irreversible manner.

## Introduction

*Theileria annulata* and the closely related species, *T. parva* are tick-transmitted protozoan parasites of cattle. Both parasites cause debilitating and often fatal disease syndromes, tropical theileriosis in the case of *T. annulata* and East coast fever by *T. parva.* Following introduction into the host animal by a feeding tick, sporozoites rapidly invade and establish a membrane delineated, multi-nucleate macroschizont within white blood cells, predominantly those of the monocyte-macrophage lineage in the case of *T. annulata* and T-cells for *T. parva*, reviewed in [1]. Infection of leukocytes by either species induces numerous, conserved changes to the host cell that promote cell division and generate a neoplastic phenotype. Expansion of the parasitised lymphocyte population results in development of multiple tumour-like foci throughout the body of an infected animal [2]. Host cell division is dependent on the viable parasite since treatment with the theileriacidal drug, buparvaquone (BW720c) results in a cessation of proliferation followed by apoptosis of the infected cell [3], [4]. Establishment of the infected cell phenotype is known to involve constitutive activation of the pro-inflammatory transcription factor NFκB (reviewed in [1]). This is a critical event, as it provides the proliferating infected cell with anti-apoptotic properties [5]. NFκB activation appears to be parasite dependent and is initiated by sequestration of ‘IKK (IκB-kinase) signalosomes’ to the surface of the macroschizont [6]. In addition to hijacking host IKK signalosome function, there is evidence that the parasite-infected cell modulates other signalling pathways. These include constitutive activation of the AP1 transcription factor [7] by cJUN NH2-terminal kinase signalling [8]; induction of TGF-β signalling which also appears to be involved in regulation of metastatic potential and hence virulence of *T. annulata* infected leukocytes [9] and constitutive phosphoinositide 3′-kinase (PI3-K) activity that supports proliferation and possibly contributes to elevation of AP1 and NFκB activity [10]. Activity of the transcription factor, cMYC is also up-regulated [11]. Such perturbation of multiple signalling events associated with the inflammatory response must have a profound influence on host cell phenotype and the associated profile of gene expression.

Parasite proteins that are exported to the host cell nucleus may also play a role in establishment of the infected host cell phenotype Those identified are encoded either within the large SVSP (sub-telomere-encoded variable secreted proteins) gene family [12] or the distinct TashAT/TpHN families [13]. Evidence from ectopic expression studies has shown that at least two TashAT factors which bind to AT-rich DNA [14] can modify a bovine cell phenotype [15], [16], pointing to a role for these proteins in modulation of the infected cell transcriptome. These studies together with the extensive data on manipulation of cell signalling pathways and leukocyte differentiation status [17], [18] suggest that *Theileria* parasites orchestrate a major reorganisation of leukocyte gene expression networks and illustrate the complexity of parasite governance over the host cell, reviewed in [1], [19].

A comparative analysis of gene expression changes that occur in disease resistant versus susceptible cattle breeds following *T. annulata* sporozoite infection of primary cells was carried out by [20] using a macrophage based cDNA array representing 5,026 bovine genes [21]. It was reported that significant modification of the bovine transcriptome (>1,000 of the genes represented on the array) occurred following parasite infection (Jensen et al, unpublished data cited in [20]) and more recently, a microarray analysis demonstrated that the *Theileria* parasite can substantially modulate the outcome and gene expression profiles associated with an LPS-induced inflammatory response [22]. However, a comprehensive study to investigate the full extent to which the parasite can modify a host cell gene expression profile has not been undertaken. It can be predicted that such a study will identify a plethora of parasite-induced alterations to the host cell transcriptome, but whether these can be attributed to modulation of a few or many primary host cell targets is an intriguing question.

This study has used an unbiased oligonucleotide microarray platform designed using the entire bovine mRNA REFSEQ database and the predicted coding sequences of the *T. annulata* genome, to obtain a transcriptome representative of *Theileria*-infected leukocytes. This was achieved by comparing array data generated for a bovine lymphosarcoma-derived cell line, BL20 [23] and its *T. annulata* (Hissar stock) infected counterpart, TBL20. BL20 is typical of an immortalised lymphoid cell line; sustained cell division with concomitant failure to initiate apoptosis. It is readily infected *in vitro* by *T. annulata* sporozoites resulting in establishment of a uniform population of infected cells [24]. TBL20 cells have features that are characteristic of parasitised cell lines derived from a natural infection, such as the presence of macroschizont-associated IKK signalosomes [6], [25] and the ability to generate merozoites when cultured at 41°C [26]. Thus, the BL20/TBL20 model is an ideal tool to investigate changes induced by intracellular *Theileria* parasites, as it provides an identical host background and does not rely on chemical means of cell stimulation to provide a control population for array analysis. Using these lines together with BW720c treatment to kill the parasite, we aimed to identify changes to host cell gene expression that are under control of the viable parasite. The results show that *Theileria* infection modifies the malignant phenotype, as host cell death is initiated following parasite elimination rather than reversion to the original immortalised BL20 phenotype. Moreover, the data predicts that this event is linked to a major irreversible reconfiguration of host cell gene expression networks, resulting from alterations to the activation status of a number of important transcription factors and modulation of chromatin structure. Detailed documentation of the modifications imposed by this apicomplexan parasite that effectively tailor the host transcriptome towards a phenotype necessary for survival, proliferation and differentiation of the infected cell is provided.

## Materials and Methods

### Cell Lines and Culture

The uninfected control (BL20) was a bovine lymphosarcoma cell line [23] and the *T. annulata* infected line (TBL20) was derived previously from BL20 by *in vitro* infection with *T. annulata* (strain Hissar) [24]. Cells were cultured in RPMI with 20% foetal calf serum [27]. For RNA preparation, identical cultures of 2.5×10^5^ cells were set up in triplicate for each condition using fresh starter cultures. BW720c was added as appropriate to 50 ng ml^−1^ after 4 hours. Cell numbers were estimated each day by Trypan Blue exclusion and cultures fed, if required, by 2-fold dilution in fresh medium to maintain cells in exponential growth over the time course of the experiment. For analysis of comparative growth rates, cultures were set up in triplicate and treated as above except that the starting cell number was 1.5×10^5^ ml^−1^ and cultures were allowed to adapt overnight before addition of BW720c. Calculation of growth rate (cell number ml^−1^) was determined by extrapolation using the dilution factors.

### RNA preparation for microarray analysis, Northern Blotting and RT-PCR

Total RNA was prepared from 3 replicate cultures for each condition. Cell pellets were re-suspended in Tri-Reagent (Sigma) immediately after harvesting. Following extraction, the RNA was further purified on RNAeasy columns (Qiagen) according to the manufacturers' protocol. For RT-PCR, a DNAse treatment step was included.

### Microarray hybridisation and analysis of data

The custom designed 60-mer oligonucleotide *Bos taurus*/*T. annulata* microarray array platform (Roche NimbleGen Inc., Madison WI.) was described in detail in an earlier publication [22]. Briefly, 19,777 bovine sequences from the mRNA RefSeq database available at NCBI (http://www.ncbi.nlm.nih.gov/RefSeq/) together with 3,769 *T. annulata* coding sequences obtained from the *T. annulata* genome database at GeneDB (www.genedb.org) were represented on the array. The *T. annulata* probe set served as negative controls for bovine hybridisations and positive controls were bovine genes already known from previous studies to be elevated by *Theileria* infection such as members of the matrix metalloproteinase family.

Conversion of RNA to cDNA, labelling of cDNA, microarray hybridisation and RMA-normalisation of hybridisation data [28] were carried out by Roche NimbleGen. The log_2_ hybridisation values were analysed by the non-parametric Rank Product (RP) method [29], which compares favourably with similar methods in its robustness for detection of differentially expressed genes [30]. Only genes with a False Discover Rate (FDR) of less than 5% were included in subsequent analysis of differentially expressed gene sets. Genes sets classed as differentially expressed were subjected to functional analysis using Ingenuity Pathway Analysis framework V9 (IPA) (Ingenuity® Systems, http://www.ingenuity.com) as well as data obtained from the BioSystems database at NCBI (http://www.ncbi.nlm.nih.gov/biosystems/). In some instances manual curation of the data was undertaken. The datasets analysed in this publication have been deposited in NCBI's Gene Expression Omnibus and are accessible through GEO Series Accession No. GSE44414 (http://www.ncbi.nlm.nih.gov/geo/query/acc.cgi?token=txqrjgyaoyqukzg&acc=GSE44414).

### Designation of expression levels of bovine genes

An estimate of baseline or background hybridisation was determined by taking the mean of all the values obtained for hybridisation of BL20 (uninfected) cDNA to 3,769 parasite genes represented on the array. This derived a log_2_ value of 4.5 (range 3.59–9.04 log_2_), which we used to represent the background hybridisation value for a putatively silent gene in uninfected BL20 cells. Mean expression values for bovine genes taken across replicate hybridisations for each experimental condition below 4.5 (n = 1,283) were designated as being ‘not expressed’ (N) in BL20, although we believe that this is probably a conservative estimate; genes with mean values between 4.5 and 7 were classed as ‘low expressed’ (L); 7.0 and 9.0, medium expression level (M); 9.0–12.0, high expression (H); 12.0 and above as very high (VH). Due to the nature of hybridisation kinetics for different probes we emphasise that this classification system merely serves as a convenient guideline for interpretation.

### Validation of microarray gene expression changes by RT-PCR

Microarray hybridisation values for host actin, β-tubulin and GAPDH probe sets were very similar for BL20 and TBL20 and did not change significantly during BW720c treatment, therefore we used these as constitutive controls for semi-quantitative (SQRT-PCR) and quantitative RT-PCR (QRT-PCR). SQRT-PCR was carried out in one step using an Invitrogen One-Step RT-PCR kit with 10–50 ng total RNA and analysed as described in [22]. A control without reverse transcriptase was included for every condition. QRT-PCR was carried out in two steps; firstly cDNA was prepared from 2 μg total RNA using Agilent Technologies AffinityScript QPCR cDNA Synthesis Kit with oligo dT primers. QRT-PCR amplifications of cDNA stocks were carried out in duplicate using a Stratagene Mx3005P QPCR system and incorporating SybrGreen for detection. All procedures were carried out according to the manufacturers' protocols and quantitative data analysed using MxPro QPCR software. Reaction conditions and analysis are described in detail in a previous publication [22]. Briefly, GAPDH was selected as the best overall constitutive control gene for QRT-PCR from the 3 constitutive genes that were assessed. Relative gene expression values for each gene of interest were calculated by normalising to those of GAPDH. Fold change was calculated for selected genes in parasite-infected and BW720c treated samples relative to control BL20 as calibrator. Unique primers pairs used to amplify bovine mRNAs (Table S1) were designed using Primer-Blast at NCBI (http://www.ncbi.nlm.nih.gov/tools/primer-blast/index.cgi) and assessed for secondary structure using M-Fold (http://mfold.rna.albany.edu/). Where possible, they were designed to span or include an intron to avoid amplification/detection of genomic DNA and to have similar melting temperatures in the range 58–61°C.

### Preparation of protein extracts and Western Blotting

Total protein extracts were prepared from uninfected BL20, infected TBL20 and TBL20 treated with BW720c cultured under the same conditions as for preparation of RNA for microarray analysis. 10^6^ cells were lysed in 40µl 1× SDS-PAGE sample cocktail buffer and SDS-PAGE gels run, transferred and blotted using standard procedures as described previously in [14]. Approximately 2.5×10^5^ cells were loaded per track and equal protein loadings confirmed by Ponceau S staining of the transfer membrane. Due to the relative lack of available bovine-specific antibody reagents, commercial antibodies chosen for testing were generated against regions of high conservation across mammalian species. Antibodies were used at the following dilutions; anti-HDAC9 (Santa Cruz Biotechnology, Cat No sc-28732) 1/200 dilution; HDAC2 (Santa Cruz Biotechnology, sc-81599), 1/200; NFκB2 (Santa Cruz Biotechnology, sc-298), 1/100; *T. annulata* specific antibodies, anti-TA10720 (ER HSP90, J. Kinnaird, unpublished), 1/100 and anti-Tasp [31] 1/5000, were generated in this laboratory; antibodies against constitutively expressed control host proteins β-tubulin (Sigma, T9028), 1/5000 and actin (Sigma, A4700), 1/1000. Primary antibody was detected using the appropriate secondary antibody conjugated to horseradish peroxidase. Immunoblots were performed three times for HDAC9 and twice for NFκB2. The cultures used to generate protein extracts were completely independent of those used to generate mRNA.

### Measurement of Caspase 3/7 Activity

Caspase 3/7 activity was estimated from logarithmically growing cultures under the conditions and at the time points indicated in the text. Activity from 10^4^ viable cells was determined using a luciferase-based assay (Promega) according to the manufacturer's protocol.

## Results and Discussion

### BW720c treatment of TBL20 is detrimental to host cell proliferation, survival and parasite gene expression

**Figure 1:**
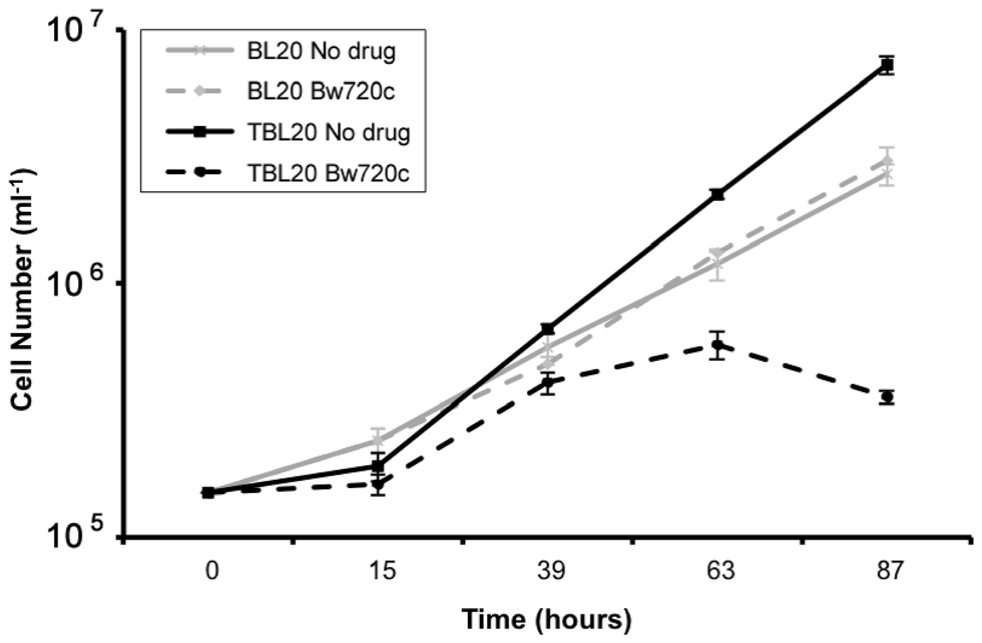
Comparative growth rates of BL20 and TBL20 cell lines during BW720c treatment. Comparison of growth rates of uninfected (BL20) and infected (TBL20) cell lines in the presence and absence of BW720c at 50 ng ml^−1^. Culturing was as described in Methods section. All cultures were counted daily with three replicate cultures used for each condition. Error bars represent standard deviations from the mean for each time point. Solid lines, non-drug treated cultures; dashed lines, BW720c treated; thin line, BL20 and thick line, TBL20.

**Figure 2:**
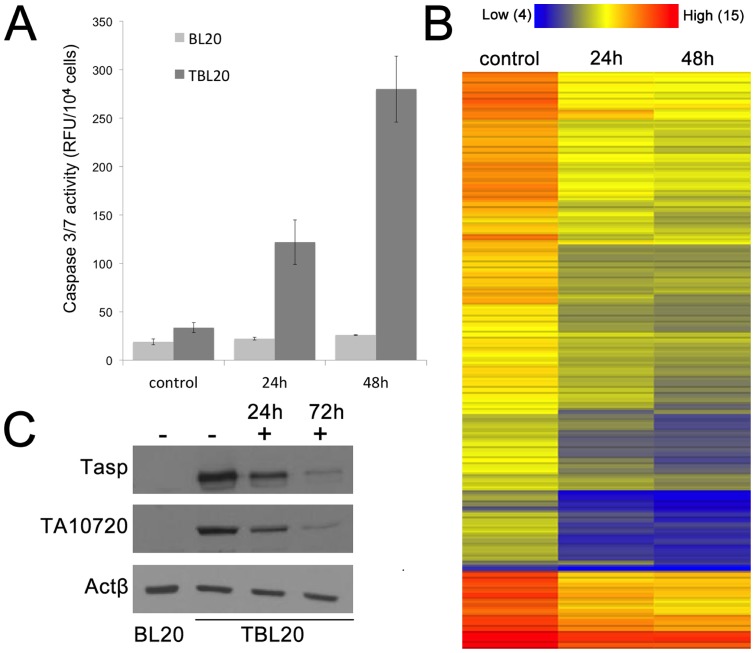
**A. Caspase 3/7 activity in BW720c-treated uninfected BL20 and infected TBL20.** Caspase 3/7 activity was assayed on samples of 10^4^ cells taken from triplicate cultures for each cellular condition; BL 20 and TBL20, untreated and treated with BW720c for 24 and 48 h. Error bars represent the standard deviation from the mean. **B. Heat map illustration of the relative expression levels for parasite house-keeping genes before and after exposure to BW720c for 24 and 48** **h** 106 parasite house-keeping genes were randomly selected based on their annotation. The majority of parasite genes show considerably decreased levels by 24 h. Expression level range is 3.9 (blue, low) to 14.9 (red, high). Values are log_2_. **C. Western analysis demonstrated that expression of parasite proteins Tasp and TA10720 decreased during exposure to BW720c** Total protein cell extracts were prepared from untreated BL20 and TBL20 (−) and in TBL20 cells at 24 and 72 (+) hours after addition of BW72c. Equal protein loadings were verified by Ponceau S staining. Parasite proteins, Tasp (schizont surface) and TA10720 (HSP90-related, schizont organelle) are shown relative to constitutive bovine actin. Antibodies are as described in methods section.

The specific theileriacidal activity of BW720c is well established both *in vivo* [32] and *in vitro.* This latter property has been utilised in several studies including those by [18], [33] and [17] to predict whether individual host genes/pathways are under direct influence of the viable macroschizont. BW720c, a hydroxynaphthoquinone, most probably acts as a ubiquinone analogue [34], [35] to specifically block parasite electron transport and exposure of infected cells to the drug leads to rapid arrest and death of the parasite, followed by cessation of host cell proliferation and apoptosis within a few days [4]. However, given the *T. annulata* infected line, TBL20, has arisen from the infection of a cell line that already displays an immortalised phenotype, reversal of proliferation status and induction of apoptosis may not occur on treatment with BW720c. To test this premise, infected TBL20 cells were cultured in the presence and absence of the drug and their proliferation potential assessed relative to uninfected BL20. The results clearly demonstrated that TBL20 cells proliferate at a faster rate than their uninfected counterpart, BL20 (Figure 1). Moreover, treatment of TBL20 with BW720c resulted in a significant reduction in proliferation rate, detectable from about 30 h, with complete arrest by ∼48 h. In contrast, BL20 showed no detectable effect of BW720c on the rate of division. A similar growth arrest phenotype following BW720c treatment was recently described for *T. annulata* infected cells derived from a different immortalised host cell line [36], indicating that the result is not restricted to the BL20 host cell background. Estimation of caspase 3/7 activity, a marker of progression towards apoptosis, showed a significant increase in the TBL20 BW720c-treated culture that was not detected in control (no drug) TBL20 cells or uninfected BL20 cells. Interestingly, caspase 3/7 activity was detectable in TBL20 by 24 h exposure to the drug, indicating progression towards apoptosis was initiated prior to this sampling point (Figure 2A). Further support was provided by Western blot detection of another early marker of apoptosis, the poly-ADP ribose polymerase (PARP) cleavage product, in TBL20 cells during BW720c treatment but not in BL20 cells (data not shown).

Confirmation that BW720c was detrimental to the macroschizont was achieved by morphology and at the RNA and polypeptide level. Giemsa staining indicated an overall reduction in size and nuclear content of the macroschizonts by 48 hours (data not shown). To assess the outcome of BW720c treatment on the parasite transcriptome, Rank Product analysis of the data obtained from hybridisation of TBL20 cDNA to the parasite genes represented on the Glasgow bovine/*Theileria* genome microarray (see methods) was undertaken. Expression of the vast majority of parasite genes was significantly reduced (3,282 out of 3,769 > 2-fold) and is illustrated for 120 randomly selected housekeeping genes by a heat map over the 48 h of BW720c treatment (Figure 2B). Loss of parasite protein production was also indicated as Western blot analysis over a 72 h BW720c time course using antibodies specific for parasite proteins, Tasp and an HSP90-like polypeptide expressed at the macroschizont stage, showed a clear reduction in levels (Figure 2C). It can be concluded that a clear outcome of killing the parasite in the infected TBL20 lymphosarcoma line is a cessation of host cell division together with initiation of cellular processes leading to cell death. Thus, it can be proposed that infection with the parasite reconfigures the BL20 gene expression network in such a way that upon removal of a parasite-dependent transforming signal(s), the host cell cannot revert to its original immortalised phenotype and undergoes apoptosis in a manner comparable to naturally infected *T. annulata* cell lines.

### Overall changes in host cell gene expression associated with parasite infection

A major aim of this study was to identify the capacity of *Theileria* to manipulate the gene expression network of the bovine host cell and identify differentially expressed genes that are most likely to be directly controlled by a parasite mediated mechanism (expression profile altered by BW720c treatment). To achieve this we compared the transcriptome of the immortalised bovine cell line, BL20, with that of TBL20; followed by profiling the transcriptional changes induced by treatment of TBL20 cells with BW720c at 24 and 48 hours. BL20 cultures were also BW720c treated and analysed in parallel to exclude alterations in gene expression caused by a direct effect of the drug on uninfected host cells. Thus, the entire experimental dataset represented differentially expressed genes (using a 5% FDR) from the following four pair-wise comparisons: BL20 v TBL20, TBL20 v TBL20_24 h_BW720c, TBL20 v TBL20_48 h_BW720c and TBL20_24 h_BW720c v TBL20_48 h_BW720c.

RP analysis of the pair-wise comparison between BL20 (uninfected) and TBL20 (infected) generated a dataset of 3,079 differentially expressed genes with 1,822 elevated and 1,257 repressed in TBL20 compared to BL20. As expected, this number of alterations was considerably higher than the previous study investigating modulation of an LPS response associated with *T. annulata* infected leukocytes (1214 genes [22]), a large number of which showed no significant difference in expression between TBL20 and BL20 (748 genes). Thus, the current study represents a more comprehensive analysis of host cell gene expression changes associated with the infected leukocyte. After 48 h exposure to BW720c, RP analysis indicated that 37% of elevated genes and 39% of repressed genes in TBL20 showed statistically significant reversal (Table 1). A number of previously reported bovine genes whose expression is altered at RNA or protein level by *T. annulata* or *T. parva* infection were also identified in our study and serve to validate the array analysis (Table S2).

**Table 1:** Overall gene expression changes for different experimental conditions.

Observation	Number of genes
*Elevated in TBL20*	*1822*
Reversed in BW720c (24h)	552
Reversed in BW720c (48h)	676
No change in BW720c	872
*Repressed in TBL20*	*1257*
Reversed in BW720c (24h)	399
Reversed in BW720c (48h)	487
No change in BW720c	678
No change in TBL20 vs BL20 (8048) but BW720c response*	*383*

Summary of the total numbers of bovine genes in the dataset where expression is altered by the presence of the *Theileria* parasite (TBL20 cells) compared to uninfected BL20 cells and the overall response following BW720c treatment of TBL20 cells to kill the parasite. Differentially expressed gene sets were derived by Rank Product analysis (FDR <5%). *This gene set included only genes for which there was no evidence for differential expression by RP analysis.

Of the genes showing elevated expression in parasite-infected TBL20 cells, the most highly up-regulated encodes the chemokine XCL2, a member of a large gene family, many of which exhibit alteration associated with the *Theileria*-infected cell. Members of the matrix metalloproteinase family (MMPs) are also found to be highly up-regulated, e.g. MMP9 which was previously identified as up-regulated in parasite infected TBL20 [7] and shown to be linked to metastasis of *Theileria* infected cells [37], was elevated 192 fold. Expression of MMP13, 14 and 25 were also greatly elevated in the BL20-TBL20 model. Thus our study provides a more comprehensive profile of MMP expression that is likely to contribute to the metastatic potential of the *Theileria* infected cell, although variable expression of family members across different infected cell lines is likely to operate [38]. 1,257 genes showed significant repression by *Theileria* infection. For example, the gene encoding SORCS3, a member of the family of Vps10p domain transmembrane receptors, displayed the greatest down-regulation. SORCS3 may have a role in mediation of endocytosis [39]. Genes encoding NFX3, a nuclear hormone receptor 3 (repressed by 166 fold) and BLK, encoding B-lymphoid tyrosine kinase (repressed 110 fold), also displayed a high level of repressed expression. This small selection of genes showing markedly elevated or repressed expression along with their response to BW720c is summarised in Table 2 and the top-ranking genes in each case are listed in Tables S3A and S3B. It should be noted that if a gene was also present in the LPS-modulated dataset described by Durrani *et al.*, the fold change will be different because in that study, the LPS response in BL20 was taken into account.

**Table 2:** Top-ranking genes elevated or repressed in TBL20 compared to BL20.

Symbol	Entrez Gene Name	ID	FC	BW720c response
XCL2	chemokine (C motif) ligand 2	319096	+741.9	−12.3
LRRTM4	leucine rich repeat transmembrane neuronal 4	521137	+314.3	
KIAA1598	No annotation	532603	+234.2	−5.1
MMP9	matrix metallopeptidase 9 (gelatinase B, 92kDa gelatinase, 92kDa type IV collagenase)	282871	+192.4	−1.9
MMP13	matrix metallopeptidase 13 (collagenase 3)	281914	+172.1	−3.9
LDLR	low density lipoprotein receptor	281276	+117.9	
LITAF	lipopolysaccharide-induced TNF factor	520564	+116.2	
CLEC4D	C-type lectin domain family 4, member D	616646	+114.8	+2.6
AHNAK	AHNAK nucleoprotein	531336	+90.2	
SERPINE1	serpin peptidase inhibitor, clade E (nexin, plasminogen activator inhibitor type 1), member 1	281375	+83.9	−3.7
IL2RB	interleukin 2 receptor, beta	510185	+80.3	
BCL2A1	BCL2-related protein A1	282151	+43.6	
SORCS3	sortilin-related VPS10 domain containing receptor 3	531405	−530.7	
F13A1	coagulation factor XIII, A1 polypeptide	617881	−289.9	
PPP1R14C	protein phosphatase 1, regulatory (inhibitor) subunit 14C	617148	−275.5	
SLC11A1	solute carrier family 11 (proton-coupled divalent metal ion transporters), member 1	282470	−238.1	+8.2
CPA4	carboxypeptidase A4	512903	−195.4	+6.6
NXF3	nuclear RNA export factor 3	523530	−166.6	
ALPK2	alpha-kinase 2	510218	−154.9	
CCR6	chemokine (C-C motif) receptor 6	519716	−110.6	+2.7
BLK	B lymphoid tyrosine kinase	532587	−110.2	+4.5
PIGR	polymeric immunoglobulin receptor	281401	−85.2	+3.7
SLAMF1	signaling lymphocytic activation molecule family member 1	281489	−84.4	
TLR4	toll-like receptor 4	281536	−70.6	+4.9

Top ranking genes with (A) elevated (+) or (B) repressed (−) expression in parasite infected TBL20 compared uninfected BL20 cells. Fold change (FC) and the direction of any BW720c response (+, up; –, down; blank no change) are indicated. Differential expression was determined by RP analysis using a FDR cut-off of 5%.

Genes that are up-regulated by *Theileria* infection might include those induced from a basal level i.e. a transcriptionally silent locus, in BL20. Each gene in the BL20 dataset was classified according to its level of hybridisation (see methods) and this resulted in 1,283 genes being categorised as potentially silent. No genes in this group showed statistically significant repression by parasite infection, supporting their prediction as silent loci. Conversely, 46 genes in the group displayed elevated expression levels associated with TBL20, with fold changes from 1.3 to 300 (5% FDR, Table S4A) and 33 of these genes showed altered expression on exposure to BW720c. Therefore, a high proportion of genes in this class exhibit modulation in BW720c (67%). This data illustrates that *Theileria* can induce expression of loci that were previously transcriptionally silent and may represent a set of genes whose expression is of importance to the *Theileria* infected cell. Those of interest include genes encoding: KIAA1598 (up 234-fold), recently identified in a protein array screen as a candidate for interaction with NEMO [40] a central component of the NFκB activation pathway; CD28 (up 52.1-fold) with a role in stimulation of the transcription factor FOX3P, mediated by NFκB [41]; BCL2A1 (up 43.6-fold) which has anti-apoptotic properties [42] and NANOG a homeobox transcription factor (see below).

In addition to the large group of genes exhibiting differential expression in parasite-infected cells, an effect of BW720c treatment on host genes classified as unaltered between BL20 and TBL20 was detected. This group was within a large gene set that contained all those judged as highly unlikely to be differentially expressed in the output from the RP analysis. 383 genes from this set showed statistically significant alterations upon treatment with BW720c that was specific to parasite-infected cells. The top ranking changes in this group are shown in Table S4B. Examples include genes encoding a poly-ADP-ribose polymerase, PARP9 (up 4-fold), transcription factor SP7 (up 17-fold) and Vanin 1 (VNN1, up 24-fold), a stress/inflammatory response regulator [43]. This dataset indicates that the parasite also modifies host gene expression to an extent that is not apparent until parasite viability is lost and in general, suggests that in TBL20, the host gene expression network is substantially altered in a non-reversible manner.

### BW720c treatment alters host cell gene expression in a non-reversible manner

**Figure 3:**
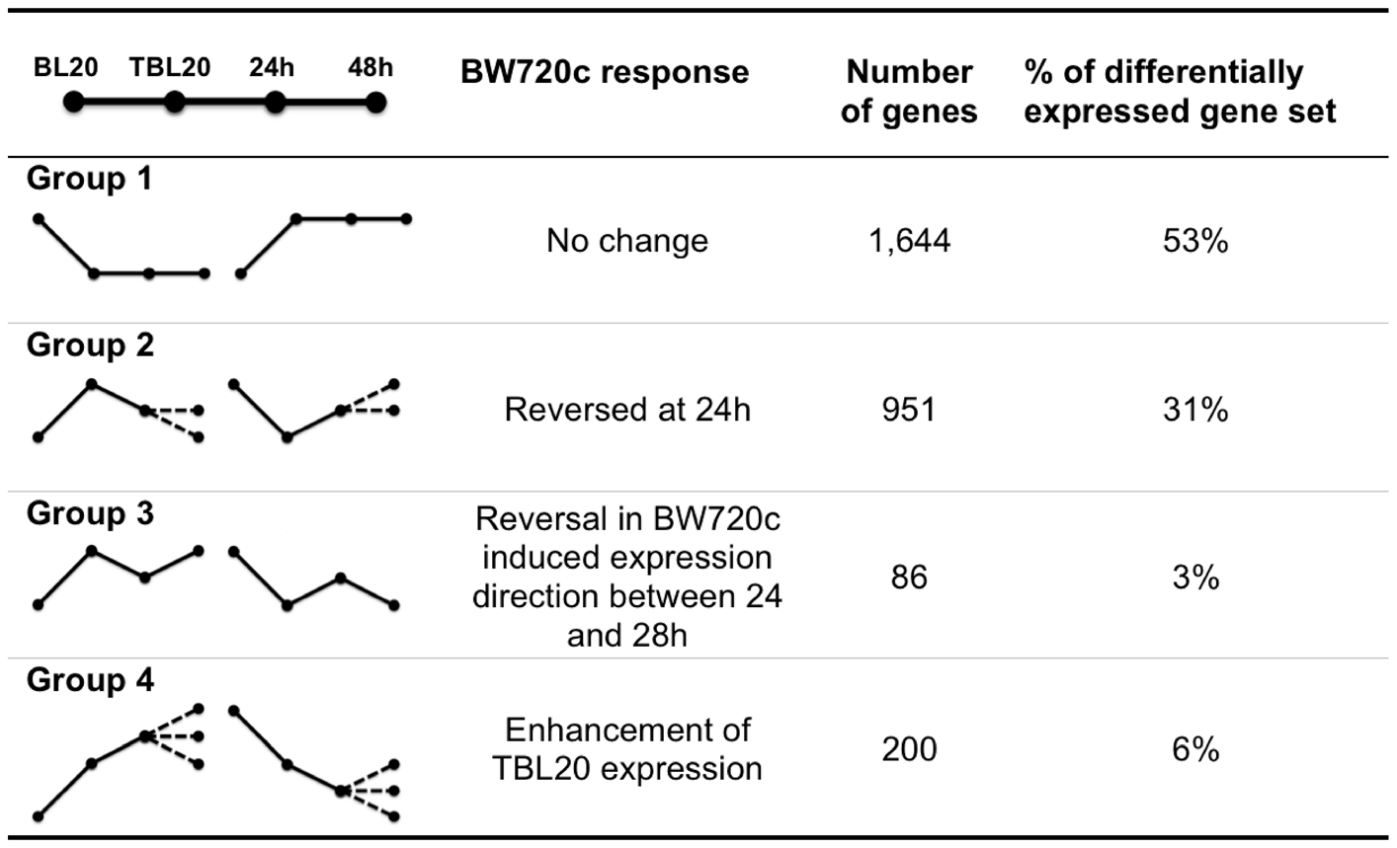
Main groups of gene expression profiles obtained over the BW720c time course. Profiles were derived from pairwise comparisons, BL20 vs TBL20; TBL20 vs TBL20_BW720c_24 h; TBL20_BW720c_24 h vs TBL20_BW720c_48 h as defined using RP with 5% FDR. The total number of differentially expressed genes in TBL20 compared to BL20 was 3,079. In addition to those genes exhibiting reversal in expression at 24 h, a further 303 (10%) showed reversal in the parasite induced expression profile at 48 h. We cannot distinguish whether these represent delayed reversal due to parasite death and hence associated with Group 2 or an apoptosis associated switch in expression of a gene that is non-responsive to parasite death (Group 3). Within Group 4, 30% showed enhancement in original parasite induced expression at 48 h only. Predominant trend in direction of expression in the group, solid line; alternative direction trend in the group, dashed line.

Following infection of the leukocyte it is possible that the parasite induces the expression of a key set of host genes that in turn lead to the activation/repression of pathways that are essential for survival/growth of the infected cell. Such genes might be predicted to be under direct control of infection and show an early response to loss of parasite viability relative to more delayed events that arise as a consequence of treatment (e.g. induction of cell death). To attempt to identify such genes and determine if TBL20 cells are characterised by a network of altered host cell gene expression that cannot be reversed on parasite death, expression patterns representing the response to BW720c treatment at 24 hours (early) and 48 hours (late) were derived. Genes differentially expressed between TBL20 and BL20 were broadly sorted into 4 main groups based on their response profile over sequential time points of BW720c treatment (Figure 3).

#### Group 1

no change in the parasite induced expression profile following BW720c treatment; this was the predominant group as it represented greater than 50% of the differentially expressed TBL20 dataset.

#### Group 2

reversal to a statistically significant degree following BW720c exposure for 24 h: this gene set was the next largest with 31% of differentially expressed genes showing this profile at 24 h. However, only a minority (18% of group 2) maintained this trend by 48 h. Thus for the majority of genes in group 2 the trend to revert towards expression values displayed by uninfected BL20 cells was not maintained between 24 and 48 h and these largely exhibited no further change between 24 and 48 h (76%). Furthermore, only a further 12% of genes altered by parasite infection initiated reversal at the 48 h time point. These results clearly indicate that the reversal process fails to complete, implying that it is overridden by a secondary change to factors controlling expression of these genes.

#### Group 3

genes that display a change between the 24 and 48 h time points in the initial direction of the expression pattern associated with BW720c treatment: 3% of the differentially expressed gene set displayed this pattern which may indicate, like group 2, a switch in gene regulation associated with initiation of secondary events in response to drug treatment, such as cell cycle arrest and cell death.

#### Group 4

enhancement of parasite-induced expression (elevation and repression) following BW720c exposure at 24 h and, in some cases, at 48 h. This pattern may represent a very early response to loss of parasite regulation. A similar profile has been described in other studies and was associated with repression of high level expression of genes induced by cellular activation, macrophage differentiation or the inflammatory response, that may be detrimental to the parasite infected cell [15], [17]. Taken together, the data indicated that only approximately 6% of differentially expressed genes in TBL20 cells show a sustained trend associated with reversion to the BL20 phenotype although >30% show evidence of immediate susceptibility to parasite death.

**Figure 4:**
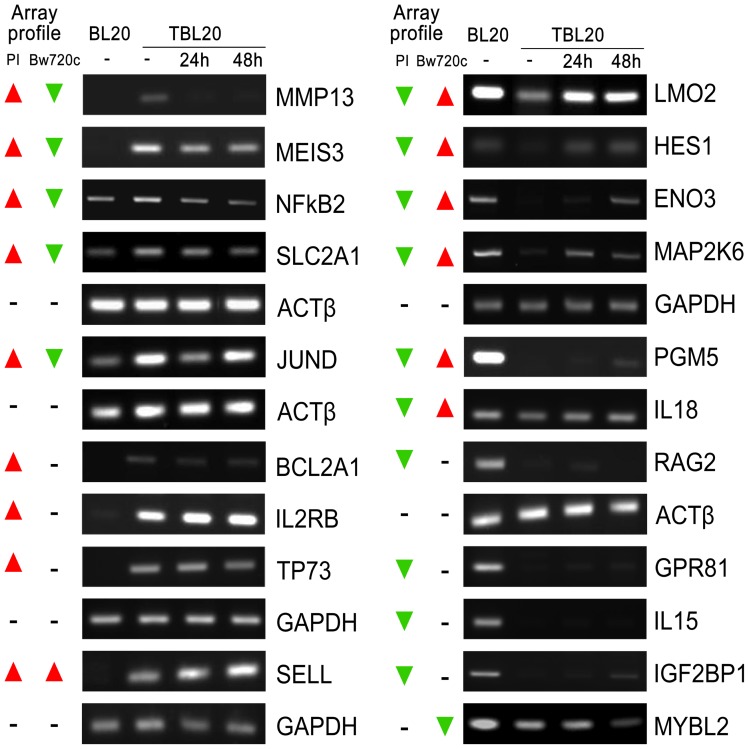
Semi-quantitative RT-PCR for selected bovine genes confirms the microarray expression data. Semi-quantitative RT-PCR carried out for a range of bovine genes to confirm the differential expressions determined from the microarray hybridisation and analysis of BL20 compared to TBL20 and for TBL20 following BW720c treatment. Actin or GAPDH were used as constitutively expressed reference genes and all genes tested showed similar profiles to hybridisation data from the array. The overall microarray profile is illustrated on the left of each panel; parasite infection (PI); buparvaquone (BW720c); elevated in TBL20 (▴); repressed in TBL20 (▾). BL20 and TBL20 without BW720c (−) and during BW720c treatment of TBL20 at 24 and 48 hour time points.

The microarray expression profiles of a range genes were selected for validation by SQRT-PCR and by QRT-PCR and these results are presented in Figure 4 and Figure S1. In general, there was good correlation with the microarray data and RT-PCR validations for specific genes are referred to as appropriate in the text.

To control for non-parasite specific effects of BW720c, uninfected BL20 cells were also treated with BW720c and RNA from 24 and 48 h time points analysed by microarray. Modulated expression of 563 genes at 24 h and a further 1121 by 48 h was detected (5% FDR) following exposure to the drug. Compared to the BW720c response in TBL20, very few showed large changes in expression (data not shown). Relatively few drug response genes were common to the infected and uninfected datasets (74 and 164 at 24 h and 48 h respectively) and they tended not to exhibit expression changes of similar magnitude e.g. genes encoding the G protein-coupled receptor, CX3CR1 and chemokine, CXCL10. These expression differences were confirmed by semi-quantitative RT-PCR (Figure S2). Thus the vast majority of changes to gene expression detected upon BW720c treatment of TBL20 (98% at 24 h and 95% at 48 h treatment) are associated with the response of the infected cell.

In conclusion, infection-associated changes to gene expression respond in a variable non-reversible manner following the loss of parasite viability. The obvious lack of uniformity in the transcriptional response of functionally important genes following BW720c inhibition of parasite viability would permit a drastic cellular imbalance to emerge and could lead to death of a previously immortal cell.

### Pathway analysis of BW720c responsive and non-responsive infection-associated changes to host cell gene expression

**Figure 5:**
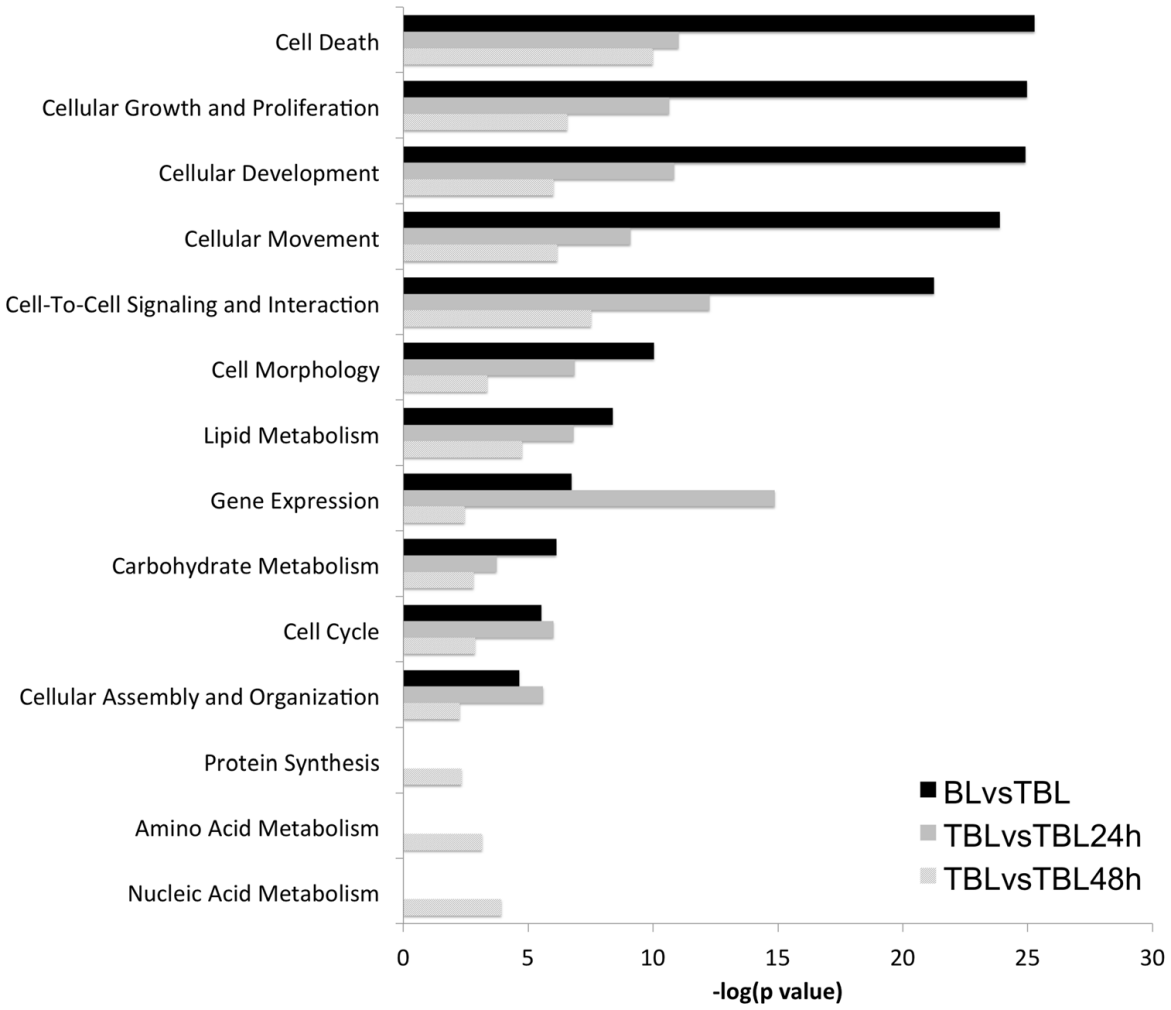
Differentially expressed genes in parasite infection and after BW720c treatment are enriched for fundamental molecular functions. A comparative analysis for enrichment in cellular molecular functions was carried out for each dataset. Fundamental cellular molecular functions contained within the IPA database and above the threshold for significance (-log(p value), p = 0.05) were selected for comparison. Black bars, enrichment inTBL20 compared to BL20; mid-grey, TBL20 at 24 h BW720c compared to TBL20 control; light-grey bars, TBL20 at 48 h BW720c compared to TBL20 control.

Changes to gene expression identified as an early (24 h) response to BW720c are most likely to highlight targets regulated by a parasite-dependent mechanism. To investigate whether this early response could be associated with any particular molecular function, pathway analysis performed for this dataset was compared to those of the complete infection-associated (IA) dataset and the BW720c dataset from the later (48 h) time point. This comparative analysis for enrichment in various Molecular Functions revealed a wide range of significant categories in all three datasets. A selection of the top-scoring functions is shown in Figure 5. Functions such as ‘Cellular Development’, ‘Growth and Proliferation’, ‘Cell Movement’, ‘Cell Death’, ‘Cell-To-Cell Signalling’, ‘Cell Morphology’ that scored highly in TBL20 compared to BL20 were also highly significant in BW720c treated TBL20. None of these categories, however, showed greater significance for the early (24 h) time point relative to the other two datasets. In contrast, genes encoding factors placed in the ‘Gene Expression’ category showed evidence of a more significant enrichment in the 24 h dataset, relative to the other two. This result was not observed in the study of Durrani *et al.* and suggests a marked alteration to expression of genes that function in regulation of gene expression following the initial loss of parasite viability at 24 h. A similar but less pronounced trend was also obtained for ‘Cell Cycle’ and ‘Cellular Assembly and Organisation’ two categories that can easily be linked to known parasite-associated alterations of host cell phenotype. For several functional categories (‘Amino Acid Metabolism’, ‘Nucleic Acid Metabolism’ and ‘Protein Synthesis’) significant enrichment of differentially expressed genes was only obtained for the BW720c 48 h gene set. The most straightforward interpretation of this result is that enrichment of genes in these categories is a strong indicator of a radical alteration to cellular metabolism that results from death of the parasite.

**Figure 6:**
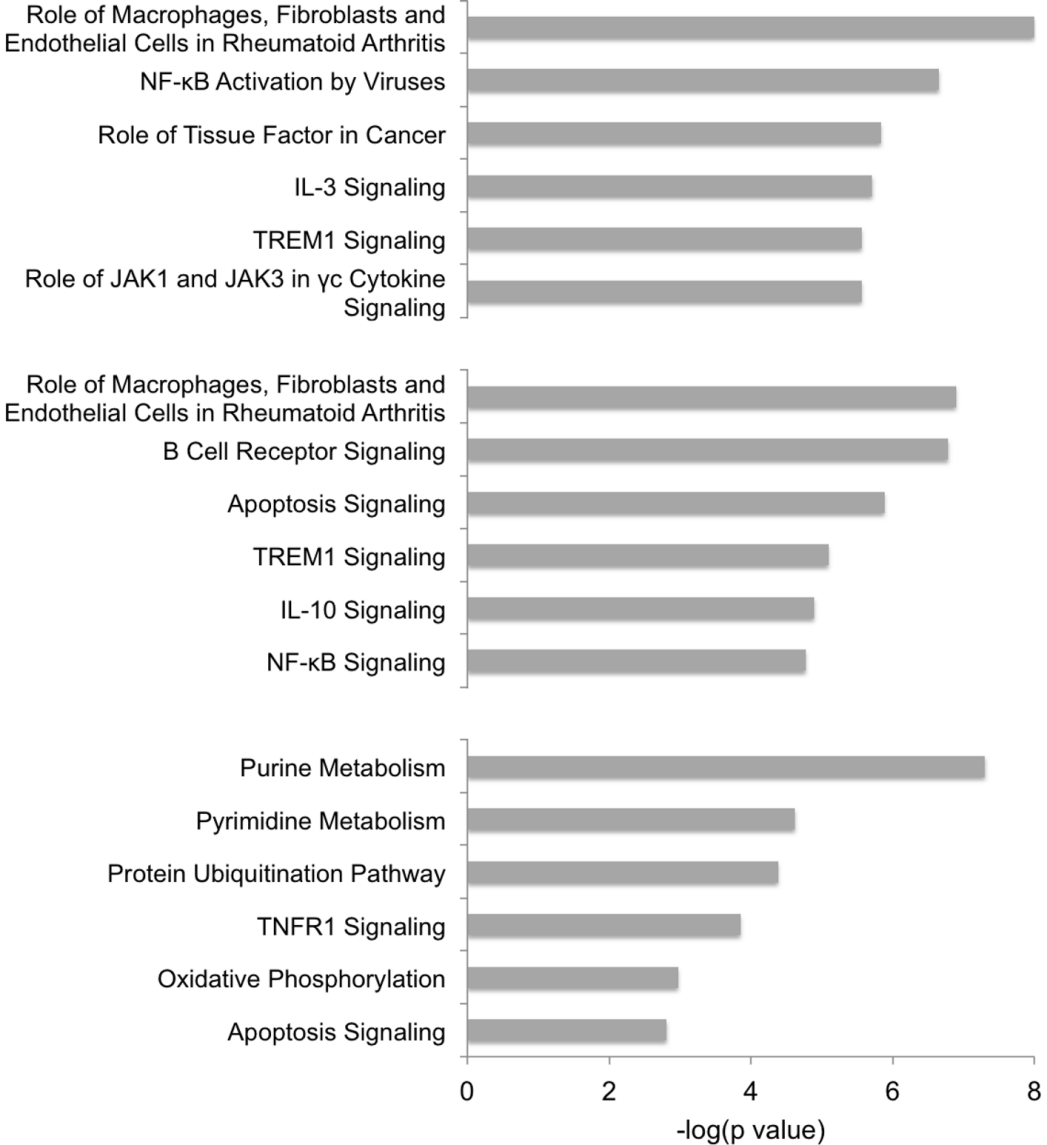
Enrichment in canonical pathways. Enriched Canonical Pathways for comparisons, BL20 vs TBL20; TBL20 vs TBL20_24 h_BW720c; TBL20 vs TBL20_48 h_BW720c were selected from the top 12 defined in each comparison. Top chart, BL20 compared to TBL20; middle chart, TBL20 at 24 h BW720c compared to TBL20 control; bottom chart, TBL20 at 48 h BW720c compared to TBL20. The significance of the enrichment is indicted in each case (-log(p value).

Analysis for enrichment in ‘Canonical Pathways’ indicated that pathways associated with Inflammatory disease and ‘NFκB’ activation by viruses', Cancer and IL3, TREM1, JAK1/JAK3 signalling were amongst the most highly significant in TBL20 compared to BL20 (Figure 6). The pathways ‘TREM1 Signalling’ and ‘Role of Macrophages in Rheumatoid Arthritis’ also appeared as highly significant in the TBL20 at 24 h BW720c dataset. These results were in broad agreement with pathway enrichment performed in the previous LPS study [22]. In general, signalling pathways predominated amongst those showing enrichment at 24 h BW720c and included enrichment in genes encoding molecules associated with ‘Apoptosis Signalling’, which was also evident at 48 h. The 48 h time point contrasted strongly with the TBL20 and TBL24 h BW720c datasets, as here the predominant enrichments were in pathways associated with basic cellular metabolism: ‘Purine and Pyrimidine Metabolism’, ‘Protein Ubiquitination’, and ‘Oxidative Phosphorylation’, indicating initiation of degradative cellular processes concerned with apoptosis. The data indicates that upon loss of parasite viability, alteration of signalling events are likely to occur prior to the initiation of pathways that result in cellular death and destruction.

While infection-associated genes that show an altered response upon BW720c treatment are more likely to be directly controlled by the viable parasite, it is clear from the analysis that a large number of differentially expressed genes were not altered following BW720c treatment. 697 were mapped in the pathway analysis framework and assigned to functional categories that can be linked to the transformed phenotype of the infected cell. The majority (n = 267, 38%), were classed as enzymes, but 91 (13%) were classified as transcriptional regulators; other defined functional classes were kinases, 9%; G-protein/transmembrane receptors, 9%; peptidase/phosphatase, 8% and transporters/ion channels, 17%. Only 3% were classed as cytokines/growth factors. Amongst non-functionally designated genes that do not exhibit any degree of reversal are the highly elevated BCL2A1 (43-fold, Figure S1), which is known to have important anti-apoptotic functions by inhibiting activation of the intrinsic mitochondrial cell death pathway (reviewed in [44]). Examples of other non-reversible genes that could be linked to the infected cell phenotype include the CDK inhibitors. There are two main groups of CDK inhibitors, the INK4 gene family and the CIP/KIP family. The INK4 family competitively inhibits the formation of active G1 phase CDK4/6-cyclin D complexes [45] and 3 of the 4 members exhibit repression in TBL20: CDKN2D (p19INK4d, 5-fold), CDKN2C (p18 INK4c, 3.1-fold), CDKN2A (p16INK4a, 2.9-fold) (Table S5). In TBL20, two members of the CIP/KIP family, p57 KIP2 (CDKN1C), and p27KIP1 (CDKN1B) are each repressed ∼3.5-fold. The CIP/KIP family of CDK inhibitors bind to a wide range of cyclin-CDK complexes to modulate activity towards substrates involved in cell cycle and in interconnected functions such as apoptosis (reviewed in [46]). In addition, this family also binds to/inhibits a range of other proteins, particularly those that regulate cytoskeletal dynamics, e.g. p27 with RhoA-GTPase, p57 with LIMK1 and p21 with Rho kinase ROCK1, reviewed in [47]. Notably, several *T. annulata* infected cell lines including TBL20 exhibit considerable change to cytoskeletal architecture compared to uninfected BL20 [25]. As none of the repressed CDK inhibitors exhibit significant reversal on BW720c treatment, it seems probable that in *Theileria* infection differential expression of the family of CDK inhibitors arises as a secondary event, possibly as a result of the activity(s) of transcription factors such as cMYC and FOXO3. cMyc is constitutively active in *Theileria* infected cells [11] and FOXO3 gene expression exhibits minor up-regulation in our array analysis. In addition, positive cell cycle regulators CDK14 and CDK3 also show altered (elevated) expression associated with infected TBL20 that did not reverse upon BW720c treatment. CDK14 has a possible role in the G1-S transition point. CDK3 is involved in the initiation of S phase, is known to phosphorylate retinoblastoma protein (pRb) and the transcription factor ATF1 [48]. Therefore, the balance between these positive and negative regulators of cell cycle progression is considerably altered in TBL20 and even though modulation of expression cannot be linked to the viable parasite, it is likely to contribute to the infected cell phenotype, the faster cell division rate for example. Within the dataset of genes not reversed by BW720c, there are many other similar examples of a potential association with the infected cell phenotype. A failure to reverse expression of such genes upon loss of parasite viability is likely to contribute to the failure to revert to the BL20 phenotype.

### Pathway analysis predicts altered production and perception of cytokines, chemokines and inflammatory mediators in TBL20

Both the TBL20 infection-associated and the BW720c 24 hr datasets showed significant enrichment for pathways associated with the inflammatory/immune response. Given the potential importance of these responses to survival of the infected leukocyte, genes encoding molecules that function to regulate the inflammatory/immune response were analysed in further detail. 52 cytokines/chemokines and growth factors were differentially expressed between BL20 and TBL20 (Table S6A). Approximately equal numbers were repressed (range: 3 to 60-fold) and elevated (range: 1.6 to 742-fold). Those highly repressed include IL15 (60-fold), TNSF11 (22-fold), CD70 (13-fold) and IL24 (6-fold). Five of this repressed group of 26 were reversed by BW720c treatment and three were enhanced. In addition, we found a 4-fold down-regulation of FASLG gene expression but a 3.5-fold up-regulation of the FAS receptor gene. Expression of FasLG and FAS have previously been reported in *T. parva* infected T-cells where the normally pro-apoptotic influence of FASL ligation is suppressed [33]. Elevated cytokines include TNFSF13B (20-fold) and IL7 (3.7-fold). Chemokine and chemokine receptor gene expression in the infected cell show considerable alteration. In general, expression of chemokine genes tend to be highly elevated (6 out 7 identified) e.g. XCL2 (742-fold elevated) and CCL1 (30-fold elevated) and all were highly responsive to reversal on exposure of TBL20 to BW720c. Overall, amongst differentially expressed cytokine genes, 60% of those elevated by parasite infection but only 23% of repressed, were reversed on BW720c treatment.

Modulation in expression of genes encoding polypeptides predicted to be located to the cell surface appears extensive. In the large families of G-protein-coupled receptors/transmembrane receptors/associated membrane localised kinases (167 genes), 74 are elevated and 93 repressed in TBL20 vs BL20. Included in this group are genes encoding the family of Toll receptors involved in perception of inflammatory stimulation. The gene for TLR4, the receptor for bacterial lipopolysaccharide is highly down-regulated, as to a lesser extent are genes encoding TLRs 1, 6, 8, 9 and 10 (range 3–70 fold) (Table 2 and Table S6B). All except TLR8 expression exhibit BW720c-induced reversal. In contrast to repression of Toll family receptor genes, expression of genes for the IL2 receptors IL2RA and B are both considerably elevated 25 and 80-fold respectively. The gene encoding the IL2 cytokine itself, however, is not differentially expressed and the level is predicted to be low, a scenario that is precisely as described for several *T. parva* infected cell lines, reviewed in [1]. Unsurprisingly, some of these modulations show similarity to those obtained in the previous LPS study [22] e.g. repression of IL15 and TLR4 and elevation in expression of XCL2, CCL1 and IL2RB were also detected in parasite-infected cells compared to LPS-stimulated BL20. Thus, these results indicate that multiple signalling pathways involved in perception of extracellular ligands are extensively modified in *Theileria* infected cells, as is expression of genes encoding molecules that signal to cells of the immune response. Moreover, it would appear that the *Theileria* infected cell has the capacity to reduce or enhance the ability to perceive a range of specific signaling ligands and it can be predicted that this may provide an advantage to the establishment and survival of the infected cell.

### Major reorganisation of transcriptional regulation is predicted to occur in Theileria infected leukocytes

The enrichment identified in the pathway analysis for the category ‘Gene Expression’ provided evidence that expression of molecules performing this function are likely to be under direct control of the parasite. Moreover, a high proportion of the functionally annotated differentially expressed gene set was classed as ‘transcriptional regulator’ (13%) and within ‘Gene Expression’ the sub-category ‘Transcription of RNA’ was of greatest significance (p = 1.34×10^−15^). We chose to investigate modulation of transcriptional regulators in greater depth because changes in the expression/activity of each one may result in multiple downstream effects on expression of their target genes and could, therefore, play a major role in orchestration of the gene expression network that characterises the infected TBL20 cell line. 177 transcription regulators were differentially expressed in TBL20 compared to BL20 (Table S7A). 68% (121) were elevated and 32% (56) were repressed. 40% of the elevated genes had statistically significant reversal in expression at 24 h while 31% showed reversal at 48 h. 25% of the repressed gene set showed elevated expression after 24 h BW720c treatment and 39% at 48 h. These results clearly indicate a bias towards up-regulation of host cell transcriptional regulators by parasite infection with a considerable number sensitive to loss of parasite viability. Interestingly, 8% of transcriptional regulators elevated in TBL20 exhibited enhanced expression in BW720c, including PAX6, TP63 and PARP12.

The most highly up-regulated transcription factors in TBL20 included MEIS3, FOSL1 (a subunit of AP1 with Jun), SSBP4, JUND, NFκB2, FOXA3 and CITED2, all of which showed reversal of the elevated expression with BW720c treatment (Table 3). Several others including FOSL2, TP73, LITAF and RUNX3, showed no alteration in expression following BW720c. Levels of HOXC13 and HOXB8, also elevated in TBL20, were enhanced by BW720c, Amongst those showing the greatest fold-change in the group with reduced expression in TBL20 were NEF2, LEF1 (lymphoid enhancer 1), HDAC9 (histone deacetylase 9) and KANK1 (Table 3). Repression of LEF1, HDAC9 and KANK1 expression was significantly reversed following 24 h exposure to BW720c suggesting that the viable parasite actively represses expression of pre-existing key transcription factors that might be detrimental to its growth and survival. Expression profiles of MEIS3, JUND, TP73 and LEF1 were validated by RT-PCR (Figure 4 and Figure S1).

**Table 3:** Transcription regulators elevated or repressed in TBL20 compared to BL20.

Symbol	Entrez Gene Name	ID	FC	BW720c response
MEIS3	Meis homeobox 3	617311	+65.2	−2.0
FOSL1	FOS-like antigen 1	531389	+37.3	−5.0
SSBP4	single stranded DNA binding protein 4	508156	+14.3	−1.7
JUND	jun D proto-oncogene	517192	+12.3	−2.5
NFKB2	nuclear factor of kappa light polypeptide gene enhancer in B-cells 2 (p49/p100)	526392	+9.4	−2.8
FOXA3	forkhead box A3	503622	+6.4	−1.6
CITED2	Cbp/p300-interacting transactivator, with Glu/Asp-rich carboxy-terminal domain, 2	521378	+5.9	−2.9
HOXC13	homeobox C13	538716	+16.0	+2.2
HOXB8	homeobox B8	785855	+5.1	+2.3
LITAF	lipopolysaccharide-induced TNF factor	520564	+116.2	
TP73	tumor protein p73	515105	+98.7	
ID3	inhibitor of DNA binding 3, dominant negative helix-loop-helix protein	538690	+58.1	
FOSL2	FOS-like antigen 2	509889	+12.2	
RUNX3	runt-related transcription factor 3	617389	+8.6	
PAX9		540196	+5.1	
LEF1		535399	−60.5	+3.6
HDAC9	histone deacetylase 9	535415	−36.8	+18.7
KANK1	KN motif and ankyrin repeat domains 1	534869	−34.3	+2.5
HEYL	hairy/enhancer-of-split related with YRPW motif-like	538609	−26.5	+2.7
SPIB	Spi-B transcription factor (Spi-1/PU.1 related)	784460	−17.8	+2.3
KCNIP3	Kv channel interacting protein 3, calsenilin	513316	−10.4	+2.9
PARP 14	poly (ADP-ribose) polymerase family, member 14	540789	−4.9	+5.5
NFE2	nuclear factor (erythroid-derived 2), 45kDa	514006	−78.8	
POU6F2	POU class 6 homeobox 2	517861	−27.8	
ZNF423	zinc finger protein 423	508025	−13.7	
EBF1	early B-cell factor 1	537712	−11.2	
HTATIP2	HIV-1 Tat interactive protein 2, 30kDa	539276	−11.0	
GCM2	glial cells missing homolog 2 (Drosophila)	539454	−10.8	
ZNF211	zinc finger protein 211	789989	−10.8	
POU6F2	POU class 6 homeobox 2	537815	−10.1	
NEO1	neogenin homolog 1 (chicken)	535307	−9.9	

Transcription regulators identified in the Ingenuity Pathway Analysis Database (IPA) that were differentially expressed in TBL20 compared to BL20 (FDR 5%). The fold change in TBL20 relative to BL20 is indicated; +, elevated in TBL20; –, repressed in TBL20. The fold change response in TBL20 treated with BW720c relative to TBL20 (FDR 5%) is indicated to the right. The top 15 ranking transcriptional regulations, both elevated and repressed, are shown grouped according to BW720c response.

Below we consider some of the notable changes to transcriptional regulators and the predicted consequences of these alterations in more detail.

### Homeodomain/HOX Family Proteins

141 genes encoding homeodomain-containing transcription regulators were represented on the microarray. This large family typically has important roles in pattern formation during development and in tissue regeneration and differentiation. 16 of these (11%) exhibit differential expression (5% FDR) between BL20 and TBL20. 4 members of the HOX family, are differentially expressed in TBL20; HOXC13 (15.9-fold) and HOXB8 (5.1-fold) are considerably up-regulated in TBL20 and show sensitivity (further elevation) to BW720c treatment (Table 3, Table S7A). Two, HOXC4 and HOXC9, are down-regulated by 5-fold and 3.6-fold respectively (Table S7A) but show no significant change on BW720c treatment. HOX transcription factors function as co-ordinators of developmental progression and in differentiation of tissues such as hematopoietic stem cells, where differential regulation is often associated with neoplasia [49]. Known co-activators/repressors are the TALE group of homeodomain proteins that include MEIS and PBX (reviewed in [50]) and SMAD. MEIS3 is up-regulated 65-fold by parasite infection and reversed with BW720c (confirmed by RT-PCR). A known direct target of MEIS3 is the 3-phosphoinositide-dependent protein kinase 1 (PDK1) involved in PI3-K-AKT signalling, engendering an anti-apoptotic role for MEIS3 [51]. Defined HOX targets are functionally wide ranging (reviewed in [52]) and include the CDK inhibitor p21, OPN (osteopontin), and bFGF (basic fibroblast growth factor). A function for HOXC13 in recognition/assembly of DNA replication origins has recently been described [53]. Other homeodomain family members that are differentially regulated include members of the POU and ZNF families. Therefore it is clear that *Theileria* infection imposes considerable adjustment to the BL20 homeotic gene expression profile.

Regulation of homeodomain gene expression and target genes of homeodomain transcription factors almost certainly involves chromatin remodelling, reviewed in [54]. There are also reports that transcription of HOX family members are subject to auto-regulation [49]. It is conceivable that these mechanisms and the known role of homeobox proteins to determine cellular fate, contribute to both reversible and irreversible alterations to host gene expression associated with *T. annulata* infection. For example, while our results indicate considerable change in the homeotic gene family expression profile, only a low number (n = 3) are directly responsive to parasite death. In addition, as might be expected of this fluid family, some, while not greatly altered by parasite infection, are elevated on BW720c treatment; PAX6 (up 5.3-fold), POU6F1 (up 3-fold) (Table S7A) and PBX1 (up 2-fold). Such altered expression on parasite death could clearly influence the inability to revert to a BL20 phenotype.

Expression of NANOG, also a homeobox transcription factor and predicted to be silent in BL20 (see Table S4A) was elevated by 2.3-fold in TBL20 and was responsive to BW720c. NANOG is associated with maintenance of cellular pluripotency, reduced levels being correlated with differentiation [55] and even minor changes in concentration can strongly influence differentiation status [56]. Thus, elevation of NANOG gene expression, particularly from a basal level, together with its sensitivity to BW720c, provides evidence that *Theileria* infection may re-define pre-existing chromatin structure possibly to effect alteration in differentiation status.

### Chromatin Structure Modifiers

**Figure 7:**
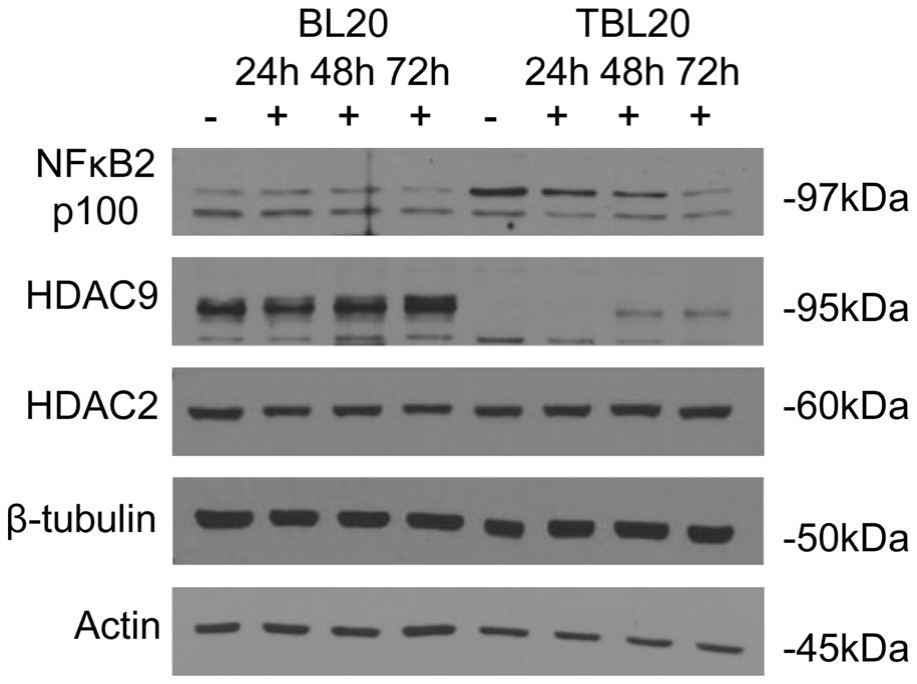
Microarray expression profiles of HDAC9 and NFκB2 genes are also reflected at protein level. Western analysis of total protein cell extracts from uninfected (BL20) and infected (TBL20) cells treated with BW720c for 0 (−), 24, 48 and 72 (+) hours. Equal protein loadings were verified by Ponceau S staining. Host HDAC9, HDAC2 and NFκB2 are shown relative to constitutive tubulin and actin. Antibodies are as described in Methods section.

Modification of histones is known to play a crucial role in determining chromatin structure and whether a gene is transcriptionally active or silent. We observed differential expression in three histone modifying enzymes. By far the most striking change was in histone deacetylase 9 (HDAC9) expression levels: 36-fold down-regulation with reversal on BW720c treatment (Table 3 and Table S7A). The microarray expression profile for HDAC9 was validated by Western blot analysis using an antibody specific for HDAC9. This confirmed that the 95 kDa polypeptide detected in uninfected BL20 was present at much reduced levels in parasite-infected TBL20 (Figure 7). An increase in the level of the polypeptide was observed following 48 h of BW720c treatment of TBL20 cells, exactly mirroring the RNA microarray profile. HDAC9 protein expression profile was compared with that of another histone deacetylase, HDAC2, predicted to be constitutively expressed from the microarray data and confirmed to be constitutive across the protein time course. In addition, we did not detect HDAC9 protein in two other *T. annulata* infected cell lines derived from *in vivo* infection of primary cells (data not shown).

Several members of PARP family, also modifiers of DNA binding proteins including histones and members of the HOX family [57], are differentially expressed in TBL20, examples are PARP11 and 14, repressed by 3.0 and 4.9-fold respectively and both reversed by BW720c. In contrast, expression of PARP12 was elevated 2.8-fold and elevation was enhanced 12-fold following BW720c treatment. Taken together these observations indicate that a general modulation of host cell chromatin structure is promoted by *Theileria* infection of the leukocyte. However, the strikingly large decrease in the level of one histone modifying enzyme, HDAC9, implies that HDAC9 may have a specific function that is incompatible with parasite survival, at least in the context of BL20 cells. While HDACs are normally associated with chromatin remodelling, there is increasing evidence that class IIa HDACs in particular, may have highly specific functions as essential co-repressors in regulation of specific gene targets. For example, a unique role for HDAC7 in repression of the gene encoding the cell cycle inhibitor RPRM has recently been identified that is not due to its deacetylase properties [58].

### Transcription factors constitutively activated by the Theileria infected leukocyte

It is well documented that infection of leukocytes by *Theileria* parasites results in constitutive activation of AP1, cMYC and NFκB. We predicted, therefore, that for these transcription factors the array data would show alteration to expression of genes involved in activation signalling or manipulation of the consequences of the activation event. JUN and FOS family members which heterodimerise to form the AP1 complex along with other components, exhibit considerable differential gene expression between BL20 and TBL20 and several are sensitive to BW720c treatment. JUN, JUNB and JUND are all elevated (Table 4) in TBL20 compared to BL20 (range 2–12 fold) and JUNB and JUND are both reversed with BW720c treatment. In contrast, JUN RNA shows a 5-fold enhanced elevation at 48 h BW720c, a likely indicator of the known role of AP1 in apoptosis (reviewed in [59]). Both up- and down-regulation are observed for FOS isoforms; FOS, FOSL1 and FOSL2 are elevated (range 4–37 fold) but FOSB is repressed. All show reversal with BW720c exposure except FOSL2. Earlier studies have indicated that while activity is increased, the AP1 composition is highly plastic in different *Theileria* infections [60] and is likely to impact on the expression profile of recognised target genes.

**Table 4:** AP1 sub-units with altered expression in TBL20 compared to BL20.

Gene symbol	FC in TBL20 vs BL20	Max BW720c response
JUNB	+2.0	−3.2
JUND	+12.3	−2.5
JUN	+1.7	+4.7
FOS	+4.0	−1.5
FOSL1	+37.3	−5.0
FOSL2	+12.2	
FOSB	−2.5 *	+4.9
ATF6	+2.5	−1.7
ATF7IP	−2.6 **	+2.6
MAF		+3.8
MAFF	+2.7	−3.2
MAFK		−1.8

Various components of the AP1 transcription factor complex that were elevated (+) in TBL20 or repressed (−) in TBL20 compared to BL20. The BW720c response is indicated to the right. The FDR generated by RP for FOSB* and ATF7IP** in TBL20 compared to BL20 were above our normal threshold of 5% but were included in this table as both showed a strong reversible BW720c response.

cMYC RNA and protein have been shown to be induced in a *T. parva* infected B cell line compared to primary B-cells and an anti-apoptotic role for cMYC protein in *Theileria* infected cells has been proposed [11]. In the BL20-TBL20 model, levels of MYC mRNA were not altered by parasite infection or influenced by BW720c, but were predicted to be very highly expressed, as was its partner in the active heterodimer, MAX. Interestingly, the highly expressed cMYC binding protein, MYCBP, did show slightly elevated expression in TBL20 (1.5-fold) that was reversed immediately by BW720c (1.6-fold at 24 h, rising to 2.1-fold at 48 h). The MYCBP gene encodes a protein that is a positive regulator of MYC. It binds to the N-terminal region of MYC and promotes E box-dependent transcription by MYC [61]. These results suggest cMYC activity or the spectrum of target genes may be manipulated in the infected cell by altered expression of binding partners.

Activation of NFκB is a central event in the establishment of the *Theileria* infected leukocyte [6], but it is not fully known whether the expression level of important components of this transcription factor complex is altered in the infected cell. Our study reveals a 9.3-fold up-regulation in NFκB2 mRNA (p100/p52) in TBL20 which showed reversal on BW720c treatment; and expression of RELB, which is typically the partner of NFκB2 in the active heterodimer, was up-regulated by 3.4-fold and responsive to BW720c. NFκB1 (p105/p50) was up-regulated by 2.1-fold but RELA (p65), the canonical partner of NFκB1 showed no differential expression between BL20 and TBL20. Up-regulation of NFκB2 and the decrease with BW720c was confirmed by SQRT-PCR (Figure 4), and was also established at the protein level by Western blotting (Figure 7). Interestingly, we could not detect proteolytic cleavage of precursor p100 to the active component, p52. This is most likely due to processing of small amounts, as is the case for most cell types [62]. An elevated level of NFκB2 mRNA and protein was also indicated for an *in vivo* derived infected cell line (data not shown). Alternatively, p100 may act predominantly as an inhibitory mechanism by forming high molecular weight complexes that bind pre-formed NFκB dimers thus serving as a molecular “buffer” to regulate availability of active NFκB for transcription [63]. BW720c sensitive nuclear localisation of NFκB2 was defined for *T. parva* infected cells in a study by [64], but they did not detect NFκB2 in complex with DNA. These results highlight the existence of transcriptional control mechanisms for components of the NFκB signalling pathway that are influenced by the presence of a live parasite and which are additional to the mechanism(s) that directly induce constitutive activation. The results imply alteration in the bias of sub-unit composition of the DNA binding complex, possibly reflecting alterations in substrate specificity to promote survival of the infected cell.

### The macroschizont-infected leukocyte manipulates the existing network of BL20 transcription regulators

To examine how extensively the presence of the *Theileria* parasite might influence the complex transcription factor network of immortalised BL20 cells, we grouped all transcriptional regulators in BL20 that were predicted to be highly or very highly expressed and examined how expression of this group was modulated by presence of the parasite. We found 688 genes designated as transcriptional regulators that were predicted to be highly expressed in BL20 (Table S8). Although 119 exhibited statistically significant differential expression in TBL20, only 7 transcription factor encoding genes were elevated more than 4-fold while 21 were down-regulated more than 4-fold, with 6 of these displaying >20-fold repression. Expression of 4 of these, HEYL, LEF1, HDAC9 (discussed earlier) and KANK1 was significantly reversed following exposure to BW720c suggesting that the viable parasite actively represses pre-existing expression of key transcription factor genes that might be detrimental to its growth and survival.

**Figure 8:**
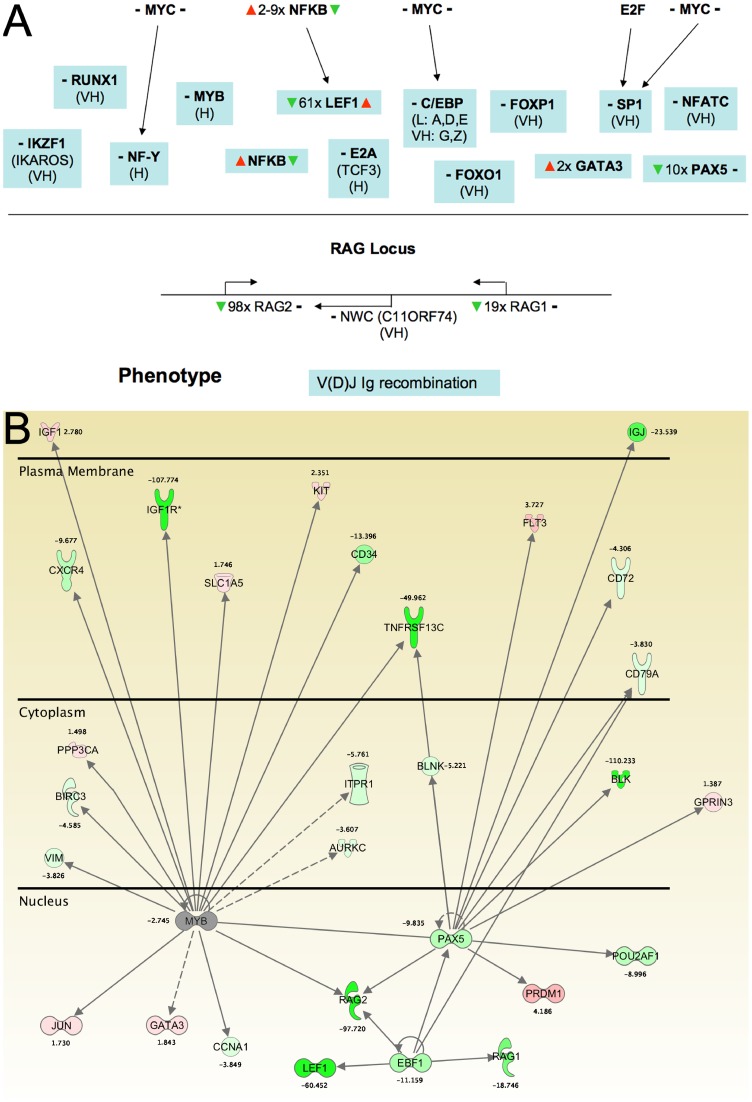
**A. Transcriptional control defined for the RAG1/2 locus showing points of regulation in Theileria infected cells** Prediction of expression level was as defined in the methods section; (VH), very high; (H), high. **–** to the left of the gene symbol, no predicted change in TBL20 compared to BL20. **–** to the right of the gene symbol, no change in BW720c. ▴**▾**, elevated or repressed in TBL20 (to the left) or BW720c (to the right) of the gene symbol. **B. Modulation of an established BL20 transcription factor network by parasite infection** Components of the MYB transcription factor network within different cellular compartments that show altered expression in TBL20 compared to BL20. Green, repressed expression in TBL20; red, elevated expression in TBL20.

Thus, the majority of transcription factor genes predicted to be highly expressed in BL20 cells do not appear to be modulated in the context of TBL20. Examples of these are MYB family members, RELA and members of the E2F and SMARC families. How might activity of transcription factors such as these be manipulated in the infected cell? To investigate how this could occur for the transcription factor MYB, whose expression is generally associated with myeloid cell lineages, we utilised the well-characterised transcriptional regulation defined for the RAG1/2 locus [65]. RAG1 and 2 are involved in regulation of recombination at the V(D)J Ig locus to generate immunoglobulin variation and both genes are highly repressed in TBL20. Transcriptional regulation of RAG1 and 2 occurs in a complex, defined hierarchy composed of minimally 14 transcriptional regulators, including MYB, and from our dataset it can be predicted that repression at the RAG1/2 locus could involve up to 5 transcription regulators that are modulated in TBL20 cells (Figure 8A), namely NFκB, LEF1, GATA3, PAX5 and EBF1, although only LEF1 and NFκB exhibited reversal of differential expression in the presence of BW720c. Therefore, while expression of MYB may be unaltered in TBL20 relative to BL20, a profound effect on expression of its target genes could occur via modulation of co-factors that operate in MYB functional networks. The possible consequences of such complex infection-associated manipulation of transcriptional regulation in pathways involved in myeloid cell lineage is illustrated in Figure 8B where TBL20 cells show modulated expression in a wide range of transcriptional regulators and their target genes. Several of these alterations have been validated by RT-PCR; MYBL2 and RAG2 (Figure 4), LEF1 and EBF1 (Figure S1).

### Prediction of transcription regulator activation status

Transformation of the *Theileria*-infected leukocyte is known to involve constitutive activation of host cell transcription factors, such as AP1 and NFκB. To obtain further information on the importance of these and other transcription factors modulated in infected TBL20 cells, a prediction of transcription regulator activation status was obtained for each dataset; infected cells compared to uninfected BL20 and BW720c-treated infected cells compared to non-treated. The analysis utilises data sourced from experimentally defined and predicted transcription factor target genes. Prediction of activation or inhibition relies on the proportion of target genes for any one transcription factor differentially expressed in a direction consistent with either activation or inhibition. The analysis predicted a wide range of transcription factors to be active or inhibited under all experimental conditions but also indicated that relatively few showed reversion of the infected cell activation or inhibition status when the parasite was killed (Table 5 and Tables S9A–C). Thus, the activity status of 7 transcription regulators in parasite-infected cells was predicted to be reversed during BW720c treatment (Table 5). SREBF1/2 (sterol regulatory element binding factor) and XBP1 (X-box binding protein) were all strongly predicted to be activated in TBL20 and inhibited in the presence of BW720c. XBP1 is now understood to be part of the unfolded protein stress response in the endoplasmic reticulum [66], and targets of XBP1 include several other transcription regulators including SREBF1.The majority of SREBF1/2 targets genes encode proteins located in the cytoplasm or cell surface and include SERPINE1 whose array expression profile (elevated 83-fold in TBL20 and repressed 4-fold in BW720c at 24 h) was confirmed by QRT-PCR (Figure S1).

**Table 5:** Transcription regulators where the predicted activation status is reversed following BW720c exposure.

Regulator	Predicted Activation Status in TBL20	Z score	P value	Target gene ratio	Predicted Activation Status in TBL20 24h BW720c	Z score	P value	Target gene ratio	Predicted Activation Status in TBL20 48h BW720c	Z score	P value	Target gene ratio
SREBF1	Activated	3.689	8.15×10^−6^*	28/40	Inhibited	−3.632	4.70×10^−5^*	23/30	Inhibited	−4.089	1.88×10^−6^*	28/39
SREBF2	Activated	3.165	1.08×10^−6^*	21/24	Inhibited	−2.874	9.10×10^−7^*	16/20	Inhibited	−3.330	1.74×10^−7^*	20/24
XBP1 (includes EG:140614)	Activated	2.652	6.98×10^−4^*	9/12	Inhibited	−3.294	2.60×10^−2^	19/22	Inhibited	−3.892	2.14×10^−3^	27/31
JUND	Activated	2.177	2.65×10^−4^*	6/17	Inhibited (JUN)	−2.579	1.37×10^−3^	20/43	-	-	-	-
NFkB (complex)	Activated	2.129	2.39×10^−19^*	74/135	Inhibited (NfkB1-RelA)	−2.070	1.16×10^−2^	6/6	-	-	-	-
TBX2	Activated	2.010	1.91×10^−1^	10/12	-	-	-	-	Inhibited	−2.731	1.16×10^−1^	11/12
CLOCK	Inhibited	−2.035	8.38×10^−5^*	26/34	Activated	2.236	1.12×10^−3^	19/24	Activated	2.268	1.80×10^−3^	22/28
ARNT2	Inhibited	−2.188	1.75×10^−1^	17/30	-	-	-	-	Activated	2.211	3.97×10^−1^	14/21
SIM1 (includes EG:20464)	Inhibited	−2.319	4.18×10^−1^	16/27	-	-	-	-	Activated	2.978	1.46×10^−1^	14/18
SMARCA4	Inhibited	−2.862	4.33×10^−12^*	18/72	-	-	-	-	Activated	3.127	2.04×10^−3^	15/44
IRF7	Activated?	1.349	1.85×10^−7^*	27/36	Activated	3.765	7.72×10^−8^*	24/30	Activated	4.289	5.08×10^−6^*	26/31
PPARGC1B	Activated?	1.885	1.02×10^−1^	7/7	Inhibited	−2.071	6.36×10^−5^*	10/11	Inhibited	−2.047	5.88×10^−4^*	10/11
SIRT2	Activated?	1.798	1.62×10^−3^	6/6	Inhibited	−2.271	1.92×10^−5^*	7/7	Inhibited	−2.251	9.90×10^−5^*	7/7
MYC	Uncertain	1.572	8.37×10^−8^*	9/16	Inhibited	−4.528	1.44×10^−3^	43/80	Inhibited	−5.396	2.20×10^−7^*	62/117
SMARCB1 **	No data	-	-		Activated	2.826	1.23×10^−1^	11/18	Activated	3.278	6.52×10^−3^	17/28

The predictions of activation or inhibition of transcription regulators are based on the direction of expression of differentially expressed known target genes collated in the IPA database. Cut-off criteria, Z score >2 for activation and <−2 for inhibition. * indicates those predictions that are most significant based on p value. Target gene ratio represents the number of genes expressed in a direction consistent with the prediction over the total number of target genes for that transcription regulator identified in the database. ** SMARCB1 is predicted to be activated at 24 *and* 48 h as a direct result of parasite inhibition by BW720c.

CLOCK (Circadian Locomotor Output Kaput), is a transcription factor involved in regulation of circadian rhythm and is now understood to be important in control of metabolic homeostasis [67]. Activity is predicted to be inhibited in *Theileria* infected cells and activated in the presence of BW720c. Differential expression of CLOCK targets include ICAM1 (elevated in TBL20 and other infected cell lines, reversed 2-fold at 24 h BW720c) and MAP2K6 (elevated 2-fold in TBL20 and reversed in BW720c). Both these profiles were validated by RT-PCR (Figure 4 and Figure S1).

SMARCB1 encoding BAF47, a component of mammalian SWI/SNF complexes which function in chromatin remodelling (reviewed in [68]), was the only transcription regulator predicted to be activated consistently at 24 and 48 h BW720c time points. Expression profiles of SMARCB1 targets that have been confirmed by RT-PCR include HES1, MYBL2 and MYOM1 (Figure 4 and Figure S1). Differential expression of the transcription factor, HES1 (hairy and enhancer of split in Drosophila), a target of the NOTCH signalling pathway and an important regulator of cellular status [69], is significant. HES1 is repressed 3-fold by parasite infection but shows an immediate and over-compensating elevation during the BW720c time course (12-fold by 48 h). Thus HES1 is likely to represent a key regulator of apoptosis in our model system. In addition to a role in apoptosis, HES1 has functions in proliferation and quiescence [70] and may provide a link between these closely integrated cellular processes.

A second subunit of the SWI/SNF complex SMARCA4, encoding BRG1, was also predicted to be inhibited in TBL20 and re-activated at 48 h BW720c. The function of SWI/SNF complexes in regulation of gene expression is believed to involve modification of nucleosome conformation to generate a chromatin structure that is more accessible for transcription. These predictions, together with differential expression of several genes encoding enzymes involved in modulation of chromatin structure, HDAC9 and PARP family members for example (see above), support the hypothesis that manipulation of the host cell by the *Theileria* parasite involves substantial modifications to host cell chromatin structure.

Compared to the 24 h BW720c time point, at 48 h substantially more transcriptional regulators were predicted to be activated/inhibited (Tables S9B and C), with 19/29 of the most significant (z-score <-2 or>2) for activation. These include the cyclin dependent kinase inhibitor CDKN2A and p53. Activation of CDKN2A is likely to result in inhibition of the kinase, CDK4 [71] and hence to reflect the phenotype of division arrest that occurs during BW720c treatment. Moreover, the predictions on transcription factor activation status after 48 h in BW720c strongly indicate progression towards apoptosis. Minimally these include the predicted inhibition of NRF2 activity [72], activation of p53, (reviewed in [73]) and inhibition of several members of the E2F family transcription factors which have varied roles in promoting cell cycle progression and in apoptosis (reviewed in [74]).

In support of our predictive analysis, the NFκB complex, well established by experiment to be activated in *Theileria* infected cells, was scored as active in TBL20. Inhibition of NFκB activity was predicted at 24 h BW720c but interestingly this was restricted to the RELA component where relatively few targets are identified. Expression profiles of target genes MMP13 and ICAM1 have been confirmed by RT-PCR (Figure 4 and Figure S1). Therefore, the analysis highlights alterations to selected NFκB complex components very early in the response to the loss of parasite viability. General activity of the NFκB complex was strongly predicted to be retained following BW720c treatment and may function in pathways leading to cell death. This prediction is also illustrated by separate analysis of expression changes in known NFκB target genes (data not shown) where only a proportion show reversal in expression following BW720c exposure. Previous studies have demonstrated that while EMSA for NFκB showed markedly reduced levels of one specific mobility shift complex upon BW720c treatment, a second complex was still clearly detectable at 48 hr [6]. Components of the AP1 complex were also predicted to be active in TBL20 and following BW720c treatment. Thus, overall this analysis clearly predicts that following the loss of parasite viability the activation status of host cell transcription factors does not reverse to the profile displayed by the uninfected BL20 cell line.

### Summary Discussion

This study has shown that the presence of the macroschizont of *Theileria annulata* can dramatically alter the transcriptome of an immortalised host cell line, with over 3,000 host cell gene expression changes associated with the presence of a viable parasite. Moreover, by exposing *Theileria*-infected TBL20 cells to BW720c, it was found that the host cell undergoes cell death and does not revert to a proliferating BL20 cell, indicating that the parasite can irreversibly alter the immortalised phenotype. This was mirrored by the failure to reverse infection-associated changes to gene expression in multiple cellular processes. The majority showed no response to BW720c treatment, a result also obtained in our previous analysis of the more limited LPS-modulated gene set [22]. We believe that this profile is most likely due to the generation of irreversible alterations to host gene expression that occur during establishment of the proliferating infected cell. However we cannot discount that some of these changes may be due to epigenetic events that occur during growth of infected cell lines *in vitro*. Further work is required to distinguish between these possibilities. Moreover, it is also evident that infection-associated gene expression changes that display a BW720c response do not always reverse: some genes showed an enhanced infection-associated profile on parasite death, while other genes not showing differential expression between infected and uninfected cells were modulated when the parasite was killed. The remainder, in the main, showed a transient alteration that either stalled or displayed a directional change in response after 24 h of drug treatment. These changes could be explained by a major reorganisation of factors controlling the host cell gene expression network that is initiated following loss of parasite viability, leading to a switch in favour of progression towards a cell death programme over division.

Pathway analysis for genes that showed an early (24 h) response to loss of parasite viability highlighted those within the category ‘gene expression’. This included a significant number of predicted transcription factors and proteins that modify chromatin architecture. In support of these findings, a recent study has revealed that the chromatin modifying histone methyl transferase, SYMD3 is up-regulated in *Theileria* infected cells and associated with generation of expression changes at the MMP9 promoter [36]. Our analysis also highlighted factors that operate to determine cell fate and revealed that the activation status of transcription factors such as NFκB and AP1, known to be involved in generating the transformed infected cell phenotype, did not show an expected reversal on parasite death but rather prediction of qualitative changes to subunit composition. These results imply that parasite infection may target regulators of the host cell gene expression network as a primary mechanism to alter host cell phenotype. Altered expression of factors (HOX, LMO2, RUNX and SMAD) and networks (MYB) involved in regulation of haematopoetic cell fate decisions [75] is clearly evident, supporting previous work indicating that parasite-mediated transformation is associated with a de-differentiated myeloid type cell [17]. It is accepted that transcription factors and modulation of chromatin status operate to produce stable changes to cellular phenotype [54], [75]. Thus, it is possible that modulation of these events in the *Theileria* infected cell contribute to the reconfiguration of the bovine cell regulatory network and promote an irreversible change to the resulting phenotype. Moreover, for janus type transcriptional regulators such NFκB, retention of activity on BW720c treatment but loss of cofactors that selectively modulate target gene expression, could switch a pro-survival function to that of pro-death.

**Figure 9:**
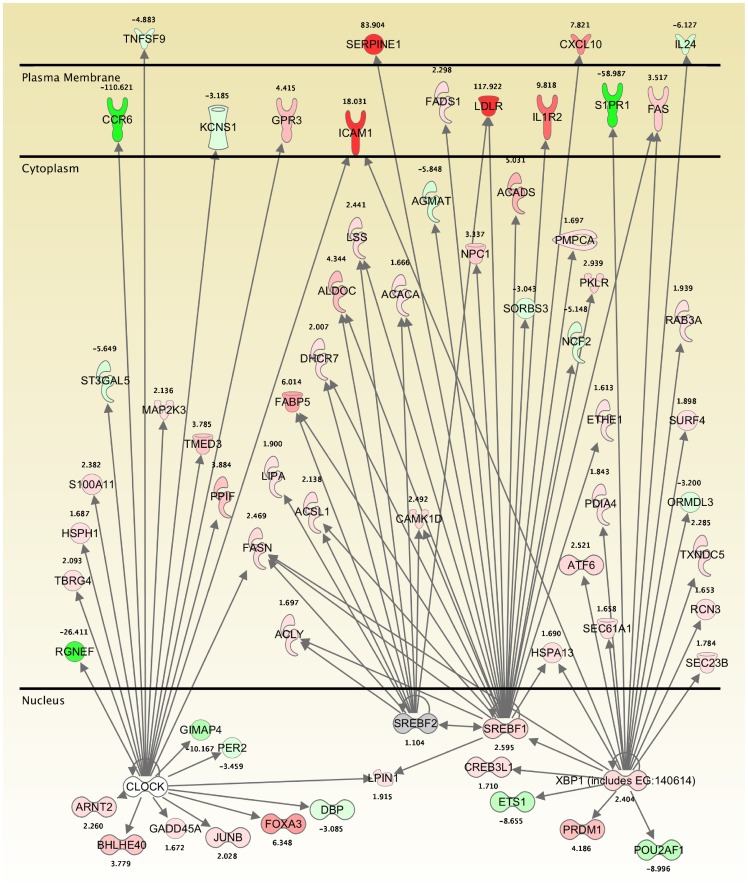
CLOCK, SCREB and XBP1 transcriptional regulators, predicted to have altered activities in TBL20 that are reversed in the presence of BW720c, have target genes in common. Illustration of the target gene network from our analysis where differential expression may result from activation or inhibition of 3 transcription regulators, CLOCK, SCREB and XBP1, each with activation status predicted to be altered by parasite infection and reversed by BW720c in our analysis (see text). Red means up-regulated in TBL20 compared to BL20 (range 1.7–117 fold); green is down-regulated in TBL20 (range 1.7–110 fold); grey, no differential expression.

An interesting question is whether the plethora of infection-associated changes can be attributed to a few or many primary events. While our study cannot answer this question with clarity it does highlight the complexity of changes that can potentially result from modulation of a small number of key regulators. For example, analysis utilising a relatively small number of known bovine NFκB target genes indicated that 17 out of the 95 that are modulated in the infected cell encode transcription regulators and manipulation of just three transcription factors, CLOCK, SREB1/2 and XBP1, which were predicted to display altered activity in the infected cell, has potentially widespread consequences (Figure 9). Moreover, it is becoming clear that the target range of some transcription factors is likely to be much larger than was previously understood; a recent estimate for NFκB suggests more than 3,000 binding sites on mammalian DNA [76], <10,000 are indicated for the myeloid lineage specific transcription regulator [77] and a minimum estimate of 600 for MYC [78]. Target ranges of this magnitude, in combination with modulation of chromatin architecture, stimulation from exogenous cytokines/chemokines, modulation of the NFκB activation response by a *Theileria*-dependent mechanism [22] and export of parasite-derived proteins with DNA-binding properties to the host cell nucleus [14] are likely to result in major reconfiguration of the host cell transcriptome. Such a model is not dissimilar from events proposed for related apicomplexan parasites. A global microarray analysis following infection of human fibroblasts with *Toxoplasma gondii* revealed a comparable number of changes to host cell gene expression [79] and evidence for *T. gondii*-mediated modulation of host cell transcription factor activation and manipulation of chromatin structure has been reported [80]–[82]. These studies reveal the extent to which intracellular apicomplexan parasites can re-organise the gene expression network of their host cells. In addition, it is intriguing to speculate whether apicomplexan parasite models could contribute to our understanding of the changes to mammalian cell transcriptomes that are associated with disease such as inflammatory disorders and neoplasia.

## Supporting Information

Figure S1 QRT-PCR vs microarray for selected genes.
(PDF)

Figure S2 Semi-QRT-PCR for BW720c treated BL20 and TBL20.
(PDF)

Table S1 Primers pairs for RT-PCR.
(PDF)

Table S2 Validation of microarray expression from literature.
(PDF)

Table S3 (PDF)

Table S4 (PDF)

Table S5 Cell cycle associated genes.
(PDF)

Table S6 (PDF)

Table S7 (PDF)

Table S8 Transcription regulators predicted to be highly expressed in BL20.
(PDF)

Table S9 Transcription regulators predicted to be either activated or inhibited in untreated TBL20 (A) OR at 24 h (B) OR 48 h (C) following exposure to BW720c.
(PDF)
