# Supplementary figures and images for: A Bovine Lymphosarcoma Cell Line Infected with Theileria annulata Exhibits an Irreversible Reconfiguration of Host Cell Gene Expression
Source: PLoS One. 2013 Jun 26;8(6):e66833. doi: 10.1371/journal.pone.0066833 (PMC3694138; doi:10.1371/journal.pone.0066833)

Figure S1: QRT-PCR vs microarray for selected genes

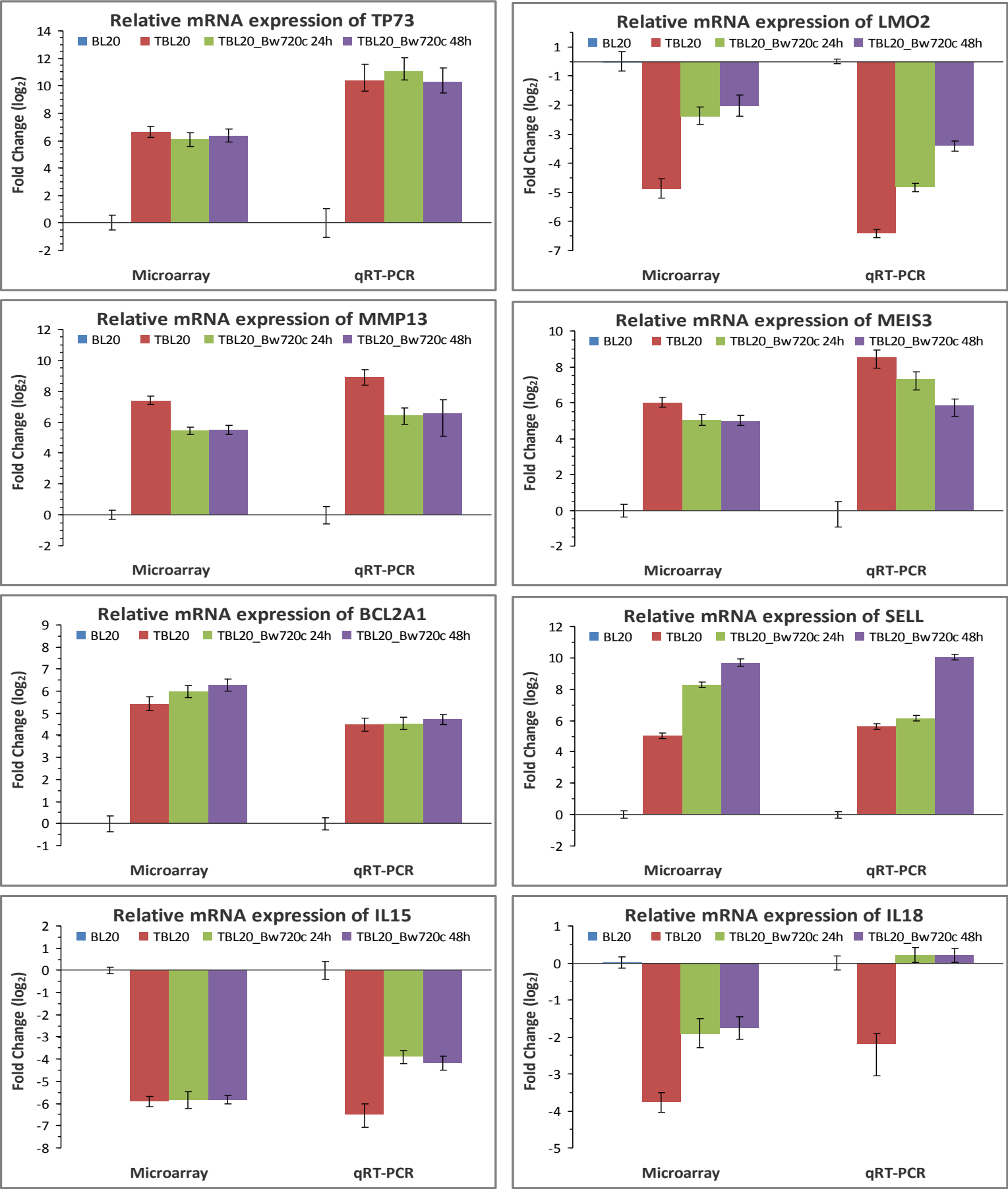

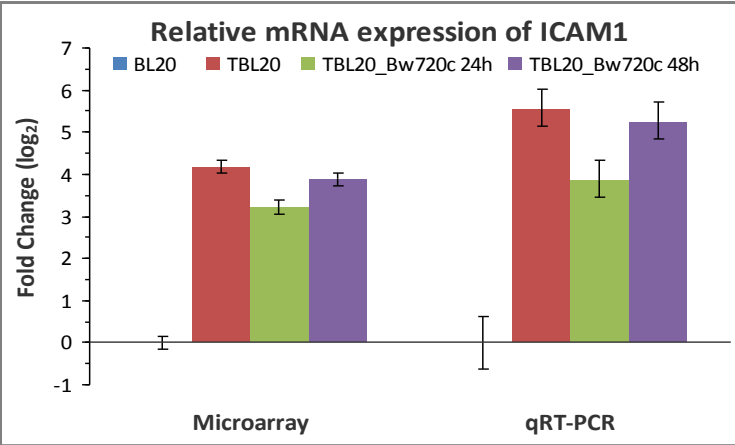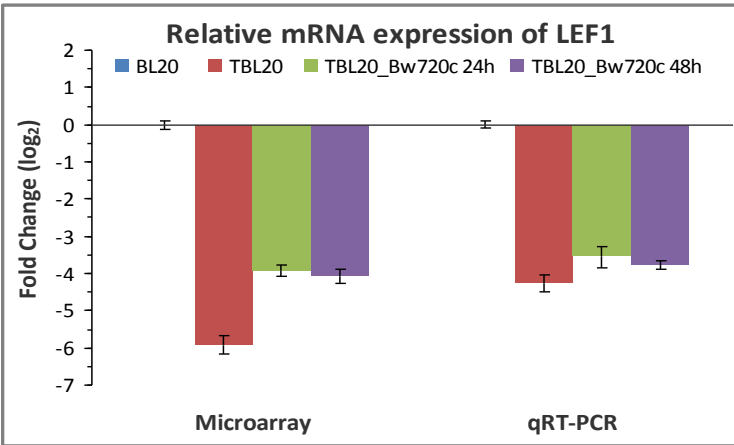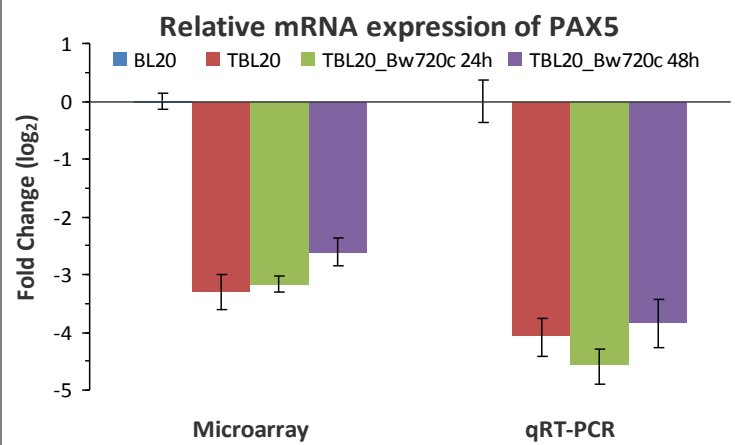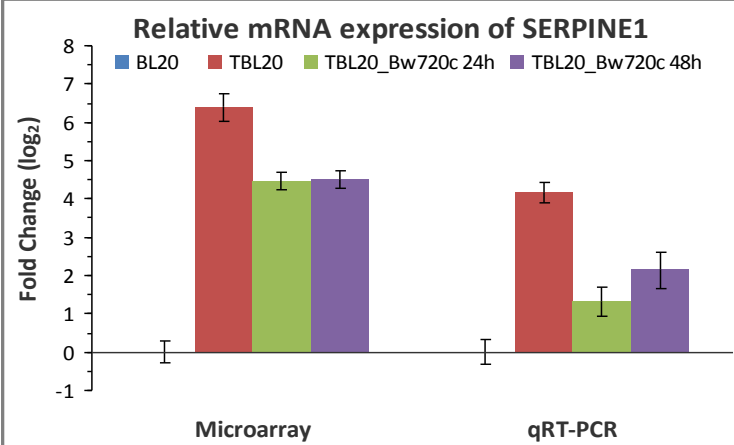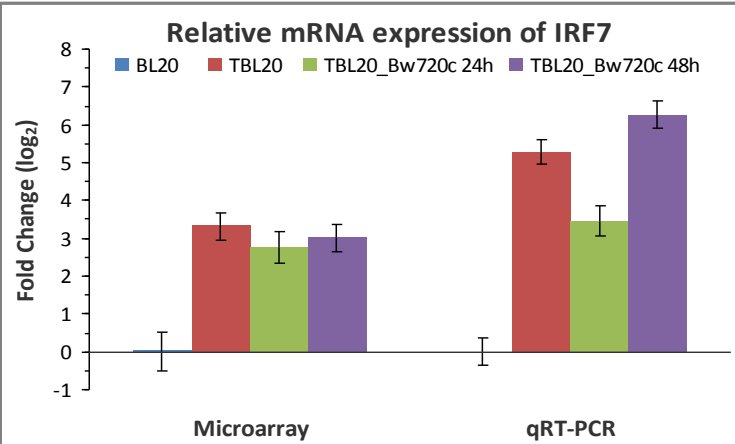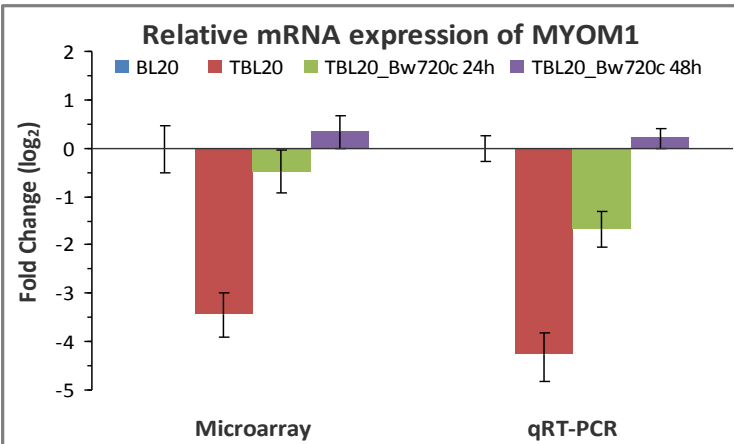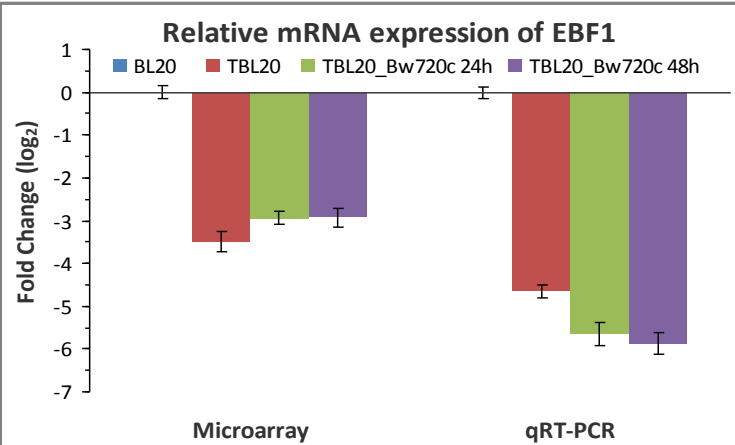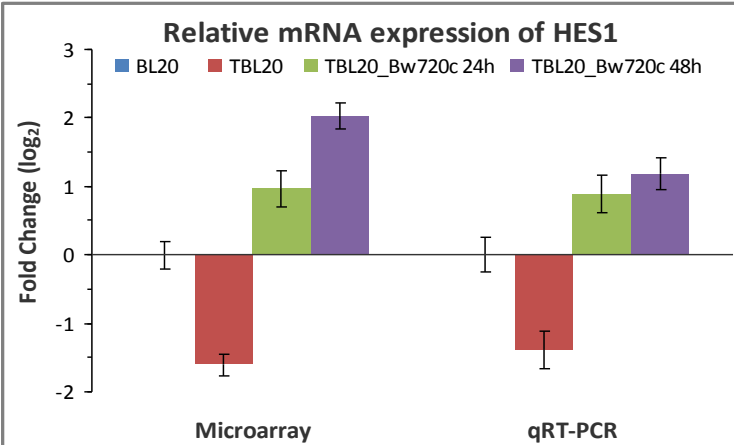

Supplement: Figure S1 — QRT-PCR vs microarray for selected genes. (PDF) [file pone.0066833.s001.pdf]

**Figure S2: Semi-QRT-PCR for BW720c treated BL20 and TBL20**

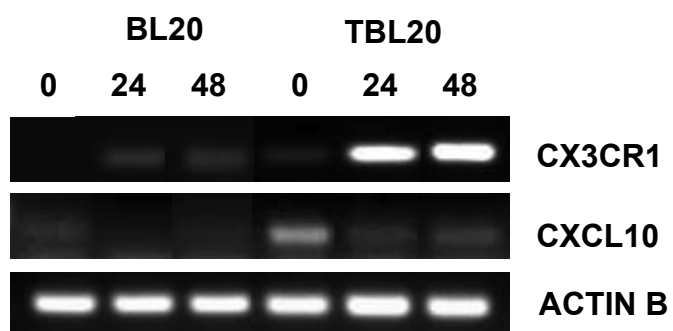

Supplement: Figure S2 — Semi-QRT-PCR for BW720c treated BL20 and TBL20. (PDF) [file pone.0066833.s002.pdf]
