# Supplementary material for: A Bovine Lymphosarcoma Cell Line Infected with Theileria annulata Exhibits an Irreversible Reconfiguration of Host Cell Gene Expression
Source: PLoS One. 2013 Jun 26;8(6):e66833. doi: 10.1371/journal.pone.0066833 (PMC3694138; doi:10.1371/journal.pone.0066833)
Supplement: Table S1 — Primers pairs for RT-PCR. (PDF) [file pone.0066833.s003.pdf]

Table S1: Primers pairs for RT-PCR

Symbol	Entrez Gene Name	Entrez Gene ID	SEQ_ID	RefSeq	Primer Pair sequences	Tm	Length (bp)
ACTB	actin, beta	280979	gi_75832053	NM_173979.3	F^{871}CTCTCTTCCAGCCTTCCTTCCTGG^{894}	58.2	240
					R^{1110}ACACGGAGTACTTGCGCTCAGG^{1089}	58.9	
MMP13	matrix metallopeptidase 13 (collagenase 3)	281914	gi_31342133	NM_174389.2	F^{516} TGGTGACTTCTACCCATTTGATGG^{539}	54.6	275
					R^{790} ACTGAATCCCTTGGACATCATCATC^{766}	54.7	
IL2RB	interleukin 2 receptor, beta	510185	gi_119894051	XM_587301.3	F^{228}ACTCACTCCAGGTCGTCCGCATAG^{251}	59.8	217
					R^{444}ACCTGAAGCTCATATTCCGTGCCTG^{420}	58.9	
MEIS3	Meis homeobox 3	617311	gi_119910732	XM_869544.2	F^{203}CGCTGCTAGCCCTGGTCTTTGAG^{225}	59.6	232
					R^{434}AGGTGGAAGCGGAGCACTTGAATG^{411}	59.5	
CXCL10	chemokine (C-X-C motif) ligand 10	615107	gi_114052748	NM_001046551.1	F^{214}TGCAAGTCAATCCTGCCCACGTG^{236}	59.9	203
					R^{416}GAGGTAGCTTCTCTCTGGTCCATCC^{392}	57.7	
CX3CR1	chemokine (C-X3-C motif) receptor 1	100124525	gi_156523227	NM_001102558.1	F^{27}AGGAAGACTGACGGGCACATCG^{48}	58.7	243
					R^{269}TTGATGAGGGCAAACACCACCAGC^{246}	59.9	
LMO2	LIM domain only 2 (rhombotin-like 1)	614876	gi_115496605	NM_001076352.1	F^{655}CTACTACAAGCTGGGCCGGAAGC^{677}	59.3	217
					R^{871}CACGATGTCGGAGTTGATGAGGAGG^{847}	59.0	
PGM5	phosphoglucomutase 5	785045	gi_156121314	NM_001102335.1	F^{143}TGCCCAACTTCATCCAGAGCGTGC^{166}	61.8	295
					R^{437}GCAACATTAAACTTCACTCCAAACTCTCC^{409}	57.1	
TP73	tumor protein p73	515105	gi_119927959	XM_593064.3	F^{2210}AGGCTGGTCCCTCTGCTGGTC^{2230}	59.9	253
					R ^{2462} AGGAACCTGTGTCCGTGCTCA^{2442}	57.8	
NFKB2	nuclear factor of kappa light polypeptide gene enhancer in B-cells 2 (p49/p100)	526392	gi_156120848	NM_001102101.1	F^{252}CCAGAGTCATGGACAGTTGC ^{271}	60.4	437
					R^{688}TTTGGTCACATGGAGGACAC ^{669}	59.6	
IGF2BP1	insulin-like growth factor 2 mRNA binding protein 1	516853	gi_119912092	XM_595012.3	F^{1301}TCGCCAGCGCCTCTATCAAGA ^{1321}	57.8	271
					R^{1571}GGCACCACCACCTCAGCTGC^{1552}	60.3	
SLC2A1	solute carrier family 2 (facilitated glucose transporter), member 1	282356	gi_31341556	NM_174602.2	F ^{105}CGGGATCCACAGAGCGCAGC^{124}	60.2	109
					R^{213} CCAGCATGAGGCGGCCTGTC^{194}	60.1	
ENO3	enolase 3 (beta, muscle)	540303	gi_77736348	NM_001034702.1	F^{29} AGAGCCCAGCGAAGACATCCC^{49}	58.3	364
					R ^{392}GCCCAGGATGGCATTGGCCC^{373}	60.4	
BCL2A1	BCL2-related protein A1	282151	gi_115432056	NM_001037100.2	F^{304}TGTGGCTTCCTCTGTCCAGGAC^{325}	64	363
					R^{666}ACTTCCAGAAAAGTCAGCCAGCCA^{643}	62.7	
MAP2K6	mitogen-activated protein kinase kinase 6	286883	gi_77404210	NM_001034045.1	F^{120}GTCCATCTGCTGCCTCGGTCAAG^{142}	66.0	398
					R^{517}GATCCGCTTCACCGCCATGATC^{496}	64.0	
IL18	interleukin 18 (interferon-gamma-inducing factor)	281249	gi_31342868	NM_174091.2	F^{8}TGCTCTCCAATGCTTTCAGCGC^{29}	62.1	151
					R^{158}TGCAGCCATCTTTATGCCTGTGC^{136}	62.4	
IL15	interleukin 15	281248	gi_41386773	NM_174090.1	F^{79}ACAGAGGCTGGCATTCATGTCTTCA^{103}	63.0	204
					R^{282}CTCCAGGAGAAAGCACTGCATCG^{260}	64.2	

Table S1: Primers pairs for RT-PCR (continued)

GPR81	G protein-coupled receptor 81	538759	gi_76638859	XM_582510.2	F^{742}CCCTCCAGCGCCTGCAATCC^{761}	65.5	197
					R^{938}GTGGGCATCCCCTCTGGCCT^{919}	65.5	
GAPDH	glyceraldehyde-3-phosphate dehydrogenase	281181	gi_77404272	NM_001034034.1	F^{927}CAGCGACACTCACTCTTCTACCTTC^{951}	57.0	194
					R^{1120}GAACTCTTCCTCTCGTGCTCCTG^{1098}	56.7	
HES1	hairy and enhancer of split 1, (Drosophila)	539547	gi_77736300	NM_001034678.1	F^{262}GTGGCTGCTACTCCAGCCAGTG^{283}	59.5	268
					R^{529}CTGTGCTTAGCGCGGCCGTCA ^{509}	62.3	
SELL	selectin L	281485	gi_27901800	NM_174182.1	F^{620}ATGCAGCGGCCATGGACAATGTG^{642}	60.6	211
					R^{830}AATCATGTCTGTTCCCTTGGAGCAG^{806}	57.0	
LEF1	lymphoid enhancer-binding factor 1	535399	gi_119893570	XM_615475.3	F^{890}GTCTCCGGGTGGTGTTGGACAG^{911}	59.1	103
					R ^{992}CAGTGAGGATGGGTAGGGCTGC ^{971}	58.7	
PAX5	paired box 5	538371	gi_119900560	XM_618574.3	F^{1053}TGTGCCGAACGGCCACTCAC^{1072}	59.9	386
					R^{1438}CCATCCCTGTCAGCGTCGGC^{1419}	59.8	
EBF1	early B-cell factor 1	537712	gi_119895769	XM_617891.3	F^{1277}ACACCTCGGTCCATGCGGGA^{1296}	59.9	290
					R ^{1566}GGGGCTAGATGGCACGATGGC^{1546}	59.7	
SERPINE1	serpin peptidase inhibitor, clade E (nexin, plasminogen activator inhibitor type 1), member 1	281375	gi_31342766	NM_174137.2	F^{128}ATGTCTCCGGTCTTTGCCTGCCTC^{151}	60.8	346
					R^{473}CGGCTGTGCTGATCTCATCCTTGTTC^{448}	59.9	
MYOM1	myomesin 1, 185kDa	538404	gi_119916232	XM_618609.3	F^{4077}CTACGTGGATGATCTGAAAGTTAACTGG^{4104}	55.8	186
					R^{4262}TCCACCATCTTCTGGTGTCCGG^{4241}	57.9	
ICAM1	intercellular adhesion molecule 1	281839	gi_31342240	NM_174348.2	F^{594}TGGCCCTGCTCGGGACTCTG^{613}	60.3	191
					R^{784}AACCTTCCAGTTGTCCCCACGG^{763}	58.7	
IRF7	interferon regulatory factor 7	100125591	gi_157279988	NM_001105040.1	F^{970}CCAGGGACCTCTTGATCCAGGC^{991}	58.4	265
					R^{1234}CGGCCCTTATACAGGATGCTCAAG^{1211}	57.2	
JUND	jun D proto-oncogene	517192	gi_157168340	NM_001103253.1	F^{1205}AGGGCGCCTCAGACTCGACA^{1224}	59.6	125
					R^{1329} GCGGGTTTGTGCAACACGGG^{1310}	59.6	
MYBL2	v-myb myeloblastosis viral oncogene homolog (avian)-like 2	510420	gi_115495776	NM_001075448.1	F^{422}CCAGCCACTTTCCCAACCGCA^{442}	59.8	151
					R^{572}AGCGTCCACTGCTTCGTGCC^{553}	59.9	
RAG2	recombination activating gene 2	782387	gi_126723156	NM_001082469.1	F^{379}GGCCACTTGCACATTCAGCGG^{399}	59.2	317
					R^{695}GGGGCAGGCAGTCAGCTACAC^{675}	59.5	
