# Supplementary material for: A Bovine Lymphosarcoma Cell Line Infected with Theileria annulata Exhibits an Irreversible Reconfiguration of Host Cell Gene Expression
Source: PLoS One. 2013 Jun 26;8(6):e66833. doi: 10.1371/journal.pone.0066833 (PMC3694138; doi:10.1371/journal.pone.0066833)
Supplement: Table S2 — Validation of microarray expression from literature. (PDF) [file pone.0066833.s004.pdf]

Table S2: Validation of microarray expression from literature

Symbol	Annotation	Array FC		BW720c	Previous Report
MMP9	Matrix Metalloproteinase 9	192.4	▲	-	▲ Baylis et al (1995)
GMCSF/CSF2	Granulocyte-macrophage colony stimulating factor	3.5	▲	▼	▲ Baumgartner et al (2000)
IL2RA	Interleukin 2 receptor, alpha	25.9	▲	▲	▲ Dobbelaere et al (1990)
ILR2B	Interleukin 2 receptor, beta	80.3	▲	-	▲ Dobbelaere et al (1990)
ICAM1	Intercellular adhesion molecule 1	18.0	▲	▼	▲ Jensen et al (2008)
ITGA4	Integrin, alpha 4 (antigen CD49D)	5.3	▲	-	▲ Lüder et al (2009)
FYN, LYC, LYN, HCK	Src family kinase	2.5	▲	-	▲ Baumgartner et al (2003)
