# Supplementary material for: A Bovine Lymphosarcoma Cell Line Infected with Theileria annulata Exhibits an Irreversible Reconfiguration of Host Cell Gene Expression
Source: PLoS One. 2013 Jun 26;8(6):e66833. doi: 10.1371/journal.pone.0066833 (PMC3694138; doi:10.1371/journal.pone.0066833)
Supplement: Table S3 — (PDF) [file pone.0066833.s005.pdf]

Table S3A: Top ranking genes in TBL20 vs BL20 (Up in TBL)

SEQ_ID	Entrez gene ID	Symbol	Entrez Gene Name	Location	Type	Predicted BL20 expression level	FC BLvTBL	FDR BLvsTBL	FC TBLvTBL24h	FDR TBLvTBL24h	FC TBLvTBL48h	FDR TBLvTBL48h
gi_31342239_ref_NM_175716.2_	319096	XCL2	chemokine (C motif) ligand 2	Extracellular Space	cytokine	L	741.977	0.000	-18.786	0.000	-12.467	0.000
gi_31342133_ref_NM_174389.2_	281914	MMP13	matrix metallopeptidase 13 (collagenase 3)	Extracellular Space	peptidase	N	172.114	0.000	-3.980	0.000	-3.846	0.000
gi_31342766_ref_NM_174137.2_	281375	SERPINE1	serpin peptidase inhibitor, clade E (nexin, plasminogen	Extracellular Space	other	L	83.904	0.000	-3.800	0.000	-3.681	0.000
gi_124249267_ref_NM_001080904.1_	509513					L	67.927	0.000	-2.162	0.000	-4.605	0.000
gi_119895008_ref_XM_594772.3_	516616					L	62.708	0.000	-2.378	0.000	-8.959	0.000
gi_76655242_ref_XM_582736.2_	538791	KCNK18	potassium channel, subfamily K, member 18	Plasma Membrane	ion channel	N	59.961	0.000	-2.067	0.000	-3.715	0.000
gi_31341539_ref_NM_174610.2_	282366	SLC6A6	solute carrier family 6 (neurotransmitter transporter, tau	Plasma Membrane	transporter	M	45.455	0.000	-2.500	0.000	-2.570	0.000
gi_119919278_ref_XM_609882.3_	531389	FOSL1	FOS-like antigen 1	Nucleus	transcription regulator	L	37.291	0.000	-3.872	0.000	-5.028	0.000
gi_114051104_ref_NM_001046018.1_	508916	CES1 (includes E	carboxylesterase 1 (monocyte/macrophage serine este	Cytoplasm	enzyme	N	33.640	0.000	4.991	0.000	6.642	0.000
gi_115495618_ref_NM_001075554.1_	512098	CES1 (includes E	carboxylesterase 1 (monocyte/macrophage serine este	Cytoplasm	enzyme	L	32.868	0.000	4.526	0.000	5.086	0.000
gi_27901800_ref_NM_174182.1_	281485	SELL	selectin L	Plasma Membrane	other	L	32.529	0.000	9.430	0.000	25.516	0.000
gi_119917127_ref_XM_870373.2_	618042	VGF	VGF nerve growth factor inducible	Extracellular Space	growth factor	M	31.809	0.000	-12.728	0.000	-6.743	0.000
gi_119911540_ref_XM_001253061.1_	786204	CCL1	chemokine (C-C motif) ligand 1	Extracellular Space	cytokine	M	30.930	0.000	-15.202	0.000	-15.172	0.000
gi_77736504_ref_NM_001034779.1_	615215	MAPKAPK3	mitogen-activated protein kinase-activated protein kina	Nucleus	kinase	M	30.830	0.000	-2.712	0.000	-3.102	0.000
gi_115497085_ref_NM_001075287.1_	506939	CTTN	cortactin	Plasma Membrane	other	M	26.944	0.000	-2.238	0.000	-2.406	0.000
gi_119931896_ref_XM_001256737.1_	790198					N	26.458	0.000	-3.116	0.000	-6.551	0.000
gi_119893697_ref_XM_613831.3_	534164	SLIT2	slit homolog 2 (Drosophila)	Extracellular Space	other	L	15.190	0.000	-2.336	0.000	-6.673	0.000
gi_149642934_ref_NM_001099064.1_	534625	CUEDC1	CUE domain containing 1	unknown	other	L	13.006	0.000	-2.391	0.000	-2.237	0.000
gi_115497701_ref_NM_001075592.1_	512999	MANSC1	MANSC domain containing 1	unknown	other	L	12.016	0.000	-2.025	0.000	-3.422	0.000
gi_119903194_ref_XM_001255518.1_	493716					M	11.818	0.000	-1.971	0.000	-3.038	0.000
gi_119908296_ref_XM_868619.2_	616569					N	9.925	0.000	-6.584	0.000	-5.966	0.000
gi_28461172_ref_NM_175772.1_	280781	ELN	elastin	Extracellular Space	other	M	9.008	0.000	-3.254	0.000	-11.986	0.000
gi_156523227_ref_NM_001102558.1_	100124525	CX3CR1	chemokine (C-X3-C motif) receptor 1	Plasma Membrane	G-protein coupled receptor	N	8.178	0.000	55.305	0.000	137.187	0.000
gi_114052748_ref_NM_001046551.1_	615107	CXCL10	chemokine (C-X-C motif) ligand 10	Extracellular Space	cytokine	L	7.821	0.000	-7.216	0.000	-8.178	0.000
gi_119887104_ref_XM_587930.3_	510745	ABCG1	ATP-binding cassette, sub-family G (WHITE), member	Plasma Membrane	transporter	M	7.545	0.000	-6.325	0.000	-4.659	0.000
gi_31341556_ref_NM_174602.2_	282356	SLC2A1	solute carrier family 2 (facilitated glucose transporter),	Plasma Membrane	transporter	H	7.347	0.000	-2.188	0.000	-2.633	0.000
gi_119908655_ref_XM_585954.3_	509065					N	6.971	0.000	-2.026	0.000	-2.245	0.000
gi_115497115_ref_NM_001076483.1_	617915	ANGPT4	angiopoietin 4	Extracellular Space	growth factor	H	6.317	0.000	-1.951	0.000	-2.488	0.000
gi_149944706_ref_NM_001099010.1_	519269	RRAS2	related RAS viral (r-ras) oncogene homolog 2	Plasma Membrane	enzyme	N	6.198	0.000	3.471	0.000	6.574	0.000
gi_119891585_ref_XM_581625.2_	505349					M	6.186	0.000	-4.110	0.000	-7.311	0.000
gi_89886438_ref_NM_001039726.1_	281422	PRLR	prolactin receptor	Plasma Membrane	transmembrane receptor	N	6.067	0.000	-2.220	0.000	-4.282	0.000
gi_114052065_ref_NM_001045941.1_	506415	RSAD2	radical S-adenosyl methionine domain containing 2	unknown	enzyme	L	5.993	0.000	10.435	0.000	23.103	0.000
gi_115495012_ref_NM_001075819.1_	521378	CITED2	Cbp/p300-interacting transactivator, with Glu/Asp-rich c	Nucleus	transcription regulator	H	5.909	0.000	-2.431	0.000	-2.881	0.000
gi_31340804_ref_NM_174733.2_	282851	DHRS9	dehydrogenase/reductase (SDR family) member 9	Cytoplasm	enzyme	L	5.841	0.000	5.947	0.000	7.464	0.000
gi_148234191_ref_NM_001098036.1_	515437	SLC39A14	solute carrier family 39 (zinc transporter), member 14	Plasma Membrane	transporter	H	5.231	0.000	-2.133	0.000	-2.895	0.000
gi_119901455_ref_XM_614574.3_	541106	RRAGD	Ras-related GTP binding D	Cytoplasm	enzyme	L	5.197	0.000	5.305	0.000	6.589	0.000
gi_119908653_ref_XM_583371.3_	538868	RGSL1	regulator of G-protein signaling like 1	unknown	other	N	4.792	0.000	-4.166	0.000	-4.469	0.000
gi_119907150_ref_XM_612483.3_	533166	SORL1	sortilin-related receptor, L(DLR class) A repeats-contair	Plasma Membrane	transporter	L	4.761	0.000	5.674	0.000	8.918	0.000
gi_119912220_ref_XM_869703.2_	535629	SNIP	SNAP25-interacting protein	Cytoplasm	other	M	4.552	0.000	-4.863	0.000	-5.383	0.000
gi_119888721_ref_XM_612644.3_	540687	GPR3	G protein-coupled receptor 3	Plasma Membrane	G-protein coupled receptor	L	4.415	0.000	-3.230	0.000	-5.521	0.000

Table S3B: Top ranking genes in TBL20 vs BL20 (Down in TBL)

SEQ_ID	Entrez gene ID	Symbol	Entrez Gene Name	Location	Type	Predicted BL20 expression level	FC BLvTBL	FDR BLvsTBL	FC TBLvTBL24h	FDR TBLvTBL24h	FC TBLvTBL48h	FDR TBLvTBL48h
gi_119917736_ref_XM_609898.3_	531405	SORCS3	sortilin-related VPS10 domain containing receptor 3	Nucleus	transporter	VH	-530.654	0.000	1.102	1.075	1.427	0.968
gi_119930329_ref_XM_001253356.1_	785289					VH	-388.537	0.000	1.872	0.072	3.895	0.000
gi_119908049_ref_XM_611589.3_	515828					VH	-317.451	0.000	1.037	0.714	1.101	0.743
gi_119925613_ref_XM_583887.3_	507299					VH	-301.600	0.000	1.103	1.097	1.007	0.960
gi_119915839_ref_XM_588122.3_	617881	F13A1	coagulation factor XIII, A1 polypeptide	Extracellular Space	enzyme	VH	-289.916	0.000	-1.225	0.365	-1.193	0.452
gi_118151215_ref_NM_001078068.1_	617148	PPP1R14C	protein phosphatase 1, regulatory (inhibitor) subunit 14	Cytoplasm	other	VH	-275.447	0.000	1.366	0.943	1.490	0.786
gi_119902260_ref_XM_603355.3_	404176					VH	-238.931	0.000	2.254	0.017	3.088	0.005
gi_31341436_ref_NM_174652.2_	282470	SLC11A1	solute carrier family 11 (proton-coupled divalent metal i	Plasma Membrane	transporter	VH	-238.083	0.000	2.273	0.015	8.234	0.000
gi_119911249_ref_XM_867060.2_	615295	LAIR1	leukocyte-associated immunoglobulin-like receptor 1	Plasma Membrane	transmembrane receptor	VH	-230.065	0.000	1.227	1.175	1.082	1.141
gi_134085858_ref_NM_001083409.1_	511774	C1QTNF1	C1q and tumor necrosis factor related protein 1	Extracellular Space	other	VH	-207.726	0.000	1.511	0.504	3.418	0.002
gi_119887727_ref_XM_598566.3_	520327	KYNU	kynureninase (L-kynurenine hydrolase)	Cytoplasm	enzyme	H	-197.621	0.000	1.311	1.174	2.482	0.024
gi_119891326_ref_XM_590508.3_	512903	CPA4	carboxypeptidase A4	Extracellular Space	peptidase	VH	-195.388	0.000	3.829	0.000	6.574	0.000
gi_119919107_ref_XM_591760.3_	513984	SLC15A3	solute carrier family 15, member 3	unknown	transporter	VH	-188.144	0.000	5.286	0.000	12.409	0.000
gi_31343049_ref_NM_174010.2_	281052	CD36	CD36 molecule (thrombospondin receptor)	Plasma Membrane	transmembrane receptor	VH	-185.369	0.000	2.016	0.051	2.040	0.123
gi_119910462_ref_XM_592455.3_	514582					VH	-169.428	0.000	2.149	0.027	4.403	0.000
gi_119920009_ref_XM_601830.3_	523530	NXF3	nuclear RNA export factor 3	Nucleus	transporter	VH	-166.634	0.000	1.449	0.683	1.393	0.942
gi_153792426_ref_NM_001099726.1_	782045	CYSLTR1	cysteinyl leukotriene receptor 1	Plasma Membrane	G-protein coupled receptor	VH	-162.124	0.000	1.562	0.439	12.481	0.000
gi_119916419_ref_XM_587341.3_	510218	ALPK2	alpha-kinase 2	unknown	kinase	VH	-154.916	0.000	-1.167	0.376	-1.238	0.252
gi_119918049_ref_XM_001256292.1_	789579					VH	-149.900	0.000	2.161	0.029	6.379	0.000
gi_119906950_ref_XM_001251159.1_	783497	PPP1R14C	protein phosphatase 1, regulatory (inhibitor) subunit 14	Cytoplasm	other	VH	-132.800	0.000	1.047	1.015	1.341	1.047

SEQ_ID	Entrez gene ID	Symbol	Entrez Gene Name	Location	Type	Predicted BL20 expression level	FC BLvTBL	FDR BLvsTBL	FC TBLvTBL24h	FDR TBLvTBL24h	FC TBLvTBL48h	FDR TBLvTBL48h
gi_119920237_ref_XM_001253574.1_	785599	ZCCHC13	zinc finger, CCHC domain containing 13	unknown	other	H	-118.534	0.000	2.387	0.010	15.366	0.000
gi_119934529_ref_XM_001255814.1_	788901					VH	-112.738	0.000	1.909	0.094	4.136	0.000
gi_119920839_ref_XM_001250568.1_	782007	ZCCHC13	zinc finger, CCHC domain containing 13	unknown	other	H	-112.219	0.000	2.187	0.026	14.107	0.000
gi_76660614_ref_XM_597941.2_	519716	CCR6	chemokine (C-C motif) receptor 6	Plasma Membrane	G-protein coupled receptor	H	-110.621	0.000	1.437	0.755	2.660	0.014
gi_115495094_ref_NM_001075968.1_	532587	BLK	B lymphoid tyrosine kinase	Cytoplasm	kinase	VH	-110.233	0.000	3.063	0.000	4.537	0.000
gi_157954060_ref_NM_001109795.1_	513856	A2M	alpha-2-macroglobulin	Extracellular Space	transporter	VH	-109.925	0.000	2.001	0.059	4.697	0.000
gi_118151371_ref_NM_001078149.1_	777644	GSTA3	glutathione S-transferase alpha 3	Cytoplasm	enzyme	H	-108.114	0.000	-1.036	0.534	-1.189	0.349
gi_119913552_ref_XM_606794.3_	281848	IGF1R	insulin-like growth factor 1 receptor	Plasma Membrane	transmembrane receptor	VH	-107.774	0.000	1.943	0.061	1.866	0.190
gi_119923075_ref_XM_001254039.1_	786327					VH	-100.332	0.000	3.046	0.000	9.232	0.000
gi_156120512_ref_NM_001101932.1_	510702					VH	-99.953	0.000	-1.072	0.723	-1.180	0.420
gi_149642838_ref_NM_001099200.1_	786888	PNPLA1	patatin-like phospholipase domain containing 1	unknown	enzyme	VH	-99.595	0.000	1.718	0.243	1.765	0.340
gi_157427947_ref_NM_001105411.1_	534801	GFRA1	GDNF family receptor alpha 1	Plasma Membrane	transmembrane receptor	VH	-99.175	0.000	1.884	0.106	4.459	0.000
gi_134085612_ref_NM_001083388.1_	508076	COL18A1	collagen, type XVIII, alpha 1	Extracellular Space	other	VH	-98.389	0.000	1.260	1.255	1.266	1.312
gi_126723156_ref_NM_001082469.1_	782387	RAG2	recombination activating gene 2	Nucleus	enzyme	H	-97.720	0.000	1.884	0.117	1.188	1.332
gi_119918193_ref_XM_598235.3_	520004	UNC5D	unc-5 homolog D (C. elegans)	unknown	other	H	-86.221	0.000	1.083	1.131	1.357	1.151
gi_157279834_ref_NM_001104962.1_	353108					VH	-85.808	0.000	7.308	0.000	7.674	0.000
gi_32189337_ref_NM_174143.1_	281401	PIGR	polymeric immunoglobulin receptor	Plasma Membrane	transporter	H	-85.226	0.000	2.400	0.010	3.736	0.001
gi_99028960_ref_NM_174184.3_	281489	SLAMF1	signaling lymphocytic activation molecule family memb	Plasma Membrane	transmembrane receptor	VH	-84.352	0.000	1.301	1.106	1.326	1.182
