# Supplementary material for: A Bovine Lymphosarcoma Cell Line Infected with Theileria annulata Exhibits an Irreversible Reconfiguration of Host Cell Gene Expression
Source: PLoS One. 2013 Jun 26;8(6):e66833. doi: 10.1371/journal.pone.0066833 (PMC3694138; doi:10.1371/journal.pone.0066833)
Supplement: Table S4 — (PDF) [file pone.0066833.s006.pdf]

Table S4A: No evidence of expression in BL20; up-regulated in TBL20

SEQ_ID	Entrez gene ID	Symbol	Entrez Gene Name	Location	Type	Predicted BL20 expression level	FC BLvTBL	FDR BLvsTBL	FC TBLvTBL24h	FDR TBLvTBL24h	FC TBLvTBL48h	FDR TBLvTBL48h
gi_119903692_ref_XM_871827.2_	521137	LRRTM4	leucine rich repeat transmembrane neuronal 4	unknown	other	N	314.277	0.000	2.084	0.041	1.519	0.778
gi_158519830_ref_NM_001110080.1_	532603	KIAA1598	KIAA1598	unknown	other	N	234.222	0.000	-1.917	0.000	-5.081	0.000
gi_76608886_ref_XM_868724.1_	616646	CLEC4D	C-type lectin domain family 4, member D	unknown	other	N	114.747	0.000	2.872	0.001	2.600	0.016
gi_76655242_ref_XM_582736.2_	538791	KCNK18	potassium channel, subfamily K, member 18	Plasma Membrane	ion channel	N	59.961	0.000	-2.067	0.000	-3.715	0.000
gi_119918977_ref_XM_600048.3_	521780	VWA5A	von Willebrand factor A domain containing 5A	unknown	other	N	58.984	0.000	1.731	0.236	1.417	1.014
gi_30794303_ref_NM_181004.1_	281050	CD28	CD28 molecule	Plasma Membrane	other	N	52.163	0.000	1.563	0.526	2.284	0.051
gi_119923735_ref_XM_586767.3_	539380	MUM1L1	melanoma associated antigen (mutated) 1-like 1	unknown	other	N	47.604	0.000	1.930	0.091	1.832	0.276
gi_115432056_ref_NM_001037100.2_	282151	BCL2A1	BCL2-related protein A1	Cytoplasm	other	N	43.582	0.000	1.458	0.802	1.788	0.330
gi_114051635_ref_NM_001046438.1_	538730	NRN1	neuritin 1	Cytoplasm	other	N	36.912	0.000	2.077	0.046	1.452	0.948
gi_154152112_ref_NM_001100345.1_	511799	LEPREL1	leprecan-like 1	unknown	enzyme	N	32.519	0.000	1.744	0.239	1.353	1.142
gi_167583539_ref_NM_001114506.1_	504507	TNFSF13B	tumor necrosis factor (ligand) superfamily, member 13t	Extracellular Space	cytokine	N	20.670	0.000	2.146	0.031	1.326	1.211
gi_115496847_ref_NM_001076375.1_	615436	AFAP1L2	actin filament associated protein 1-like 2	Cytoplasm	other	N	12.052	0.000	2.018	0.060	3.672	0.001
gi_119893058_ref_XM_868762.2_	616674	CLEC4A	C-type lectin domain family 4, member A	Plasma Membrane	transmembrane receptor	N	10.246	0.000	3.657	0.000	1.866	0.251
gi_119908296_ref_XM_868619.2_	616569					N	9.925	0.000	-6.584	0.000	-5.966	0.000
gi_31341597_ref_NM_174588.2_	282329	PTGER2	prostaglandin E receptor 2 (subtype EP2), 53kDa	Plasma Membrane	G-protein coupled receptor	N	6.462	0.000	-1.461	0.050	-3.223	0.000
gi_149944706_ref_NM_001099010.1_	519269	RRAS2	related RAS viral (r-ras) oncogene homolog 2	Plasma Membrane	enzyme	N	6.198	0.000	3.471	0.000	6.574	0.000
gi_89886438_ref_NM_001039726.1_	281422	PRLR	prolactin receptor	Plasma Membrane	transmembrane receptor	N	6.067	0.000	-2.220	0.000	-4.282	0.000
gi_31341440_ref_NM_173879.2_	280687	F5	coagulation factor V (proaccelerin, labile factor)	Plasma Membrane	other	N	5.666	0.000	1.151	1.195	1.782	0.312
gi_122692332_ref_NM_001080372.1_	788454	GM41	predicted gene 41	unknown	other	N	4.332	0.000	-1.623	0.005	-3.178	0.000
gi_119903179_ref_XM_871776.2_	520709	IL1RL1	interleukin 1 receptor-like 1	Plasma Membrane	transmembrane receptor	N	4.195	0.000	-2.315	0.000	-3.775	0.000
gi_115495866_ref_NM_001076246.1_	540853	SNAP25	synaptosomal-associated protein, 25kDa	Plasma Membrane	transporter	N	3.536	0.000	-1.141	0.412	-1.069	0.776
gi_115497747_ref_NM_001075596.1_	513055					N	3.403	0.000	4.314	0.000	4.768	0.000
gi_78050044_ref_NM_001035072.1_	509578	CD96	CD96 molecule	Plasma Membrane	other	N	2.976	0.000	-2.167	0.000	-1.993	0.001
gi_139948963_ref_NM_001083752.1_	540702	C3AR1	complement component 3a receptor 1	Plasma Membrane	G-protein coupled receptor	N	2.761	0.000	1.144	1.214	1.811	0.291
gi_115496797_ref_NM_001075859.1_	524513	SERPINB9	serpin peptidase inhibitor, clade B (ovalbumin), membe	Cytoplasm	other	N	2.489	0.000	-1.450	0.024	-1.578	0.015
gi_119893892_ref_XM_612028.3_	280832	KIT	v-kit Hardy-Zuckerman 4 feline sarcoma viral oncogene	Plasma Membrane	kinase	N	2.351	0.000	1.445	0.720	6.956	0.000
gi_156120552_ref_NM_001101952.1_	512227	ACSM2A	acyl-CoA synthetase medium-chain family member 2A	unknown	other	N	2.298	0.000	1.117	1.008	-1.275	0.197
gi_70778751_ref_NM_001025344.1_	538951	NANOG	Nanog homeobox	Nucleus	transcription regulator	N	2.261	0.000	-2.393	0.000	-2.434	0.000
gi_125991883_ref_NM_001081579.1_	504991	ANKRD22	ankyrin repeat domain 22	Nucleus	transcription regulator	N	2.176	0.000	1.233	1.249	-1.005	0.797
gi_119908651_ref_XM_866033.2_	614500					N	2.020	0.001	-1.807	0.002	-1.774	0.004
gi_115497133_ref_NM_001075864.1_	525072	PLA2G4A	phospholipase A2, group IVA (cytosolic, calcium-depen	Cytoplasm	enzyme	N	1.941	0.001	1.505	0.559	-1.488	0.041
gi_115497379_ref_NM_001075626.1_	513537	ALDH8A1	aldehyde dehydrogenase 8 family, member A1	unknown	enzyme	N	1.716	0.004	2.607	0.004	2.040	0.128
gi_119887028_ref_XM_592594.3_	514701	FAM3B	family with sequence similarity 3, member B	Extracellular Space	cytokine	N	1.684	0.005	1.487	0.585	1.344	1.116
gi_147898888_ref_NM_001098163.1_	100036590	KLRC1	killer cell lectin-like receptor subfamily C, member 1	Plasma Membrane	transmembrane receptor	N	1.654	0.007	-1.067	0.369	-1.611	0.011
gi_119919810_ref_XM_001252953.1_	784732					N	1.497	0.014	-1.652	0.008	-1.786	0.004
gi_77735504_ref_NM_001034275.1_	507026	ICOS	inducible T-cell co-stimulator	Plasma Membrane	other	N	1.490	0.016	-1.175	0.271	-1.562	0.021
gi_119922384_ref_XM_001254389.1_	786820	GM41	predicted gene 41	unknown	other	N	1.480	0.021	-1.227	0.324	-1.737	0.006
gi_156121332_ref_NM_001102345.1_	787444	NKAIN2	Na+/K+ transporting ATPase interacting 2	unknown	other	N	1.441	0.024	-1.037	0.929	1.015	1.090
gi_157785610_ref_NM_001105636.1_	751790	UGT1A1	UDP glucuronosyltransferase 1 family, polypeptide A1	Cytoplasm	enzyme	N	1.424	0.029	7.186	0.000	3.049	0.005
gi_115495972_ref_NM_001075520.1_	511498	CYP2C44	cytochrome P450, family 2, subfamily c, polypeptide 44	Cytoplasm	enzyme	N	1.420	0.030	-1.508	0.020	-1.828	0.003
gi_119923335_ref_XM_592662.3_	514757					N	1.420	0.037	-1.418	0.074	-1.567	0.017
gi_119909794_ref_XM_596586.3_	518395	ADAMTS18	ADAM metallopeptidase with thrombospondin type 1 m	Extracellular Space	peptidase	N	1.415	0.029	2.556	0.005	2.227	0.061
gi_119922231_ref_XM_001254266.1_	786636					N	1.414	0.027	1.157	1.129	-1.388	0.103
gi_119907873_ref_XM_599338.3_	521081	4833423E24RIK	RIKEN cDNA 4833423E24 gene	unknown	other	N	1.380	0.036	-1.759	0.004	-1.469	0.056
gi_31342852_ref_NM_174097.2_	281270	HOPX	HOP homeobox	Nucleus	transcription regulator	N	1.356	0.043	-1.138	0.443	-1.611	0.011
gi_31341980_ref_NM_174445.2_	282023	PTGS2	prostaglandin-endoperoxide synthase 2 (prostaglandin	Cytoplasm	enzyme	N	1.317	0.055	-1.130	0.502	1.050	1.007

Table S4B: No change between BL20 and TBL20

SEQ_ID	Entrez gene ID	Symbol	Entrez Gene Name	Location	Type	Predicted BL20 expression level	FC BLvTBL	FDR BLvsTBL	FC TBLvTBL24h	FDR TBLvTBL24h	FC TBLvTBL48h	FDR TBLvTBL48h
gi_119903302_ref_XM_588244.3_	511001	CLEC4F	C-type lectin domain family 4, member F	Plasma Membrane	other	L	-1.609	1.15	5.864	0.000	56.038	0.000
gi_66792901_ref_NM_001024556.1_	526704	VNN1	vanin 1	Plasma Membrane	enzyme	N	-1.351	1.321	2.957	0.000	24.904	0.000
gi_156120930_ref_NM_001102142.1_	531995	SP7	Sp7 transcription factor	Nucleus	transcription regulator	L	-1.480	1.324	5.955	0.000	17.168	0.000
gi_119914328_ref_XM_589824.3_	615277					N	-1.152	1.129	5.949	0.000	11.605	0.000
gi_114051859_ref_NM_001045963.1_	507285	CHN2	chimerin (chimaerin) 2	Cytoplasm	other	L	-1.577	1.259	5.176	0.000	8.349	0.000
gi_115495332_ref_NM_001075143.1_	404173					H	-1.616	1.17	1.175	1.254	8.065	0.000
gi_157785549_ref_NM_174320.3_	281773	FXYD2	FXYD domain containing ion transport regulator 2	Plasma Membrane	ion channel	H	-1.302	1.364	3.962	0.000	6.688	0.000
gi_119902602_ref_XM_611694.3_	532574	MYO5C	myosin VC	unknown	other	L	-1.389	1.389	7.973	0.000	6.255	0.000
gi_119910346_ref_XM_001253667.1_	785741	ZBTB32	zinc finger and BTB domain containing 32	Nucleus	transcription regulator	H	-1.416	1.359	4.011	0.000	6.205	0.000
gi_119922570_ref_XM_001254638.1_	787162	ARMCX6	armadillo repeat containing, X-linked 6	unknown	other	H	-1.697	1.053	4.351	0.000	6.084	0.000

gi_119906549_ref_XM_877427.2_	511188	EYA1	eyes absent homolog 1 (Drosophila)	Nucleus	phosphatase	L	-1.265	1.248	3.740	0.000	5.918	0.000
gi_156120694_ref_NM_001102024.1_	515418	CD1A	CD1a molecule	Plasma Membrane	other	N	-1.168	1.109	4.224	0.000	5.756	0.000
gi_119914342_ref_XM_871103.2_	509670	XIRP1	xin actin-binding repeat containing 1	Plasma Membrane	other	L	-1.551	1.217	3.174	0.000	5.696	0.000
gi_119922350_ref_XM_583562.3_	507019	ADAM28	ADAM metallopeptidase domain 28	Plasma Membrane	peptidase	L	-1.218	1.237	6.711	0.000	5.223	0.000
gi_90403615_ref_NM_001039957.1_	407124					M	-1.501	1.296	2.320	0.014	5.086	0.000
gi_59676571_ref_NM_001012284.1_	497204	UBA7	ubiquitin-like modifier activating enzyme 7	Cytoplasm	enzyme	M	-1.257	1.273	1.765	0.195	4.913	0.000
gi_114051673_ref_NM_001046435.1_	538675	CALCOCO1	calcium binding and coiled-coil domain 1	Nucleus	transcription regulator	H	-1.401	1.389	2.751	0.002	4.896	0.000
gi_66792763_ref_NM_001024486.1_	506687	SLC37A2	solute carrier family 37 (glycerol-3-phosphate transporter), m	unknown	transporter	H	-1.227	1.289	3.751	0.000	4.751	0.000
gi_31342589_ref_NM_174209.2_	281559	UACA	uveal autoantigen with coiled-coil domains and ankyrin repea	Cytoplasm	other	L	-1.206	1.167	5.477	0.000	4.568	0.000
gi_76659999_ref_XM_612871.2_	540732	ACER2	alkaline ceramidase 2	unknown	other	H	-1.551	1.238	2.997	0.000	4.485	0.000
gi_76611576_ref_XM_587002.2_	407136	HTR1D	5-hydroxytryptamine (serotonin) receptor 1D	Plasma Membrane	G-protein coupled receptor	L	-1.473	1.262	1.955	0.063	4.474	0.000
gi_119901918_ref_XM_864907.2_	511279	SMAD6	SMAD family member 6	Nucleus	transcription regulator	L	-1.659	1.01	2.819	0.001	4.357	0.000
gi_116003876_ref_NM_001076828.1_	510532	PARP9	poly (ADP-ribose) polymerase family, member 9	Nucleus	other	H	-1.266	1.335	3.643	0.000	4.327	0.000
gi_115495538_ref_NM_001075430.1_	510102	RARRES1	retinoic acid receptor responder (tazarotene induced) 1	Plasma Membrane	other	VH	-1.370	1.387	2.673	0.003	4.248	0.000
gi_115495706_ref_NM_001075507.1_	511291	CHPT1	choline phosphotransferase 1	unknown	enzyme	N	-1.220	1.202	4.984	0.000	4.209	0.000
gi_119911597_ref_XM_587287.3_	510175	PROCA1	protein interacting with cyclin A1	unknown	other	H	-1.662	1.146	2.406	0.010	4.155	0.000
gi_119917459_ref_XM_580537.3_	538469	SLC16A12	solute carrier family 16, member 12 (monocarboxylic acid tra	unknown	other	H	-1.639	1.169	2.901	0.000	4.122	0.000
gi_157074195_ref_NM_001103342.1_	100125309					H	-1.263	1.242	2.489	0.006	4.079	0.000
gi_134085668_ref_NM_001083451.1_	527470	RORC	RAR-related orphan receptor C	Nucleus	ligand-dependent nuclear rece	L	-1.236	1.132	2.306	0.014	4.023	0.000
gi_149642800_ref_NM_001098963.1_	513785	GABBR1	gamma-aminobutyric acid (GABA) B receptor, 1	Plasma Membrane	G-protein coupled receptor	H	-1.413	1.352	1.839	0.136	4.023	0.000
gi_156120784_ref_NM_001102069.1_	522763					H	-1.297	1.337	4.017	0.000	4.005	0.000
gi_115495928_ref_NM_001076243.1_	540828	PHYHD1	phytanoyl-CoA dioxygenase domain containing 1	unknown	other	H	-1.574	1.272	2.277	0.017	3.972	0.000
gi_119913871_ref_XM_617995.3_	537810	AKAP6	A kinase (PRKA) anchor protein 6	Nucleus	other	L	-1.519	1.305	2.691	0.003	3.909	0.001
gi_139949223_ref_NM_001083720.1_	533523	DDR2	discoidin domain receptor tyrosine kinase 2	Plasma Membrane	kinase	M	-1.597	1.222	2.884	0.001	3.846	0.001
gi_119913160_ref_XM_001251499.1_	511595	PARP8	poly (ADP-ribose) polymerase family, member 8	unknown	other	VH	-1.305	1.329	2.533	0.005	3.833	0.001
gi_119914298_ref_XR_028825.1_	509859					H	-1.539	1.245	2.022	0.050	3.815	0.001
gi_115495998_ref_NM_001076232.1_	540667	39508	membrane-associated ring finger (C3HC4) 8	Cytoplasm	other	H	-1.370	1.381	2.369	0.011	3.523	0.001
gi_116003900_ref_NM_001076841.1_	512672	H2-T24	histocompatibility 2, T region locus 24	unknown	other	H	-1.299	1.306	2.863	0.001	3.422	0.001
gi_119914325_ref_XM_616073.3_	535956	SCN11A	sodium channel, voltage-gated, type XI, alpha subunit	Plasma Membrane	ion channel	L	-1.325	1.111	2.338	0.012	3.418	0.002
gi_78365258_ref_NM_001035400.1_	533618	ARSK	arylsulfatase family, member K	Extracellular Space	enzyme	H	-1.571	1.262	3.160	0.000	3.336	0.002
gi_114052345_ref_NM_001046348.1_	533321	2310002L09RIK	RIKEN cDNA 2310002L09 gene	unknown	other	M	-1.318	1.108	2.685	0.002	3.306	0.003
gi_119906688_ref_XM_601083.3_	522795	KLF10	Kruppel-like factor 10	Nucleus	transcription regulator	H	-1.750	1.019	2.065	0.047	3.260	0.003
gi_119890208_ref_XM_588670.3_	511350	CC2D1B	coiled-coil and C2 domain containing 1B	unknown	other	H	-1.178	1.174	1.651	0.357	3.260	0.003
gi_149642646_ref_NM_001099168.1_	617625	RBM43	RNA binding motif protein 43	unknown	other	H	-1.653	1.181	2.502	0.006	3.201	0.003
gi_77735692_ref_NM_001034368.1_	509896	ABHD4	abhydrolase domain containing 4	unknown	peptidase	H	-1.755	1.009	2.257	0.019	3.201	0.003
gi_77735580_ref_NM_001034314.1_	508132	NAGK	N-acetylglucosamine kinase	Cytoplasm	kinase	H	-1.428	1.369	2.125	0.032	3.178	0.003
gi_78370160_ref_NM_001035483.1_	616801	TRAF3IP2	TRAF3 interacting protein 2	unknown	other	H	-1.376	1.39	2.271	0.017	3.157	0.003
gi_119912581_ref_XR_027582.1_	782067	PITPNC1	phosphatidylinositol transfer protein, cytoplasmic 1	Cytoplasm	transporter	H	-1.569	1.272	1.724	0.269	3.153	0.003
gi_119905187_ref_XM_606371.3_	527964	PARD3	par-3 partitioning defective 3 homolog (C. elegans)	Plasma Membrane	other	L	-1.442	1.359	2.480	0.006	3.092	0.004
gi_119911873_ref_XM_583737.3_	507171	CYB5D1	cytochrome b5 domain containing 1	unknown	other	H	-1.647	1.099	2.224	0.020	3.084	0.004
gi_119880356_ref_XM_866793.2_	533510	KLHL24	kelch-like 24 (Drosophila)	unknown	other	VH	-1.509	1.335	2.096	0.042	3.081	0.004
gi_78365245_ref_NM_001035418.1_	537718	ARHGEF9	Cdc42 guanine nucleotide exchange factor (GEF) 9	Cytoplasm	other	H	-1.330	1.327	2.351	0.011	3.077	0.004
gi_119889996_ref_XR_028184.1_	508347	IFI44L	interferon-induced protein 44-like	unknown	other	H	-1.121	1.08	2.321	0.014	3.031	0.005
gi_119891466_ref_XM_869387.2_	617183					L	-1.262	1.237	3.486	0.000	3.024	0.005
gi_119910902_ref_XR_028394.1_	618463	SIGLEC12	sialic acid binding Ig-like lectin 12	Plasma Membrane	other	L	-1.513	1.309	1.393	0.903	3.024	0.005
gi_119930198_ref_XM_001251085.1_	786617	ZNF280B	zinc finger protein 280B	Nucleus	other	M	-1.396	1.381	-1.477	0.017	-3.007	0.000
gi_84000064_ref_NM_001038041.1_	505855					H	-1.653	1.155	-1.642	0.009	-3.291	0.000
gi_115497007_ref_NM_001075292.1_	506986	SEC61B	Sec61 beta subunit	Cytoplasm	transporter	M	-1.189	1.157	-2.223	0.000	-4.028	0.000
gi_156120382_ref_NM_001101867.1_	506282	EMID1	EMI domain containing 1	Extracellular Space	other	H	-1.347	1.371	-4.284	0.000	-4.141	0.000
gi_119889055_ref_XM_584927.3_	508183	SLAMF6	SLAM family member 6	Plasma Membrane	transmembrane receptor	H	-1.475	1.354	3.977	0.000	2.417	0.031
gi_139948604_ref_NM_001083695.1_	516522	FRRS1	ferric-chelate reductase 1	Plasma Membrane	transmembrane receptor	M	-1.558	1.214	3.444	0.000	2.845	0.008
gi_119902080_ref_XM_588656.3_	407204					L	-1.227	1.071	3.378	0.000	2.085	0.107
gi_122692458_ref_NM_001080307.1_	538604	ABI3BP	ABI family, member 3 (NESH) binding protein	unknown	other	L	-1.502	1.211	3.374	0.000	2.639	0.014
gi_150247098_ref_NM_001099388.1_	539898	ZBED5	zinc finger, BED-type containing 5	Nucleus	other	H	-1.737	1.059	3.170	0.000	2.901	0.006
gi_119916198_ref_XM_868601.2_	616555	OSBPL1A	oxysterol binding protein-like 1A	Cytoplasm	other	H	-1.214	1.261	3.163	0.000	2.815	0.008
gi_84370144_ref_NM_001038558.1_	529873	CADM1	cell adhesion molecule 1	Plasma Membrane	other	L	-1.715	1.026	3.099	0.000	2.451	0.026
gi_119905251_ref_XM_864298.2_	613475	MSRB2	methionine sulfoxide reductase B2	Nucleus	transcription regulator	VH	-1.416	1.388	3.091	0.000	2.561	0.019
gi_126165263_ref_NM_001081712.1_	505820	SERPINA3	serpin peptidase inhibitor, clade A (alpha-1 antiproteinase, ar	Extracellular Space	other	L	-1.495	1.295	3.066	0.000	2.181	0.073
gi_119895022_ref_XR_028583.1_	510442					VH	-1.157	1.12	-3.052	0.000	-1.309	0.211
