# Supplementary material for: A Bovine Lymphosarcoma Cell Line Infected with Theileria annulata Exhibits an Irreversible Reconfiguration of Host Cell Gene Expression
Source: PLoS One. 2013 Jun 26;8(6):e66833. doi: 10.1371/journal.pone.0066833 (PMC3694138; doi:10.1371/journal.pone.0066833)
Supplement: Table S5 — Cell cycle associated genes. (PDF) [file pone.0066833.s007.pdf]

Table S5: Cell cycle associated genes

Symbol/Class	Entrez Gene Name	Entrez Gene ID	FC		BPQ Response	
CDKs						
PFTK1	PFTAIRE protein kinase 1	784403	7.55	▲	-	-
CDK3	cyclin-dependent kinase 3	618631	1.90	▲	-	-
Cyclins						
CCNA1	cyclin A1	521939	3.85	▼	4.38	▲
CCNE1	cyclin E1	533526	1.32	▲	1.88	▼
CCNA2	cyclin A2	281667	1.31	▲	1.61	▼
CCNYL1	cyclin Y-like 1	538167	1.38	▲	-	-
CCND2	cyclin D2	615414	-	-	1.46	▼
CCNE2	cyclin E2	538436	-	-	2.16	▼
CCNG2	cyclin G2	512960	-	-	2.57	▲
CDK Inhibitors						
CDKN2D	cyclin-dependent kinase inhibitor 2D (p19, inhibits CDK4)	510244	4.99	▼	-	
CDKN1B	cyclin-dependent kinase inhibitor 1B (p27, Kip1)	512613	3.52	▼	-	
CDKN1C	cyclin-dependent kinase inhibitor 1C (p57, Kip2)	510972	3.22	▼	-	
CDKN2C	cyclin-dependent kinase inhibitor 2C (p18, inhibits CDK4)	505691	3.12	▼	-	
CDKN2A	cyclin-dependent kinase inhibitor 2A (melanoma, p16, inhibits CDK4)	616369	2.96	▼	-	
CDK-activation						
CDC25A	cell division cycle 25 homolog A (S. pombe)	520188	1.42	▲	2.06	▼
