# Supplementary material for: A Bovine Lymphosarcoma Cell Line Infected with Theileria annulata Exhibits an Irreversible Reconfiguration of Host Cell Gene Expression
Source: PLoS One. 2013 Jun 26;8(6):e66833. doi: 10.1371/journal.pone.0066833 (PMC3694138; doi:10.1371/journal.pone.0066833)
Supplement: Table S6 — (PDF) [file pone.0066833.s008.pdf]

## Table S6A: Extracellular - cytokines and growth factors

| SEQ_ID | Entrez gene ID | Symbol | Entrez Gene Name | Location | Type | Predicted BL20 expression level | FC BLvTBL | FDR BLvsTBL | FC TBLvTBL24h | FDR TBLvTBL24h | FC TBLvTBL48h | FDR TBLvTBL48h |
|---|---|---|---|---|---|---|---|---|---|---|---|---|
| gi_31342239_ref_NM_175716.2_ | 319096 | XCL2 | chemokine (C motif) ligand 2 | Extracellular Space | cytokine | L | 741.977 | 0.000 | -18.786 | 0.000 | -12.467 | 0.000 |
| gi_119917127_ref_XM_870373.2_ | 618042 | VGF | VGF nerve growth factor inducible | Extracellular Space | growth factor | M | 31.809 | 0.000 | -12.728 | 0.000 | -6.743 | 0.000 |
| gi_119911540_ref_XM_001253061.1_ | 786204 | CCL1 | chemokine (C-C motif) ligand 1 | Extracellular Space | cytokine | M | 30.930 | 0.000 | -15.202 | 0.000 | -15.172 | 0.000 |
| gi_167583539_ref_NM_001114506.1_ | 504507 | TNFSF13B | tumor necrosis factor (ligand) superfamily, member 13 | Extracellular Space | cytokine | N | 20.670 | 0.000 | 2.146 | 0.031 | 1.326 | 1.211 |
| gi_31342229_ref_NM_174357.2_ | 281860 | IL1RN | interleukin 1 receptor antagonist | Extracellular Space | cytokine | L | 17.914 | 0.000 | -2.608 | 0.000 | -1.868 | 0.002 |
| gi_31343615_ref_NM_175827.2_ | 327712 | CCL5 | chemokine (C-C motif) ligand 5 | Extracellular Space | cytokine | M | 10.841 | 0.000 | -1.524 | 0.023 | -2.809 | 0.000 |
| gi_114052748_ref_NM_001046551.1_ | 615107 | CXCL10 | chemokine (C-X-C motif) ligand 10 | Extracellular Space | cytokine | L | 7.821 | 0.000 | -7.216 | 0.000 | -8.178 | 0.000 |
| gi_154152166_ref_NM_001100365.1_ | 514933 | EBI3 | Epstein-Barr virus induced 3 | Extracellular Space | cytokine | L | 6.837 | 0.000 | -1.108 | 0.671 | 1.813 | 0.292 |
| gi_115497115_ref_NM_001076483.1_ | 617915 | ANGPT4 | angiopoietin 4 | Extracellular Space | growth factor | H | 6.317 | 0.000 | -1.951 | 0.000 | -2.488 | 0.000 |
| gi_119884701_ref_XM_583785.3_ | 507215 | TNFSF10 | tumor necrosis factor (ligand) superfamily, member 10 | Extracellular Space | cytokine | M | 4.526 | 0.000 | 2.292 | 0.015 | 2.362 | 0.036 |
| gi_119896170_ref_XM_593584.3_ | 515549 | IFNW1 | interferon, omega 1 | Extracellular Space | cytokine | L | 3.866 | 0.000 | -2.052 | 0.000 | -3.302 | 0.000 |
| gi_31343249_ref_NM_173924.2_ | 280827 | IL7 | interleukin 7 | Extracellular Space | cytokine | L | 3.741 | 0.000 | -1.078 | 0.753 | -2.570 | 0.000 |
| gi_76668250_ref_XM_589595.2_ | 539800 | TNFSF4 | tumor necrosis factor (ligand) superfamily, member 4 | Extracellular Space | cytokine | M | 3.633 | 0.000 | -2.115 | 0.000 | -1.930 | 0.001 |
| gi_31343004_ref_NM_174027.2_ | 281095 | CSF2 | colony stimulating factor 2 (granulocyte-macrophage) | Extracellular Space | cytokine | L | 3.541 | 0.000 | -3.935 | 0.000 | -3.576 | 0.000 |
| gi_119911538_ref_XM_001253011.1_ | 786156 | CCL1 | chemokine (C-C motif) ligand 1 | Extracellular Space | cytokine | H | 3.371 | 0.000 | -6.822 | 0.000 | -9.536 | 0.000 |
| gi_149773587_ref_NM_001099162.1_ | 616996 | CCL22 | chemokine (C-C motif) ligand 22 | Extracellular Space | cytokine | L | 3.144 | 0.000 | -2.312 | 0.000 | -1.853 | 0.002 |
| gi_118150781_ref_NM_001077828.1_ | 281239 | IGF1 | insulin-like growth factor 1 (somatomedin C) | Extracellular Space | growth factor | L | 2.780 | 0.000 | 1.475 | 0.726 | 1.528 | 0.684 |
| gi_119905043_ref_XM_001251494.1_ | 783681 | JAG1 | jagged 1 (Alagille syndrome) | Extracellular Space | growth factor | L | 2.457 | 0.000 | 1.236 | 1.257 | 1.019 | 1.104 |
| gi_114052243_ref_NM_001046358.1_ | 533671 | CMTM8 | CKLF-like MARVEL transmembrane domain containin | Extracellular Space | cytokine | H | 2.427 | 0.000 | -1.780 | 0.002 | -2.289 | 0.000 |
| gi_31343250_ref_NM_173925.2_ | 280828 | IL8 | interleukin 8 | Extracellular Space | cytokine | L | 2.216 | 0.000 | -1.141 | 0.520 | 1.819 | 0.275 |
| gi_77736244_ref_NM_001034647.1_ | 538567 | TNFSF13 | tumor necrosis factor (ligand) superfamily, member 13 | Extracellular Space | cytokine | M | 2.077 | 0.001 | -1.465 | 0.041 | -1.767 | 0.004 |
| gi_119911822_ref_XM_001254761.1_ | 788103 | TNFSF12 | tumor necrosis factor (ligand) superfamily, member 12 | Extracellular Space | cytokine | H | 1.977 | 0.001 | 1.082 | 1.145 | 1.034 | 1.157 |
| gi_75812913_ref_NM_001033608.1_ | 280858 | MIF | macrophage migration inhibitory factor (glycosylation-i | Extracellular Space | cytokine | VH | 1.941 | 0.001 | -2.126 | 0.000 | -2.049 | 0.000 |
| gi_119887028_ref_XM_592594.3_ | 514701 | FAM3B | family with sequence similarity 3, member B | Extracellular Space | cytokine | N | 1.684 | 0.005 | 1.487 | 0.585 | 1.344 | 1.116 |
| gi_31341809_ref_NM_181030.2_ | 282233 | FLT3LG | fms-related tyrosine kinase 3 ligand | Extracellular Space | cytokine | H | 1.556 | 0.008 | -1.219 | 0.412 | -1.295 | 0.271 |
| gi_116004250_ref_NM_001077014.1_ | 767942 | GRN | granulin | Extracellular Space | growth factor | H | 1.549 | 0.009 | 1.967 | 0.081 | 2.576 | 0.018 |
| gi_119908328_ref_XM_001252797.1_ | 784352 | FASLG | Fas ligand (TNF superfamily, member 6) | Extracellular Space | cytokine | H | -2.979 | 0.048 | -1.586 | 0.015 | -1.634 | 0.012 |
| gi_116004478_ref_NM_001077129.1_ | 768073 | OSGIN1 | oxidative stress induced growth inhibitor 1 | unknown | growth factor | M | -3.317 | 0.025 | 1.022 | 0.654 | 1.227 | 1.263 |
| gi_119908200_ref_XM_613627.3_ | 534018 | LEFTY2 | left-right determination factor 2 | Extracellular Space | growth factor | M | -3.379 | 0.018 | -1.022 | 0.854 | 1.025 | 1.110 |
| gi_119892499_ref_XR_028517.1_ | 540517 | GDF11 | growth differentiation factor 11 | Extracellular Space | growth factor | H | -3.429 | 0.018 | -1.141 | 0.574 | 1.067 | 1.232 |
| gi_75832046_ref_NM_173935.2_ | 280852 | MDK | midkine (neurite growth-promoting factor 2) | Extracellular Space | growth factor | H | -3.479 | 0.013 | 1.293 | 1.235 | 1.662 | 0.501 |
| gi_119909004_ref_XM_870711.2_ | 618377 | MDK | midkine (neurite growth-promoting factor 2) | Extracellular Space | growth factor | H | -3.567 | 0.011 | 1.382 | 0.952 | 1.502 | 0.832 |
| gi_115497151_ref_NM_001076398.1_ | 615855 | NRADD | neurotrophin receptor associated death domain | Extracellular Space | cytokine | H | -3.640 | 0.010 | 1.102 | 1.150 | 1.400 | 1.068 |
| gi_114052742_ref_NM_001045877.1_ | 407216 | BMP4 | bone morphogenetic protein 4 | Extracellular Space | growth factor | H | -3.758 | 0.009 | 1.575 | 0.505 | 1.952 | 0.181 |
| gi_119909897_ref_XM_869815.2_ | 617538 | IL17C | interleukin 17C | Extracellular Space | cytokine | M | -3.811 | 0.008 | 1.455 | 0.743 | 1.847 | 0.254 |
| gi_119892435_ref_XM_595759.3_ | 517587 | INHBE | inhibin, beta E | Extracellular Space | growth factor | L | -3.836 | 0.008 | 1.076 | 0.918 | 3.535 | 0.001 |
| gi_149643014_ref_NM_001098859.1_ | 407111 | FASLG | Fas ligand (TNF superfamily, member 6) | Extracellular Space | cytokine | M | -4.220 | 0.004 | -2.436 | 0.000 | -2.019 | 0.000 |
| gi_119894894_ref_XM_600015.3_ | 521748 | TNFSF9 | tumor necrosis factor (ligand) superfamily, member 9 | Extracellular Space | cytokine | H | -4.883 | 0.001 | 1.128 | 1.207 | 1.796 | 0.307 |
| gi_134085416_ref_NM_001013401.2_ | 280845 | LTA | lymphotoxin alpha (TNF superfamily, member 1) | Extracellular Space | cytokine | VH | -5.010 | 0.001 | -1.802 | 0.002 | -1.364 | 0.144 |
| gi_114051352_ref_NM_001046182.1_ | 514773 | SOCS5 | suppressor of cytokine signaling 5 | Extracellular Space | cytokine | H | -5.138 | 0.001 | 1.341 | 1.104 | 1.326 | 1.180 |
| gi_31342941_ref_NM_174055.2_ | 281160 | FGF1 | fibroblast growth factor 1 (acidic) | Extracellular Space | growth factor | M | -5.214 | 0.001 | 1.329 | 0.957 | 1.623 | 0.552 |
| gi_119910851_ref_XM_602991.3_ | 524661 | FGF21 | fibroblast growth factor 21 | Extracellular Space | growth factor | M | -5.561 | 0.000 | 1.345 | 0.929 | 1.701 | 0.415 |
| gi_77735642_ref_NM_001034345.1_ | 509167 | CCL19 | chemokine (C-C motif) ligand 19 | Extracellular Space | cytokine | M | -5.654 | 0.000 | 2.513 | 0.005 | 2.938 | 0.006 |
| gi_119923438_ref_XM_615898.3_ | 521961 | VAV3 | vav 3 guanine nucleotide exchange factor | Extracellular Space | cytokine | VH | -6.007 | 0.000 | 2.171 | 0.027 | 1.711 | 0.415 |
| gi_119908015_ref_XM_604649.3_ | 526285 | IL24 | interleukin 24 | Extracellular Space | cytokine | VH | -6.127 | 0.000 | 1.169 | 1.100 | 1.042 | 0.915 |
| gi_139948854_ref_NM_001083706.1_ | 525931 | PDGFD | platelet derived growth factor D | Extracellular Space | growth factor | VH | -8.249 | 0.000 | 1.622 | 0.413 | 2.979 | 0.005 |
| gi_119894908_ref_XM_600347.3_ | 522074 | CD70 | CD70 molecule | Extracellular Space | cytokine | H | -13.147 | 0.000 | 3.857 | 0.000 | 8.446 | 0.000 |
| gi_31342868_ref_NM_174091.2_ | 281249 | IL18 | interleukin 18 (interferon-gamma-inducing factor) | Extracellular Space | cytokine | H | -13.668 | 0.000 | 3.652 | 0.000 | 4.042 | 0.000 |
| gi_119904450_ref_XM_591585.3_ | 513836 | TNFSF11 | tumor necrosis factor (ligand) superfamily, member 11 | Extracellular Space | cytokine | H | -22.409 | 0.000 | -1.019 | 0.692 | 1.336 | 1.085 |
| gi_72534795_ref_NM_001031751.1_ | 282879 | HGF | hepatocyte growth factor (hepapoietin A; scatter factor | Extracellular Space | growth factor | H | -58.668 | 0.000 | -1.340 | 0.110 | -1.401 | 0.083 |
| gi_41386773_ref_NM_174090.1_ | 281248 | IL15 | interleukin 15 | Extracellular Space | cytokine | H | -60.142 | 0.000 | 1.041 | 1.014 | 1.059 | 1.227 |

## Table S6B: Cell Surface - receptors

| SEQ_ID | Entrez gene ID | Symbol | Entrez Gene Name | Location | Type | Predicted BL20 expression level | FC BLvTBL | FDR BLvsTBL | FC TBLvTBL24h | FDR TBLvTBL24h | FC TBLvTBL48h | FDR TBLvTBL48h |
|---|---|---|---|---|---|---|---|---|---|---|---|---|
| gi_119894051_ref_XM_587301.3_ | 510185 | IL2RB | interleukin 2 receptor, beta | Plasma Membrane | transmembrane receptor | M | 80.276 | 0.000 | 1.023 | 1.141 | 1.072 | 1.277 |

| | | | | | | | | | | | | |
|---|---|---|---|---|---|---|---|---|---|---|---|---|
| gi_134085682_ref_NM_001083479.1_ | 534536 | ACVRL1 | activin A receptor type II-like 1 | Plasma Membrane | kinase | L | 38.543 | 0.000 | -1.041 | 0.905 | -1.068 | 0.869 |
| gi_31342230_ref_NM_174358.2_ | 281861 | IL2RA | interleukin 2 receptor, alpha | Plasma Membrane | transmembrane receptor | L | 25.884 | 0.000 | -1.401 | 0.092 | 2.023 | 0.131 |
| gi_31342240_ref_NM_174348.2_ | 281839 | ICAM1 | intercellular adhesion molecule 1 | Plasma Membrane | transmembrane receptor | H | 18.031 | 0.000 | -1.932 | 0.000 | -1.223 | 0.453 |
| gi_119917021_ref_XM_870302.2_ | 617967 | IL21R | interleukin 21 receptor | Plasma Membrane | transmembrane receptor | M | 12.421 | 0.000 | -1.055 | 0.913 | 1.166 | 1.340 |
| gi_166999226_ref_NM_001007810.3_ | 493645 | C5AR1 | complement component 5a receptor 1 | Plasma Membrane | G-protein coupled recept | H | 12.023 | 0.000 | -1.514 | 0.029 | 1.006 | 1.122 |
| gi_119893058_ref_XM_868762.2_ | 616674 | CLEC4A | C-type lectin domain family 4, member A | Plasma Membrane | transmembrane receptor | N | 10.246 | 0.000 | 3.657 | 0.000 | 1.866 | 0.251 |
| gi_114051118_ref_NM_001046210.1_ | 515700 | IL1R2 | interleukin 1 receptor, type II | Plasma Membrane | transmembrane receptor | L | 9.818 | 0.000 | 2.911 | 0.001 | 1.608 | 0.590 |
| gi_77735630_ref_NM_001034339.1_ | 508932 | IL11RA | interleukin 11 receptor, alpha | Plasma Membrane | transmembrane receptor | M | 8.542 | 0.000 | -1.063 | 0.743 | 1.012 | 1.116 |
| gi_156523227_ref_NM_001102558.1_ | 100124525 | CX3CR1 | chemokine (C-X3-C motif) receptor 1 | Plasma Membrane | G-protein coupled recept | L | 8.178 | 0.000 | 55.305 | 0.000 | 137.187 | 0.000 |
| gi_62988287_ref_NM_001017937.1_ | 507837 | BAIAP2 | BAI1-associated protein 2 | Plasma Membrane | kinase | M | 7.760 | 0.000 | 1.181 | 1.208 | 1.899 | 0.207 |
| gi_31341597_ref_NM_174588.2_ | 282329 | PTGER2 | prostaglandin E receptor 2 (subtype EP2), 53kDa | Plasma Membrane | G-protein coupled recept | N | 6.462 | 0.000 | -1.461 | 0.050 | -3.223 | 0.000 |
| gi_89886438_ref_NM_001039726.1_ | 281422 | PRLR | prolactin receptor | Plasma Membrane | transmembrane receptor | N | 6.067 | 0.000 | -2.220 | 0.000 | -4.282 | 0.000 |
| gi_115496689_ref_NM_001075699.1_ | 515172 | P2RY10 | purinergic receptor P2Y, G-protein coupled, 10 | Plasma Membrane | G-protein coupled recept | M | 5.878 | 0.000 | 1.914 | 0.104 | 1.921 | 0.203 |
| gi_115495646_ref_NM_001075139.1_ | 404058 | KLRK1 | killer cell lectin-like receptor subfamily K, member 1 | Plasma Membrane | transmembrane receptor | M | 5.551 | 0.000 | 1.339 | 1.144 | 1.344 | 1.234 |
| gi_119888721_ref_XM_612644.3_ | 540687 | GPR3 | G protein-coupled receptor 3 | Plasma Membrane | G-protein coupled recept | L | 4.415 | 0.000 | -3.230 | 0.000 | -5.521 | 0.000 |
| gi_119903179_ref_XM_871776.2_ | 520709 | IL1RL1 | interleukin 1 receptor-like 1 | Plasma Membrane | transmembrane receptor | N | 4.195 | 0.000 | -2.315 | 0.000 | -3.775 | 0.000 |
| gi_76626421_ref_XM_599517.2_ | 521257 | HRH2 | histamine receptor H2 | Plasma Membrane | G-protein coupled recept | L | 4.103 | 0.000 | -1.896 | 0.001 | -1.641 | 0.011 |
| gi_31343037_ref_NM_174014.2_ | 281058 | CD69 | CD69 molecule | Plasma Membrane | transmembrane receptor | M | 4.063 | 0.000 | -1.724 | 0.004 | -2.905 | 0.000 |
| gi_119904648_ref_XM_590263.3_ | 512700 | FLT3 | fms-related tyrosine kinase 3 | Plasma Membrane | kinase | H | 3.727 | 0.000 | 2.044 | 0.055 | 2.633 | 0.014 |
| gi_31342041_ref_NM_174423.2_ | 281983 | PLAUR | plasminogen activator, urokinase receptor | Plasma Membrane | transmembrane receptor | H | 3.707 | 0.000 | -1.666 | 0.007 | -1.539 | 0.033 |
| gi_31340793_ref_NM_174662.2_ | 282488 | FAS | Fas (TNF receptor superfamily, member 6) | Plasma Membrane | transmembrane receptor | M | 3.517 | 0.000 | 1.007 | 1.113 | -1.089 | 0.854 |
| gi_119918814_ref_XM_869491.2_ | 445416 | FZD4 | frizzled homolog 4 (Drosophila) | Plasma Membrane | G-protein coupled recept | M | 3.502 | 0.000 | 1.008 | 1.091 | -2.685 | 0.000 |
| gi_119889339_ref_XM_613650.3_ | 353111 | NTRK1 | neurotrophic tyrosine kinase, receptor, type 1 | Plasma Membrane | kinase | L | 3.454 | 0.000 | -1.281 | 0.224 | -2.685 | 0.000 |
| gi_119889854_ref_XM_001253071.1_ | 784894 | TGFBR3 | transforming growth factor, beta receptor III | Plasma Membrane | kinase | L | 3.377 | 0.000 | 5.319 | 0.000 | 10.447 | 0.000 |
| gi_119912530_ref_XM_607489.3_ | 529049 | MRC2 | mannose receptor, C type 2 | Plasma Membrane | transmembrane receptor | M | 3.353 | 0.000 | -2.126 | 0.000 | 1.743 | 0.369 |
| gi_31342610_ref_NM_174197.2_ | 281534 | TLR2 | toll-like receptor 2 | Plasma Membrane | transmembrane receptor | M | 3.246 | 0.000 | 2.619 | 0.004 | 2.615 | 0.015 |
| gi_118150987_ref_NM_001077947.1_ | 522144 | GPR77 | G protein-coupled receptor 77 | Plasma Membrane | G-protein coupled recept | L | 3.184 | 0.000 | 1.331 | 0.898 | -1.010 | 0.692 |
| gi_94966784_ref_NM_001040490.1_ | 338033 | TNFRSF1B | tumor necrosis factor receptor superfamily, member 1E | Plasma Membrane | transmembrane receptor | VH | 3.062 | 0.000 | -1.794 | 0.002 | -1.251 | 0.367 |
| gi_119914227_ref_XM_585197.3_ | 539146 | GPR132 | G protein-coupled receptor 132 | Plasma Membrane | G-protein coupled recept | H | 3.039 | 0.000 | -1.007 | 1.028 | 1.153 | 1.330 |
| gi_28603783_ref_NM_176661.1_ | 338066 | CD97 | CD97 molecule | Plasma Membrane | G-protein coupled recept | H | 3.000 | 0.000 | -1.033 | 0.930 | 1.015 | 1.173 |
| gi_139948963_ref_NM_001083752.1_ | 540702 | C3AR1 | complement component 3a receptor 1 | Plasma Membrane | G-protein coupled recept | N | 2.761 | 0.000 | 1.144 | 1.214 | 1.811 | 0.291 |
| gi_76608734_ref_XM_582381.2_ | 538740 | GPR15 | G protein-coupled receptor 15 | Plasma Membrane | G-protein coupled recept | L | 2.612 | 0.000 | 4.919 | 0.000 | 11.340 | 0.000 |
| gi_110350680_ref_NM_001011676.2_ | 497022 | CD2 | CD2 molecule | Plasma Membrane | transmembrane receptor | L | 2.588 | 0.000 | 2.912 | 0.000 | 4.218 | 0.000 |
| gi_119903192_ref_XM_590497.3_ | 407221 | IL18R1 | interleukin 18 receptor 1 | Plasma Membrane | transmembrane receptor | L | 2.478 | 0.000 | -2.446 | 0.000 | -2.324 | 0.000 |
| gi_119907079_ref_XM_591164.3_ | 513478 | IL10RA | interleukin 10 receptor, alpha | Plasma Membrane | transmembrane receptor | M | 2.429 | 0.000 | -1.112 | 0.602 | 1.218 | 1.302 |
| gi_119893892_ref_XM_612028.3_ | 280832 | KIT | v-kit Hardy-Zuckerman 4 feline sarcoma viral oncogen | Plasma Membrane | kinase | N | 2.351 | 0.000 | 1.445 | 0.720 | 6.956 | 0.000 |
| gi_119892995_ref_XM_607058.3_ | 536328 | LRP6 | low density lipoprotein receptor-related protein 6 | Plasma Membrane | transmembrane receptor | L | 2.267 | 0.000 | -1.015 | 0.969 | -1.091 | 0.758 |
| gi_119925169_ref_XM_594146.3_ | 444870 | TLR5 | toll-like receptor 5 | Plasma Membrane | transmembrane receptor | L | 2.242 | 0.000 | 1.485 | 0.689 | -1.003 | 1.020 |
| gi_27806448_ref_NM_174162.1_ | 281439 | DAG1 | dystroglycan 1 (dystrophin-associated glycoprotein 1) | Plasma Membrane | transmembrane receptor | H | 2.210 | 0.000 | -1.750 | 0.004 | -1.143 | 0.703 |
| gi_117935052_ref_NM_001024930.2_ | 510668 | CCR7 | chemokine (C-C motif) receptor 7 | Plasma Membrane | G-protein coupled recept | VH | 2.134 | 0.000 | -1.981 | 0.000 | -1.794 | 0.004 |
| gi_119903186_ref_XM_593695.3_ | 515640 | IL1R1 | interleukin 1 receptor, type I | Plasma Membrane | transmembrane receptor | VH | 2.074 | 0.000 | 1.074 | 1.201 | 1.104 | 1.315 |
| gi_114052594_ref_NM_001046316.1_ | 531137 | LGALS3BP | lectin, galactoside-binding, soluble, 3 binding protein | Plasma Membrane | transmembrane receptor | VH | 2.049 | 0.001 | 1.165 | 1.246 | 1.209 | 1.340 |
| gi_119895013_ref_XM_587940.3_ | 539534 | S1PR4 | sphingosine-1-phosphate receptor 4 | Plasma Membrane | G-protein coupled recept | L | 2.017 | 0.001 | -1.238 | 0.199 | -3.049 | 0.000 |
| gi_119912855_ref_XM_603404.3_ | 525061 | CD300C | CD300c molecule | Plasma Membrane | transmembrane receptor | M | 1.980 | 0.001 | -1.071 | 0.757 | 1.049 | 1.123 |
| gi_119879744_ref_XM_613645.3_ | 540907 | EPHB3 | EPH receptor B3 | Plasma Membrane | kinase | L | 1.944 | 0.001 | -1.247 | 0.346 | -1.220 | 0.420 |
| gi_119902362_ref_XM_001253886.1_ | 788224 | TYRO3 | TYRO3 protein tyrosine kinase | Plasma Membrane | kinase | M | 1.827 | 0.002 | 1.304 | 1.066 | -1.087 | 0.741 |
| gi_119894537_ref_XM_602621.3_ | 524299 | IL12RB1 | interleukin 12 receptor, beta 1 | Plasma Membrane | transmembrane receptor | L | 1.807 | 0.002 | -2.010 | 0.000 | -2.047 | 0.000 |
| gi_119915419_ref_XM_001255620.1_ | 788613 | MICB | MHC class I polypeptide-related sequence B | Plasma Membrane | transmembrane receptor | L | 1.803 | 0.003 | -2.747 | 0.000 | -1.894 | 0.002 |
| gi_119890635_ref_XM_581281.3_ | 505056 | GPR35 | G protein-coupled receptor 35 | Plasma Membrane | G-protein coupled recept | M | 1.802 | 0.002 | 1.797 | 0.183 | 2.359 | 0.038 |
| gi_77736420_ref_NM_001034738.1_ | 613482 | LTB4R | leukotriene B4 receptor | Plasma Membrane | G-protein coupled recept | H | 1.771 | 0.002 | -2.302 | 0.000 | -1.560 | 0.024 |
| gi_149642840_ref_NM_001099043.1_ | 528782 | TNFRSF4 | tumor necrosis factor receptor superfamily, member 4 | Plasma Membrane | transmembrane receptor | M | 1.760 | 0.003 | -3.008 | 0.000 | -1.701 | 0.006 |
| gi_119914870_ref_XR_027440.1_ | 781625 | PLXND1 | plexin D1 | Plasma Membrane | transmembrane receptor | H | 1.740 | 0.003 | -1.541 | 0.023 | -1.449 | 0.068 |
| gi_27806206_ref_NM_174499.1_ | 282135 | ADRA2A | adrenergic, alpha-2A-, receptor | Plasma Membrane | G-protein coupled recept | L | 1.724 | 0.006 | -1.908 | 0.000 | -1.879 | 0.002 |
| gi_119888952_ref_XM_590380.3_ | 512798 | EPHA2 | EPH receptor A2 | Plasma Membrane | kinase | M | 1.718 | 0.005 | -1.305 | 0.194 | -1.120 | 0.658 |
| gi_147898888_ref_NM_001098163.1_ | 100036590 | KLRC1 | killer cell lectin-like receptor subfamily C, member 1 | Plasma Membrane | transmembrane receptor | N | 1.654 | 0.007 | -1.067 | 0.369 | -1.611 | 0.011 |
| gi_119923923_ref_XM_001249398.1_ | 781056 | GPR174 | G protein-coupled receptor 174 | Plasma Membrane | G-protein coupled recept | H | 1.623 | 0.005 | -1.400 | 0.092 | -1.927 | 0.001 |
| gi_94966908_ref_NM_001040554.1_ | 782684 | HLA-A | major histocompatibility complex, class I, A | Plasma Membrane | transmembrane receptor | H | 1.608 | 0.007 | -1.020 | 0.852 | -1.108 | 0.577 |
| gi_114051336_ref_NM_001046183.1_ | 514832 | PEX5 | peroxisomal biogenesis factor 5 | Cytoplasm | transmembrane receptor | H | 1.589 | 0.006 | -1.236 | 0.400 | -1.116 | 0.776 |
| gi_119893717_ref_XM_614752.3_ | 541147 | GPR125 | G protein-coupled receptor 125 | Plasma Membrane | G-protein coupled recept | VH | 1.576 | 0.009 | 1.167 | 1.248 | 1.078 | 1.259 |
| gi_139949072_ref_NM_001083656.1_ | 509703 | GPR56 | G protein-coupled receptor 56 | Plasma Membrane | G-protein coupled recept | L | 1.574 | 0.010 | 1.261 | 1.233 | 1.155 | 1.313 |
| gi_77736382_ref_NM_001034655.1_ | 538903 | SIGMAR1 | sigma non-opioid intracellular receptor 1 | Plasma Membrane | G-protein coupled recept | VH | 1.542 | 0.009 | -1.172 | 0.560 | -1.668 | 0.010 |
| gi_31341778_ref_NM_174516.2_ | 282179 | CHRNB1 | cholinergic receptor, nicotinic, beta 1 (muscle) | Plasma Membrane | transmembrane receptor | H | 1.522 | 0.011 | -1.761 | 0.002 | -1.767 | 0.005 |
| gi_119903188_ref_XM_600388.3_ | 522114 | IL1RL2 | interleukin 1 receptor-like 2 | Plasma Membrane | transmembrane receptor | L | 1.519 | 0.019 | 1.334 | 0.847 | 1.619 | 0.438 |
| gi_155372282_ref_NM_001101284.1_ | 782518 | LPAR6 | lysophosphatidic acid receptor 6 | Plasma Membrane | G-protein coupled recept | L | 1.483 | 0.018 | -1.242 | 0.311 | 1.628 | 0.530 |
| gi_119889654_ref_XM_618390.3_ | 538194 | CELSR2 | cadherin, EGF LAG seven-pass G-type receptor 2 (flai | Plasma Membrane | G-protein coupled recept | H | 1.465 | 0.022 | -3.904 | 0.000 | -1.117 | 0.682 |
| gi_119894271_ref_XM_610839.3_ | 532327 | FGFRL1 | fibroblast growth factor receptor-like 1 | Plasma Membrane | transmembrane receptor | M | 1.454 | 0.023 | -1.387 | 0.096 | -1.318 | 0.208 |

| | | | | | | | | | | | | |
|---|---|---|---|---|---|---|---|---|---|---|---|---|
| gi_156120416_ref_NM_001101884.1_ | 507427 | RIPK3 | receptor-interacting serine-threonine kinase 3 | Plasma Membrane | kinase | H | 1.438 | 0.021 | 1.224 | 1.253 | 1.643 | 0.537 |
| gi_31342195_ref_NM_174368.2_ | 281876 | ITGB1 | integrin, beta 1 (fibronectin receptor, beta polypeptide, | Plasma Membrane | transmembrane receptor | VH | 1.414 | 0.025 | 1.010 | 1.116 | 1.139 | 1.330 |
| gi_119920696_ref_XM_608251.3_ | 529792 | CRLF2 | cytokine receptor-like factor 2 | Plasma Membrane | transmembrane receptor | L | 1.395 | 0.046 | 1.273 | 1.211 | -1.493 | 0.037 |
| gi_78045494_ref_NM_001035048.1_ | 507439 | NMUR2 | neuromedin U receptor 2 | Plasma Membrane | G-protein coupled recept | L | 1.359 | 0.048 | 1.751 | 0.164 | -1.040 | 0.611 |
| gi_155372320_ref_NM_001101303.1_ | 788507 | GPR65 | G protein-coupled receptor 65 | Plasma Membrane | G-protein coupled recept | VH | 1.357 | 0.038 | 1.381 | 1.031 | -1.122 | 0.724 |
| gi_31341841_ref_NM_174495.2_ | 282131 | ACVR2B | activin A receptor, type IIB | Plasma Membrane | kinase | H | 1.341 | 0.047 | -1.047 | 0.939 | 1.488 | 0.865 |
| gi_119894606_ref_XM_588006.3_ | 510803 | PGLYRP2 | peptidoglycan recognition protein 2 | Plasma Membrane | transmembrane receptor | L | 1.326 | 0.054 | -1.116 | 0.610 | -1.564 | 0.018 |
| gi_119910893_ref_XM_582800.3_ | 506364 | SIGLEC8 | sialic acid binding Ig-like lectin 8 | Plasma Membrane | transmembrane receptor | L | -2.953 | 0.054 | 1.368 | 0.967 | 1.432 | 0.983 |
| gi_147900316_ref_NM_001098123.1_ | 541056 | GRM3 | glutamate receptor, metabotropic 3 | Plasma Membrane | G-protein coupled recept | M | -2.985 | 0.050 | 2.766 | 0.002 | 3.379 | 0.002 |
| gi_31341426_ref_NM_173878.2_ | 280686 | F3 | coagulation factor III (thromboplastin, tissue factor) | Plasma Membrane | transmembrane receptor | M | -3.014 | 0.048 | 2.364 | 0.010 | 2.742 | 0.010 |
| gi_31342593_ref_NM_174206.2_ | 281553 | TSHR | thyroid stimulating hormone receptor | Plasma Membrane | G-protein coupled recept | L | -3.063 | 0.047 | -1.041 | 0.743 | 1.092 | 1.152 |
| gi_110347601_ref_NM_001040481.2_ | 282491 | HLA-DMB | major histocompatibility complex, class II, DM beta | Plasma Membrane | transmembrane receptor | VH | -3.219 | 0.027 | 1.329 | 1.142 | -1.026 | 0.995 |
| gi_119890574_ref_XM_871140.2_ | 618817 | OR6B2 | olfactory receptor, family 6, subfamily B, member 2 | Plasma Membrane | G-protein coupled recept | L | -3.255 | 0.029 | 1.799 | 0.152 | 2.068 | 0.115 |
| gi_119920926_ref_XM_586199.3_ | 509267 | GM6495 | predicted gene 6495 | Plasma Membrane | G-protein coupled recept | M | -3.279 | 0.023 | 1.337 | 1.126 | 1.464 | 0.939 |
| gi_156121074_ref_NM_001102214.1_ | 538495 | ILDR1 | immunoglobulin-like domain containing receptor 1 | Plasma Membrane | transmembrane receptor | L | -3.299 | 0.025 | 1.098 | 1.153 | 1.805 | 0.301 |
| gi_76677886_ref_NM_001033937.1_ | 532262 | TLR8 (includes EG:! | toll-like receptor 8 | Plasma Membrane | transmembrane receptor | L | -3.302 | 0.025 | 1.069 | 1.104 | 1.207 | 1.319 |
| gi_73853749_ref_NM_174011.3_ | 281054 | CD3E | CD3e molecule, epsilon (CD3-TCR complex) | Plasma Membrane | transmembrane receptor | M | -3.312 | 0.022 | 1.175 | 1.235 | -1.225 | 0.418 |
| gi_119901592_ref_XR_027451.1_ | 534358 | UTRN | utrophin | Plasma Membrane | transmembrane receptor | VH | -3.333 | 0.021 | 1.618 | 0.387 | 2.313 | 0.044 |
| gi_119910556_ref_XM_594754.3_ | 516598 | AXL | AXL receptor tyrosine kinase | Plasma Membrane | kinase | L | -3.363 | 0.025 | 1.330 | 1.135 | 1.682 | 0.467 |
| gi_119895312_ref_XM_866364.2_ | 614762 | OR2G3 | olfactory receptor, family 2, subfamily G, member 3 | Plasma Membrane | G-protein coupled recept | L | -3.432 | 0.019 | 1.295 | 0.985 | 1.625 | 0.539 |
| gi_76669265_ref_XM_590306.2_ | 512736 | OR52J3 | olfactory receptor, family 52, subfamily J, member 3 | Plasma Membrane | G-protein coupled recept | L | -3.464 | 0.015 | 1.468 | 0.740 | 2.520 | 0.021 |
| gi_119914827_ref_XM_589282.3_ | 408018 | CCR3 | chemokine (C-C motif) receptor 3 | Plasma Membrane | G-protein coupled recept | H | -3.492 | 0.016 | -1.997 | 0.000 | -1.834 | 0.002 |
| gi_148277667_ref_NM_174547.2_ | 282244 | GUCY2C | guanylate cyclase 2C (heat stable enterotoxin receptor | Plasma Membrane | kinase | L | -3.620 | 0.012 | 1.259 | 1.210 | 1.214 | 1.306 |
| gi_158819080_ref_NM_001110191.1_ | 100126272 | CD302 | CD302 molecule | Plasma Membrane | transmembrane receptor | H | -3.631 | 0.010 | 2.071 | 0.048 | 1.437 | 0.966 |
| gi_119879508_ref_XM_617543.3_ | 407131 | CD80 | CD80 molecule | Plasma Membrane | transmembrane receptor | H | -3.651 | 0.010 | 2.625 | 0.004 | 3.352 | 0.002 |
| gi_119921029_ref_XM_001252156.1_ | 783597 | OLFR617 | olfactory receptor 617 | Plasma Membrane | G-protein coupled recept | L | -3.671 | 0.013 | 3.389 | 0.000 | 12.014 | 0.000 |
| gi_34147166_ref_NM_183081.1_ | 282602 | TLR9 | toll-like receptor 9 | Plasma Membrane | transmembrane receptor | H | -3.686 | 0.009 | 2.333 | 0.012 | 3.138 | 0.003 |
| gi_76657295_ref_XM_869298.1_ | 617108 | OLFR890 | olfactory receptor 890 | Plasma Membrane | G-protein coupled recept | L | -3.760 | 0.010 | 2.601 | 0.004 | 4.413 | 0.000 |
| gi_119902238_ref_XM_866698.2_ | 615014 | OLFR49 | olfactory receptor 49 | Plasma Membrane | G-protein coupled recept | M | -3.788 | 0.009 | 2.040 | 0.053 | 2.609 | 0.016 |
| gi_119926223_ref_XM_588414.2_ | 539623 | OR2J2 | olfactory receptor, family 2, subfamily J, member 2 | Plasma Membrane | G-protein coupled recept | M | -3.794 | 0.007 | 2.277 | 0.015 | 3.038 | 0.005 |
| gi_164448559_ref_NM_174266.3_ | 281674 | CD79A | CD79a molecule, immunoglobulin-associated alpha | Plasma Membrane | transmembrane receptor | VH | -3.830 | 0.006 | 1.533 | 0.605 | 1.975 | 0.163 |
| gi_31341726_ref_NM_174539.2_ | 282229 | FCGR2B | Fc fragment of IgG, low affinity IIb, receptor (CD32) | Plasma Membrane | transmembrane receptor | H | -3.841 | 0.006 | 3.525 | 0.000 | 6.007 | 0.000 |
| gi_187937198_ref_NM_001127317.1_ | 533051 | MICB | MHC class I polypeptide-related sequence B | Plasma Membrane | transmembrane receptor | VH | -3.961 | 0.005 | 2.154 | 0.030 | 3.242 | 0.003 |
| gi_119924734_ref_XM_001251058.1_ | 782431 | OR2W1 | olfactory receptor, family 2, subfamily W, member 1 | Plasma Membrane | G-protein coupled recept | M | -3.970 | 0.005 | 1.464 | 0.630 | 2.400 | 0.032 |
| gi_119915533_ref_XM_001251657.1_ | 783002 | OR2B2 | olfactory receptor, family 2, subfamily B, member 2 | Plasma Membrane | G-protein coupled recept | M | -3.984 | 0.006 | 3.159 | 0.000 | 2.918 | 0.007 |
| gi_119913108_ref_XM_600430.3_ | 522155 | IL6ST | interleukin 6 signal transducer (gp130, oncostatin M re | Plasma Membrane | transmembrane receptor | H | -4.092 | 0.004 | 1.783 | 0.203 | 1.514 | 0.800 |
| gi_119912528_ref_XM_616376.3_ | 282642 | ITGB3 | integrin, beta 3 (platelet glycoprotein IIIa, antigen CD6 | Plasma Membrane | transmembrane receptor | L | -4.099 | 0.004 | 1.910 | 0.095 | 2.263 | 0.054 |
| gi_154152062_ref_NM_001100304.1_ | 506159 | GPR52 | G protein-coupled receptor 52 | Plasma Membrane | G-protein coupled recept | L | -4.119 | 0.005 | 2.269 | 0.016 | 1.907 | 0.207 |
| gi_119915602_ref_XM_001253092.1_ | 784917 | OLFR129 | olfactory receptor 129 | Plasma Membrane | G-protein coupled recept | M | -4.215 | 0.004 | 1.790 | 0.196 | 2.376 | 0.036 |
| gi_99028970_ref_NM_001012674.2_ | 282490 | HLA-DMA | major histocompatibility complex, class II, DM alpha | Plasma Membrane | transmembrane receptor | VH | -4.249 | 0.003 | 1.216 | 1.257 | 1.062 | 1.238 |
| gi_119900522_ref_XM_001251357.1_ | 783725 | CD72 | CD72 molecule | Plasma Membrane | transmembrane receptor | H | -4.306 | 0.003 | 2.136 | 0.034 | 2.555 | 0.019 |
| gi_119910464_ref_XM_605910.3_ | 527517 | FFAR3 | free fatty acid receptor 3 | Plasma Membrane | G-protein coupled recept | M | -4.411 | 0.004 | 1.164 | 1.055 | 1.036 | 0.872 |
| gi_116004060_ref_NM_001076918.1_ | 539791 | TLR10 | toll-like receptor 10 | Plasma Membrane | transmembrane receptor | L | -4.414 | 0.003 | 1.198 | 1.235 | 2.227 | 0.061 |
| gi_119911318_ref_XM_001256784.1_ | 790265 | VN1R1 | vomeronasal 1 receptor 1 | Plasma Membrane | G-protein coupled recept | L | -4.618 | 0.003 | 1.320 | 1.077 | 1.743 | 0.350 |
| gi_119893047_ref_XM_613380.3_ | 533844 | CD163 | CD163 molecule | Plasma Membrane | transmembrane receptor | M | -4.650 | 0.002 | 4.651 | 0.000 | 17.050 | 0.000 |
| gi_62460553_ref_NM_001014929.1_ | 515011 | ITGA2B (includes E | integrin, alpha 2b (platelet glycoprotein IIb of IIb/IIIa co | Plasma Membrane | transmembrane receptor | H | -4.680 | 0.002 | 2.023 | 0.054 | 9.254 | 0.000 |
| gi_58332431_ref_NM_178317.3_ | 352960 | TRIB2 | tribbles homolog 2 (Drosophila) | Plasma Membrane | kinase | VH | -4.720 | 0.002 | -1.246 | 0.367 | 1.021 | 1.161 |
| gi_119895827_ref_XM_001250068.1_ | 407140 | ADRA1B | adrenergic, alpha-1B-, receptor | Plasma Membrane | G-protein coupled recept | H | -4.784 | 0.001 | 1.937 | 0.078 | 2.770 | 0.009 |
| gi_119911392_ref_XM_601098.3_ | 522810 | TEX14 | testis expressed 14 | Plasma Membrane | kinase | M | -4.846 | 0.001 | 1.727 | 0.224 | 2.126 | 0.089 |
| gi_119907588_ref_XM_588870.3_ | 539703 | P2RY6 | pyrimidinergic receptor P2Y, G-protein coupled, 6 | Plasma Membrane | G-protein coupled recept | M | -4.904 | 0.001 | 2.032 | 0.055 | 2.966 | 0.006 |
| gi_155372148_ref_NM_001101214.1_ | 540647 | GPR19 | G protein-coupled receptor 19 | Plasma Membrane | G-protein coupled recept | H | -5.050 | 0.001 | 1.682 | 0.297 | 1.303 | 1.253 |
| gi_77735560_ref_NM_001034304.1_ | 507598 | LPAR5 | lysophosphatidic acid receptor 5 | Plasma Membrane | G-protein coupled recept | H | -5.371 | 0.001 | 5.882 | 0.000 | 7.336 | 0.000 |
| gi_119919670_ref_XM_592079.3_ | 540179 | GPC4 | glypican 4 | Plasma Membrane | transmembrane receptor | M | -5.458 | 0.001 | -1.155 | 0.470 | 1.267 | 1.285 |
| gi_41386730_ref_NM_176657.1_ | 338062 | FCGRT | Fc fragment of IgG, receptor, transporter, alpha | Plasma Membrane | transmembrane receptor | H | -5.615 | 0.000 | 2.746 | 0.002 | 3.519 | 0.001 |
| gi_119919105_ref_XM_001254753.1_ | 337885 | GPR44 | G protein-coupled receptor 44 | Plasma Membrane | G-protein coupled recept | M | -5.669 | 0.000 | 1.542 | 0.367 | 1.446 | 0.906 |
| gi_119915616_ref_XM_870400.2_ | 618070 | OLFR131 | olfactory receptor 131 | Plasma Membrane | G-protein coupled recept | M | -5.819 | 0.000 | 1.628 | 0.402 | 4.000 | 0.000 |
| gi_148223508_ref_NM_001098056.1_ | 523822 | TNFRSF11B | tumor necrosis factor receptor superfamily, member 11 | Plasma Membrane | transmembrane receptor | M | -5.852 | 0.000 | 1.562 | 0.461 | 1.398 | 1.033 |
| gi_115497649_ref_NM_001075583.1_ | 512876 | GPR4 | G protein-coupled receptor 4 | Plasma Membrane | G-protein coupled recept | M | -6.134 | 0.000 | 2.102 | 0.036 | 4.322 | 0.000 |
| gi_31343523_ref_NM_175776.2_ | 281033 | TSPO | translocator protein (18kDa) | Cytoplasm | transmembrane receptor | H | -6.284 | 0.000 | 1.025 | 1.049 | 1.986 | 0.155 |
| gi_95147663_ref_NM_001040472.1_ | 281055 | CD3G | CD3g molecule, gamma (CD3-TCR complex) | Plasma Membrane | transmembrane receptor | VH | -6.452 | 0.000 | 2.617 | 0.004 | 3.186 | 0.003 |
| gi_119915382_ref_XM_582099.3_ | 538700 | HLA-DRB5 | major histocompatibility complex, class II, DR beta 5 | Plasma Membrane | transmembrane receptor | H | -6.499 | 0.000 | 1.262 | 1.251 | 1.364 | 1.171 |
| gi_119913259_ref_XM_599818.3_ | 521554 | IL7R | interleukin 7 receptor | Plasma Membrane | transmembrane receptor | H | -6.767 | 0.000 | 1.198 | 1.256 | -1.113 | 0.687 |
| gi_118150797_ref_NM_001077839.1_ | 407771 | CCR1 | chemokine (C-C motif) receptor 1 | Plasma Membrane | G-protein coupled recept | H | -6.915 | 0.000 | 1.057 | 1.118 | 1.002 | 0.971 |
| gi_119921486_ref_XM_582624.3_ | 506206 | MR1 | major histocompatibility complex, class I-related | Plasma Membrane | transmembrane receptor | H | -7.033 | 0.000 | 3.997 | 0.000 | 5.540 | 0.000 |
| gi_31341591_ref_NM_174589.2_ | 282331 | PTGER4 | prostaglandin E receptor 4 (subtype EP4) | Plasma Membrane | G-protein coupled recept | H | -7.034 | 0.000 | 1.555 | 0.532 | 1.932 | 0.187 |
| gi_115494923_ref_NM_001075952.1_ | 531519 | ASGR2 | asialoglycoprotein receptor 2 | Plasma Membrane | transmembrane receptor | H | -7.502 | 0.000 | 1.712 | 0.285 | 3.466 | 0.001 |

| | | | | | | | | | | | | |
|---|---|---|---|---|---|---|---|---|---|---|---|---|
| gi_164448623_ref_NM_001034564.2_ | 528174 | GPR133 | G protein-coupled receptor 133 | Plasma Membrane | G-protein coupled recept | M | -7.570 | 0.000 | -1.216 | 0.318 | 1.014 | 1.051 |
| gi_76628635_ref_XM_594575.2_ | 516422 | ADRA2B | adrenergic, alpha-2B-, receptor | Plasma Membrane | G-protein coupled recept | M | -8.283 | 0.000 | 1.639 | 0.295 | 2.255 | 0.055 |
| gi_119891802_ref_XM_598424.3_ | 520189 | LGR5 | leucine-rich repeat-containing G protein-coupled recep | Plasma Membrane | G-protein coupled recept | H | -8.580 | 0.000 | 1.119 | 1.203 | -3.245 | 0.000 |
| gi_77736322_ref_NM_001034689.1_ | 540038 | GPR18 | G protein-coupled receptor 18 | Plasma Membrane | G-protein coupled recept | H | -8.868 | 0.000 | 2.327 | 0.013 | 1.879 | 0.227 |
| gi_76253706_ref_NM_174301.3_ | 281736 | CXCR4 | chemokine (C-X-C motif) receptor 4 | Plasma Membrane | G-protein coupled recept | VH | -9.677 | 0.000 | 4.072 | 0.000 | 3.127 | 0.004 |
| gi_119894939_ref_XM_582663.3_ | 538783 | TBXA2R | thromboxane A2 receptor | Plasma Membrane | G-protein coupled recept | VH | -10.482 | 0.000 | 2.146 | 0.031 | 2.757 | 0.010 |
| gi_47564061_ref_NM_001001159.1_ | 407237 | TLR6 | toll-like receptor 6 | Plasma Membrane | transmembrane receptor | H | -10.744 | 0.000 | 2.641 | 0.003 | 4.438 | 0.000 |
| gi_119917945_ref_XM_596577.3_ | 518385 | GPR123 | G protein-coupled receptor 123 | Plasma Membrane | G-protein coupled recept | M | -11.584 | 0.000 | 1.007 | 0.958 | -1.014 | 0.715 |
| gi_119915062_ref_XM_604082.3_ | 525727 | HLA-DOA | major histocompatibility complex, class II, DO alpha | Plasma Membrane | transmembrane receptor | VH | -11.787 | 0.000 | 2.613 | 0.004 | 2.799 | 0.009 |
| gi_119892797_ref_XM_606956.3_ | 528530 | CSF2RB | colony stimulating factor 2 receptor, beta, low-affinity (g | Plasma Membrane | transmembrane receptor | H | -12.824 | 0.000 | 5.807 | 0.000 | 17.980 | 0.000 |
| gi_119915324_ref_XM_590184.3_ | 512637 | GPR110 | G protein-coupled receptor 110 | Plasma Membrane | G-protein coupled recept | M | -13.880 | 0.000 | 1.227 | 1.257 | 1.220 | 1.343 |
| gi_31341725_ref_NM_174538.2_ | 282227 | FCGR1A | Fc fragment of IgG, high affinity Ia, receptor (CD64) | Plasma Membrane | transmembrane receptor | H | -15.082 | 0.000 | 8.087 | 0.000 | 14.337 | 0.000 |
| gi_157427855_ref_NM_001105365.1_ | 514031 | ITGB7 | integrin, beta 7 | Plasma Membrane | transmembrane receptor | H | -15.582 | 0.000 | 6.285 | 0.000 | 26.661 | 0.000 |
| gi_84370178_ref_NM_001038568.1_ | 540044 | GPR84 | G protein-coupled receptor 84 | Plasma Membrane | G-protein coupled recept | H | -17.628 | 0.000 | 10.342 | 0.000 | 20.487 | 0.000 |
| gi_119901378_ref_XM_611161.3_ | 538797 | EPHA7 | EPH receptor A7 | Plasma Membrane | kinase | H | -18.744 | 0.000 | 1.512 | 0.525 | 1.697 | 0.393 |
| gi_119914848_ref_XM_584158.3_ | 539002 | CCR2 | chemokine (C-C motif) receptor 2 | Plasma Membrane | G-protein coupled recept | H | -19.856 | 0.000 | 1.142 | 1.233 | 1.064 | 1.236 |
| gi_119915290_ref_XM_864510.2_ | 514819 | PTK7 | PTK7 protein tyrosine kinase 7 | Plasma Membrane | kinase | H | -22.433 | 0.000 | 1.708 | 0.205 | 6.498 | 0.000 |
| gi_119903128_ref_XM_580552.3_ | 504429 | MERTK | c-mer proto-oncogene tyrosine kinase | Plasma Membrane | kinase | VH | -24.859 | 0.000 | 1.335 | 1.057 | 1.143 | 1.276 |
| gi_156120860_ref_NM_001102107.1_ | 527140 | CALCRL | calcitonin receptor-like | Plasma Membrane | G-protein coupled recept | H | -36.115 | 0.000 | 1.848 | 0.135 | 2.121 | 0.084 |
| gi_31342075_ref_NM_174410.2_ | 281963 | P2RY1 | purinergic receptor P2Y, G-protein coupled, 1 | Plasma Membrane | G-protein coupled recept | H | -37.328 | 0.000 | 3.886 | 0.000 | 3.873 | 0.001 |
| gi_114052616_ref_NM_001046504.1_ | 574090 | TLR1 | toll-like receptor 1 | Plasma Membrane | transmembrane receptor | VH | -38.763 | 0.000 | 4.670 | 0.000 | 21.531 | 0.000 |
| gi_76638859_ref_XM_582510.2_ | 538759 | GPR81 | G protein-coupled receptor 81 | Plasma Membrane | G-protein coupled recept | H | -42.492 | 0.000 | -1.064 | 0.732 | -1.008 | 0.896 |
| gi_119893274_ref_XM_870848.2_ | 618516 | TNFRSF13C | tumor necrosis factor receptor superfamily, member 13 | Plasma Membrane | transmembrane receptor | VH | -49.962 | 0.000 | -1.017 | 0.953 | 1.979 | 0.161 |
| gi_119912116_ref_XM_584605.3_ | 353110 | NGFR | nerve growth factor receptor (TNFR superfamily, meml | Plasma Membrane | transmembrane receptor | VH | -50.012 | 0.000 | -1.285 | 0.254 | -1.112 | 0.644 |
| gi_89886135_ref_NM_001013585.3_ | 281135 | S1PR1 | sphingosine-1-phosphate receptor 1 | Plasma Membrane | G-protein coupled recept | VH | -58.987 | 0.000 | 4.088 | 0.000 | 13.674 | 0.000 |
| gi_61825106_ref_XM_583123.1_ | 538837 | GPR141 | G protein-coupled receptor 141 | Plasma Membrane | G-protein coupled recept | H | -59.180 | 0.000 | 1.317 | 1.100 | 2.078 | 0.112 |
| gi_126723186_ref_NM_174198.6_ | 281536 | TLR4 | toll-like receptor 4 | Plasma Membrane | transmembrane receptor | VH | -70.557 | 0.000 | 4.771 | 0.000 | 4.846 | 0.000 |
| gi_99028960_ref_NM_174184.3_ | 281489 | SLAMF1 | signaling lymphocytic activation molecule family memb | Plasma Membrane | transmembrane receptor | VH | -84.352 | 0.000 | 1.301 | 1.106 | 1.326 | 1.182 |
| gi_157427947_ref_NM_001105411.1_ | 534801 | GFRA1 | GDNF family receptor alpha 1 | Plasma Membrane | transmembrane receptor | VH | -99.175 | 0.000 | 1.884 | 0.106 | 4.459 | 0.000 |
| gi_119913552_ref_XM_606794.3_ | 281848 | IGF1R | insulin-like growth factor 1 receptor | Plasma Membrane | transmembrane receptor | VH | -107.774 | 0.000 | 1.943 | 0.061 | 1.866 | 0.190 |
| gi_76660614_ref_XM_597941.2_ | 519716 | CCR6 | chemokine (C-C motif) receptor 6 | Plasma Membrane | G-protein coupled recept | H | -110.621 | 0.000 | 1.437 | 0.755 | 2.660 | 0.014 |
| gi_153792426_ref_NM_001099726.1_ | 782045 | CYSLTR1 | cysteinyl leukotriene receptor 1 | Plasma Membrane | G-protein coupled recept | VH | -162.124 | 0.000 | 1.562 | 0.439 | 12.481 | 0.000 |
| gi_31343049_ref_NM_174010.2_ | 281052 | CD36 | CD36 molecule (thrombospondin receptor) | Plasma Membrane | transmembrane receptor | VH | -185.369 | 0.000 | 2.016 | 0.051 | 2.040 | 0.123 |
| gi_119911249_ref_XM_867060.2_ | 615295 | LAIR1 | leukocyte-associated immunoglobulin-like receptor 1 | Plasma Membrane | transmembrane receptor | VH | -230.065 | 0.000 | 1.227 | 1.175 | 1.082 | 1.141 |

## Table S6C: Cell Surface - ion channels and transporters

| SEQ_ID | Entrez gene ID | Symbol | Entrez Gene Name | Location | Type | Predicted BL20 expression level | FC BLvTBL | FDR BLvsTBL | FC TBLvTBL24h | FDR TBLvTBL24h | FC TBLvTBL48h | FDR TBLvTBL48h |
|---|---|---|---|---|---|---|---|---|---|---|---|---|
| gi_119894775_ref_XM_868927.2_ | 281276 | LDLR | low density lipoprotein receptor | Plasma Membrane | transporter | L | 117.922 | 0.000 | -1.400 | 0.095 | -1.191 | 0.528 |
| gi_76655242_ref_XM_582736.2_ | 538791 | KCNK18 | potassium channel, subfamily K, member 18 | Plasma Membrane | ion channel | N | 59.961 | 0.000 | -2.067 | 0.000 | -3.715 | 0.000 |
| gi_31341539_ref_NM_174610.2_ | 282366 | SLC6A6 | solute carrier family 6 (neurotransmitter transporter, ta | Plasma Membrane | transporter | M | 45.455 | 0.000 | -2.500 | 0.000 | -2.570 | 0.000 |
| gi_134085654_ref_NM_001083448.1_ | 527014 | SV2B | synaptic vesicle glycoprotein 2B | Plasma Membrane | transporter | L | 33.466 | 0.000 | -1.134 | 0.658 | -3.784 | 0.000 |
| gi_119911706_ref_XM_594012.3_ | 338035 | P2RX5 | purinergic receptor P2X, ligand-gated ion channel, 5 | Plasma Membrane | ion channel | M | 24.146 | 0.000 | -1.444 | 0.056 | -1.855 | 0.002 |
| gi_119892068_ref_XM_868185.2_ | 537277 | SCN8A | sodium channel, voltage gated, type VIII, alpha subuni | Plasma Membrane | ion channel | L | 16.099 | 0.000 | 2.166 | 0.027 | -1.071 | 0.916 |
| gi_125630722_ref_NM_001081543.1_ | 615761 | KCNE4 | potassium voltage-gated channel, Isk-related family, m | Plasma Membrane | ion channel | L | 11.016 | 0.000 | 2.399 | 0.010 | 2.891 | 0.007 |
| gi_47564023_ref_NM_001001134.1_ | 407132 | SLCO3A1 | solute carrier organic anion transporter family, membe | Plasma Membrane | transporter | L | 9.861 | 0.000 | -1.500 | 0.034 | -2.761 | 0.000 |
| gi_119887104_ref_XM_587930.3_ | 510745 | ABCG1 | ATP-binding cassette, sub-family G (WHITE), member | Plasma Membrane | transporter | M | 7.545 | 0.000 | -6.325 | 0.000 | -4.659 | 0.000 |
| gi_31341556_ref_NM_174602.2_ | 282356 | SLC2A1 | solute carrier family 2 (facilitated glucose transporter), | Plasma Membrane | transporter | H | 7.347 | 0.000 | -2.188 | 0.000 | -2.633 | 0.000 |
| gi_150247069_ref_NM_001099378.1_ | 521181 | SLC15A1 | solute carrier family 15 (oligopeptide transporter), mem | Plasma Membrane | transporter | L | 5.429 | 0.000 | -1.654 | 0.008 | -3.253 | 0.000 |
| gi_148234191_ref_NM_001098036.1_ | 515437 | SLC39A14 | solute carrier family 39 (zinc transporter), member 14 | Plasma Membrane | transporter | H | 5.231 | 0.000 | -2.133 | 0.000 | -2.895 | 0.000 |
| gi_119907150_ref_XM_612483.3_ | 533166 | SORL1 | sortilin-related receptor, L(DLR class) A repeats-contai | Plasma Membrane | transporter | L | 4.761 | 0.000 | 5.674 | 0.000 | 8.918 | 0.000 |
| gi_31341534_ref_NM_174613.2_ | 282369 | SLC7A5 | solute carrier family 7 (cationic amino acid transporter, | Plasma Membrane | transporter | H | 4.114 | 0.000 | -4.668 | 0.000 | -4.840 | 0.000 |
| gi_158341669_ref_NM_001109980.1_ | 510085 | SLC16A3 | solute carrier family 16, member 3 (monocarboxylic ac | Plasma Membrane | transporter | L | 3.566 | 0.000 | -1.188 | 0.370 | -1.008 | 0.857 |
| gi_119893596_ref_XM_584935.3_ | 508193 | SLC39A8 | solute carrier family 39 (zinc transporter), member 8 | Extracellular Space | transporter | L | 3.557 | 0.000 | 1.351 | 0.976 | -1.767 | 0.005 |
| gi_115495866_ref_NM_001076246.1_ | 540853 | SNAP25 | synaptosomal-associated protein, 25kDa | Plasma Membrane | transporter | N | 3.536 | 0.000 | -1.141 | 0.412 | -1.069 | 0.776 |
| gi_119889235_ref_XM_868686.2_ | 534180 | KCNN3 | potassium intermediate/small conductance calcium-ac | Plasma Membrane | ion channel | M | 3.158 | 0.000 | 1.006 | 1.003 | 1.481 | 0.874 |
| gi_119890545_ref_XM_614767.3_ | 541151 | TRPM8 | transient receptor potential cation channel, subfamily M | Plasma Membrane | ion channel | L | 3.149 | 0.000 | 2.133 | 0.032 | 1.306 | 1.202 |
| gi_119889676_ref_XM_603860.3_ | 525507 | KCNA3 | potassium voltage-gated channel, shaker-related subfa | Plasma Membrane | ion channel | L | 3.090 | 0.000 | 2.308 | 0.014 | -1.403 | 0.084 |
| gi_119924879_ref_XM_590317.3_ | 281585 | ABCB1 | ATP-binding cassette, sub-family B (MDR/TAP), memb | Plasma Membrane | transporter | L | 2.858 | 0.000 | 1.133 | 0.956 | 1.072 | 0.886 |
| gi_136255756_ref_NM_001075630.2_ | 513621 | VAMP1 | vesicle-associated membrane protein 1 (synaptobrevin | Plasma Membrane | transporter | M | 2.340 | 0.000 | -1.019 | 0.988 | -1.227 | 0.379 |
| gi_31341239_ref_NM_174833.2_ | 317654 | SLC9A1 | solute carrier family 9 (sodium/hydrogen exchanger), r | Plasma Membrane | ion channel | H | 1.907 | 0.001 | -1.404 | 0.093 | -1.049 | 0.984 |
| gi_27807092_ref_NM_174605.1_ | 282360 | SLC4A4 | solute carrier family 4, sodium bicarbonate cotransport | Plasma Membrane | transporter | H | 1.889 | 0.001 | 1.465 | 0.740 | 4.175 | 0.000 |

| | | | | | | | | | | | |
|---|---|---|---|---|---|---|---|---|---|---|---|
| gi_88319926_ref_NM_174068.2_ | 281193 | GJA1 | gap junction protein, alpha 1, 43kDa | Plasma Membrane | transporter | H | 1.858 | 0.002 | -1.270 | 0.272 | -2.729 | 0.000 |
| gi_119912484_ref_XM_587873.3_ | 510698 | VAT1 | vesicle amine transport protein 1 homolog (T. californic | Plasma Membrane | transporter | H | 1.835 | 0.001 | 1.983 | 0.078 | 2.075 | 0.114 |
| gi_125991751_ref_NM_174603.3_ | 282358 | SLC2A3 | solute carrier family 2 (facilitated glucose transporter), | Plasma Membrane | transporter | H | 1.823 | 0.002 | -1.626 | 0.009 | -2.090 | 0.000 |
| gi_149643066_ref_NM_001099123.1_ | 613446 | PLLP | plasma membrane proteolipid (plasmolipin) | Plasma Membrane | transporter | L | 1.810 | 0.002 | 3.740 | 0.000 | 2.780 | 0.009 |
| gi_119910207_ref_XM_865652.2_ | 533876 | SLC12A4 | solute carrier family 12 (potassium/chloride transporter | Plasma Membrane | transporter | H | 1.807 | 0.002 | -1.546 | 0.023 | -1.244 | 0.364 |
| gi_56699419_ref_NM_174607.1_ | 282362 | SLC5A3 | solute carrier family 5 (sodium/myo-inositol cotranspor | Plasma Membrane | transporter | M | 1.762 | 0.002 | -1.440 | 0.054 | -1.575 | 0.022 |
| gi_119912899_ref_XM_587636.3_ | 510497 | ABCA5 | ATP-binding cassette, sub-family A (ABC1), member 5 | Plasma Membrane | transporter | H | 1.749 | 0.003 | -1.022 | 0.974 | -1.224 | 0.423 |
| gi_31341563_ref_NM_174601.2_ | 282355 | SLC1A5 | solute carrier family 1 (neutral amino acid transporter), | Plasma Membrane | transporter | VH | 1.746 | 0.002 | -1.519 | 0.027 | -1.697 | 0.008 |
| gi_62177171_ref_NM_001014384.1_ | 541597 | KCNAB2 | potassium voltage-gated channel, shaker-related subfa | Plasma Membrane | ion channel | H | 1.739 | 0.002 | -1.292 | 0.239 | 1.075 | 1.274 |
| gi_122692594_ref_NM_001080288.1_ | 526291 | SLC13A3 | solute carrier family 13 (sodium-dependent dicarboxyla | Plasma Membrane | transporter | L | 1.703 | 0.005 | -1.037 | 0.830 | -1.241 | 0.227 |
| gi_119924210_ref_XM_001249496.1_ | 781113 | SLC5A3 | solute carrier family 5 (sodium/myo-inositol cotranspor | Plasma Membrane | transporter | M | 1.681 | 0.004 | -1.984 | 0.000 | -2.597 | 0.000 |
| gi_154152194_ref_NM_001100381.1_ | 518976 | SLC31A1 | solute carrier family 31 (copper transporters), member | Plasma Membrane | transporter | VH | 1.638 | 0.005 | -1.380 | 0.100 | -2.126 | 0.000 |
| gi_164448599_ref_NM_001024488.2_ | 507107 | SLC3A2 | solute carrier family 3 (activators of dibasic and neutra | Plasma Membrane | transporter | H | 1.594 | 0.007 | -1.861 | 0.001 | -1.686 | 0.008 |
| gi_115497811_ref_NM_001076453.1_ | 617307 | SLC19A1 | solute carrier family 19 (folate transporter), member 1 | Plasma Membrane | transporter | VH | 1.579 | 0.007 | -1.531 | 0.022 | -1.670 | 0.009 |
| gi_155371936_ref_NM_001101103.1_ | 521189 | SLC11A2 | solute carrier family 11 (proton-coupled divalent metal | Plasma Membrane | transporter | H | 1.565 | 0.008 | 1.168 | 1.252 | 1.050 | 1.226 |
| gi_119908342_ref_XM_598445.3_ | 520210 | CLCN6 | chloride channel 6 | Plasma Membrane | ion channel | H | 1.552 | 0.009 | -1.536 | 0.028 | 1.006 | 1.133 |
| gi_122692298_ref_NM_001080223.1_ | 504503 | SLC29A3 | solute carrier family 29 (nucleoside transporters), mem | Plasma Membrane | transporter | M | 1.544 | 0.009 | 1.059 | 1.183 | 1.323 | 1.241 |
| gi_119908509_ref_XM_607245.3_ | 528813 | SCNN1D | sodium channel, nonvoltage-gated 1, delta | Plasma Membrane | ion channel | L | 1.522 | 0.016 | -1.335 | 0.143 | -1.332 | 0.168 |
| gi_157954054_ref_NM_001109793.1_ | 511800 | ANKH | ankylosis, progressive homolog (mouse) | Plasma Membrane | transporter | VH | 1.515 | 0.010 | 1.701 | 0.297 | 1.562 | 0.705 |
| gi_119894467_ref_XM_614120.3_ | 534369 | ATP13A1 | ATPase type 13A1 | Extracellular Space | transporter | VH | 1.512 | 0.011 | -1.620 | 0.011 | -1.733 | 0.006 |
| gi_31341773_ref_NM_174519.2_ | 282184 | CLDN16 | claudin 16 | Plasma Membrane | transporter | L | 1.496 | 0.017 | 3.504 | 0.000 | 3.568 | 0.001 |
| gi_115497975_ref_NM_001075207.1_ | 505563 | KCNK1 | potassium channel, subfamily K, member 1 | Plasma Membrane | ion channel | H | 1.478 | 0.015 | -1.767 | 0.003 | -2.348 | 0.000 |
| gi_126158902_ref_NM_001081577.1_ | 326577 | SLC1A4 | solute carrier family 1 (glutamate/neutral amino acid tr | Plasma Membrane | transporter | VH | 1.453 | 0.019 | -1.955 | 0.000 | -1.932 | 0.001 |
| gi_122692468_ref_NM_001080300.1_ | 534568 | KCNMB2 | potassium large conductance calcium-activated chann | Plasma Membrane | ion channel | L | 1.423 | 0.025 | 4.022 | 0.000 | 2.952 | 0.006 |
| gi_31340942_ref_NM_174782.2_ | 286845 | SLC12A2 | solute carrier family 12 (sodium/potassium/chloride tra | Plasma Membrane | transporter | H | 1.413 | 0.026 | -1.117 | 0.756 | -1.713 | 0.007 |
| gi_84370157_ref_NM_001038563.1_ | 538144 | TMED1 | transmembrane emp24 protein transport domain conta | Extracellular Space | transporter | H | 1.407 | 0.026 | -1.419 | 0.077 | -1.411 | 0.092 |
| gi_148224853_ref_NM_001076237.3_ | 540716 | SLC39A7 | solute carrier family 39 (zinc transporter), member 7 | Plasma Membrane | transporter | VH | 1.406 | 0.026 | -1.474 | 0.045 | -1.560 | 0.025 |
| gi_119905633_ref_XM_611249.3_ | 507969 | SLCO4A1 | solute carrier organic anion transporter family, member | Plasma Membrane | transporter | H | 1.394 | 0.030 | -1.841 | 0.001 | -2.532 | 0.000 |
| gi_119905474_ref_XM_001251156.1_ | 783536 | SLC23A2 | solute carrier family 23 (nucleobase transporters), mer | Plasma Membrane | transporter | H | 1.375 | 0.035 | 1.067 | 1.068 | 1.055 | 1.215 |
| gi_78369389_ref_NM_001035381.1_ | 530352 | SLC39A1 | solute carrier family 39 (zinc transporter), member 1 | Plasma Membrane | transporter | H | 1.373 | 0.037 | -1.137 | 0.695 | -1.097 | 0.854 |
| gi_156121042_ref_NM_001102198.1_ | 537280 | SLC19A3 | solute carrier family 19, member 3 | Plasma Membrane | transporter | H | 1.369 | 0.037 | 1.591 | 0.477 | 1.275 | 1.331 |
| gi_139949143_ref_NM_001083743.1_ | 539478 | TM9SF1 | transmembrane 9 superfamily member 1 | Plasma Membrane | transporter | VH | 1.357 | 0.042 | 1.075 | 1.180 | -1.085 | 0.858 |
| gi_161760672_ref_NM_001075937.2_ | 530661 | SLC7A6 | solute carrier family 7 (cationic amino acid transporter, | Plasma Membrane | transporter | VH | 1.332 | 0.045 | -1.465 | 0.036 | -1.457 | 0.065 |
| gi_116734842_ref_NM_001075129.1_ | 282648 | CACNA1A | calcium channel, voltage-dependent, P/Q type, alpha ' | Plasma Membrane | ion channel | L | -2.985 | 0.049 | -1.128 | 0.612 | 1.231 | 1.329 |
| gi_119894372_ref_XR_027411.1_ | 618257 | SORCS2 | sortilin-related VPS10 domain containing receptor 2 | Plasma Membrane | transporter | L | -3.038 | 0.049 | 1.809 | 0.165 | 2.189 | 0.070 |
| gi_114052125_ref_NM_001046116.1_ | 512311 | SLC5A7 | solute carrier family 5 (choline transporter), member 7 | Plasma Membrane | transporter | M | -3.057 | 0.042 | 1.118 | 1.174 | 1.186 | 1.323 |
| gi_155371972_ref_NM_001101123.1_ | 525480 | SLC16A4 | solute carrier family 16, member 4 (monocarboxylic ac | Plasma Membrane | transporter | L | -3.068 | 0.042 | 1.475 | 0.636 | 1.497 | 0.812 |
| gi_119904862_ref_XM_593336.3_ | 515333 | ABCC4 | ATP-binding cassette, sub-family C (CFTR/MRP), mer | Plasma Membrane | transporter | VH | -3.073 | 0.037 | -1.059 | 0.960 | -1.056 | 0.984 |
| gi_119902660_ref_XM_610426.3_ | 531921 | SLC28A2 | solute carrier family 28 (sodium-coupled nucleoside tra | Plasma Membrane | transporter | L | -3.109 | 0.042 | 1.123 | 1.146 | 1.504 | 0.814 |
| gi_119906046_ref_XM_599598.3_ | 521338 | KCNS1 | potassium voltage-gated channel, delayed-rectifier, su | Plasma Membrane | ion channel | L | -3.185 | 0.034 | -1.090 | 0.653 | -1.067 | 0.778 |
| gi_114050716_ref_NM_001046476.1_ | 540406 | VAMP5 | vesicle-associated membrane protein 5 (myobrevin) | Plasma Membrane | transporter | H | -3.205 | 0.029 | 1.363 | 0.965 | 1.404 | 1.079 |
| gi_76667956_ref_XM_603455.2_ | 525109 | KCNK10 | potassium channel, subfamily K, member 10 | Plasma Membrane | ion channel | H | -3.278 | 0.023 | 1.642 | 0.379 | 1.237 | 1.343 |
| gi_157785612_ref_NM_001105628.1_ | 536495 | ATP10D | ATPase, class V, type 10D | Plasma Membrane | transporter | H | -3.282 | 0.023 | 2.266 | 0.017 | 3.647 | 0.001 |
| gi_155722980_ref_NM_001101042.1_ | 282868 | SLC2A5 | solute carrier family 2 (facilitated glucose/fructose tran: | Plasma Membrane | transporter | L | -3.312 | 0.024 | 48.731 | 0.000 | 38.989 | 0.000 |
| gi_119906802_ref_XM_869405.2_ | 617196 | SLC26A7 | solute carrier family 26, member 7 | Plasma Membrane | transporter | M | -3.360 | 0.021 | 1.467 | 0.684 | -1.146 | 0.458 |
| gi_139948256_ref_NM_001083777.1_ | 617017 | RAMP1 | receptor (G protein-coupled) activity modifying protein | Plasma Membrane | transporter | H | -3.388 | 0.021 | -1.297 | 0.206 | -1.385 | 0.116 |
| gi_119919426_ref_XM_001252337.1_ | 784876 | KCNQ1 | potassium voltage-gated channel, KQT-like subfamily, | Plasma Membrane | ion channel | M | -3.445 | 0.017 | 1.260 | 1.165 | 1.195 | 1.244 |
| gi_122692576_ref_NM_001080280.1_ | 518905 | SLC20A2 | solute carrier family 20 (phosphate transporter), memb | Plasma Membrane | transporter | H | -3.469 | 0.016 | 4.643 | 0.000 | 12.140 | 0.000 |
| gi_114050956_ref_NM_001046076.1_ | 511119 | GJC1 | gap junction protein, gamma 1, 45kDa | Plasma Membrane | transporter | VH | -3.578 | 0.011 | 1.236 | 1.255 | 1.023 | 1.185 |
| gi_119901333_ref_XM_866973.2_ | 615226 | GRIK2 | glutamate receptor, ionotropic, kainate 2 | Plasma Membrane | ion channel | M | -3.609 | 0.010 | -1.358 | 0.112 | 1.173 | 1.273 |
| gi_125991871_ref_NM_001081596.1_ | 529939 | LAMB3 | laminin, beta 3 | Extracellular Space | transporter | M | -3.835 | 0.007 | -1.201 | 0.432 | 1.010 | 1.140 |
| gi_76638883_ref_XM_591410.2_ | 286814 | P2RX7 | purinergic receptor P2X, ligand-gated ion channel, 7 | Plasma Membrane | ion channel | M | -3.860 | 0.007 | 1.728 | 0.232 | 2.158 | 0.079 |
| gi_119912813_ref_XM_610029.3_ | 531531 | SLC16A5 | solute carrier family 16, member 5 (monocarboxylic ac | Plasma Membrane | transporter | M | -3.988 | 0.006 | -1.073 | 0.541 | 1.125 | 1.262 |
| gi_99028972_ref_NM_174195.3_ | 281518 | TCN2 | transcobalamin II; macrocytic anemia | Extracellular Space | transporter | H | -4.054 | 0.004 | 2.883 | 0.001 | 4.362 | 0.000 |
| gi_119909528_ref_XM_599391.3_ | 521133 | SEC14L3 | SEC14-like 3 (S. cerevisiae) | Extracellular Space | transporter | M | -4.078 | 0.005 | 1.423 | 0.707 | 1.688 | 0.444 |
| gi_66792907_ref_NM_001024563.1_ | 535990 | KCNB2 | potassium voltage-gated channel, Shab-related subfar | Plasma Membrane | ion channel | M | -4.108 | 0.005 | -1.010 | 0.914 | -1.045 | 0.813 |
| gi_31341853_ref_NM_174489.2_ | 282123 | VLDLR | very low density lipoprotein receptor | Plasma Membrane | transporter | M | -4.127 | 0.005 | 1.638 | 0.341 | 3.007 | 0.005 |
| gi_119892308_ref_XM_598691.2_ | 520448 | KCNMB4 | potassium large conductance calcium-activated chann | Plasma Membrane | ion channel | H | -4.207 | 0.004 | 1.762 | 0.214 | -1.022 | 1.056 |
| gi_119911727_ref_XM_864597.2_ | 507000 | SLC13A5 | solute carrier family 13 (sodium-dependent citrate tran | Plasma Membrane | transporter | H | -4.506 | 0.002 | 2.710 | 0.002 | 3.208 | 0.003 |
| gi_119919062_ref_XM_585917.3_ | 539250 | KCNJ1 | potassium inwardly-rectifying channel, subfamily J, me | Plasma Membrane | ion channel | M | -4.758 | 0.002 | -1.175 | 0.479 | 1.071 | 1.224 |
| gi_77735752_ref_NM_001034399.1_ | 511064 | FXYD6 | FXYD domain containing ion transport regulator 6 | Plasma Membrane | ion channel | M | -4.893 | 0.001 | 4.279 | 0.000 | 4.377 | 0.000 |
| gi_119910843_ref_XM_001253416.1_ | 785362 | SLC6A16 | solute carrier family 6, member 16 | Plasma Membrane | transporter | M | -5.131 | 0.001 | 1.033 | 1.097 | 1.441 | 0.940 |
| gi_66792889_ref_NM_001024553.1_ | 518384 | KCND1 | potassium voltage-gated channel, Shal-related subfam | Plasma Membrane | ion channel | H | -5.194 | 0.001 | 1.463 | 0.748 | 5.931 | 0.000 |
| gi_119894510_ref_XM_868291.2_ | 408008 | KCNN1 | potassium intermediate/small conductance calcium-ac | Plasma Membrane | ion channel | M | -5.298 | 0.001 | 1.189 | 1.178 | 1.272 | 1.303 |
| gi_148223920_ref_NM_001076336.2_ | 614573 | SLC16A7 | solute carrier family 16, member 7 (monocarboxylic ac | Plasma Membrane | transporter | VH | -5.727 | 0.000 | 2.714 | 0.002 | 2.591 | 0.016 |
| gi_77404276_ref_NM_001034049.1_ | 338036 | P2RX4 | purinergic receptor P2X, ligand-gated ion channel, 4 | Plasma Membrane | ion channel | H | -5.759 | 0.000 | 1.983 | 0.071 | 2.748 | 0.010 |

| | | | | | | | | | | | | |
|---|---|---|---|---|---|---|---|---|---|---|---|---|
| gi_119931673_ref_XM_001249309.1_ | 781005 | SERINC2 | serine incorporator 2 | Plasma Membrane | transporter | H | -5.825 | 0.000 | 5.902 | 0.000 | 11.210 | 0.000 |
| gi_70778797_ref_NM_001025333.1_ | 516626 | OSTALPHA | organic solute transporter alpha | Plasma Membrane | transporter | M | -5.879 | 0.000 | 1.471 | 0.655 | 1.075 | 1.279 |
| gi_119918891_ref_XM_606459.3_ | 528050 | SLC6A5 | solute carrier family 6 (neurotransmitter transporter, gly | Plasma Membrane | transporter | H | -6.032 | 0.000 | 1.300 | 1.189 | -1.107 | 0.748 |
| gi_78370166_ref_NM_001035285.1_ | 513593 | SERINC2 | serine incorporator 2 | Plasma Membrane | transporter | H | -6.116 | 0.000 | 5.071 | 0.000 | 9.998 | 0.000 |
| gi_119912178_ref_XM_612461.3_ | 533151 | ABCC3 | ATP-binding cassette, sub-family C (CFTR/MRP), mer | Plasma Membrane | transporter | VH | -6.476 | 0.000 | 1.872 | 0.122 | 4.046 | 0.000 |
| gi_112817614_ref_NM_001037478.2_ | 536203 | ABCG2 | ATP-binding cassette, sub-family G (WHITE), member | Plasma Membrane | transporter | VH | -7.117 | 0.000 | 2.645 | 0.004 | 2.579 | 0.018 |
| gi_119895570_ref_XM_581784.3_ | 505492 | SLC23A1 | solute carrier family 23 (nucleobase transporters), mer | Plasma Membrane | transporter | M | -7.174 | 0.000 | 1.382 | 0.974 | 1.739 | 0.387 |
| gi_115497519_ref_NM_001075151.1_ | 504220 | SLC7A7 | solute carrier family 7 (cationic amino acid transporter, | Plasma Membrane | transporter | VH | -7.348 | 0.000 | 3.765 | 0.000 | 5.496 | 0.000 |
| gi_115496685_ref_NM_001075325.1_ | 507672 | FOLR2 | folate receptor 2 (fetal) | Plasma Membrane | transporter | M | -7.688 | 0.000 | 1.676 | 0.245 | 5.408 | 0.000 |
| gi_119906935_ref_XM_586071.3_ | 407151 | TRPC6 | transient receptor potential cation channel, subfamily C | Plasma Membrane | ion channel | H | -9.004 | 0.000 | 1.394 | 0.917 | -1.792 | 0.004 |
| gi_119911701_ref_XM_863847.2_ | 539074 | TRPV1 | transient receptor potential cation channel, subfamily V | Plasma Membrane | ion channel | M | -10.925 | 0.000 | -1.100 | 0.425 | -1.102 | 0.367 |
| gi_119920329_ref_XM_586812.3_ | 509779 | CACNA1F | calcium channel, voltage-dependent, L type, alpha 1F | Plasma Membrane | ion channel | H | -11.252 | 0.000 | 1.733 | 0.232 | 2.573 | 0.019 |
| gi_115494983_ref_NM_001076301.1_ | 613972 | APOD | apolipoprotein D | Extracellular Space | transporter | H | -11.536 | 0.000 | 2.512 | 0.006 | 2.502 | 0.024 |
| gi_119331227_ref_NM_001079794.1_ | 780866 | AQP3 | aquaporin 3 (Gill blood group) | Plasma Membrane | transporter | VH | -12.681 | 0.000 | 8.980 | 0.000 | 12.438 | 0.000 |
| gi_119915752_ref_XM_584165.3_ | 507535 | SLC17A1 | solute carrier family 17 (sodium phosphate), member 1 | Plasma Membrane | transporter | M | -12.793 | 0.000 | 1.197 | 1.255 | 1.454 | 0.924 |
| gi_119912146_ref_XM_001252666.1_ | 282411 | CACNA1G | calcium channel, voltage-dependent, T type, alpha 1G | Plasma Membrane | ion channel | H | -16.162 | 0.000 | -1.340 | 0.097 | 1.016 | 0.827 |
| gi_56710326_ref_NM_001008666.1_ | 493988 | SLC14A1 | solute carrier family 14 (urea transporter), member 1 (I | Plasma Membrane | transporter | M | -17.271 | 0.000 | 1.230 | 1.257 | 1.169 | 1.323 |
| gi_118150847_ref_NM_001077867.1_ | 507185 | OSTBETA | organic solute transporter beta | Plasma Membrane | transporter | H | -18.206 | 0.000 | 1.927 | 0.092 | 5.650 | 0.000 |
| gi_31341522_ref_NM_174618.2_ | 282377 | STX1B | syntaxin 1B | Plasma Membrane | ion channel | H | -18.646 | 0.000 | 1.792 | 0.155 | 4.925 | 0.000 |
| gi_119911051_ref_XM_599883.3_ | 521616 | CACNG6 | calcium channel, voltage-dependent, gamma subunit 6 | Plasma Membrane | ion channel | H | -28.982 | 0.000 | 1.384 | 0.568 | 1.338 | 1.041 |
| gi_119918570_ref_XM_001252944.1_ | 784716 | CDH23 | cadherin-like 23 | Plasma Membrane | transporter | H | -28.982 | 0.000 | -1.263 | 0.281 | -1.120 | 0.651 |
| gi_119904117_ref_XM_605012.3_ | 526639 | LCN2 | lipocalin 2 | Extracellular Space | transporter | H | -30.326 | 0.000 | -1.055 | 0.804 | 1.446 | 0.887 |
| gi_32189337_ref_NM_174143.1_ | 281401 | PIGR | polymeric immunoglobulin receptor | Plasma Membrane | transporter | H | -85.226 | 0.000 | 2.400 | 0.010 | 3.736 | 0.001 |
| gi_157954060_ref_NM_001109795.1_ | 513856 | A2M | alpha-2-macroglobulin | Extracellular Space | transporter | VH | -109.925 | 0.000 | 2.001 | 0.059 | 4.697 | 0.000 |
| gi_31341436_ref_NM_174652.2_ | 282470 | SLC11A1 | solute carrier family 11 (proton-coupled divalent metal | Plasma Membrane | transporter | VH | -238.083 | 0.000 | 2.273 | 0.015 | 8.234 | 0.000 |
