# Supplementary material for: A Bovine Lymphosarcoma Cell Line Infected with Theileria annulata Exhibits an Irreversible Reconfiguration of Host Cell Gene Expression
Source: PLoS One. 2013 Jun 26;8(6):e66833. doi: 10.1371/journal.pone.0066833 (PMC3694138; doi:10.1371/journal.pone.0066833)
Supplement: Table S7 — (PDF) [file pone.0066833.s009.pdf]

Table S7A: Transcription

SEQ_ID	Entrez gene ID	Symbol	Entrez Gene Name	Location	Type	Predicted BL20 expression level	FC BLvTBL	FDR BLvsTBL	FC TBLvTBL24h	FDR TBLvTBL24h	FC TBLvTBL48h	FDR TBLvTBL48h
gi_114053110_ref_NM_001046252.1_	520564	LITAF	lipopolysaccharide-induced TNF factor	Nucleus	transcription regulator	L	116.161	0.000	1.021	1.055	-1.231	0.391
gi_119927959_ref_XM_593064.3_	515105	TP73	tumor protein p73	Nucleus	transcription regulator	L	98.669	0.000	-1.451	0.049	-1.169	0.512
gi_119910732_ref_XM_869544.2_	617311	MEIS3	Meis homeobox 3	Nucleus	transcription regulator	M	65.152	0.000	-1.973	0.000	-2.028	0.000
gi_62460599_ref_NM_001014950.1_	538690	ID3	inhibitor of DNA binding 3, dominant negative helix-loo	Nucleus	transcription regulator	L	58.057	0.000	1.392	0.811	1.849	0.251
gi_119919278_ref_XM_609882.3_	531389	FOSL1	FOS-like antigen 1	Nucleus	transcription regulator	L	37.291	0.000	-3.872	0.000	-5.028	0.000
gi_134085833_ref_NM_001083490.1_	538716	HOXC13	homeobox C13	Nucleus	transcription regulator	L	15.968	0.000	2.058	0.046	2.149	0.084
gi_78369225_ref_NM_001035273.1_	508156	SSBP4	single stranded DNA binding protein 4	Nucleus	transcription regulator	M	14.333	0.000	-1.660	0.009	-1.000	1.092
gi_157168340_ref_NM_001103253.1_	517192	JUND	jun D proto-oncogene	Nucleus	transcription regulator	M	12.326	0.000	-2.471	0.000	-1.479	0.054
gi_119903790_ref_XM_864138.2_	509889	FOSL2	FOS-like antigen 2	Nucleus	transcription regulator	H	12.227	0.000	-1.035	0.922	1.401	1.087
gi_156120848_ref_NM_001102101.1_	526392	NFKB2	nuclear factor of kappa light polypeptide gene enhance	Nucleus	transcription regulator	H	9.354	0.000	-2.838	0.000	-1.390	0.123
gi_119888839_ref_XM_869638.2_	617389	RUNX3	runt-related transcription factor 3	Nucleus	transcription regulator	H	8.600	0.000	-1.414	0.084	1.562	0.689
gi_95147677_ref_NM_001033119.2_	503622	FOXA3	forkhead box A3	Nucleus	transcription regulator	L	6.348	0.000	-1.269	0.300	-1.576	0.021
gi_115495012_ref_NM_001075819.1_	521378	CITED2	Cbp/p300-interacting transactivator, with Glu/Asp-rich c	Nucleus	transcription regulator	H	5.909	0.000	-2.431	0.000	-2.881	0.000
gi_119912123_ref_XR_028313.1_	785855	HOXB8	homeobox B8	Nucleus	transcription regulator	L	5.121	0.000	2.258	0.018	1.788	0.318
gi_119913920_ref_XM_592227.3_	540196	PAX9	paired box 9	Nucleus	transcription regulator	L	5.083	0.000	1.662	0.305	1.309	1.221
gi_123959745_ref_NM_001080726.1_	504742	CITED4	Cbp/p300-interacting transactivator, with Glu/Asp-rich c	Nucleus	transcription regulator	M	4.724	0.000	-1.607	0.012	-1.245	0.321
gi_167583505_ref_NM_001114515.1_	614673	NUPR1	nuclear protein, transcriptional regulator, 1	Nucleus	transcription regulator	M	4.187	0.000	2.472	0.007	7.045	0.000
gi_119901328_ref_XM_618588.3_	538384	PRDM1	PR domain containing 1, with ZNF domain	Nucleus	transcription regulator	H	4.186	0.000	-1.006	1.041	1.355	1.166
gi_119901924_ref_XM_001251809.1_	515125	SMAD3	SMAD family member 3	Nucleus	transcription regulator	H	4.105	0.000	1.177	1.249	1.416	1.060
gi_119908089_ref_XM_586687.3_	539364	BTG2	BTG family, member 2	Nucleus	transcription regulator	M	4.041	0.000	-1.428	0.057	-1.093	0.799
gi_156120726_ref_NM_001102040.1_	516952	SATB1	SATB homeobox 1	Nucleus	transcription regulator	M	4.014	0.000	-1.226	0.377	-1.082	0.818
gi_110347584_ref_NM_182786.2_	280795	FOS	FBJ murine osteosarcoma viral oncogene homolog	Nucleus	transcription regulator	H	3.966	0.000	-1.263	0.279	-1.537	0.029
gi_119915104_ref_XM_591149.3_	513466	TEAD3	TEA domain family member 3	Nucleus	transcription regulator	M	3.941	0.000	-2.533	0.000	-2.676	0.000
gi_67972412_ref_NM_001024929.1_	506945	BHLHE40	basic helix-loop-helix family, member e40	Nucleus	transcription regulator	VH	3.779	0.000	1.098	1.217	1.019	1.188
gi_41386700_ref_NM_174726.1_	282713	NFKBIZ	nuclear factor of kappa light polypeptide gene enhance	Nucleus	transcription regulator	H	3.766	0.000	1.912	0.102	1.945	0.183
gi_119919224_ref_XM_615207.3_	541233	RCOR2	REST corepressor 2	Nucleus	transcription regulator	M	3.528	0.000	-1.122	0.673	2.139	0.086
gi_119910676_ref_XM_600955.3_	522670	RELB	v-rel reticuloendotheliosis viral oncogene homolog B	Nucleus	transcription regulator	M	3.422	0.000	-2.161	0.000	-1.074	0.923
gi_119900643_ref_XM_604596.3_	526233	EGR3	early growth response 3	Nucleus	transcription regulator	H	3.238	0.000	-3.012	0.000	-1.776	0.004
gi_119879716_ref_XM_001250887.1_	539020	BCL6	B-cell CLL/lymphoma 6	Nucleus	transcription regulator	L	3.229	0.000	-1.954	0.000	-1.165	0.541
gi_77735420_ref_NM_001034231.1_	505025	ID2	inhibitor of DNA binding 2, dominant negative helix-loo	Nucleus	transcription regulator	VH	2.939	0.000	1.192	1.252	1.082	1.282
gi_119891401_ref_XM_590833.3_	513185	PARP12	poly (ADP-ribose) polymerase family, member 12	Nucleus	other	M	2.811	0.000	5.184	0.000	12.238	0.000
gi_78365288_ref_NM_001035465.1_	615340	IRF5	interferon regulatory factor 5	Nucleus	transcription regulator	M	2.796	0.000	2.108	0.038	2.786	0.009
gi_94966918_ref_NM_001040564.1_	539321	SERTAD1	SERTA domain containing 1	Nucleus	transcription regulator	M	2.786	0.000	-1.795	0.002	-1.892	0.002
gi_157074115_ref_NM_001103300.1_	617914	MAFF	v-maf musculoaponeurotic fibrosarcoma oncogene hor	Nucleus	transcription regulator	M	2.672	0.000	-3.228	0.000	-2.321	0.000
gi_164519007_ref_NM_001113302.1_	539361	SREBF1	sterol regulatory element binding transcription factor 1	Nucleus	transcription regulator	H	2.595	0.000	-7.844	0.000	-2.673	0.000
gi_118601779_ref_NM_001079591.1_	513983	SCAND1	SCAN domain containing 1	Nucleus	transcription regulator	H	2.563	0.000	-1.869	0.000	-2.168	0.000
gi_115495324_ref_NM_001075935.1_	530610	ATF6	activating transcription factor 6	Cytoplasm	transcription regulator	VH	2.521	0.000	-1.551	0.020	-1.693	0.008
gi_119910608_ref_XM_610447.3_	531942	BCL3	B-cell CLL/lymphoma 3	Nucleus	transcription regulator	L	2.483	0.000	-1.149	0.391	2.639	0.014
gi_77736398_ref_NM_001034727.1_	541236	XBP1	X-box binding protein 1	Nucleus	transcription regulator	H	2.404	0.000	-2.298	0.000	-1.747	0.006
gi_77735604_ref_NM_001034326.1_	508638	NOC2L	nucleolar complex associated 2 homolog (S. cerevisiae	Nucleus	transcription regulator	VH	2.327	0.000	-1.498	0.032	-1.790	0.004
gi_70778751_ref_NM_001025344.1_	538951	NANOG	Nanog homeobox	Nucleus	transcription regulator	N	2.261	0.000	-2.393	0.000	-2.434	0.000
gi_60592797_ref_NM_001012673.1_	282375	STAT5A	signal transducer and activator of transcription 5A	Nucleus	transcription regulator	VH	2.254	0.000	-1.087	0.840	1.193	1.342
gi_125991883_ref_NM_001081579.1_	504991	ANKRD22	ankyrin repeat domain 22	Nucleus	transcription regulator	N	2.176	0.000	1.233	1.249	-1.005	0.797
gi_115497301_ref_NM_001076409.1_	616115	NFKB1	nuclear factor of kappa light polypeptide gene enhance	Nucleus	transcription regulator	VH	2.141	0.000	-1.398	0.093	-1.145	0.698
gi_119909893_ref_XM_606758.3_	528338	ZFPM1 (includes	zinc finger protein, multitype 1	Nucleus	transcription regulator	M	2.126	0.000	-1.704	0.006	-1.084	0.775
gi_119879695_ref_XM_867115.2_	615335	TP63	tumor protein p63	Nucleus	transcription regulator	L	2.073	0.001	2.399	0.010	11.094	0.000
gi_119916372_ref_XM_616030.3_	535916	SMAD7	SMAD family member 7	Nucleus	transcription regulator	H	2.067	0.000	1.297	1.200	2.153	0.082
gi_115497081_ref_NM_001075656.1_	514246	JUNB	jun B proto-oncogene	Nucleus	transcription regulator	H	2.028	0.001	-3.199	0.000	-2.049	0.000
gi_119893790_ref_XM_608827.3_	530358	KLF3	Kruppel-like factor 3 (basic)	Nucleus	transcription regulator	H	2.005	0.001	1.040	1.146	1.263	1.337
gi_154152114_ref_NM_001100336.1_	510923	TRIM25	tripartite motif-containing 25	Cytoplasm	transcription regulator	VH	1.957	0.001	-1.054	0.953	1.074	1.262
gi_119911776_ref_XM_590665.3_	513045	MYBBP1A	MYB binding protein (P160) 1a	Nucleus	transcription regulator	VH	1.927	0.001	-2.545	0.000	-2.738	0.000
gi_106880502_ref_NM_001034710.2_	540605	CREM	cAMP responsive element modulator	Nucleus	transcription regulator	H	1.919	0.001	-1.359	0.144	-1.403	0.103
gi_119904107_ref_XM_599033.3_	493639	PTGES2	prostaglandin E synthase 2	Cytoplasm	transcription regulator	H	1.917	0.001	-1.753	0.002	-1.995	0.001
gi_119889242_ref_XM_585895.3_	509019	ZBTB7B	zinc finger and BTB domain containing 7B	Nucleus	transcription regulator	H	1.900	0.001	-2.238	0.000	1.707	0.419
gi_119913325_ref_XM_604226.3_	525869	RAI14	retinoic acid induced 14	Nucleus	transcription regulator	L	1.855	0.002	-1.520	0.029	-1.143	0.594
gi_118151389_ref_NM_001077900.1_	510814	STAT1	signal transducer and activator of transcription 1, 91kD	Nucleus	transcription regulator	VH	1.847	0.001	1.189	1.255	1.154	1.333
gi_116003828_ref_NM_001076804.1_	505169	GATA3	GATA binding protein 3	Nucleus	transcription regulator	L	1.843	0.005	-1.330	0.117	1.126	1.133
gi_119915866_ref_XM_607555.3_	529115	HIVEP1	human immunodeficiency virus type I enhancer binding	Nucleus	transcription regulator	L	1.802	0.004	-1.680	0.006	-1.130	0.652
gi_190360732_ref_NM_001128499.1_	510050	TGIF1	TGFB-induced factor homeobox 1	Nucleus	transcription regulator	H	1.793	0.002	1.089	1.179	-1.444	0.070
gi_76611568_ref_XM_869196.1_	617024	E2F2	E2F transcription factor 2	Nucleus	transcription regulator	H	1.780	0.002	-2.427	0.000	-2.359	0.000
gi_118150933_ref_NM_001077914.1_	513047	ING1	inhibitor of growth family, member 1	Nucleus	transcription regulator	M	1.776	0.003	-1.629	0.010	-1.853	0.002
gi_119906085_ref_XM_608872.3_	530401	NFATC2	nuclear factor of activated T-cells, cytoplasmic, calcine	Nucleus	transcription regulator	M	1.755	0.004	-1.798	0.006	1.372	1.058
gi_119917017_ref_XM_877865.2_	509874	GTF3C1	general transcription factor IIIC, polypeptide 1, alpha 2	Nucleus	transcription regulator	H	1.748	0.002	-1.615	0.013	-1.028	1.046
gi_118150773_ref_NM_001077827.1_	280831	JUN	jun oncogene	Nucleus	transcription regulator	M	1.730	0.004	1.390	0.774	4.708	0.000
gi_119907762_ref_XM_590741.3_	513105	CREB3L1	cAMP responsive element binding protein 3-like 1	Nucleus	transcription regulator	L	1.710	0.004	1.651	0.334	1.330	1.179
gi_119905866_ref_XM_615740.3_	535632	TGIF2	TGFB-induced factor homeobox 2	Nucleus	transcription regulator	H	1.704	0.004	-1.476	0.045	-1.058	0.891
gi_116004218_ref_NM_001077001.1_	767928	RBPJ	recombination signal binding protein for immunoglobuli	Nucleus	transcription regulator	VH	1.689	0.004	1.042	1.151	-1.195	0.527
gi_115497487_ref_NM_001076141.1_	539003	CTNNB1	catenin (cadherin-associated protein), beta 1, 88kDa	Nucleus	transcription regulator	VH	1.680	0.004	1.025	1.137	-1.034	1.042

gi_78369189_ref_NM_001035349.1_	524118	ARID5A	AT rich interactive domain 5A (MRF1-like)	Nucleus	transcription regulator	H	1.592	0.006	-2.243	0.000	-1.682	0.008
gi_119901277_ref_XM_615634.3_	535530	FOXO3	forkhead box O3	Nucleus	transcription regulator	VH	1.571	0.007	-1.003	0.925	1.823	0.287
gi_115495776_ref_NM_001075448.1_	510420	MYBL2	v-myb myeloblastosis viral oncogene homolog (avian)-	Nucleus	transcription regulator	VH	1.567	0.008	-1.877	0.000	-2.078	0.000
gi_78042545_ref_NM_001035098.1_	511280	NMI	N-myc (and STAT) interactor	Cytoplasm	transcription regulator	H	1.564	0.007	1.158	1.234	-1.206	0.457
gi_119894953_ref_XM_001255535.1_	788491	ZBTB7A	zinc finger and BTB domain containing 7A	Nucleus	transcription regulator	H	1.550	0.010	-2.271	0.000	-1.444	0.073
gi_115497489_ref_NM_001075872.1_	525408	NFKBIB	nuclear factor of kappa light polypeptide gene enhance	Nucleus	transcription regulator	H	1.545	0.008	-1.911	0.000	-2.075	0.000
gi_119894776_ref_XR_028159.1_	784795	CARM1	coactivator-associated arginine methyltransferase 1	Nucleus	transcription regulator	VH	1.543	0.010	-1.542	0.024	-1.214	0.456
gi_84000132_ref_NM_001038072.1_	510666	BMI1	BMI1 polycomb ring finger oncogene	Nucleus	transcription regulator	VH	1.542	0.009	1.093	1.217	-1.036	1.038
gi_114052756_ref_NM_001045875.1_	407125	EGR1	early growth response 1	Nucleus	transcription regulator	H	1.533	0.010	-1.221	0.400	1.800	0.314
gi_47564055_ref_NM_001001150.1_	407219	MITF	microphthalmia-associated transcription factor	Nucleus	transcription regulator	H	1.532	0.010	1.643	0.373	1.919	0.196
gi_125630716_ref_NM_001081542.1_	541125	ELK3	ELK3, ETS-domain protein (SRF accessory protein 2)	Nucleus	transcription regulator	VH	1.517	0.011	-1.007	1.057	-1.079	0.887
gi_84370155_ref_NM_001038565.1_	539291	MYCBP	c-myc binding protein	Nucleus	transcription regulator	H	1.513	0.012	-1.597	0.013	-2.021	0.000
gi_115497653_ref_NM_001075240.1_	506097	NFIL3	nuclear factor, interleukin 3 regulated	Nucleus	transcription regulator	H	1.511	0.011	1.026	1.137	-1.191	0.536
gi_119911729_ref_XM_870907.2_	618576	MNT	MAX binding protein	Nucleus	transcription regulator	H	1.509	0.013	-2.683	0.000	1.009	1.092
gi_164420754_ref_NM_001076875.2_	525256	PREB	prolactin regulatory element binding	Nucleus	transcription regulator	H	1.507	0.013	-1.706	0.005	-1.772	0.004
gi_119911595_ref_XM_868906.2_	538671	SUPT6H	suppressor of Ty 6 homolog (S. cerevisiae)	Nucleus	transcription regulator	VH	1.504	0.013	-1.338	0.165	-1.077	0.908
gi_119915909_ref_XM_865312.2_	506141	IRF4	interferon regulatory factor 4	Nucleus	transcription regulator	H	1.470	0.018	-1.848	0.001	-2.502	0.000
gi_115496443_ref_NM_001075346.1_	508127	DMAP1	DNA methyltransferase 1 associated protein 1	Nucleus	transcription regulator	VH	1.458	0.018	-1.353	0.145	-1.244	0.351
gi_165905558_ref_NM_001046125.2_	512562	MAGED1	melanoma antigen family D, 1	Plasma Membrane	transcription regulator	VH	1.452	0.018	1.158	1.252	1.181	1.341
gi_119916184_ref_XM_596212.3_	518030	TAF4B	TAF4b RNA polymerase II, TATA box binding protein (T	Nucleus	transcription regulator	H	1.448	0.021	-1.169	0.541	-1.591	0.019
gi_99028933_ref_NM_001040645.1_	286857	PAX6	paired box 6	Nucleus	transcription regulator	M	1.446	0.022	4.146	0.000	5.370	0.000
gi_119915359_ref_XM_583214.3_	506727	NFKBIE	nuclear factor of kappa light polypeptide gene enhance	Nucleus	transcription regulator	H	1.419	0.028	-1.186	0.484	1.002	1.105
gi_66792933_ref_NM_001024574.1_	553098	NFIC	nuclear factor I/C (CCAAT-binding transcription factor)	Nucleus	transcription regulator	M	1.419	0.031	1.075	1.178	1.039	1.172
gi_114052113_ref_NM_001046375.1_	534228	PRPF6	PRP6 pre-mRNA processing factor 6 homolog (S. cere	Nucleus	transcription regulator	VH	1.415	0.026	-1.062	0.902	-1.072	0.915
gi_62751533_ref_NM_001015625.1_	520639	CLPB	ClpB caseinolytic peptidase B homolog (E. coli)	Nucleus	transcription regulator	H	1.405	0.030	-1.429	0.064	-1.847	0.003
gi_115497971_ref_NM_001076104.1_	538474	NFIB	nuclear factor I/B	Nucleus	transcription regulator	L	1.404	0.031	1.421	0.865	1.137	1.312
gi_119905767_ref_XM_606138.3_	527740	CBFA2T2	core-binding factor, runt domain, alpha subunit 2; trans	Nucleus	transcription regulator	H	1.402	0.032	1.114	1.186	1.269	1.330
gi_77736577_ref_NM_001034800.1_	617277	TCEB2	transcription elongation factor B (SIII), polypeptide 2 (1	Nucleus	transcription regulator	VH	1.401	0.031	-1.468	0.048	-1.421	0.090
gi_149642704_ref_NM_001099175.1_	618232	MED15	mediator complex subunit 15	Nucleus	transcription regulator	H	1.398	0.028	-1.943	0.000	-1.368	0.143
gi_119884881_ref_XM_606317.3_	527910	SKIL	SKI-like oncogene	Nucleus	transcription regulator	H	1.394	0.028	2.352	0.012	2.431	0.029
gi_119879733_ref_XM_614853.3_	541166	ETV5	ets variant 5	Nucleus	transcription regulator	VH	1.388	0.032	-1.266	0.303	-1.796	0.004
gi_119907153_ref_XM_594648.3_	516493	POU2F3	POU class 2 homeobox 3	Nucleus	transcription regulator	L	1.373	0.036	-1.129	0.718	-1.566	0.023
gi_119903024_ref_XM_581758.3_	505469	FOXN3	forkhead box N3	Nucleus	transcription regulator	M	1.373	0.037	1.271	1.180	1.690	0.450
gi_119904250_ref_XM_001251674.1_	783869	GTF3C5	general transcription factor IIIC, polypeptide 5, 63kDa	Nucleus	transcription regulator	VH	1.372	0.036	-1.400	0.097	-1.390	0.124
gi_164448549_ref_NM_001024497.2_	507867	ANKZF1	ankyrin repeat and zinc finger domain containing 1	Nucleus	transcription regulator	VH	1.371	0.041	-1.629	0.010	-1.725	0.006
gi_119912402_ref_XM_866236.2_	614679	HEXIM2	hexamthylene bis-acetamide inducible 2	Nucleus	transcription regulator	M	1.366	0.039	-1.593	0.013	-2.095	0.000
gi_154707855_ref_NM_001099177.1_	618386	TCF25	transcription factor 25 (basic helix-loop-helix)	Nucleus	transcription regulator	VH	1.365	0.037	-1.075	0.865	1.026	1.193
gi_115497343_ref_NM_001075627.1_	513544	SAP30BP	SAP30 binding protein	Nucleus	transcription regulator	VH	1.360	0.039	-1.219	0.296	-1.442	0.072
gi_31342852_ref_NM_174097.2_	281270	HOPX	HOP homeobox	Nucleus	transcription regulator	N	1.356	0.043	-1.138	0.443	-1.611	0.011
gi_119903596_ref_XM_614504.3_	534661	PAX8	paired box 8	Nucleus	transcription regulator	L	1.356	0.045	1.240	1.100	1.273	1.079
gi_62751635_ref_NM_001015527.1_	505702	GTF2F1	general transcription factor IIF, polypeptide 1, 74kDa	Nucleus	transcription regulator	VH	1.355	0.040	-1.424	0.071	-1.553	0.025
gi_119919541_ref_XM_586187.3_	509260	DEAF1	deformed epidermal autoregulatory factor 1 (Drosophila	Nucleus	transcription regulator	H	1.355	0.043	-1.206	0.444	-1.103	0.732
gi_134085849_ref_NM_001083382.1_	507180	PHF5A	PHD finger protein 5A	Nucleus	transcription regulator	VH	1.352	0.041	-1.605	0.012	-1.945	0.001
gi_77736090_ref_NM_001034572.1_	530409	PHB (includes EG	prohibitin	Nucleus	transcription regulator	VH	1.350	0.041	-1.498	0.030	-1.757	0.005
gi_156120882_ref_NM_001102118.1_	528433	DDX20	DEAD (Asp-Glu-Ala-Asp) box polypeptide 20	Nucleus	transcription regulator	VH	1.349	0.041	-1.069	0.905	-1.154	0.659
gi_119907824_ref_XM_583654.3_	507100	SPI1	spleen focus forming virus (SFFV) proviral integration o	Nucleus	transcription regulator	VH	1.349	0.042	1.111	1.209	1.256	1.335
gi_119915255_ref_XM_612306.3_	533039	SRF	serum response factor (c-fos serum response element-	Nucleus	transcription regulator	VH	1.346	0.043	-1.461	0.054	-1.263	0.334
gi_157280010_ref_NM_001105051.1_	100125778	RING1	ring finger protein 1	Nucleus	transcription regulator	VH	1.345	0.044	-1.632	0.010	-1.266	0.339
gi_119907105_ref_XM_585092.3_	508330	MLL	myeloid/lymphoid or mixed-lineage leukemia (trithorax	Nucleus	transcription regulator	H	1.343	0.044	-1.283	0.072	1.289	1.321
gi_119892074_ref_XM_613160.3_	533680	POU6F1	POU class 6 homeobox 1	Nucleus	transcription regulator	M	1.341	0.053	1.654	0.300	3.000	0.005
gi_119907798_ref_XM_585224.3_	508448	PSMC3	proteasome (prosome, macropain) 26S subunit, ATPas	Nucleus	transcription regulator	VH	1.339	0.048	-1.324	0.194	-1.507	0.043
gi_156121100_ref_NM_001102227.1_	539120	VEZF1	vascular endothelial zinc finger 1	Nucleus	transcription regulator	H	1.339	0.045	1.126	1.237	1.181	1.339
gi_6852489_gb_AW353499.1_AW35349	353105	STAT6	signal transducer and activator of transcription 6, interl	Nucleus	transcription regulator	VH	1.335	0.049	-1.169	0.544	1.202	1.341
gi_115497533_ref_NM_001075873.1_	525428	E2F4	E2F transcription factor 4, p107/p130-binding	Nucleus	transcription regulator	VH	1.333	0.049	-1.382	0.107	-1.447	0.069
gi_119902986_ref_XM_001253578.1_	785612	GTF2A1	general transcription factor IIA, 1, 19/37kDa	Cytoplasm	transcription regulator	H	-3.004	0.049	1.745	0.210	1.628	0.544
gi_149642758_ref_NM_001099186.1_	782690	TCF7	transcription factor 7 (T-cell specific, HMG-box)	Nucleus	transcription regulator	H	-3.006	0.047	3.702	0.000	3.762	0.001
gi_126722664_ref_NM_001082452.1_	539763	PARP11	poly (ADP-ribose) polymerase family, member 11	unknown	other	H	-3.012	0.045	1.826	0.147	2.414	0.030
gi_118151123_ref_NM_001078016.1_	538793	ZNF238	zinc finger protein 238	Nucleus	transcription regulator	H	-3.027	0.042	2.290	0.016	2.459	0.026
gi_77736300_ref_NM_001034678.1_	539547	HES1	hairy and enhancer of split 1, (Drosophila)	Nucleus	transcription regulator	H	-3.027	0.041	5.906	0.000	12.323	0.000
gi_114052622_ref_NM_001046113.1_	512254	MEF2C	myocyte enhancer factor 2C	Nucleus	transcription regulator	VH	-3.044	0.040	1.403	0.947	2.144	0.088
gi_82697320_ref_NM_001037445.1_	503577	DBP	D site of albumin promoter (albumin D-box) binding pro	Nucleus	transcription regulator	M	-3.085	0.039	1.835	0.147	2.068	0.113
gi_155371838_ref_NM_001101054.1_	505691	CDKN2C	cyclin-dependent kinase inhibitor 2C (p18, inhibits CDK	Nucleus	transcription regulator	VH	-3.118	0.034	1.619	0.422	1.431	1.012
gi_119888342_ref_XM_617930.3_	537747	KLF7	Kruppel-like factor 7 (ubiquitous)	Nucleus	transcription regulator	H	-3.374	0.018	3.378	0.000	3.028	0.005
gi_119905625_ref_XM_581853.3_	505552	MYT1	myelin transcription factor 1	Nucleus	transcription regulator	M	-3.505	0.022	-1.094	0.645	-1.049	0.837
gi_31340626_ref_NM_174725.2_	282711	EPAS1	endothelial PAS domain protein 1	Nucleus	transcription regulator	M	-3.575	0.013	-1.279	0.250	2.332	0.042
gi_119892029_ref_XM_873794.2_	317660	HOXC9	homeobox C9	Nucleus	transcription regulator	M	-3.619	0.011	1.031	0.951	1.151	1.299
gi_119905934_ref_XM_598075.3_	519845	TOX2	TOX high mobility group box family member 2	Nucleus	transcription regulator	M	-3.625	0.011	1.150	1.091	1.598	0.588
gi_119918399_ref_XR_027461.1_	781665	IRF2BP2	interferon regulatory factor 2 binding protein 2	Nucleus	transcription regulator	VH	-3.868	0.006	2.524	0.006	2.921	0.006
gi_158519872_ref_NM_001110095.1_	782847	ZHX1	zinc fingers and homeoboxes 1	Nucleus	transcription regulator	VH	-3.902	0.006	1.943	0.090	2.116	0.093
gi_75832071_ref_NM_174730.2_	282846	PYCARD	PYD and CARD domain containing	Cytoplasm	transcription regulator	H	-3.906	0.006	2.311	0.014	2.603	0.015
gi_156120990_ref_NM_001102172.1_	534475	RFX2	regulatory factor X, 2 (influences HLA class II expressi	Nucleus	transcription regulator	H	-4.055	0.005	2.215	0.022	2.505	0.022
gi_114050810_ref_NM_001046507.1_	613523	EAF2	ELL associated factor 2	Nucleus	transcription regulator	H	-4.060	0.005	-1.223	0.353	-1.638	0.009
gi_149944722_ref_NM_001099000.1_	517588	GLI1	GLI family zinc finger 1	Nucleus	transcription regulator	M	-4.104	0.005	1.615	0.403	2.109	0.096
gi_119921700_ref_XM_873620.2_	539139	KLF9	Kruppel-like factor 9	Nucleus	transcription regulator	M	-4.111	0.005	1.895	0.104	3.042	0.005

gi_62751708_ref_NM_001015520.1_	505233	LASS4	LAG1 homolog, ceramide synthase 4	Cytoplasm	transcription regulator	M	-4.131	0.004	1.624	0.349	4.282	0.000
gi_119911320_ref_XM_001256793.1_	790276	ZNF256	zinc finger protein 256	Nucleus	transcription regulator	H	-4.273	0.003	1.608	0.429	2.090	0.105
gi_119914354_ref_XM_001250711.1_	782819	ZNF35	zinc finger protein 35	Nucleus	transcription regulator	L	-4.450	0.003	1.118	1.202	1.173	1.332
gi_119879546_ref_XM_001251104.1_	540789	PARP14	poly (ADP-ribose) polymerase family, member 14	Cytoplasm	other	H	-4.897	0.001	4.644	0.000	5.496	0.000
gi_119906406_ref_XM_616463.3_	536336	ST18	suppression of tumorigenicity 18 (breast carcinoma) (zi	Nucleus	transcription regulator	M	-4.978	0.001	1.805	0.156	3.084	0.004
gi_114051376_ref_NM_001046050.1_	510244	CDKN2D	cyclin-dependent kinase inhibitor 2D (p19, inhibits CDK	Nucleus	transcription regulator	H	-4.994	0.001	-1.342	0.110	1.030	1.096
gi_156139064_ref_NM_001102373.1_	613740	HOXC4	homeobox C4	Nucleus	transcription regulator	M	-5.003	0.001	1.370	0.883	1.684	0.431
gi_119920266_ref_XM_584087.3_	538983	TAF9B	TAF9B RNA polymerase II, TATA box binding protein (T	Nucleus	transcription regulator	VH	-5.401	0.001	2.948	0.000	3.124	0.004
gi_119914390_ref_XM_593599.3_	515564	ZNF197	zinc finger protein 197	Nucleus	transcription regulator	H	-5.491	0.000	1.844	0.136	2.376	0.036
gi_119911158_ref_XM_001254867.1_	509810	ZNF256	zinc finger protein 256	Nucleus	transcription regulator	M	-5.493	0.001	1.040	1.065	1.286	1.276
gi_156121204_ref_NM_001102280.1_	614517	GAS7	growth arrest-specific 7	Cytoplasm	transcription regulator	VH	-5.542	0.000	1.375	0.996	1.621	0.574
gi_76668819_ref_XM_864611.1_	537223	SIM1	single-minded homolog 1 (Drosophila)	Nucleus	transcription regulator	M	-6.813	0.000	2.914	0.000	2.196	0.067
gi_119917624_ref_XM_614245.3_	534470	LBX1	ladybird homeobox 1	Nucleus	transcription regulator	M	-6.966	0.000	-1.097	0.392	1.003	0.796
gi_115496541_ref_NM_001076531.1_	767586	KANK2	KN motif and ankyrin repeat domains 2	Nucleus	transcription regulator	H	-7.103	0.000	1.597	0.441	2.209	0.066
gi_115496619_ref_NM_001075708.1_	515334	ZNF350	zinc finger protein 350	Nucleus	transcription regulator	M	-7.564	0.000	1.136	0.944	-1.129	0.402
gi_149642786_ref_NM_001099152.1_	616148	LBH	limb bud and heart development homolog (mouse)	Nucleus	transcription regulator	VH	-7.566	0.000	1.514	0.664	2.657	0.012
gi_125991955_ref_NM_001081613.1_	540676	IKZF3	IKAROS family zinc finger 3 (Aiolos)	Nucleus	transcription regulator	M	-7.714	0.000	2.715	0.003	2.229	0.063
gi_119910890_ref_XR_028348.1_	537103	ZNF175	zinc finger protein 175	Nucleus	transcription regulator	M	-7.866	0.000	1.110	1.210	1.434	0.948
gi_31340727_ref_NM_174687.2_	282591	ASB15	ankyrin repeat and SOCS box-containing 15	Nucleus	transcription regulator	M	-7.957	0.000	-1.069	0.870	1.071	1.214
gi_149773516_ref_NM_001099106.1_	540313	ETS1	v-ets erythroblastosis virus E26 oncogene homolog 1 (	Nucleus	transcription regulator	VH	-8.655	0.000	-1.299	0.127	-1.439	0.066
gi_115496321_ref_NM_001075915.1_	528475	POU2AF1	POU class 2 associating factor 1	Nucleus	transcription regulator	VH	-8.996	0.000	1.633	0.406	1.551	0.707
gi_119900560_ref_XM_618574.3_	538371	PAX5	paired box 5	Nucleus	transcription regulator	VH	-9.835	0.000	1.096	1.206	1.617	0.567
gi_119902037_ref_XM_864937.2_	535307	NEO1	neogenin homolog 1 (chicken)	Plasma Membrane	transcription regulator	M	-9.887	0.000	1.780	0.135	1.984	0.145
gi_115497895_ref_NM_001075612.1_	513316	KCNIP3	Kv channel interacting protein 3, calsenilin	Nucleus	transcription regulator	M	-10.426	0.000	1.780	0.156	2.895	0.007
gi_119911185_ref_XM_001256592.1_	789989	ZNF211	zinc finger protein 211	Nucleus	transcription regulator	H	-10.771	0.000	-1.136	0.588	-1.073	0.857
gi_119915881_ref_XM_587261.3_	539454	GCM2	glial cells missing homolog 2 (Drosophila)	Nucleus	transcription regulator	M	-10.783	0.000	1.200	1.218	1.374	1.060
gi_94966920_ref_NM_001040563.1_	539276	HTATIP2	HIV-1 Tat interactive protein 2, 30kDa	Nucleus	transcription regulator	M	-11.018	0.000	1.147	1.116	1.270	1.241
gi_119895769_ref_XM_617891.3_	537712	EBF1	early B-cell factor 1	Nucleus	transcription regulator	VH	-11.159	0.000	1.459	0.788	1.462	0.928
gi_156120434_ref_NM_001101893.1_	508025	ZNF423	zinc finger protein 423	Nucleus	transcription regulator	M	-13.720	0.000	1.342	0.966	1.313	1.228
gi_119910807_ref_XM_001252761.1_	784460	SPIB	Spi-B transcription factor (Spi-1/PU.1 related)	Nucleus	transcription regulator	H	-17.754	0.000	1.380	0.795	2.313	0.045
gi_164451497_ref_NM_001024565.2_	538609	HEYL	hairy/enhancer-of-split related with YRPW motif-like	Nucleus	transcription regulator	H	-26.514	0.000	1.804	0.168	2.719	0.010
gi_156120738_ref_NM_001102046.1_	517861	POU6F2 (includes	POU class 6 homeobox 2	Nucleus	transcription regulator	H	-27.841	0.000	1.283	1.141	1.826	0.279
gi_119900339_ref_XM_614789.3_	534869	KANK1	KN motif and ankyrin repeat domains 1	Nucleus	transcription regulator	VH	-34.265	0.000	3.082	0.000	2.505	0.022
gi_119890798_ref_XM_615498.3_	535415	HDAC9 (includes	histone deacetylase 9	Nucleus	transcription regulator	H	-36.787	0.000	7.269	0.000	18.700	0.000
gi_119893570_ref_XM_615475.3_	535399	LEF1	lymphoid enhancer-binding factor 1	Nucleus	transcription regulator	VH	-60.452	0.000	3.948	0.000	3.572	0.001
gi_62460541_ref_NM_001014923.1_	514006	NFE2	nuclear factor (erythroid-derived 2), 45kDa	Nucleus	transcription regulator	H	-78.828	0.000	1.191	1.237	1.244	1.298

Table S7B: Translation

SEQ_ID	Entrez gene ID	Symbol	Entrez Gene Name	Location	Type	Predicted BL20 expression level	FC BLvTBL	FDR BLvsTBL	FC TBLvTBL24h	FDR TBLvTBL24h	FC TBLvTBL48h	FDR TBLvTBL48h
gi_148233315_ref_NM_001098165.1_	100048947	RNASEL	ribonuclease L (2',5'-oligoisoadenylate synthetase-depe	Cytoplasm	enzyme	M	11.966	0.000	-1.284	0.255	-1.357	0.161
gi_119923325_ref_XM_584997.3_	508245	RNASET2	ribonuclease T2	Extracellular Space	enzyme	H	4.757	0.000	1.076	1.192	1.338	1.227
gi_147903999_ref_NM_001098126.1_	614429	YBX2	Y box binding protein 2	Cytoplasm	translation regulator	M	3.082	0.000	2.191	0.024	2.271	0.054
gi_134085871_ref_NM_001083384.1_	507417	ABTB1	ankyrin repeat and BTB (POZ) domain containing 1	Cytoplasm	translation regulator	H	2.904	0.000	-1.961	0.000	-1.013	1.103
gi_114052031_ref_NM_001046387.1_	534734	MRI1	methylthioribose-1-phosphate isomerase homolog (S. c	Cytoplasm	translation regulator	H	2.002	0.001	-1.576	0.016	-2.042	0.000
gi_115495424_ref_NM_001075874.1_	525521	RPUSD1	RNA pseudouridylate synthase domain containing 1	unknown	enzyme	H	1.941	0.001	-1.422	0.073	-1.335	0.198
gi_149944724_ref_NM_001099185.1_	782674	NOL6	nucleolar protein family 6 (RNA-associated)	Nucleus	other	H	1.888	0.001	-2.634	0.000	-2.754	0.000
gi_126165253_ref_NM_001081735.1_	540139	REXO2	REX2, RNA exonuclease 2 homolog (S. cerevisiae)	Cytoplasm	enzyme	VH	1.749	0.002	-1.304	0.239	-1.682	0.009
gi_119893809_ref_XM_613340.3_	540831	RBM47	RNA binding motif protein 47	unknown	other	M	1.700	0.004	1.060	1.188	1.027	1.100
gi_134085929_ref_NM_001083399.1_	509932	MRTO4	mRNA turnover 4 homolog (S. cerevisiae)	Cytoplasm	other	VH	1.686	0.003	-1.812	0.001	-1.988	0.001
gi_119914241_ref_XM_001252033.1_	784766	RBMS3	RNA binding motif, single stranded interacting protein	unknown	other	L	1.682	0.006	2.099	0.042	1.546	0.738
gi_115497695_ref_NM_001075589.1_	512925	RRP1	ribosomal RNA processing 1 homolog (S. cerevisiae)	Nucleus	other	VH	1.671	0.004	-1.554	0.020	-1.725	0.006
gi_119911687_ref_XM_883226.2_	531049	KIAA0664	KIAA0664	Cytoplasm	translation regulator	H	1.671	0.005	-2.295	0.000	-1.562	0.023
gi_114051909_ref_NM_001045955.1_	507065	RBM19	RNA binding motif protein 19	Nucleus	other	VH	1.635	0.006	-1.316	0.206	-1.442	0.072
gi_156121190_ref_NM_001102273.1_	614025	RRP9	ribosomal RNA processing 9, small subunit (SSU) proc	Nucleus	other	VH	1.621	0.006	-2.021	0.000	-2.028	0.000
gi_119913741_ref_XM_606001.3_	527607	EFTUD1	elongation factor Tu GTP binding domain containing 1	Cytoplasm	translation regulator	VH	1.547	0.008	-1.158	0.596	-1.388	0.121
gi_41386714_ref_NM_174830.1_	286811	EIF6	eukaryotic translation initiation factor 6	Cytoplasm	translation regulator	VH	1.547	0.008	-1.642	0.010	-1.721	0.007
gi_119889249_ref_XM_581374.3_	505134	ADAR	adenosine deaminase, RNA-specific	Nucleus	enzyme	H	1.521	0.010	1.366	1.070	1.647	0.543
gi_77735812_ref_NM_001034429.1_	512886	RNASEK	ribonuclease, RNase K	unknown	peptidase	VH	1.518	0.011	1.054	1.143	-1.013	1.078
gi_134085616_ref_NM_001083464.1_	532824	EEFSEC	eukaryotic elongation factor, selenocysteine-tRNA-spe	Cytoplasm	translation regulator	H	1.513	0.012	-1.513	0.032	-1.697	0.008
gi_83523772_ref_NM_174788.3_	286853	RPLP2	ribosomal protein, large, P2	Cytoplasm	other	VH	1.506	0.013	-1.469	0.041	-1.411	0.102
gi_45430014_ref_NM_205794.1_	404130	EIF2C2	eukaryotic translation initiation factor 2C, 2	Cytoplasm	translation regulator	VH	1.504	0.013	-1.288	0.272	-1.139	0.697
gi_75812945_ref_NM_001033622.1_	404165	NOP56	NOP56 ribonucleoprotein homolog (yeast)	Nucleus	other	VH	1.495	0.012	-1.283	0.265	-1.388	0.124
gi_119910146_ref_XR_028278.1_	513171	EDC4	enhancer of mRNA decapping 4	unknown	other	H	1.492	0.014	-1.663	0.007	-1.659	0.010
gi_119891303_ref_XM_583691.3_	507132	RBM28	RNA binding motif protein 28	Nucleus	other	H	1.490	0.015	-1.407	0.081	-2.114	0.000
gi_77404208_ref_NM_001034044.1_	286819	EIF4A2	eukaryotic translation initiation factor 4A, isoform 2	Cytoplasm	translation regulator	VH	1.483	0.015	1.059	1.155	-1.091	0.840
gi_156120514_ref_NM_001101933.1_	510820	TSR1	TSR1, 20S rRNA accumulation, homolog (S. cerevisiae	unknown	other	VH	1.464	0.017	-1.294	0.241	-1.486	0.051
gi_31342599_ref_NM_174204.2_	281551	TSFM (includes E	Ts translation elongation factor, mitochondrial	Cytoplasm	translation regulator	H	1.461	0.017	-1.516	0.024	-2.047	0.000
gi_118601775_ref_NM_001079588.1_	511560	IMP3	IMP3, U3 small nucleolar ribonucleoprotein, homolog (	Cytoplasm	other	H	1.455	0.018	-1.623	0.010	-1.991	0.001

gi_94966953_ref_NM_001040581.1_	615178	RPS21	ribosomal protein S21	Cytoplasm	other	VH	1.453	0.021	-1.489	0.032	-1.300	0.233
gi_119906215_ref_XM_594628.3_	516473	EEF1D	eukaryotic translation elongation factor 1 delta (guanin	Cytoplasm	translation regulator	VH	1.441	0.021	-1.617	0.011	-1.403	0.111
gi_115497461_ref_NM_001076143.1_	539052	EIF3J	eukaryotic translation initiation factor 3, subunit J	Cytoplasm	translation regulator	VH	1.439	0.021	-1.408	0.063	-1.725	0.006
gi_119892449_ref_XM_001252988.1_	538820	PAN2 (includes E	PAN2 poly(A) specific ribonuclease subunit homolog (S	Cytoplasm	peptidase	VH	1.421	0.025	1.249	1.253	1.075	1.258
gi_78369251_ref_NM_001035361.1_	526135	RBMS1	RNA binding motif, single stranded interacting protein 1	Nucleus	other	VH	1.415	0.025	1.350	1.106	1.166	1.332
gi_114052113_ref_NM_001046375.1_	534228	PRPF6	PRP6 pre-mRNA processing factor 6 homolog (S. cere	Nucleus	transcription regulator	VH	1.415	0.026	-1.062	0.902	-1.072	0.915
gi_134085778_ref_NM_001083474.1_	534063	EIF2B3	eukaryotic translation initiation factor 2B, subunit 3 gan	Cytoplasm	translation regulator	VH	1.404	0.027	-1.003	1.085	-1.400	0.117
gi_115497947_ref_NM_001075481.1_	510956	RRS1	RRS1 ribosome biogenesis regulator homolog (S. cere	Nucleus	other	VH	1.398	0.029	-1.557	0.020	-1.963	0.001
gi_84000148_ref_NM_001038085.1_	511723	RPP38	ribonuclease P/MRP 38kDa subunit	Nucleus	enzyme	H	1.372	0.036	-1.586	0.015	-1.961	0.001
gi_139948859_ref_NM_001083679.1_	513598	SMG5	Smg-5 homolog, nonsense mediated mRNA decay fact	Nucleus	other	VH	1.370	0.038	-2.320	0.000	-1.432	0.075
gi_77736196_ref_NM_001034625.1_	535056	RPL8	ribosomal protein L8	Cytoplasm	other	VH	1.362	0.037	-1.593	0.013	-1.549	0.028
gi_119881238_ref_XM_001251995.1_	783760	EIF2B5	eukaryotic translation initiation factor 2B, subunit 5 eps	Cytoplasm	translation regulator	VH	1.361	0.038	-1.054	0.953	-1.437	0.080
gi_62460451_ref_NM_001014876.1_	508948	RSL1D1	ribosomal L1 domain containing 1	Cytoplasm	other	VH	1.349	0.042	-1.224	0.420	-1.409	0.104
gi_156718145_ref_NM_001103108.1_	100125222	RAVER1	ribonucleoprotein, PTB-binding 1	Nucleus	other	VH	1.330	0.049	-1.677	0.006	-1.493	0.048
gi_77736378_ref_NM_001034718.1_	540982	GLE1	GLE1 RNA export mediator homolog (yeast)	Nucleus	other	VH	1.320	0.054	-1.165	0.617	-1.426	0.096
gi_156121354_ref_NM_001102356.1_	789999	EIF3B	eukaryotic translation initiation factor 3, subunit B	Cytoplasm	translation regulator	VH	1.318	0.053	-1.273	0.286	-1.326	0.216
gi_119895025_ref_XM_610805.3_	532293	BRUNOL5	bruno-like 5, RNA binding protein (Drosophila)	unknown	other	M	-3.310	0.022	1.290	1.217	1.684	0.477
gi_119923409_ref_XM_613436.3_	540852	SAMD4A	sterile alpha motif domain containing 4A	Cytoplasm	translation regulator	H	-3.339	0.021	1.092	1.215	1.230	1.342
gi_119917775_ref_XM_603772.3_	525419	RBM20	RNA binding motif protein 20	unknown	other	M	-3.563	0.013	1.414	0.838	1.575	0.650
gi_119902448_ref_XM_596374.3_	518188	RBPMS2	RNA binding protein with multiple splicing 2	unknown	other	M	-4.875	0.001	1.232	1.248	1.332	1.248
gi_114052323_ref_NM_001046535.1_	614417	RBPMS	RNA binding protein with multiple splicing	unknown	other	VH	-6.600	0.000	-1.291	0.234	2.448	0.028
gi_119901747_ref_XM_596135.3_	517953	RPS6KA2	ribosomal protein S6 kinase, 90kDa, polypeptide 2	Nucleus	kinase	H	-7.201	0.000	1.119	1.214	1.087	1.149
gi_119912092_ref_XM_595012.3_	516853	IGF2BP1	insulin-like growth factor 2 mRNA binding protein 1	Cytoplasm	translation regulator	M	-9.065	0.000	1.226	1.242	1.834	0.272
gi_31341580_ref_NM_174594.2_	282341	RNASE6	ribonuclease, RNase A family, k6	Extracellular Space	enzyme	H	-11.457	0.000	14.633	0.000	18.983	0.000
gi_119920009_ref_XM_601830.3_	523530	NXF3	nuclear RNA export factor 3	Nucleus	transporter	VH	-166.634	0.000	1.449	0.683	1.393	0.942
