# Supplementary material for: A Bovine Lymphosarcoma Cell Line Infected with Theileria annulata Exhibits an Irreversible Reconfiguration of Host Cell Gene Expression
Source: PLoS One. 2013 Jun 26;8(6):e66833. doi: 10.1371/journal.pone.0066833 (PMC3694138; doi:10.1371/journal.pone.0066833)
Supplement: Table S8 — Transcription regulators predicted to be highly expressed in BL20. (PDF) [file pone.0066833.s010.pdf]

Table S8: Transcription regulators predicted to be highly expressed in BL20

SEQ_ID	Entrez gene ID	Symbol	Entrez Gene Name	Location	Type	Predicted BL20 expression level	FC BLvTBL	FDR BLvsTBL	FC TBLvTBL24h	FDR TBLvTBL24h	FC TBLvTBL48h	FDR TBLvTBL48h
gi_62460541_ref_NM_001014923.1_	514006	NFE2	nuclear factor (erythroid-derived 2), 45kDa	Nucleus	transcription regulator	H	-78.828	0.000	1.191	1.237	1.244	1.298
gi_119893570_ref_XM_615475.3_	535399	LEF1	lymphoid enhancer-binding factor 1	Nucleus	transcription regulator	VH	-60.452	0.000	3.948	0.000	3.572	0.001
gi_119890798_ref_XM_615498.3_	535415	HDAC9 (includes	histone deacetylase 9	Nucleus	transcription regulator	H	-36.787	0.000	7.269	0.000	18.700	0.000
gi_119900339_ref_XM_614789.3_	534869	KANK1	KN motif and ankyrin repeat domains 1	Nucleus	transcription regulator	VH	-34.265	0.000	3.082	0.000	2.505	0.022
gi_156120738_ref_NM_001102046.1_	517861	POU6F2 (includes	POU class 6 homeobox 2	Nucleus	transcription regulator	H	-27.841	0.000	1.283	1.141	1.826	0.279
gi_164451497_ref_NM_001024565.2_	538609	HEYL	hairy/enhancer-of-split related with YRPW motif-like	Nucleus	transcription regulator	H	-26.514	0.000	1.804	0.168	2.719	0.010
gi_119910807_ref_XM_001252761.1_	784460	SPIB	Spi-B transcription factor (Spi-1/PU.1 related)	Nucleus	transcription regulator	H	-17.754	0.000	1.380	0.795	2.313	0.045
gi_119895769_ref_XM_617891.3_	537712	EBF1	early B-cell factor 1	Nucleus	transcription regulator	VH	-11.159	0.000	1.459	0.788	1.462	0.928
gi_119911185_ref_XM_001256592.1_	789989	ZNF211	zinc finger protein 211	Nucleus	transcription regulator	H	-10.771	0.000	-1.136	0.588	-1.073	0.857
gi_119900560_ref_XM_618574.3_	538371	PAX5	paired box 5	Nucleus	transcription regulator	VH	-9.835	0.000	1.096	1.206	1.617	0.567
gi_115496321_ref_NM_001075915.1_	528475	POU2AF1	POU class 2 associating factor 1	Nucleus	transcription regulator	VH	-8.996	0.000	1.633	0.406	1.551	0.707
gi_149773516_ref_NM_001099106.1_	540313	ETS1	v-ets erythroblastosis virus E26 oncogene homolog 1 (	Nucleus	transcription regulator	VH	-8.655	0.000	-1.299	0.127	-1.439	0.066
gi_149642786_ref_NM_001099152.1_	616148	LBH	limb bud and heart development homolog (mouse)	Nucleus	transcription regulator	VH	-7.566	0.000	1.514	0.664	2.657	0.012
gi_115496541_ref_NM_001076531.1_	767586	KANK2	KN motif and ankyrin repeat domains 2	Nucleus	transcription regulator	H	-7.103	0.000	1.597	0.441	2.209	0.066
gi_156121204_ref_NM_001102280.1_	614517	GAS7	growth arrest-specific 7	Cytoplasm	transcription regulator	VH	-5.542	0.000	1.375	0.996	1.621	0.574
gi_119914390_ref_XM_593599.3_	515564	ZNF197	zinc finger protein 197	Nucleus	transcription regulator	H	-5.491	0.000	1.844	0.136	2.376	0.036
gi_119920266_ref_XM_584087.3_	538983	TAF9B	TAF9B RNA polymerase II, TATA box binding protein (T	Nucleus	transcription regulator	VH	-5.401	0.001	2.948	0.000	3.124	0.004
gi_114051376_ref_NM_001046050.1_	510244	CDKN2D	cyclin-dependent kinase inhibitor 2D (p19, inhibits CDK	Nucleus	transcription regulator	H	-4.994	0.001	-1.342	0.110	1.030	1.096
gi_119911320_ref_XM_001256793.1_	790276	ZNF256	zinc finger protein 256	Nucleus	transcription regulator	H	-4.273	0.003	1.608	0.429	2.090	0.105
gi_114050810_ref_NM_001046507.1_	613523	EAF2	ELL associated factor 2	Nucleus	transcription regulator	H	-4.060	0.005	-1.223	0.353	-1.638	0.009
gi_156120990_ref_NM_001102172.1_	534475	RFX2	regulatory factor X, 2 (influences HLA class II expressi	Nucleus	transcription regulator	H	-4.055	0.005	2.215	0.022	2.505	0.022
gi_75832071_ref_NM_174730.2_	282846	PYCARD	PYD and CARD domain containing	Cytoplasm	transcription regulator	H	-3.906	0.006	2.311	0.014	2.603	0.015
gi_158519872_ref_NM_001110095.1_	782847	ZHX1	zinc fingers and homeoboxes 1	Nucleus	transcription regulator	VH	-3.902	0.006	1.943	0.090	2.116	0.093
gi_119918399_ref_XR_027461.1_	781665	IRF2BP2	interferon regulatory factor 2 binding protein 2	Nucleus	transcription regulator	VH	-3.868	0.006	2.524	0.006	2.921	0.006
gi_119888342_ref_XM_617930.3_	537747	KLF7	Kruppel-like factor 7 (ubiquitous)	Nucleus	transcription regulator	H	-3.374	0.018	3.378	0.000	3.028	0.005
gi_155371838_ref_NM_001101054.1_	505691	CDKN2C	cyclin-dependent kinase inhibitor 2C (p18, inhibits CDK	Nucleus	transcription regulator	VH	-3.118	0.034	1.619	0.422	1.431	1.012
gi_114052622_ref_NM_001046113.1_	512254	MEF2C	myocyte enhancer factor 2C	Nucleus	transcription regulator	VH	-3.044	0.040	1.403	0.947	2.144	0.088
gi_77736300_ref_NM_001034678.1_	539547	HES1	hairy and enhancer of split 1, (Drosophila)	Nucleus	transcription regulator	H	-3.027	0.041	5.906	0.000	12.323	0.000
gi_118151123_ref_NM_001078016.1_	538793	ZNF238	zinc finger protein 238	Nucleus	transcription regulator	H	-3.027	0.042	2.290	0.016	2.459	0.026
gi_149642758_ref_NM_001099186.1_	782690	TCF7	transcription factor 7 (T-cell specific, HMG-box)	Nucleus	transcription regulator	H	-3.006	0.047	3.702	0.000	3.762	0.001
gi_119902986_ref_XM_001253578.1_	785612	GTF2A1	general transcription factor IIA, 1, 19/37kDa	Cytoplasm	transcription regulator	H	-3.004	0.049	1.745	0.210	1.628	0.544
gi_119909958_ref_XM_001251963.1_	523815	CBFA2T3	core-binding factor, runt domain, alpha subunit 2; trans	Nucleus	transcription regulator	VH	-2.949	0.053	2.170	0.028	2.319	0.044
gi_77736282_ref_NM_001034669.1_	539244	ASB2	ankyrin repeat and SOCS box-containing 2	Nucleus	transcription regulator	H	-2.938	0.055	1.090	1.168	2.502	0.022
gi_115497505_ref_NM_001076222.1_	540474	LMCD1	LIM and cysteine-rich domains 1	Cytoplasm	transcription regulator	H	-2.917	0.054	1.106	1.215	-1.230	0.404
gi_41386732_ref_NM_177490.1_	280961	ZFX	zinc finger protein, X-linked	Nucleus	transcription regulator	VH	-2.914	0.055	1.871	0.131	1.761	0.354
gi_119916759_ref_XM_585540.3_	508720	CIITA	class II, major histocompatibility complex, transactivato	Nucleus	transcription regulator	H	-2.903	0.064	-2.235	0.000	-1.446	0.068
gi_115497499_ref_NM_001075894.1_	527124	CDKN2B	cyclin-dependent kinase inhibitor 2B (p15, inhibits CDK	Nucleus	transcription regulator	H	-2.797	0.087	1.564	0.393	2.129	0.089
gi_119889385_ref_XM_590074.3_	539884	RFX5	regulatory factor X, 5 (influences HLA class II expressi	Nucleus	transcription regulator	H	-2.768	0.083	1.860	0.136	1.737	0.389
gi_119932393_ref_XM_001250820.1_	786432	MED7 (includes E	mediator complex subunit 7	Nucleus	transcription regulator	H	-2.757	0.090	2.167	0.025	3.450	0.001
gi_40538781_ref_NM_175050.1_	317776	MYB (includes EG	v-myb myeloblastosis viral oncogene homolog (avian)	Nucleus	transcription regulator	VH	-2.745	0.090	1.407	0.932	-1.210	0.464
gi_139949110_ref_NM_001083769.1_	614909	IRF8	interferon regulatory factor 8	Nucleus	transcription regulator	VH	-2.717	0.099	-2.021	0.000	-2.819	0.000
gi_125991921_ref_NM_001081583.1_	508893	TLE4	transducin-like enhancer of split 4 (E(sp1) homolog, Dr	Nucleus	transcription regulator	VH	-2.658	0.116	1.184	1.249	1.332	1.212
gi_119885693_ref_XM_589799.3_	539843	ZBTB38	zinc finger and BTB domain containing 38	Nucleus	transcription regulator	H	-2.637	0.131	2.061	0.048	2.099	0.104
gi_155371924_ref_NM_001101097.1_	519165	HLX	H2.0-like homeobox	Nucleus	transcription regulator	H	-2.635	0.124	1.600	0.443	1.747	0.373
gi_148226872_ref_NM_001098035.1_	515406	CSRNP1	cysteine-serine-rich nuclear protein 1	Nucleus	transcription regulator	H	-2.609	0.134	1.114	1.207	1.682	0.468
gi_156121294_ref_NM_001102325.1_	781834	ATF7IP	activating transcription factor 7 interacting protein	Nucleus	transcription regulator	H	-2.608	0.138	2.295	0.015	2.561	0.019
gi_156523181_ref_NM_001102535.1_	785815	HR	hairless homolog (mouse)	Nucleus	transcription regulator	H	-2.563	0.155	-1.758	0.009	1.462	0.903
gi_156523193_ref_NM_001102541.1_	100124497	ZNF256	zinc finger protein 256	Nucleus	transcription regulator	H	-2.559	0.156	1.593	0.487	1.557	0.722
gi_119890844_ref_XM_864821.2_	539676	DMTF1	cyclin D binding myb-like transcription factor 1	Nucleus	transcription regulator	H	-2.548	0.151	1.993	0.067	2.573	0.018
gi_119915240_ref_XM_865759.2_	504797	TRERF1	transcriptional regulating factor 1	Nucleus	transcription regulator	H	-2.527	0.170	2.437	0.008	3.000	0.005
gi_75677539_ref_NM_001033121.1_	613265	ZNF148	zinc finger protein 148	Nucleus	transcription regulator	H	-2.509	0.179	1.443	0.828	1.369	1.129
gi_116004232_ref_NM_001077008.1_	767935	SERTAD3	SERTA domain containing 3	Nucleus	transcription regulator	H	-2.475	0.194	1.816	0.170	2.462	0.026
gi_114051256_ref_NM_001046193.1_	515266	ATF3	activating transcription factor 3	Nucleus	transcription regulator	H	-2.420	0.223	-1.246	0.335	2.237	0.060
gi_139949255_ref_NM_001083638.1_	407231	MEF2A (includes	myocyte enhancer factor 2A	Nucleus	transcription regulator	VH	-2.410	0.228	2.079	0.045	2.417	0.029
gi_118150815_ref_NM_001077853.1_	505251	ELF1	E74-like factor 1 (ets domain transcription factor)	Nucleus	transcription regulator	VH	-2.410	0.225	1.783	0.205	1.817	0.302
gi_78369187_ref_NM_001035478.1_	616564	SSBP2	single-stranded DNA binding protein 2	Nucleus	transcription regulator	VH	-2.397	0.228	2.042	0.054	2.537	0.020
gi_115496825_ref_NM_001076041.1_	534915	ASCC1	activating signal cointegrator 1 complex subunit 1	Nucleus	transcription regulator	VH	-2.384	0.241	1.618	0.429	1.899	0.219
gi_119920199_ref_XM_592333.3_	514473	ATRX	alpha thalassemia/mental retardation syndrome X-linke	Nucleus	transcription regulator	VH	-2.371	0.238	1.652	0.328	2.030	0.130
gi_155372030_ref_NM_001101153.1_	534170	ZNF274	zinc finger protein 274	Nucleus	transcription regulator	H	-2.340	0.274	1.084	1.197	1.001	1.158
gi_155372268_ref_NM_001101277.1_	618793	FOXO4	forkhead box O4	Nucleus	transcription regulator	H	-2.320	0.278	1.686	0.295	2.457	0.026
gi_157073983_ref_NM_001103231.1_	506188	MEF2B	myocyte enhancer factor 2B	Nucleus	transcription regulator	H	-2.259	0.339	-1.121	0.662	-1.166	0.597
gi_154152090_ref_NM_001100344.1_	511794	ZNF192	zinc finger protein 192	Nucleus	transcription regulator	VH	-2.217	0.347	1.994	0.063	2.403	0.031
gi_156120762_ref_NM_001102058.1_	520250	ANKRD32	ankyrin repeat domain 32	Nucleus	transcription regulator	H	-2.202	0.398	1.763	0.231	1.240	1.341
gi_84000408_ref_NM_001038209.1_	615371	NFIA	nuclear factor I/A	Nucleus	transcription regulator	H	-2.169	0.407	3.492	0.000	4.474	0.000
gi_27819621_ref_NM_174493.1_	282127	ZFP36	zinc finger protein 36, C3H type, homolog (mouse)	Nucleus	transcription regulator	H	-2.166	0.417	1.050	1.132	2.240	0.058
gi_156739264_ref_NM_001075644.2_	514023	ZNF180	zinc finger protein 180	Nucleus	transcription regulator	H	-2.144	0.436	1.782	0.194	1.636	0.551
gi_119919077_ref_XM_001251164.1_	507760	PRDM10	PR domain containing 10	Nucleus	transcription regulator	H	-2.134	0.431	1.453	0.733	1.532	0.736
gi_148232125_ref_NM_001098062.1_	527056	RNF6	ring finger protein (C3H2C3 type) 6	Nucleus	transcription regulator	H	-2.125	0.474	1.255	1.251	1.471	0.884

gi_115497199_ref_NM_001076077.1_	536504	PHTF1	putative homeodomain transcription factor 1	Nucleus	transcription regulator	VH	-2.124	0.483	1.343	1.134	1.449	0.971
gi_119888259_ref_XM_614966.3_	541192	SATB2	SATB homeobox 2	Nucleus	transcription regulator	H	-2.111	0.479	1.516	0.643	1.221	1.341
gi_118151261_ref_NM_001078095.1_	768207	SS18L1	synovial sarcoma translocation gene on chromosome `	Nucleus	transcription regulator	H	-2.096	0.491	1.116	1.141	-1.185	0.373
gi_119887167_ref_XM_865771.2_	529631	RUNX1	runt-related transcription factor 1	Nucleus	transcription regulator	VH	-2.059	0.559	-2.009	0.000	-1.273	0.313
gi_156120724_ref_NM_001102039.1_	516781	NAB1	NGFI-A binding protein 1 (EGR1 binding protein 1)	Nucleus	transcription regulator	H	-2.047	0.568	1.346	1.095	1.320	1.242
gi_119901585_ref_XM_593747.3_	540396	HIVEP2	human immunodeficiency virus type I enhancer binding	Nucleus	transcription regulator	VH	-2.047	0.555	-1.182	0.520	-1.288	0.263
gi_119917098_ref_XM_601985.3_	523683	CUX1	cut-like homeobox 1	Nucleus	transcription regulator	VH	-2.015	0.586	1.102	1.071	1.584	0.640
gi_84370150_ref_NM_001038559.1_	533232	SMARCD1	SWI/SNF related, matrix associated, actin dependent r	Nucleus	transcription regulator	VH	-1.997	0.639	1.217	1.257	1.597	0.630
gi_78369035_ref_NM_001035424.1_	539376	MYNN	myoneurin	Nucleus	transcription regulator	H	-1.982	0.667	1.725	0.265	1.530	0.772
gi_119905298_ref_XM_615192.3_	535183	ZEB1	zinc finger E-box binding homeobox 1	Nucleus	transcription regulator	VH	-1.982	0.656	-1.146	0.671	1.093	1.300
gi_119918507_ref_XM_864818.2_	613629	SIRT1	sirtuin (silent mating type information regulation 2 hom	Nucleus	transcription regulator	VH	-1.981	0.671	1.980	0.073	2.107	0.099
gi_115495882_ref_NM_001076242.1_	540818	GABPB2	GA binding protein transcription factor, beta subunit 2	Nucleus	transcription regulator	H	-1.974	0.656	1.445	0.759	1.180	1.328
gi_112817611_ref_NM_001015514.2_	504512	ETV6	ets variant 6	Nucleus	transcription regulator	VH	-1.974	0.660	1.279	1.246	1.436	0.977
gi_149944716_ref_NM_001099115.1_	540904	SMARCA2	SWI/SNF related, matrix associated, actin dependent r	Nucleus	transcription regulator	VH	-1.972	0.672	1.608	0.438	1.528	0.772
gi_119911770_ref_XM_606825.3_	528403	ANKFY1	ankyrin repeat and FYVE domain containing 1	Nucleus	transcription regulator	H	-1.961	0.701	-1.010	1.021	1.488	0.850
gi_75832109_ref_NM_001014965.2_	540949	ANKRD49	ankyrin repeat domain 49	Nucleus	transcription regulator	H	-1.949	0.715	2.472	0.007	2.636	0.014
gi_119907162_ref_XM_605809.3_	527418	CBL	Cas-Br-M (murine) ecotropic retroviral transforming se	Nucleus	transcription regulator	VH	-1.947	0.702	1.182	1.182	1.707	0.435
gi_119877632_ref_XM_603300.3_	524960	NRIP1	nuclear receptor interacting protein 1	Nucleus	transcription regulator	VH	-1.942	0.739	1.369	0.936	1.888	0.231
gi_119905202_ref_XM_598095.3_	519864	MLLT10	myeloid/lymphoid or mixed-lineage leukemia (trithorax	Nucleus	transcription regulator	VH	-1.927	0.711	1.856	0.133	1.879	0.233
gi_119924137_ref_XM_609443.3_	530961	BARD1	BRCA1 associated RING domain 1	Nucleus	transcription regulator	H	-1.925	0.652	1.393	0.903	-1.015	1.042
gi_119911512_ref_XM_866591.2_	536067	TAF15	TAF15 RNA polymerase II, TATA box binding protein (T	Nucleus	transcription regulator	H	-1.920	0.766	1.421	0.491	1.364	1.177
gi_31340639_ref_NM_174722.2_	282707	CEP290	centrosomal protein 290kDa	Cytoplasm	transcription regulator	H	-1.865	0.841	2.017	0.059	1.555	0.703
gi_66792815_ref_NM_001024511.1_	510184	TADA3	transcriptional adaptor 3	Nucleus	transcription regulator	VH	-1.864	0.821	1.446	0.787	1.733	0.393
gi_119911162_ref_XM_872583.2_	523865	MZF1	myeloid zinc finger 1	Nucleus	transcription regulator	H	-1.863	0.834	-1.451	0.059	1.595	0.629
gi_149642932_ref_NM_001099107.1_	540378	MDM2	Mdm2 p53 binding protein homolog (mouse)	Nucleus	transcription regulator	VH	-1.850	0.891	1.059	1.144	1.163	1.330
gi_32189343_ref_NM_173993.1_	281010	ARNT	aryl hydrocarbon receptor nuclear translocator	Nucleus	transcription regulator	H	-1.847	0.833	1.506	0.656	1.672	0.488
gi_119924356_ref_XM_589474.3_	512036	ZNF449	zinc finger protein 449	Nucleus	transcription regulator	H	-1.838	0.871	1.987	0.072	1.241	1.342
gi_116003910_ref_NM_001076848.1_	514085	E2F6	E2F transcription factor 6	Nucleus	transcription regulator	VH	-1.838	0.875	1.319	1.147	1.294	1.308
gi_119909509_ref_XM_001252556.1_	532416	PATZ1	POZ (BTB) and AT hook containing zinc finger 1	Nucleus	transcription regulator	H	-1.834	0.839	1.588	0.456	2.365	0.038
gi_31342886_ref_NM_174080.2_	281225	UBE2K	ubiquitin-conjugating enzyme E2K (UBC1 homolog, ye	Cytoplasm	transcription regulator	VH	-1.832	0.905	1.219	1.253	1.149	1.332
gi_119902367_ref_XM_869255.2_	532556	MGA	MAX gene associated	Nucleus	transcription regulator	VH	-1.823	0.895	1.627	0.351	1.751	0.372
gi_119908981_ref_XM_001250053.1_	613439	ELF2	E74-like factor 2 (ets domain transcription factor)	Nucleus	transcription regulator	H	-1.811	0.946	2.071	0.044	1.780	0.335
gi_118150879_ref_NM_001077885.1_	509039	TCF12	transcription factor 12	Nucleus	transcription regulator	VH	-1.810	0.934	1.609	0.456	1.866	0.251
gi_114053148_ref_NM_001046556.1_	615211	MTF2	metal response element binding transcription factor 2	Nucleus	transcription regulator	VH	-1.808	0.934	1.093	1.206	1.013	1.154
gi_77735776_ref_NM_001034411.1_	511795	ZSCAN16	zinc finger and SCAN domain containing 16	Nucleus	transcription regulator	H	-1.802	0.932	1.284	1.242	-1.130	0.689
gi_157279880_ref_NM_001104986.1_	530184	SSBP3	single stranded DNA binding protein 3	Nucleus	transcription regulator	VH	-1.800	0.951	1.140	1.244	1.214	1.343
gi_156718125_ref_NM_001103098.1_	530411	UHRF1	ubiquitin-like with PHD and ring finger domains 1	Nucleus	transcription regulator	VH	-1.792	0.934	-1.176	0.561	-1.142	0.679
gi_119914156_ref_XM_582981.3_	506525	RCOR1	REST corepressor 1	Nucleus	transcription regulator	VH	-1.789	0.945	1.137	1.209	2.163	0.079
gi_119892216_ref_XM_869350.2_	509843	HDAC7	histone deacetylase 7	Nucleus	transcription regulator	H	-1.785	0.939	-1.166	0.570	1.379	1.132
gi_155372158_ref_NM_001101219.1_	541180	FEM1C	fem-1 homolog c (C. elegans)	Nucleus	transcription regulator	VH	-1.784	0.943	1.487	0.705	1.497	0.837
gi_73853751_ref_NM_174201.2_	281542	TP53	tumor protein p53	Nucleus	transcription regulator	VH	-1.777	0.969	1.411	0.906	1.510	0.802
gi_115497281_ref_NM_001075637.1_	513719	HMG20A	high-mobility group 20A	Nucleus	transcription regulator	VH	-1.755	1.013	1.773	0.219	1.735	0.396
gi_119906688_ref_XM_601083.3_	522795	KLF10	Kruppel-like factor 10	Nucleus	transcription regulator	H	-1.750	1.019	2.065	0.047	3.260	0.003
gi_119902916_ref_XM_879035.2_	531476	JDP2	Jun dimerization protein 2	Nucleus	transcription regulator	H	-1.735	1.023	-1.334	0.148	-1.020	1.046
gi_114052638_ref_NM_001045892.1_	504613	MED24	mediator complex subunit 24	Nucleus	transcription regulator	H	-1.731	1.001	-1.016	1.033	1.357	1.167
gi_119905729_ref_XM_600851.3_	522566	PLAGL2	pleiomorphic adenoma gene-like 2	Nucleus	transcription regulator	H	-1.728	1.036	-1.568	0.019	-1.204	0.475
gi_119905921_ref_XM_589971.3_	512451	ZHX3	zinc fingers and homeoboxes 3	Nucleus	transcription regulator	H	-1.723	1.034	-1.334	0.185	-1.091	0.797
gi_119895922_ref_XM_869174.2_	617002	FAM172A	family with sequence similarity 172, member A	unknown	transcription regulator	VH	-1.721	1.027	1.407	0.885	1.446	0.953
gi_125991919_ref_NM_001081612.1_	540309	ELK4	ELK4, ETS-domain protein (SRF accessory protein 1)	Nucleus	transcription regulator	VH	-1.720	1.049	-1.532	0.010	1.223	1.340
gi_73808098_ref_NM_001032294.1_	509072	ALS2CR8	amyotrophic lateral sclerosis 2 (juvenile) chromosome	Nucleus	transcription regulator	H	-1.715	1.052	1.919	0.100	1.757	0.356
gi_119917787_ref_XM_611661.3_	540481	TCF7L2 (includes	transcription factor 7-like 2 (T-cell specific, HMG-box)	Nucleus	transcription regulator	VH	-1.703	1.071	1.291	1.205	1.686	0.458
gi_149642584_ref_NM_001099113.1_	540816	ZNF189	zinc finger protein 189	Nucleus	transcription regulator	H	-1.696	1.109	1.318	1.159	1.209	1.343
gi_115495392_ref_NM_001075804.1_	520313	GABPB1 (include:	GA binding protein transcription factor, beta subunit 1	Nucleus	transcription regulator	H	-1.691	1.132	1.235	1.257	-1.089	0.799
gi_119887568_ref_XM_613036.3_	540766	SP3	Sp3 transcription factor	Nucleus	transcription regulator	VH	-1.691	1.119	1.343	1.124	1.699	0.444
gi_114052399_ref_NM_001045908.1_	505051	TAF1A	TATA box binding protein (TBP)-associated factor, RNA	Nucleus	transcription regulator	H	-1.686	1.078	2.043	0.053	1.413	1.017
gi_78369291_ref_NM_001035368.1_	527103	ZSCAN21	zinc finger and SCAN domain containing 21	Nucleus	transcription regulator	H	-1.685	1.123	1.961	0.082	1.800	0.307
gi_119892260_ref_XM_596055.3_	517876	ARID2	AT rich interactive domain 2 (ARID, RFX-like)	Nucleus	transcription regulator	VH	-1.685	1.134	1.500	0.693	1.396	1.103
gi_114052331_ref_NM_001046351.1_	533430	TRIP4	thyroid hormone receptor interactor 4	Nucleus	transcription regulator	VH	-1.682	1.145	1.684	0.325	1.566	0.703
gi_119893929_ref_XM_867691.2_	615792	REST	RE1-silencing transcription factor	Nucleus	transcription regulator	H	-1.680	1.048	2.139	0.032	1.426	0.911
gi_119892032_ref_XM_615033.3_	541204	ATF7	activating transcription factor 7	Nucleus	transcription regulator	VH	-1.671	1.086	1.401	0.710	1.892	0.218
gi_155372120_ref_NM_001101200.1_	539938	TAF8	TAF8 RNA polymerase II, TATA box binding protein (TE	Nucleus	transcription regulator	VH	-1.666	1.166	1.548	0.580	1.567	0.688
gi_118151399_ref_NM_001077960.1_	524111	ZNF227	zinc finger protein 227	Nucleus	transcription regulator	VH	-1.666	1.144	1.122	1.218	1.089	1.264
gi_77736579_ref_NM_001034801.1_	617530	CEBPG	CCAAT/enhancer binding protein (C/EBP), gamma	Nucleus	transcription regulator	VH	-1.661	1.150	1.009	1.102	1.097	1.296
gi_148356239_ref_NM_001098377.1_	506056	EID1	EP300 interacting inhibitor of differentiation 1	Nucleus	transcription regulator	VH	-1.659	1.170	1.452	0.819	1.347	1.212
gi_115496659_ref_NM_001075329.1_	507729	TCEA2	transcription elongation factor A (SII), 2	Nucleus	transcription regulator	H	-1.656	1.107	1.891	0.108	2.229	0.063
gi_119888072_ref_XM_001250304.1_	521089	CCNT2	cyclin T2	Nucleus	transcription regulator	H	-1.651	1.181	1.520	0.632	1.498	0.856
gi_119901461_ref_XM_581379.3_	505138	CASP8AP2	caspase 8 associated protein 2	Nucleus	transcription regulator	VH	-1.651	1.170	1.354	1.070	1.387	1.126
gi_165377267_ref_NM_001113531.1_	504463	SIRT2	sirtuin (silent mating type information regulation 2 hom	Nucleus	transcription regulator	H	-1.642	1.158	1.689	0.310	1.751	0.371
gi_157074093_ref_NM_001103289.1_	539761	PLAGL1	pleiomorphic adenoma gene-like 1	Nucleus	transcription regulator	VH	-1.642	1.184	1.912	0.104	2.151	0.083
gi_118151061_ref_NM_001077983.1_	529698	INVS	inversin	Nucleus	transcription regulator	H	-1.632	1.182	1.820	0.171	2.282	0.051
gi_116004080_ref_NM_001076928.1_	540806	SMAD9	SMAD family member 9	Nucleus	transcription regulator	VH	-1.631	1.200	1.392	0.973	1.514	0.814
gi_119906083_ref_XM_613264.3_	533757	ADNP	activity-dependent neuroprotector homeobox	Nucleus	transcription regulator	VH	-1.630	1.204	1.536	0.608	1.823	0.288
gi_156120812_ref_NM_001102083.1_	524585	TTF1	transcription termination factor, RNA polymerase I	Nucleus	transcription regulator	H	-1.624	1.183	1.763	0.216	1.483	0.870
gi_114052996_ref_NM_001046237.1_	518176	ZNF323	zinc finger protein 323	Nucleus	transcription regulator	H	-1.623	1.189	2.297	0.015	2.129	0.093

gi_119901261_ref_XM_596663.3_	518472	GTF3C6	general transcription factor IIIC, polypeptide 6, alpha 35	unknown	transcription regulator	H	-1.618	1.208	1.424	0.893	1.006	1.110
gi_119918864_ref_XM_864539.2_	541248	RSF1	remodeling and spacing factor 1	Nucleus	transcription regulator	H	-1.613	1.227	1.552	0.514	1.707	0.438
gi_119894165_ref_XM_001249488.1_	781149	AFF1	AF4/FMR2 family, member 1	Nucleus	transcription regulator	H	-1.611	1.193	-1.002	1.049	1.110	1.238
gi_119905647_ref_XR_028807.1_	789854	TAF4	TAF4 RNA polymerase II, TATA box binding protein (TB	Nucleus	transcription regulator	H	-1.610	1.237	1.579	0.509	1.803	0.315
gi_119927108_ref_XM_600466.3_	522191	MTERF	mitochondrial transcription termination factor	Cytoplasm	transcription regulator	H	-1.608	1.232	1.460	0.790	1.129	1.276
gi_114052738_ref_NM_001046298.1_	529124	FLI1	Friend leukemia virus integration 1	Nucleus	transcription regulator	VH	-1.605	1.209	1.102	1.196	1.093	1.246
gi_157427973_ref_NM_001105424.1_	539542	HHEX	hematopoietically expressed homeobox	Nucleus	transcription regulator	H	-1.603	1.180	-1.550	0.024	1.668	0.466
gi_47564039_ref_NM_001001147.1_	407194	CCNT1	cyclin T1	Nucleus	transcription regulator	H	-1.596	1.182	1.023	1.021	1.101	1.236
gi_157279924_ref_NM_001105008.1_	618133	SUPT3H (includes	suppressor of Ty 3 homolog (S. cerevisiae)	Nucleus	transcription regulator	H	-1.590	1.232	1.598	0.443	1.297	1.243
gi_119901004_ref_XM_598421.3_	520186	CEP110	centrosomal protein 110kDa	Nucleus	transcription regulator	VH	-1.583	1.239	1.193	1.252	1.088	1.276
gi_164414432_ref_NM_176635.3_	338034	MYBL1	v-myb myeloblastosis viral oncogene homolog (avian)-	Nucleus	transcription regulator	H	-1.582	1.244	1.719	0.271	1.064	1.256
gi_62751780_ref_NM_001015654.1_	534403	SP2	Sp2 transcription factor	Nucleus	transcription regulator	H	-1.572	1.262	1.324	1.153	1.263	1.340
gi_119875275_ref_XM_609842.3_	531350	BACH1	BTB and CNC homology 1, basic leucine zipper transc	Nucleus	transcription regulator	H	-1.569	1.255	1.172	1.248	1.625	0.566
gi_119901876_ref_XM_606924.3_	528498	JMY	junction mediating and regulatory protein, p53 cofactor	Nucleus	transcription regulator	H	-1.563	1.288	1.725	0.247	1.819	0.292
gi_156120954_ref_NM_001102154.1_	533243	ZNF143	zinc finger protein 143	Nucleus	transcription regulator	VH	-1.562	1.293	1.281	1.241	1.129	1.325
gi_119883223_ref_XM_865825.2_	614346	TBL1XR1	transducin (beta)-like 1 X-linked receptor 1	Nucleus	transcription regulator	VH	-1.559	1.293	1.302	1.220	1.083	1.264
gi_119926771_ref_XM_584325.3_	507668	LCOR	ligand dependent nuclear receptor corepressor	Nucleus	transcription regulator	H	-1.557	1.265	1.090	0.826	1.177	1.319
gi_115497767_ref_NM_001075224.1_	505818	PPP1R13L	protein phosphatase 1, regulatory (inhibitor) subunit 13	Nucleus	transcription regulator	H	-1.555	1.276	-1.008	1.025	1.211	1.343
gi_115497323_ref_NM_001075265.1_	506485	PBXIP1	pre-B-cell leukemia homeobox interacting protein 1	Nucleus	transcription regulator	H	-1.546	1.300	1.161	1.252	2.097	0.104
gi_119912578_ref_XM_001249745.1_	532986	BPTF	bromodomain PHD finger transcription factor	Nucleus	transcription regulator	VH	-1.544	1.266	1.432	0.814	1.636	0.548
gi_119890380_ref_XM_879774.2_	512622	KDM4A	lysine (K)-specific demethylase 4A	Nucleus	transcription regulator	H	-1.542	1.290	1.264	1.253	1.788	0.321
gi_119893251_ref_XM_585886.3_	509011	MKL1	megakaryoblastic leukemia (translocation) 1	Nucleus	transcription regulator	VH	-1.542	1.300	-1.171	0.520	1.483	0.890
gi_119910578_ref_XM_001256446.1_	789798	ERF	Ets2 repressor factor	Nucleus	transcription regulator	H	-1.537	1.212	-1.189	0.151	2.448	0.028
gi_155372102_ref_NM_001101190.1_	539320	ZSCAN2	zinc finger and SCAN domain containing 2	Nucleus	transcription regulator	H	-1.530	1.280	1.239	1.256	1.312	1.255
gi_78050074_ref_NM_001035057.1_	508445	HCLS1	hematopoietic cell-specific Lyn substrate 1	Nucleus	transcription regulator	VH	-1.528	1.299	1.370	1.027	1.449	0.949
gi_115495820_ref_NM_001076249.1_	540932	FBXW7	F-box and WD repeat domain containing 7	Nucleus	transcription regulator	H	-1.522	1.313	-1.366	0.133	-1.231	0.382
gi_114051342_ref_NM_001046143.1_	513265	CDKN2AIP	CDKN2A interacting protein	Nucleus	transcription regulator	H	-1.516	1.318	1.103	1.194	-1.040	0.989
gi_114051613_ref_NM_001046519.1_	613828	DR1	down-regulator of transcription 1, TBP-binding (negativ	Nucleus	transcription regulator	VH	-1.515	1.332	1.344	1.121	-1.112	0.736
gi_158819092_ref_NM_001110181.1_	518857	ERCC8	excision repair cross-complementing rodent repair defi	Nucleus	transcription regulator	H	-1.513	1.324	1.324	1.129	-1.099	0.783
gi_119884867_ref_XM_610719.3_	532209	MECOM	MDS1 and EVI1 complex locus	Nucleus	transcription regulator	H	-1.508	1.324	1.871	0.132	1.847	0.271
gi_115495874_ref_NM_001075460.1_	510654	HDAC10	histone deacetylase 10	Nucleus	transcription regulator	H	-1.498	1.317	-1.446	0.057	1.258	1.340
gi_119914492_ref_XM_868895.2_	616786	TMF1	TATA element modulatory factor 1	Cytoplasm	transcription regulator	H	-1.493	1.347	1.098	1.200	-1.117	0.728
gi_76634364_ref_XM_585648.2_	539210	PLAG1	pleiomorphic adenoma gene 1	Nucleus	transcription regulator	H	-1.491	1.332	1.457	0.789	1.303	1.296
gi_84000084_ref_NM_001038054.1_	507723	HMG20B	high-mobility group 20B	Nucleus	transcription regulator	VH	-1.488	1.347	-1.081	0.896	1.230	1.342
gi_115495314_ref_NM_001076290.1_	613818	TBPL1	TBP-like 1	Nucleus	transcription regulator	VH	-1.488	1.355	1.070	1.187	-1.058	0.962
gi_94966798_ref_NM_001040494.1_	404183	EED	embryonic ectoderm development	Nucleus	transcription regulator	VH	-1.487	1.356	1.314	1.199	1.167	1.333
gi_119916696_ref_XM_867976.2_	528545	ANKS3	ankyrin repeat and sterile alpha motif domain containin	Nucleus	transcription regulator	H	-1.477	1.358	-2.095	0.000	-2.156	0.000
gi_119910150_ref_XM_614673.3_	541127	NFATC3	nuclear factor of activated T-cells, cytoplasmic, calcine	Nucleus	transcription regulator	H	-1.470	1.355	1.264	1.253	1.690	0.462
gi_119911784_ref_XM_582242.3_	505878	SOX15	SRY (sex determining region Y)-box 15	Nucleus	transcription regulator	H	-1.468	1.356	1.031	1.116	1.062	1.232
gi_118151147_ref_NM_001078028.1_	540824	BCL10	B-cell CLL/lymphoma 10	Cytoplasm	transcription regulator	H	-1.461	1.357	1.290	1.170	1.034	1.066
gi_156121092_ref_NM_001102223.1_	539013	CDYL	chromodomain protein, Y-like	Nucleus	transcription regulator	H	-1.461	1.358	1.137	1.215	1.126	1.314
gi_82697318_ref_NM_001037448.1_	506667	LOC642521	similar to nuclear DNA-binding protein	unknown	transcription regulator	H	-1.461	1.363	1.073	1.177	1.111	1.277
gi_119903867_ref_XM_867432.2_	525346	NCOA1	nuclear receptor coactivator 1	Nucleus	transcription regulator	H	-1.461	1.351	1.787	0.188	2.211	0.065
gi_119904828_ref_XM_001249546.1_	781157	HTATSF1	HIV-1 Tat specific factor 1	Nucleus	transcription regulator	VH	-1.460	1.373	1.232	1.248	1.150	1.332
gi_119892443_ref_XM_876015.2_	509799	BAZ2A	bromodomain adjacent to zinc finger domain, 2A	Nucleus	transcription regulator	VH	-1.458	1.368	-1.018	1.023	1.573	0.686
gi_149642726_ref_NM_001099174.1_	618214	HOXB3	homeobox B3	Nucleus	transcription regulator	H	-1.455	1.368	1.263	1.253	1.507	0.826
gi_77735978_ref_NM_001034516.1_	516078	SNAPC2	small nuclear RNA activating complex, polypeptide 2, 4	Nucleus	transcription regulator	H	-1.454	1.364	-1.455	0.048	-1.217	0.420
gi_119916711_ref_XM_604254.3_	525895	UBN1	ubinuclein 1	Nucleus	transcription regulator	VH	-1.450	1.378	-1.311	0.185	1.126	1.327
gi_156718109_ref_NM_001103090.1_	508384	GATAD2A	GATA zinc finger domain containing 2A	Nucleus	transcription regulator	VH	-1.447	1.370	-1.130	0.710	1.149	1.328
gi_115497357_ref_NM_001075262.1_	506448	ZNF397	zinc finger protein 397	Nucleus	transcription regulator	H	-1.445	1.379	1.448	0.847	1.250	1.334
gi_119904245_ref_XM_592658.3_	540249	GTF3C4	general transcription factor IIIC, polypeptide 4, 90kDa	Nucleus	transcription regulator	H	-1.436	1.364	1.195	1.218	1.530	0.731
gi_115494991_ref_NM_001075396.1_	509231	PIAS1	protein inhibitor of activated STAT, 1	Nucleus	transcription regulator	VH	-1.434	1.380	1.213	1.256	1.285	1.326
gi_119889209_ref_XM_612098.3_	540563	ASH1L	ash1 (absent, small, or homeotic)-like (Drosophila)	Nucleus	transcription regulator	H	-1.431	1.373	1.334	1.062	1.813	0.301
gi_119903894_ref_XM_868832.2_	616730	KLF11	Kruppel-like factor 11	Nucleus	transcription regulator	H	-1.430	1.386	1.936	0.095	2.482	0.024
gi_119911470_ref_XM_603205.3_	524868	MED13	mediator complex subunit 13	Nucleus	transcription regulator	VH	-1.426	1.389	1.139	1.238	1.476	0.920
gi_84370168_ref_NM_001038566.1_	539496	MED21	mediator complex subunit 21	Nucleus	transcription regulator	VH	-1.424	1.389	1.026	1.118	-1.053	0.989
gi_166157495_ref_NM_001113767.1_	533620	ANKRD26	ankyrin repeat domain 26	Nucleus	transcription regulator	H	-1.423	1.378	1.895	0.110	1.500	0.817
gi_119918081_ref_XM_592435.3_	337916	IRF2	interferon regulatory factor 2	Nucleus	transcription regulator	VH	-1.423	1.388	1.546	0.594	1.664	0.502
gi_119911986_ref_XM_866711.2_	507663	NCOR1	nuclear receptor co-repressor 1	Nucleus	transcription regulator	VH	-1.422	1.379	1.098	1.194	1.323	1.234
gi_119910346_ref_XM_001253667.1_	785741	ZBTB32	zinc finger and BTB domain containing 32	Nucleus	transcription regulator	H	-1.416	1.359	4.011	0.000	6.205	0.000
gi_119905251_ref_XM_864298.2_	613475	MSRB2	methionine sulfoxide reductase B2	Nucleus	transcription regulator	VH	-1.416	1.388	3.091	0.000	2.561	0.019
gi_114051208_ref_NM_001046581.1_	616539	CHURC1	churchill domain containing 1	Nucleus	transcription regulator	H	-1.414	1.388	1.371	1.041	1.033	1.173
gi_119892694_ref_XM_864033.2_	505341	HCFC2	host cell factor C2	Nucleus	transcription regulator	H	-1.413	1.389	1.359	1.044	1.338	1.213
gi_34147042_ref_NM_183082.1_	360190	AIP	aryl hydrocarbon receptor interacting protein	Nucleus	transcription regulator	H	-1.410	1.389	1.173	1.255	1.474	0.898
gi_119901604_ref_XM_594787.3_	516630	SHPRH	SNF2 histone linker PHD RING helicase	Nucleus	transcription regulator	H	-1.408	1.389	2.226	0.022	2.066	0.116
gi_77736452_ref_NM_001034754.1_	614227	TAF12 (includes E	TAF12 RNA polymerase II, TATA box binding protein (T	Nucleus	transcription regulator	H	-1.407	1.391	1.238	1.256	-1.344	0.184
gi_118151227_ref_NM_001078074.1_	617345	VAV1	vav 1 guanine nucleotide exchange factor	Nucleus	transcription regulator	VH	-1.406	1.387	1.300	1.200	1.401	1.065
gi_119331201_ref_NM_001079786.1_	614382	NFYB	nuclear transcription factor Y, beta	Nucleus	transcription regulator	H	-1.405	1.390	1.309	1.201	1.163	1.315
gi_149642624_ref_NM_001098980.1_	515219	MAFG	v-maf musculoaponeurotic fibrosarcoma oncogene hor	Nucleus	transcription regulator	H	-1.403	1.389	1.116	1.229	1.272	1.335
gi_114051673_ref_NM_001046435.1_	538675	CALCOCO1	calcium binding and coiled-coil domain 1	Nucleus	transcription regulator	H	-1.401	1.389	2.751	0.002	4.896	0.000
gi_119916360_ref_XM_612798.3_	533403	PIAS2	protein inhibitor of activated STAT, 2	Nucleus	transcription regulator	H	-1.401	1.387	1.531	0.589	1.457	0.915
gi_119904714_ref_XM_864451.2_	613543	CENPJ	centromere protein J	Nucleus	transcription regulator	H	-1.399	1.388	1.547	0.539	1.137	1.310
gi_155371844_ref_NM_001101057.1_	100137723	GTF2H4	general transcription factor IIH, polypeptide 4, 52kDa	Nucleus	transcription regulator	H	-1.398	1.389	1.157	1.234	-1.171	0.574
gi_27805856_ref_NM_174285.1_	281713	CREB1	cAMP responsive element binding protein 1	Nucleus	transcription regulator	VH	-1.396	1.388	1.366	1.032	1.141	1.328

gi_115497737_ref_NM_001075597.1_	513065	TAF13	TAF13 RNA polymerase II, TATA box binding protein (T	Nucleus	transcription regulator	H	-1.392	1.384	1.232	1.254	-1.042	0.939
gi_119915832_ref_XM_606667.3_	528253	JARID2	jumonji, AT rich interactive domain 2	Nucleus	transcription regulator	VH	-1.391	1.387	1.345	1.063	1.890	0.221
gi_119915606_ref_XM_001252983.1_	785479	HMGN1	high-mobility group nucleosome binding domain 1	Nucleus	transcription regulator	VH	-1.389	1.340	1.070	0.837	-1.279	0.180
gi_119892486_ref_XM_599314.3_	521057	ANKRD52	ankyrin repeat domain 52	Nucleus	transcription regulator	H	-1.387	1.384	-1.975	0.002	1.038	1.161
gi_157427817_ref_NM_001105346.1_	508905	ZRANB2	zinc finger, RAN-binding domain containing 2	Nucleus	transcription regulator	H	-1.384	1.390	1.371	1.062	1.238	1.340
gi_119902710_ref_XM_864346.2_	531515	ARID4A	AT rich interactive domain 4A (RBP1-like)	Nucleus	transcription regulator	VH	-1.382	1.388	1.464	0.787	1.567	0.693
gi_119919719_ref_XM_590977.3_	540023	BCORL1	BCL6 co-repressor-like 1	Nucleus	transcription regulator	H	-1.379	1.380	-1.298	0.201	1.169	1.314
gi_134085960_ref_NM_001083405.1_	510557	HSF2	heat shock transcription factor 2	Nucleus	transcription regulator	VH	-1.376	1.390	1.424	0.905	1.158	1.324
gi_139949221_ref_NM_001083746.1_	539836	ZNF689	zinc finger protein 689	Nucleus	transcription regulator	H	-1.373	1.389	1.229	1.256	1.273	1.325
gi_164519011_ref_NM_001113316.1_	767979	HSBP1	heat shock factor binding protein 1	Nucleus	transcription regulator	VH	-1.373	1.384	1.097	1.193	1.078	1.272
gi_62751509_ref_NM_001015597.1_	514369	NFKBIL1	nuclear factor of kappa light polypeptide gene enhance	Nucleus	transcription regulator	H	-1.366	1.387	1.050	1.153	1.419	1.041
gi_78369237_ref_NM_001035305.1_	515119	C19ORF2	chromosome 19 open reading frame 2	Nucleus	transcription regulator	VH	-1.366	1.387	1.336	1.152	1.220	1.341
gi_119916689_ref_XM_581740.3_	505453	CREBBP	CREB binding protein	Nucleus	transcription regulator	VH	-1.361	1.377	-1.159	0.381	1.843	0.263
gi_119903730_ref_XM_001254141.1_	786476	SERTAD2	SERTA domain containing 2	Cytoplasm	transcription regulator	VH	-1.360	1.373	-1.080	0.738	1.192	1.330
gi_155372186_ref_NM_001101234.1_	614773	ZFP36L1	zinc finger protein 36, C3H type-like 1	Nucleus	transcription regulator	VH	-1.360	1.381	1.087	1.186	1.088	1.277
gi_119919942_ref_XM_588477.3_	539629	MECP2	methyl CpG binding protein 2 (Rett syndrome)	Nucleus	transcription regulator	VH	-1.359	1.385	1.039	1.146	1.352	1.192
gi_119915671_ref_XM_001254052.1_	786347	ZNRD1	zinc ribbon domain containing 1	Nucleus	transcription regulator	H	-1.358	1.353	-1.055	0.746	-1.745	0.005
gi_153792238_ref_NM_001099717.1_	540945	DEK	DEK oncogene	Nucleus	transcription regulator	VH	-1.357	1.372	1.337	1.132	1.240	1.343
gi_139949089_ref_NM_001083687.1_	515444	ACAP3	ArfGAP with coiled-coil, ankyrin repeat and PH domain	Nucleus	transcription regulator	H	-1.356	1.374	-1.933	0.001	1.001	1.038
gi_119892720_ref_XM_612859.3_	540731	PRDM4	PR domain containing 4	Nucleus	transcription regulator	H	-1.356	1.381	1.285	1.243	1.426	1.036
gi_115495742_ref_NM_001076256.1_	541116	MXD4	MAX dimerization protein 4	Nucleus	transcription regulator	H	-1.353	1.383	1.694	0.293	2.966	0.006
gi_82697380_ref_NM_001037479.1_	536936	SUV39H2	suppressor of variegation 3-9 homolog 2 (Drosophila)	Nucleus	transcription regulator	H	-1.350	1.385	1.437	0.881	-1.093	0.842
gi_115497065_ref_NM_001075289.1_	506967	ATF1	activating transcription factor 1	Nucleus	transcription regulator	H	-1.342	1.376	1.148	1.242	-1.129	0.673
gi_119918744_ref_XM_601197.3_	522908	ERCC6	excision repair cross-complementing rodent repair defi	Nucleus	transcription regulator	H	-1.342	1.375	1.938	0.094	2.014	0.142
gi_31340799_ref_NM_174732.2_	282848	PFDN5	prefoldin subunit 5	Nucleus	transcription regulator	H	-1.342	1.378	-1.002	1.057	1.225	1.343
gi_119918600_ref_XM_867653.2_	535403	MYST4	MYST histone acetyltransferase (monocytic leukemia)	Nucleus	transcription regulator	H	-1.340	1.353	1.625	0.385	2.237	0.059
gi_157041263_ref_NM_001103174.1_	540661	BRWD1	bromodomain and WD repeat domain containing 1	Nucleus	transcription regulator	VH	-1.337	1.380	1.288	1.191	1.733	0.389
gi_115495362_ref_NM_001076287.1_	613751	GPBP1	GC-rich promoter binding protein 1	Nucleus	transcription regulator	VH	-1.331	1.377	1.111	1.234	1.143	1.330
gi_118601757_ref_NM_001079583.1_	509729	MED7 (includes E	mediator complex subunit 7	Nucleus	transcription regulator	H	-1.329	1.381	1.213	1.257	1.014	1.150
gi_78042559_ref_NM_001035106.1_	512411	ZNF174	zinc finger protein 174	Nucleus	transcription regulator	H	-1.325	1.372	1.426	0.859	1.353	1.153
gi_119895491_ref_XM_001251355.1_	782746	PURA	purine-rich element binding protein A	Nucleus	transcription regulator	H	-1.324	1.373	1.251	1.252	1.434	1.004
gi_119892451_ref_XM_588270.3_	511023	STAT2	signal transducer and activator of transcription 2, 113kI	Nucleus	transcription regulator	H	-1.324	1.360	1.520	0.629	2.078	0.112
gi_119920644_ref_XM_593202.3_	515220	MSL3	male-specific lethal 3 homolog (Drosophila)	Nucleus	transcription regulator	VH	-1.324	1.371	1.211	1.258	1.473	0.925
gi_119900789_ref_XM_613307.3_	540823	ZNF367	zinc finger protein 367	Nucleus	transcription regulator	H	-1.323	1.376	-1.076	0.918	-1.321	0.221
gi_114052580_ref_NM_001045895.1_	504662	ZNHIT3	zinc finger, HIT type 3	Nucleus	transcription regulator	H	-1.323	1.373	1.132	1.237	1.040	1.223
gi_119908237_ref_XM_580880.3_	504716	AHCTF1	AT hook containing transcription factor 1	Nucleus	transcription regulator	H	-1.322	1.345	1.210	1.248	-1.002	1.052
gi_119916928_ref_XM_611393.3_	511420	SRCAP	Snf2-related CREBBP activator protein	Cytoplasm	transcription regulator	H	-1.321	1.349	-1.123	0.497	1.664	0.493
gi_119895763_ref_XM_612851.3_	533443	CNOT8	CCR4-NOT transcription complex, subunit 8	Nucleus	transcription regulator	VH	-1.321	1.371	1.326	1.176	1.084	1.296
gi_115495326_ref_NM_001075503.1_	511236	TAF1B	TATA box binding protein (TBP)-associated factor, RNA	Nucleus	transcription regulator	H	-1.315	1.366	1.037	1.157	-1.163	0.631
gi_119928183_ref_XR_027431.1_	539883	PTTG1	pituitary tumor-transforming 1	Nucleus	transcription regulator	H	-1.313	1.135	1.072	0.725	-1.707	0.005
gi_116004030_ref_NM_001076907.1_	534712	RB1	retinoblastoma 1	Nucleus	transcription regulator	H	-1.311	1.365	1.244	1.251	1.380	1.123
gi_119903526_ref_XM_864560.2_	509135	REL	v-rel reticuloendotheliosis viral oncogene homolog (avi	Nucleus	transcription regulator	H	-1.310	1.367	1.504	0.645	1.327	1.234
gi_119915226_ref_XM_587114.3_	510032	TFEB	transcription factor EB	Nucleus	transcription regulator	H	-1.310	1.339	-1.000	1.040	1.462	0.919
gi_115496122_ref_NM_001075748.1_	517097	CTCF	CCCTC-binding factor (zinc finger protein)	Nucleus	transcription regulator	VH	-1.308	1.371	1.342	1.129	1.206	1.343
gi_119892490_ref_XM_612597.3_	540671	IKZF4	IKAROS family zinc finger 4 (Eos)	Nucleus	transcription regulator	H	-1.305	1.335	-1.267	0.294	1.238	1.343
gi_119895244_ref_XM_883278.2_	533359	MXD3	MAX dimerization protein 3	Nucleus	transcription regulator	H	-1.303	1.335	-1.198	0.450	-1.360	0.148
gi_164448597_ref_NM_001035271.2_	505884	KLF6	Kruppel-like factor 6	Nucleus	transcription regulator	VH	-1.301	1.348	1.414	0.897	1.584	0.650
gi_119893850_ref_XM_001253114.1_	784953	NFXL1	nuclear transcription factor, X-box binding-like 1	Nucleus	transcription regulator	H	-1.298	1.358	1.568	0.534	1.285	1.327
gi_83649734_ref_NM_203362.2_	541099	MTPN	myotrophin	Nucleus	transcription regulator	VH	-1.298	1.364	1.146	1.248	1.022	1.207
gi_119918389_ref_XM_614988.3_	535018	TARBP1 (includes	TAR (HIV-1) RNA binding protein 1	Nucleus	transcription regulator	H	-1.296	1.334	1.205	1.254	1.333	1.245
gi_118151139_ref_NM_001078027.1_	540741	SP1	Sp1 transcription factor	Nucleus	transcription regulator	VH	-1.294	1.353	1.119	1.236	1.029	1.208
gi_31342416_ref_NM_174286.2_	281715	CREB3	cAMP responsive element binding protein 3	Nucleus	transcription regulator	H	-1.290	1.323	1.297	1.220	1.283	1.326
gi_119891422_ref_XM_617403.3_	537246	TRIM24	tripartite motif-containing 24	Nucleus	transcription regulator	VH	-1.287	1.345	1.288	1.233	1.051	1.224
gi_119915572_ref_XM_588389.3_	539620	ZKSCAN4	zinc finger with KRAB and SCAN domains 4	Nucleus	transcription regulator	H	-1.284	1.323	1.210	1.249	1.188	1.332
gi_31342081_ref_NM_174409.2_	281961	OSTF1	osteoclast stimulating factor 1	Nucleus	transcription regulator	VH	-1.284	1.353	1.035	1.151	1.007	1.178
gi_157427809_ref_NM_001105341.1_	507579	TTRAP	TRAF and TNF receptor associated protein	Cytoplasm	transcription regulator	H	-1.283	1.312	1.038	1.076	-1.408	0.093
gi_125630704_ref_NM_001081540.1_	540274	RNF14	ring finger protein 14	Nucleus	transcription regulator	VH	-1.281	1.321	1.209	1.257	1.283	1.325
gi_119905583_ref_XM_585061.3_	508301	GMEB2	glucocorticoid modulatory element binding protein 2	Nucleus	transcription regulator	H	-1.273	1.325	-1.328	0.176	1.058	1.251
gi_83035114_ref_NM_001037619.1_	613479	GTF2A2	general transcription factor IIA, 2, 12kDa	Nucleus	transcription regulator	VH	-1.264	1.305	1.173	1.246	-1.071	0.877
gi_118151003_ref_NM_001077955.1_	522836	ZNF394	zinc finger protein 394	Nucleus	transcription regulator	H	-1.260	1.320	1.025	1.096	-1.012	1.107
gi_157426912_ref_NM_001105257.1_	541154	IKZF1	IKAROS family zinc finger 1 (Ikaros)	Nucleus	transcription regulator	VH	-1.258	1.310	-1.210	0.459	1.057	1.258
gi_115495614_ref_NM_001075437.1_	510204	GABPA	GA binding protein transcription factor, alpha subunit 60	Nucleus	transcription regulator	VH	-1.257	1.304	1.062	1.160	-1.267	0.303
gi_155372126_ref_NM_001101203.1_	540090	RNF2	ring finger protein 2	Nucleus	transcription regulator	VH	-1.257	1.310	1.301	1.227	1.162	1.334
gi_119905771_ref_XM_615437.3_	535369	E2F1	E2F transcription factor 1	Nucleus	transcription regulator	H	-1.254	1.313	-1.577	0.017	-1.473	0.059
gi_78369499_ref_NM_001035307.1_	515380	EDF1	endothelial differentiation-related factor 1	Nucleus	transcription regulator	VH	-1.254	1.318	-1.028	1.024	-1.121	0.776
gi_119901393_ref_XM_615363.3_	541264	ZNF292	zinc finger protein 292	Nucleus	transcription regulator	VH	-1.250	1.308	1.146	1.238	1.524	0.771
gi_115496357_ref_NM_001076331.1_	614509	MXI1	MAX interactor 1	Nucleus	transcription regulator	H	-1.244	1.308	1.991	0.069	1.993	0.153
gi_119895221_ref_XM_588589.3_	511283	ARID3A	AT rich interactive domain 3A (BRIGHT-like)	Nucleus	transcription regulator	VH	-1.244	1.251	-1.659	0.009	-1.063	0.887
gi_118150917_ref_NM_001077904.1_	511407	NR2C1	nuclear receptor subfamily 2, group C, member 1	Nucleus	transcription regulator	VH	-1.244	1.314	1.273	1.244	1.218	1.342
gi_119895547_ref_XM_590199.3_	539896	BRD8	bromodomain containing 8	Nucleus	transcription regulator	VH	-1.234	1.272	1.277	1.250	1.216	1.342
gi_157785622_ref_NM_001105633.1_	540616	MAX	MYC associated factor X	Nucleus	transcription regulator	VH	-1.231	1.252	1.305	1.144	1.301	1.278
gi_155372156_ref_NM_001101218.1_	541143	ZNF131	zinc finger protein 131	Nucleus	transcription regulator	VH	-1.229	1.262	1.239	1.254	1.218	1.343
gi_119902124_ref_XM_592917.3_	514989	SUPT16H	suppressor of Ty 16 homolog (S. cerevisiae)	Nucleus	transcription regulator	VH	-1.221	1.253	1.199	1.255	-1.022	1.080
gi_119912222_ref_XM_581390.3_	538601	MLLT6	myeloid/lymphoid or mixed-lineage leukemia (trithorax	Nucleus	transcription regulator	H	-1.220	1.244	-2.412	0.000	1.197	1.338

gi_119906065_ref_XM_589621.3_	539807	ZNFX1	zinc finger, NFX1-type containing 1	Nucleus	transcription regulator	VH	-1.220	1.262	1.916	0.092	2.979	0.005
gi_119906434_ref_XM_588718.3_	511392	TGS1	trimethylguanosine synthase homolog (S. cerevisiae)	Nucleus	transcription regulator	VH	-1.219	1.231	-1.289	0.252	-1.390	0.118
gi_77736142_ref_NM_001034598.1_	534003	NFYC	nuclear transcription factor Y, gamma	Nucleus	transcription regulator	VH	-1.218	1.262	1.089	1.192	1.115	1.312
gi_62751582_ref_NM_001015582.1_	512789	BRF2	BRF2, subunit of RNA polymerase III transcription initia	Nucleus	transcription regulator	H	-1.215	1.282	-1.058	0.963	-1.062	0.964
gi_119909136_ref_XM_591880.3_	540159	ANKLE2	ankyrin repeat and LEM domain containing 2	Nucleus	transcription regulator	VH	-1.215	1.273	-1.021	1.049	1.055	1.248
gi_157785560_ref_NM_001105614.1_	414274	SMARCA4	SWI/SNF related, matrix associated, actin dependent r	Nucleus	transcription regulator	VH	-1.214	1.262	-1.289	0.262	-1.181	0.567
gi_119920411_ref_XM_001252291.1_	784529	BCOR	BCL6 co-repressor	Nucleus	transcription regulator	VH	-1.206	1.239	1.052	1.154	1.372	1.129
gi_119892198_ref_XM_583302.3_	506805	MLL2	myeloid/lymphoid or mixed-lineage leukemia 2	Nucleus	transcription regulator	H	-1.205	1.214	-1.930	0.004	1.927	0.194
gi_84000108_ref_NM_001038064.1_	509259	GTF2F2	general transcription factor IIF, polypeptide 2, 30kDa	Nucleus	transcription regulator	H	-1.203	1.225	1.250	1.251	-1.273	0.279
gi_115496329_ref_NM_001075824.1_	521975	TRIM32	tripartite motif-containing 32	Nucleus	transcription regulator	H	-1.202	1.229	1.305	1.173	1.068	1.233
gi_114051220_ref_NM_001046028.1_	509327	TAF7	TAF7 RNA polymerase II, TATA box binding protein (TE	Nucleus	transcription regulator	VH	-1.202	1.244	1.242	1.251	1.223	1.343
gi_119910227_ref_XM_877976.2_	538523	NFAT5	nuclear factor of activated T-cells 5, tonicity-responsive	Nucleus	transcription regulator	H	-1.200	1.071	1.922	0.066	2.842	0.008
gi_119908459_ref_XM_618469.3_	538270	MIB2	mindbomb homolog 2 (Drosophila)	Nucleus	transcription regulator	H	-1.198	1.173	-1.245	0.347	1.611	0.588
gi_119910191_ref_XM_866255.2_	614678	CBFB	core-binding factor, beta subunit	Nucleus	transcription regulator	VH	-1.198	1.251	-1.103	0.827	-1.138	0.707
gi_66792717_ref_NM_001024499.1_	508049	POLR3D	polymerase (RNA) III (DNA directed) polypeptide D, 44	Nucleus	transcription regulator	VH	-1.198	1.189	-1.103	0.781	-1.233	0.401
gi_114051068_ref_NM_001046493.1_	540859	TCEB1	transcription elongation factor B (SIII), polypeptide 1 (1	Nucleus	transcription regulator	VH	-1.198	1.184	-1.105	0.710	-1.491	0.046
gi_119908419_ref_XM_864814.2_	534608	ZBTB48	zinc finger and BTB domain containing 48	Nucleus	transcription regulator	H	-1.197	1.231	1.075	1.157	1.207	1.343
gi_115749623_ref_NM_001076181.2_	539696	HEXIM1	hexamethylene bis-acetamide inducible 1	Nucleus	transcription regulator	VH	-1.197	1.233	-1.006	1.075	1.001	1.129
gi_119920178_ref_XM_599229.3_	520974	MED12	mediator complex subunit 12	Nucleus	transcription regulator	VH	-1.197	1.156	-1.013	0.999	1.582	0.642
gi_119901950_ref_XM_592679.3_	540252	FEM1B	fem-1 homolog b (C. elegans)	Nucleus	transcription regulator	VH	-1.192	1.206	1.124	1.227	1.157	1.328
gi_118150783_ref_NM_001077834.1_	326578	SNW1	SNW domain containing 1	Nucleus	transcription regulator	VH	-1.188	1.204	1.151	1.245	1.080	1.273
gi_119916003_ref_XM_615234.3_	535214	ZNF236	zinc finger protein 236	Nucleus	transcription regulator	H	-1.183	1.209	-1.184	0.455	1.343	1.185
gi_119920196_ref_XM_580963.3_	504792	TAF1	TAF1 RNA polymerase II, TATA box binding protein (TE	Nucleus	transcription regulator	VH	-1.183	1.189	-1.002	1.052	1.200	1.341
gi_134085694_ref_NM_001083482.1_	534722	PIAS4	protein inhibitor of activated STAT, 4	Nucleus	transcription regulator	VH	-1.179	1.215	-1.194	0.532	1.049	1.244
gi_119893140_ref_XM_584665.3_	507962	KDM5A	lysine (K)-specific demethylase 5A	Nucleus	transcription regulator	VH	-1.176	1.201	-1.104	0.718	1.324	1.272
gi_119888429_ref_XM_872705.2_	511573	ZNF142	zinc finger protein 142	Nucleus	transcription regulator	H	-1.172	1.198	-1.205	0.352	1.039	1.214
gi_149642766_ref_NM_001099116.1_	540910	SMARCE1	SWI/SNF related, matrix associated, actin dependent r	Nucleus	transcription regulator	VH	-1.172	1.156	1.045	1.161	-1.039	1.021
gi_115749615_ref_NM_001035080.2_	509960	MTF1	metal-regulatory transcription factor 1	Nucleus	transcription regulator	VH	-1.171	1.203	-1.152	0.674	-1.178	0.587
gi_78369295_ref_NM_001035497.1_	617771	UBE2V1	ubiquitin-conjugating enzyme E2 variant 1	Nucleus	transcription regulator	VH	-1.170	1.187	1.155	1.245	1.084	1.288
gi_157428099_ref_NM_001105488.1_	100125917	RFXANK	regulatory factor X-associated ankyrin-containing prote	Nucleus	transcription regulator	VH	-1.168	1.172	1.214	1.254	1.421	1.022
gi_134085660_ref_NM_001083397.1_	509448	TFCP2	transcription factor CP2	Nucleus	transcription regulator	VH	-1.167	1.174	1.275	1.242	1.324	1.242
gi_119917332_ref_XM_871229.2_	618914	MAFK	v-maf musculoaponeurotic fibrosarcoma oncogene hor	Nucleus	transcription regulator	H	-1.166	1.184	-1.786	0.002	-1.772	0.005
gi_114052335_ref_NM_001046350.1_	533382	PKNOX1	PBX/knotted 1 homeobox 1	Nucleus	transcription regulator	H	-1.163	1.149	1.146	1.235	1.073	1.243
gi_119920764_ref_XM_605445.3_	527058	RLF	rearranged L-myc fusion	Nucleus	transcription regulator	H	-1.163	1.152	1.336	1.116	1.203	1.342
gi_119917839_ref_XM_866668.2_	614540	TIAL1	TIA1 cytotoxic granule-associated RNA binding protein	Nucleus	transcription regulator	VH	-1.163	1.193	1.131	1.247	-1.022	1.080
gi_153792752_ref_NM_001046074.2_	511077	MYC	v-myc myelocytomatosis viral oncogene homolog (avia	Nucleus	transcription regulator	VH	-1.162	1.175	1.301	1.230	1.138	1.332
gi_78369233_ref_NM_001035268.1_	505177	ZNF207	zinc finger protein 207	Nucleus	transcription regulator	VH	-1.162	1.150	1.211	1.257	1.113	1.304
gi_156120408_ref_NM_001101880.1_	507148	REST	RE1-silencing transcription factor	Nucleus	transcription regulator	H	-1.160	1.123	1.354	1.088	1.115	1.293
gi_119918095_ref_XM_874513.2_	404141	ING2	inhibitor of growth family, member 2	Nucleus	transcription regulator	H	-1.154	1.172	1.284	1.237	1.084	1.247
gi_114052217_ref_NM_001045925.1_	505722	TCEA1	transcription elongation factor A (SII), 1	Nucleus	transcription regulator	VH	-1.154	1.126	1.108	1.199	1.083	1.263
gi_134085756_ref_NM_001083392.1_	508522	GPN1	GPN-loop GTPase 1	Nucleus	transcription regulator	VH	-1.153	1.140	1.031	1.130	-1.288	0.278
gi_148228870_ref_NM_001098117.1_	540648	BCLAF1	BCL2-associated transcription factor 1	Nucleus	transcription regulator	VH	-1.152	1.155	1.035	1.153	-1.021	1.106
gi_119905975_ref_XM_602010.3_	523707	NCOA3	nuclear receptor coactivator 3	Nucleus	transcription regulator	VH	-1.152	1.131	1.432	0.839	1.968	0.167
gi_77735506_ref_NM_001034276.1_	507047	TAF11	TAF11 RNA polymerase II, TATA box binding protein (T	Nucleus	transcription regulator	H	-1.151	1.152	1.031	1.138	-1.431	0.089
gi_119908977_ref_XR_027764.1_	536676	NARG1	NMDA receptor regulated 1	Nucleus	transcription regulator	VH	-1.148	1.130	1.082	1.199	1.034	1.211
gi_115497643_ref_NM_001075582.1_	512840	POU2F1	POU class 2 homeobox 1	Nucleus	transcription regulator	VH	-1.148	1.140	1.112	1.234	1.116	1.325
gi_119912200_ref_XM_869372.2_	505147	PCGF2	polycomb group ring finger 2	Nucleus	transcription regulator	VH	-1.147	1.152	1.231	1.253	1.376	1.150
gi_149642818_ref_NM_001098968.1_	514443	TAF10	TAF10 RNA polymerase II, TATA box binding protein (T	Nucleus	transcription regulator	VH	-1.144	1.131	-1.419	0.073	-1.398	0.119
gi_114326277_ref_NM_174532.3_	282215	DNAJB6	DnaJ (Hsp40) homolog, subfamily B, member 6	Nucleus	transcription regulator	H	-1.143	1.126	-1.475	0.039	-1.713	0.007
gi_119907617_ref_XM_617789.3_	537616	POLD3	polymerase (DNA-directed), delta 3, accessory subunit	Nucleus	transcription regulator	VH	-1.140	1.115	-1.178	0.571	-1.672	0.009
gi_119918701_ref_XM_001250859.1_	510059	TFAM	transcription factor A, mitochondrial	Cytoplasm	transcription regulator	VH	-1.138	1.096	-1.012	1.042	-1.178	0.553
gi_139947655_ref_NM_001083689.1_	515903	FOXP1	forkhead box P1	Nucleus	transcription regulator	VH	-1.135	1.103	1.299	1.233	1.441	0.990
gi_119889347_ref_XM_584127.3_	538995	ETV3	ets variant 3	Nucleus	transcription regulator	H	-1.129	1.063	-1.773	0.005	1.078	1.259
gi_119904710_ref_XM_001250984.1_	507091	MPHOSPH8	M-phase phosphoprotein 8	Nucleus	transcription regulator	VH	-1.129	1.101	1.019	1.134	-1.103	0.789
gi_115496186_ref_NM_001076214.1_	540345	DRAP1	DR1-associated protein 1 (negative cofactor 2 alpha)	Nucleus	transcription regulator	VH	-1.128	1.099	-1.114	0.783	-1.120	0.782
gi_119895256_ref_XM_592234.3_	540197	NSD1	nuclear receptor binding SET domain protein 1	Nucleus	transcription regulator	VH	-1.127	1.082	1.020	0.959	1.651	0.527
gi_119889584_ref_XM_001250499.1_	533296	TRIM33	tripartite motif-containing 33	Nucleus	transcription regulator	VH	-1.127	1.092	1.086	1.206	1.185	1.341
gi_149643026_ref_NM_001098960.1_	513602	HDAC6	histone deacetylase 6	Nucleus	transcription regulator	H	-1.123	1.006	1.237	1.257	1.062	1.215
gi_75832104_ref_NM_001014916.2_	513312	NACA	nascent polypeptide-associated complex alpha subunit	Nucleus	transcription regulator	VH	-1.121	1.077	1.033	1.142	1.044	1.235
gi_84000392_ref_NM_001038212.1_	616311	PMF1	polyamine-modulated factor 1	Nucleus	transcription regulator	H	-1.120	0.970	1.023	0.976	-1.162	0.617
gi_77735780_ref_NM_001034413.1_	511852	ASB11	ankyrin repeat and SOCS box-containing 11	Nucleus	transcription regulator	H	-1.119	1.058	-1.226	0.375	-1.260	0.347
gi_114053212_ref_NM_001046271.1_	524184	ECD	ecdysoneless homolog (Drosophila)	Nucleus	transcription regulator	H	-1.119	1.066	1.253	1.251	-1.066	0.939
gi_114051857_ref_NM_001045960.1_	507187	TCEAL1	transcription elongation factor A (SII)-like 1	Nucleus	transcription regulator	H	-1.118	1.025	1.829	0.164	1.717	0.418
gi_31341991_ref_NM_174441.2_	282015	PSMC5	proteasome (prosome, macropain) 26S subunit, ATPas	Nucleus	transcription regulator	VH	-1.117	1.061	-1.193	0.505	-1.204	0.475
gi_119891154_ref_XM_863839.2_	537266	ZMIZ2	zinc finger, MIZ-type containing 2	Nucleus	transcription regulator	H	-1.113	1.040	-1.019	1.021	1.875	0.239
gi_119894912_ref_XM_580591.3_	504458	MLLT1	myeloid/lymphoid or mixed-lineage leukemia (trithorax	Nucleus	transcription regulator	VH	-1.112	1.026	-1.196	0.485	1.013	1.139
gi_115495158_ref_NM_001075975.1_	532936	TAF9	TAF9 RNA polymerase II, TATA box binding protein (TE	Nucleus	transcription regulator	VH	-1.112	1.025	1.132	1.229	-1.006	1.105
gi_77736302_ref_NM_001034679.1_	539640	RCAN1	regulator of calcineurin 1	Nucleus	transcription regulator	H	-1.111	1.053	1.731	0.255	1.372	1.128
gi_77735398_ref_NM_001034224.1_	504831	CNBP	CCHC-type zinc finger, nucleic acid binding protein	Nucleus	transcription regulator	VH	-1.109	1.032	-1.084	0.852	-1.112	0.742
gi_119889486_ref_XM_867242.2_	513730	NOTCH2	Notch homolog 2 (Drosophila)	Plasma Membrane	transcription regulator	H	-1.107	0.946	-1.971	0.002	1.316	1.224
gi_119916782_ref_XM_594080.3_	515964	RRN3	RRN3 RNA polymerase I transcription factor homolog (	Nucleus	transcription regulator	H	-1.107	0.939	1.041	1.151	1.002	1.154
gi_116003916_ref_NM_001076846.1_	513562	FUBP1	far upstream element (FUSE) binding protein 1	Nucleus	transcription regulator	VH	-1.105	1.025	1.089	1.215	1.007	1.160
gi_119908534_ref_XM_591419.3_	513692	SPEN	spen homolog, transcriptional regulator (Drosophila)	Nucleus	transcription regulator	VH	-1.105	1.055	-1.366	0.109	1.298	1.318
gi_155372154_ref_NM_001101217.1_	541080	FUBP3	far upstream element (FUSE) binding protein 3	Nucleus	transcription regulator	H	-1.104	1.030	1.126	1.244	1.186	1.341

gi_122692288_ref_NM_001080214.1_	281148	ETS2	v-ets erythroblastosis virus E26 oncogene homolog 2 (	Nucleus	transcription regulator	H	-1.099	1.005	-1.704	0.005	-1.751	0.005
gi_119909659_ref_XM_583272.3_	506776	LZTR1	leucine-zipper-like transcription regulator 1	Cytoplasm	transcription regulator	H	-1.097	1.018	-1.569	0.019	-1.039	1.006
gi_119902222_ref_XM_866403.2_	614181	HOMEZ	homeobox and leucine zipper encoding	Nucleus	transcription regulator	H	-1.095	0.949	1.668	0.334	1.832	0.279
gi_119906753_ref_XM_001250052.1_	782052	LOC100046341	basic transcription factor 3 pseudogene	unknown	transcription regulator	VH	-1.095	0.789	1.155	1.174	1.085	1.159
gi_119891667_ref_XM_865659.2_	509106	EZH2	enhancer of zeste homolog 2 (Drosophila)	Nucleus	transcription regulator	VH	-1.093	0.907	1.079	1.181	-1.358	0.161
gi_119906877_ref_XM_001250150.1_	781912	TAF2	TAF2 RNA polymerase II, TATA box binding protein (TE	Nucleus	transcription regulator	VH	-1.091	0.951	-1.059	0.924	-1.191	0.511
gi_119903945_ref_XM_001250691.1_	782101	COPS2	COP9 constitutive photomorphogenic homolog subunit	Cytoplasm	transcription regulator	H	-1.090	0.934	1.194	1.247	-1.400	0.107
gi_149642570_ref_NM_001099088.1_	538594	CREBZF	CREB/ATF bZIP transcription factor	Nucleus	transcription regulator	H	-1.090	1.003	1.118	1.227	1.347	1.218
gi_70778872_ref_NM_001025314.1_	281031	BTF3	basic transcription factor 3	Nucleus	transcription regulator	VH	-1.090	0.980	1.159	1.252	1.044	1.238
gi_119910805_ref_XM_593712.3_	515654	ATF5	activating transcription factor 5	Nucleus	transcription regulator	H	-1.089	0.970	-1.728	0.006	1.393	1.096
gi_77736488_ref_NM_001034772.1_	614915	HMGN1	high-mobility group nucleosome binding domain 1	Nucleus	transcription regulator	VH	-1.086	0.854	1.025	1.063	-1.134	0.612
gi_114051246_ref_NM_001046196.1_	515320	HBP1	HMG-box transcription factor 1	Nucleus	transcription regulator	H	-1.085	0.898	2.174	0.028	2.253	0.057
gi_119890471_ref_XM_581483.3_	505228	THRAP3	thyroid hormone receptor associated protein 3	Nucleus	transcription regulator	VH	-1.084	0.967	1.101	1.227	1.185	1.341
gi_119891865_ref_XM_590763.3_	513125	ATN1	atrophin 1	Nucleus	transcription regulator	VH	-1.083	0.957	-1.347	0.144	1.454	0.954
gi_147906545_ref_NM_001097995.1_	507659	TSG101	tumor susceptibility gene 101	Nucleus	transcription regulator	VH	-1.083	0.932	-1.173	0.563	-1.132	0.720
gi_119912207_ref_XM_598984.3_	520734	MED1	mediator complex subunit 1	Nucleus	transcription regulator	VH	-1.082	0.941	1.006	1.085	1.006	1.141
gi_119893309_ref_XM_601446.3_	523151	TCF20	transcription factor 20 (AR1)	Nucleus	transcription regulator	H	-1.081	0.861	-2.002	0.002	1.488	0.786
gi_147902867_ref_NM_001098050.1_	521194	MAML2	mastermind-like 2 (Drosophila)	Nucleus	transcription regulator	VH	-1.079	0.955	1.044	1.141	1.040	1.226
gi_115496729_ref_NM_001076365.1_	615330	YEATS4	YEATS domain containing 4	Nucleus	transcription regulator	VH	-1.077	0.843	1.170	1.242	-1.141	0.611
gi_134085919_ref_NM_001083450.1_	527134	LRCH4	leucine-rich repeats and calponin homology (CH) doma	Cytoplasm	transcription regulator	VH	-1.076	0.917	-1.820	0.002	1.155	1.334
gi_149642736_ref_NM_001098888.1_	506331	MED26	mediator complex subunit 26	Nucleus	transcription regulator	H	-1.075	0.903	-1.067	0.926	-1.112	0.782
gi_119904546_ref_XM_583090.3_	506618	FOXO1	forkhead box O1	Nucleus	transcription regulator	VH	-1.073	0.949	-1.590	0.015	-1.150	0.691
gi_77736446_ref_NM_001034751.1_	614212	LMO4	LIM domain only 4	Nucleus	transcription regulator	VH	-1.071	0.920	1.321	1.180	1.269	1.333
gi_149642796_ref_NM_001099041.1_	528606	EHMT1	euchromatic histone-lysine N-methyltransferase 1	Nucleus	transcription regulator	VH	-1.070	0.875	-1.119	0.731	1.028	1.173
gi_115495582_ref_NM_001076152.1_	539270	ABT1	activator of basal transcription 1	Nucleus	transcription regulator	VH	-1.068	0.921	-1.078	0.890	-1.427	0.087
gi_119907199_ref_XR_027800.1_	541023	ZNF202	zinc finger protein 202	Nucleus	transcription regulator	H	-1.067	0.882	1.017	1.006	1.283	1.327
gi_115495282_ref_NM_001075942.1_	530908	CREG1	cellular repressor of E1A-stimulated genes 1	Nucleus	transcription regulator	H	-1.066	0.897	2.174	0.028	1.883	0.237
gi_147898440_ref_NM_001098002.1_	509232	NRF1	nuclear respiratory factor 1	Nucleus	transcription regulator	H	-1.064	0.799	-1.048	0.938	1.042	1.186
gi_27806886_ref_NM_173912.1_	280796	FUS	fusion (involved in t(12;16) in malignant liposarcoma)	Nucleus	transcription regulator	VH	-1.064	0.846	-1.086	0.795	-1.209	0.432
gi_84370084_ref_NM_001038530.1_	511829	GTF2H2	general transcription factor IIH, polypeptide 2, 44kDa	Nucleus	transcription regulator	VH	-1.062	0.904	1.287	1.241	1.073	1.268
gi_115497917_ref_NM_001075610.1_	513297	L3MBTL2	l(3)mbt-like 2 (Drosophila)	Nucleus	transcription regulator	H	-1.061	0.883	-1.263	0.313	-1.192	0.509
gi_31343070_ref_NM_174000.2_	281036	CALR	calreticulin	Cytoplasm	transcription regulator	VH	-1.061	0.866	1.025	1.092	-1.046	1.007
gi_118151443_ref_NM_001078163.1_	777788	DDIT3	DNA-damage-inducible transcript 3	Nucleus	transcription regulator	H	-1.058	0.871	1.408	0.918	2.398	0.033
gi_94966888_ref_NM_001040546.1_	525389	MED27	mediator complex subunit 27	Nucleus	transcription regulator	VH	-1.058	0.857	-1.033	1.000	-1.101	0.797
gi_114051533_ref_NM_001046589.1_	617018	RNF4	ring finger protein 4	Nucleus	transcription regulator	VH	-1.058	0.829	-1.056	0.929	-1.093	0.846
gi_119909418_ref_XM_615157.3_	535152	DDX54	DEAD (Asp-Glu-Ala-Asp) box polypeptide 54	Nucleus	transcription regulator	H	-1.057	0.854	-1.250	0.357	-1.321	0.226
gi_119916790_ref_XM_588800.3_	511461	MKL2	MKL/myocardin-like 2	Nucleus	transcription regulator	H	-1.056	0.859	1.561	0.542	1.587	0.648
gi_117935054_ref_NM_174339.3_	281814	HIF1A	hypoxia inducible factor 1, alpha subunit (basic helix-lo	Nucleus	transcription regulator	VH	-1.056	0.859	-1.084	0.830	1.137	1.326
gi_83035094_ref_NM_001037616.1_	540444	HMGB2	high-mobility group box 2	Nucleus	transcription regulator	VH	-1.056	0.861	-1.148	0.671	-1.191	0.528
gi_156120704_ref_NM_001102029.1_	515680	NFX1	nuclear transcription factor, X-box binding 1	Nucleus	transcription regulator	VH	-1.056	0.865	1.127	1.237	1.072	1.262
gi_30466259_ref_NM_178573.1_	353120	BRCA1	breast cancer 1, early onset	Nucleus	transcription regulator	VH	-1.055	0.803	1.019	1.092	-1.352	0.163
gi_119889005_ref_XM_001254027.1_	524645	DCAF6	DDB1 and CUL4 associated factor 6	Nucleus	transcription regulator	H	-1.054	0.854	1.277	1.241	1.138	1.327
gi_119917147_ref_XM_585218.3_	508442	BAZ1B	bromodomain adjacent to zinc finger domain, 1B	Nucleus	transcription regulator	VH	-1.052	0.815	-1.395	0.077	1.091	1.280
gi_156120490_ref_NM_001101921.1_	510084	ANKRD10	ankyrin repeat domain 10	Nucleus	transcription regulator	H	-1.048	0.790	-1.075	0.801	-1.032	0.986
gi_119896053_ref_XM_001249429.1_	781150	SAP30	Sin3A-associated protein, 30kDa	Nucleus	transcription regulator	VH	-1.045	0.827	1.115	1.227	-1.528	0.034
gi_119914774_ref_XR_028584.1_	522045	SMARCC1	SWI/SNF related, matrix associated, actin dependent r	Nucleus	transcription regulator	VH	-1.045	0.759	-1.076	0.770	1.007	1.113
gi_118601877_ref_NM_001079642.1_	780787	SRA1	steroid receptor RNA activator 1	Nucleus	transcription regulator	H	-1.044	0.803	-1.100	0.796	1.073	1.255
gi_119910728_ref_XM_593095.2_	540310	GRLF1	glucocorticoid receptor DNA binding factor 1	Nucleus	transcription regulator	VH	-1.043	0.791	-2.427	0.000	1.173	1.332
gi_77735442_ref_NM_001034244.1_	505401	TADA2A	transcriptional adaptor 2A	Nucleus	transcription regulator	VH	-1.043	0.752	1.295	1.238	1.089	1.270
gi_156120910_ref_NM_001102132.1_	530776	LASS5	LAG1 homolog, ceramide synthase 5	Cytoplasm	transcription regulator	VH	-1.042	0.721	-1.072	0.868	-1.089	0.787
gi_119905848_ref_XM_585747.3_	508899	NCOA6	nuclear receptor coactivator 6	Nucleus	transcription regulator	VH	-1.041	0.827	1.180	1.254	1.400	1.101
gi_119923843_ref_XM_585257.3_	508477	ZNF593	zinc finger protein 593	Nucleus	transcription regulator	H	-1.039	0.594	-1.257	0.168	-1.404	0.067
gi_119917684_ref_XM_604843.3_	526472	LDB1	LIM domain binding 1	Nucleus	transcription regulator	VH	-1.039	0.795	1.090	1.201	1.416	1.048
gi_78369455_ref_NM_001035441.1_	614028	NPM1 (includes E	nucleophosmin 1	Nucleus	transcription regulator	VH	-1.039	0.759	1.033	1.111	-1.005	1.098
gi_119887281_ref_XM_613744.3_	407215	KAT2B	K(lysine) acetyltransferase 2B	Nucleus	transcription regulator	H	-1.038	0.798	1.233	1.257	1.063	1.262
gi_77736563_ref_NM_001034792.1_	616425	SUPT4H1	suppressor of Ty 4 homolog 1 (S. cerevisiae)	Nucleus	transcription regulator	VH	-1.036	0.752	-1.294	0.216	-1.324	0.200
gi_73853757_ref_NM_174492.2_	282126	YWHAH	tyrosine 3-monooxygenase/tryptophan 5-monooxygena	Cytoplasm	transcription regulator	VH	-1.035	0.815	-1.038	1.009	-1.184	0.568
gi_134085692_ref_NM_001083533.1_	789710	KLF13	Kruppel-like factor 13	Nucleus	transcription regulator	H	-1.033	0.740	-1.342	0.150	1.030	1.184
gi_119912595_ref_XM_610873.3_	532359	FOXK2	forkhead box K2	Nucleus	transcription regulator	VH	-1.032	0.751	-1.155	0.594	-1.117	0.745
gi_114050744_ref_NM_001046475.1_	540374	GMEB1	glucocorticoid modulatory element binding protein 1	Nucleus	transcription regulator	H	-1.031	0.688	-1.041	0.971	1.098	1.288
gi_119915495_ref_XM_869170.2_	514062	EHMT2	euchromatic histone-lysine N-methyltransferase 2	Nucleus	transcription regulator	H	-1.030	0.759	-1.351	0.136	1.832	0.278
gi_114051601_ref_NM_001046442.1_	538775	KHDRBS1	KH domain containing, RNA binding, signal transductio	Nucleus	transcription regulator	VH	-1.030	0.781	1.164	1.252	1.019	1.189
gi_157427939_ref_NM_001105407.1_	533984	SUB1	SUB1 homolog (S. cerevisiae)	Nucleus	transcription regulator	H	-1.029	0.737	1.255	1.250	1.041	1.159
gi_118151211_ref_NM_001078062.1_	616553	PFDN1	prefoldin subunit 1	Cytoplasm	transcription regulator	VH	-1.028	0.734	-1.382	0.098	-1.566	0.023
gi_156121302_ref_NM_001102329.1_	782752	GTF3C2	general transcription factor IIIC, polypeptide 2, beta 11(	Nucleus	transcription regulator	VH	-1.026	0.748	-1.008	1.065	1.079	1.285
gi_115494903_ref_NM_001076209.1_	540248	SMAD4	SMAD family member 4	Nucleus	transcription regulator	VH	-1.026	0.759	1.073	1.193	-1.020	1.051
gi_115497821_ref_NM_001076452.1_	617269	ASB8	ankyrin repeat and SOCS box-containing 8	Nucleus	transcription regulator	H	-1.024	0.698	1.409	0.821	1.587	0.630
gi_119894504_ref_XM_583044.3_	506578	ELL	elongation factor RNA polymerase II	Nucleus	transcription regulator	H	-1.024	0.718	-1.128	0.714	-1.045	0.968
gi_119888577_ref_XM_001253150.1_	786336	PTMA (includes E	prothymosin, alpha	Nucleus	transcription regulator	VH	-1.023	0.741	1.065	1.170	-1.047	0.980
gi_119895456_ref_XM_589487.3_	512048	REXO1	REX1, RNA exonuclease 1 homolog (S. cerevisiae)	Nucleus	transcription regulator	H	-1.021	0.687	-2.922	0.000	-1.240	0.360
gi_134085770_ref_NM_001083395.1_	508663	ZNF277	zinc finger protein 277	Nucleus	transcription regulator	H	-1.020	0.726	1.264	1.171	1.107	1.298
gi_148230363_ref_NM_001098064.1_	527471	HNRNPD	heterogeneous nuclear ribonucleoprotein D (AU-rich el	Nucleus	transcription regulator	VH	-1.018	0.698	-1.004	1.064	-1.067	0.938
gi_160333190_ref_NM_001034730.2_	541303	MED17	mediator complex subunit 17	Nucleus	transcription regulator	VH	-1.017	0.718	1.011	1.092	-1.376	0.142
gi_115496895_ref_NM_001075188.1_	505102	NFKBIL2	nuclear factor of kappa light polypeptide gene enhance	Cytoplasm	transcription regulator	H	-1.015	0.693	-1.507	0.033	-1.141	0.663

gi_125991889_ref_NM_001081614.1_	541229	SF1	splicing factor 1	Nucleus	transcription regulator	VH	-1.015	0.710	1.009	1.098	1.078	1.232
gi_119894806_ref_XM_592855.3_	614936	ILF3	interleukin enhancer binding factor 3, 90kDa	Nucleus	transcription regulator	VH	-1.013	0.716	-1.113	0.793	-1.021	1.089
gi_77736218_ref_NM_001034638.1_	537402	RBBP7	retinoblastoma binding protein 7	Nucleus	transcription regulator	VH	-1.013	0.692	1.024	1.096	-1.124	0.735
gi_164448722_ref_NM_001046485.2_	540632	TARDBP	TAR DNA binding protein	Nucleus	transcription regulator	VH	-1.007	0.661	1.043	1.153	1.038	1.219
gi_156120468_ref_NM_001101910.1_	509111	TP53BP1	tumor protein p53 binding protein 1	Nucleus	transcription regulator	H	1.001	0.560	1.576	0.500	1.755	0.358
gi_119913817_ref_XM_875451.2_	518504	SIN3A	SIN3 homolog A, transcription regulator (yeast)	Nucleus	transcription regulator	VH	1.002	0.658	1.044	1.153	1.178	1.334
gi_114051553_ref_NM_001046065.1_	510921	GTF2E2	general transcription factor IIE, polypeptide 2, beta 34k	Nucleus	transcription regulator	VH	1.006	0.663	-1.107	0.814	-1.297	0.264
gi_155372150_ref_NM_001101215.1_	540757	TFAP4	transcription factor AP-4 (activating enhancer binding p	Nucleus	transcription regulator	H	1.007	0.650	-1.461	0.056	-1.291	0.207
gi_119906615_ref_XM_617828.3_	537652	TRPS1	trichorhinophalangeal syndrome I	Nucleus	transcription regulator	H	1.008	0.635	1.513	0.643	1.674	0.495
gi_119901460_ref_XM_618496.3_	538296	BACH2 (includes	BTB and CNC homology 1, basic leucine zipper transc	Nucleus	transcription regulator	H	1.009	0.529	1.411	0.759	2.688	0.012
gi_158508565_ref_NM_001110004.1_	511826	HUWE1	HECT, UBA and WWE domain containing 1	Nucleus	transcription regulator	VH	1.009	0.616	-1.003	1.021	1.243	1.341
gi_115496707_ref_NM_001076363.1_	615199	UBA52	ubiquitin A-52 residue ribosomal protein fusion product	Cytoplasm	transcription regulator	VH	1.010	0.605	-1.035	0.966	1.015	1.157
gi_114050926_ref_NM_001046126.1_	512648	ELL3	elongation factor RNA polymerase II-like 3	Nucleus	transcription regulator	H	1.011	0.595	1.113	1.219	1.462	0.939
gi_114052003_ref_NM_001046389.1_	534894	PQBP1	polyglutamine binding protein 1	Nucleus	transcription regulator	H	1.011	0.612	-1.106	0.816	1.000	1.120
gi_119919219_ref_XM_001256021.1_	789199	NFRKB	nuclear factor related to kappaB binding protein	Nucleus	transcription regulator	H	1.013	0.616	-1.654	0.012	-1.155	0.630
gi_119900354_ref_XM_618263.3_	538070	RFX3	regulatory factor X, 3 (influences HLA class II expressi	Nucleus	transcription regulator	H	1.014	0.583	1.361	1.029	1.040	1.210
gi_62460491_ref_NM_001014901.1_	511512	RBM14	RNA binding motif protein 14	Nucleus	transcription regulator	VH	1.014	0.603	-1.145	0.649	-1.071	0.904
gi_118151327_ref_NM_001078131.1_	768316	NRBF2	nuclear receptor binding factor 2	Nucleus	transcription regulator	H	1.019	0.589	-1.097	0.774	-1.429	0.082
gi_77735636_ref_NM_001034342.1_	509107	ATF4	activating transcription factor 4 (tax-responsive enhanc	Nucleus	transcription regulator	VH	1.019	0.572	-1.307	0.203	-1.117	0.775
gi_116004076_ref_NM_001076927.1_	540802	ASB3	ankyrin repeat and SOCS box-containing 3	Nucleus	transcription regulator	VH	1.020	0.536	1.392	0.989	1.157	1.326
gi_114052532_ref_NM_001045899.1_	504820	BLZF1	basic leucine zipper nuclear factor 1	Cytoplasm	transcription regulator	VH	1.023	0.597	-1.050	0.983	-1.099	0.844
gi_77735898_ref_NM_001034474.1_	514852	TAX1BP3	Tax1 (human T-cell leukemia virus type I) binding prote	Nucleus	transcription regulator	H	1.025	0.514	1.373	0.953	1.532	0.750
gi_114051260_ref_NM_001046617.1_	618296	POLR3K	polymerase (RNA) III (DNA directed) polypeptide K, 12	Nucleus	transcription regulator	H	1.027	0.551	-1.520	0.028	-1.593	0.018
gi_119895680_ref_XM_869095.2_	540964	TCERG1	transcription elongation regulator 1	Nucleus	transcription regulator	VH	1.027	0.559	-1.104	0.780	-1.106	0.813
gi_155372072_ref_NM_001101173.1_	538086	CAND1	cullin-associated and neddylation-dissociated 1	Cytoplasm	transcription regulator	VH	1.028	0.518	1.047	1.143	-1.269	0.304
gi_119894811_ref_XM_881853.2_	532791	KEAP1	kelch-like ECH-associated protein 1	Cytoplasm	transcription regulator	VH	1.028	0.573	-1.685	0.006	-1.150	0.687
gi_119920299_ref_XM_001254442.1_	786886	ELK1	ELK1, member of ETS oncogene family	Nucleus	transcription regulator	H	1.029	0.539	-1.030	1.012	1.119	1.314
gi_82697312_ref_NM_001037444.1_	404126	HDAC1	histone deacetylase 1	Nucleus	transcription regulator	VH	1.029	0.535	1.142	1.239	1.166	1.331
gi_119911640_ref_XM_583607.3_	507057	PHF12	PHD finger protein 12	Nucleus	transcription regulator	VH	1.029	0.518	-1.185	0.522	1.110	1.280
gi_119912534_ref_XM_001256027.1_	789613	SMARCD2	SWI/SNF related, matrix associated, actin dependent r	Nucleus	transcription regulator	VH	1.030	0.565	1.001	1.067	-1.083	0.872
gi_66792821_ref_NM_001024517.1_	511446	CXXC1	CXXC finger 1 (PHD domain)	Nucleus	transcription regulator	VH	1.031	0.515	-1.020	0.995	1.154	1.326
gi_119923597_ref_XM_001250681.1_	784388	RPL7	ribosomal protein L7	Cytoplasm	transcription regulator	VH	1.035	0.526	-1.033	1.013	1.091	1.299
gi_87196500_ref_NM_174049.2_	281141	ENO1	enolase 1, (alpha)	Cytoplasm	transcription regulator	VH	1.036	0.552	-1.084	0.883	-1.094	0.882
gi_119893132_ref_XM_001249704.1_	513643	FOXM1	forkhead box M1	Nucleus	transcription regulator	VH	1.037	0.543	-1.105	0.821	1.055	1.225
gi_77735836_ref_NM_001034441.1_	513315	PSMD9	proteasome (prosome, macropain) 26S subunit, non-A	Cytoplasm	transcription regulator	VH	1.038	0.514	-1.146	0.654	-1.151	0.637
gi_156121000_ref_NM_001102177.1_	534669	GTF2I	general transcription factor IIi	Nucleus	transcription regulator	VH	1.044	0.518	1.458	0.810	1.449	0.952
gi_115495344_ref_NM_001075799.1_	520111	TRIM27	tripartite motif-containing 27	Nucleus	transcription regulator	VH	1.051	0.493	-1.058	0.964	-1.047	1.023
gi_88319934_ref_NM_174815.2_	287023	YBX1	Y box binding protein 1	Nucleus	transcription regulator	VH	1.056	0.468	-1.002	1.079	-1.134	0.733
gi_136256478_ref_NM_001077899.2_	510807	NFE2L3	nuclear factor (erythroid-derived 2)-like 3	Nucleus	transcription regulator	H	1.063	0.420	1.419	0.901	1.333	1.226
gi_119889268_ref_XM_581262.3_	505039	GATAD2B	GATA zinc finger domain containing 2B	Nucleus	transcription regulator	VH	1.065	0.438	-1.577	0.018	-1.082	0.882
gi_119901159_ref_XM_581531.3_	538619	HEY2	hairy/enhancer-of-split related with YRPW motif 2	Nucleus	transcription regulator	H	1.067	0.402	1.142	1.215	-1.125	0.745
gi_119924677_ref_XM_588862.3_	511514	HIRA	HIR histone cell cycle regulation defective homolog A (:	Nucleus	transcription regulator	H	1.069	0.387	-1.858	0.001	-1.220	0.406
gi_119889130_ref_XM_588564.3_	511264	SKP1	S-phase kinase-associated protein 1	Nucleus	transcription regulator	VH	1.069	0.407	1.058	1.163	-1.073	0.908
gi_78369329_ref_NM_001035467.1_	615692	SAP18	Sin3A-associated protein, 18kDa	Nucleus	transcription regulator	VH	1.072	0.418	-1.253	0.354	-1.225	0.449
gi_156121016_ref_NM_001102185.1_	535312	PIAS3	protein inhibitor of activated STAT, 3	Nucleus	transcription regulator	H	1.074	0.396	1.112	1.216	1.411	1.036
gi_62751724_ref_NM_001015658.1_	534883	VPS72	vacuolar protein sorting 72 homolog (S. cerevisiae)	Nucleus	transcription regulator	VH	1.077	0.395	1.131	1.243	1.162	1.330
gi_119887246_ref_XM_584216.3_	507577	EAF1	ELL associated factor 1	Nucleus	transcription regulator	H	1.078	0.407	-1.728	0.006	-1.358	0.163
gi_114052838_ref_NM_001046226.1_	516550	FEM1A	fem-1 homolog a (C. elegans)	Nucleus	transcription regulator	H	1.078	0.394	-1.130	0.703	-1.251	0.350
gi_119915248_ref_XM_871526.2_	522029	RPL7L1	ribosomal protein L7-like 1	Cytoplasm	transcription regulator	VH	1.078	0.392	-1.117	0.761	-1.276	0.311
gi_84992998_ref_NM_001034310.2_	508043	PTTG1	pituitary tumor-transforming 1	Nucleus	transcription regulator	VH	1.079	0.387	-1.133	0.679	-1.270	0.319
gi_119919944_ref_XM_590515.3_	512908	HCFC1	host cell factor C1 (VP16-accessory protein)	Nucleus	transcription regulator	VH	1.080	0.368	-3.954	0.000	1.023	1.154
gi_119915977_ref_XM_588519.3_	511224	NFATC1	nuclear factor of activated T-cells, cytoplasmic, calcine	Nucleus	transcription regulator	H	1.080	0.370	1.458	0.714	2.414	0.031
gi_114051440_ref_NM_001046614.1_	618141	ZNF3	zinc finger protein 3	Nucleus	transcription regulator	VH	1.084	0.365	1.004	1.085	1.111	1.316
gi_119906488_ref_XM_583747.3_	507179	COPS5	COP9 constitutive photomorphogenic homolog subunit	Nucleus	transcription regulator	VH	1.087	0.364	-1.112	0.752	-1.516	0.040
gi_78369417_ref_NM_001035417.1_	537706	THOC4	THO complex 4	Nucleus	transcription regulator	VH	1.089	0.348	-1.491	0.036	-1.713	0.007
gi_77736184_ref_NM_001034619.1_	534898	ANKRD46	ankyrin repeat domain 46	Nucleus	transcription regulator	H	1.090	0.354	1.370	1.020	1.217	1.343
gi_115496725_ref_NM_001076029.1_	534579	TFDP1	transcription factor Dp-1	Nucleus	transcription regulator	VH	1.091	0.358	-1.307	0.225	-1.461	0.062
gi_62751822_ref_NM_001015575.1_	511571	RNF25	ring finger protein 25	Nucleus	transcription regulator	H	1.092	0.341	-1.229	0.349	-1.113	0.762
gi_77735524_ref_NM_001034286.1_	507245	ECSIT	ECSIT homolog (Drosophila)	Nucleus	transcription regulator	H	1.094	0.355	-1.528	0.025	-1.879	0.002
gi_115495422_ref_NM_001075146.1_	407223	HDAC2	histone deacetylase 2	Nucleus	transcription regulator	VH	1.094	0.360	1.120	1.237	-1.133	0.730
gi_75832050_ref_NM_173999.2_	281032	BTG1	B-cell translocation gene 1, anti-proliferative	Nucleus	transcription regulator	VH	1.096	0.320	-1.595	0.011	-1.649	0.012
gi_82697392_ref_NM_001037482.1_	540069	CIAO1	cytosolic iron-sulfur protein assembly 1 homolog (S. ce	Nucleus	transcription regulator	H	1.100	0.332	-1.103	0.802	-1.181	0.546
gi_115497993_ref_NM_001076468.1_	617553	BAT4	HLA-B associated transcript 4	Nucleus	transcription regulator	VH	1.100	0.342	-1.076	0.888	-1.186	0.532
gi_115496174_ref_NM_001076523.1_	618849	HMGA1	high mobility group AT-hook 1	Nucleus	transcription regulator	VH	1.102	0.304	-1.148	0.597	-1.291	0.257
gi_119893304_ref_XM_583656.3_	507102	SREBF2	sterol regulatory element binding transcription factor 2	Nucleus	transcription regulator	VH	1.104	0.325	-2.539	0.000	1.113	1.299
gi_114052790_ref_NM_001046289.1_	528003	ANKRA2	ankyrin repeat, family A (RFXANK-like), 2	Cytoplasm	transcription regulator	H	1.104	0.309	1.074	1.169	-1.341	0.174
gi_119884987_ref_XM_588490.3_	539633	HLTF	helicase-like transcription factor	Nucleus	transcription regulator	VH	1.104	0.317	-1.007	1.047	-1.221	0.423
gi_77736278_ref_NM_001034667.1_	539223	LASS2	LAG1 homolog, ceramide synthase 2	Nucleus	transcription regulator	VH	1.104	0.333	1.027	1.149	-1.039	1.047
gi_118151295_ref_NM_001078114.1_	768240	HOXB4	homeobox B4	Nucleus	transcription regulator	H	1.105	0.319	-1.136	0.691	-1.178	0.563
gi_115496355_ref_NM_001076329.1_	614472	POLR2I	polymerase (RNA) II (DNA directed) polypeptide I, 14.5	Nucleus	transcription regulator	H	1.105	0.310	-1.253	0.314	-1.912	0.002
gi_114050962_ref_NM_001046481.1_	540558	MBD1	methyl-CpG binding domain protein 1	Nucleus	transcription regulator	H	1.106	0.320	-1.440	0.064	-1.099	0.775
gi_77735922_ref_NM_001034486.1_	515299	MED4	mediator complex subunit 4	Nucleus	transcription regulator	H	1.107	0.312	1.006	1.075	-1.200	0.468
gi_118150887_ref_NM_001077888.1_	509238	TAF1C	TATA box binding protein (TBP)-associated factor, RNA	Nucleus	transcription regulator	H	1.109	0.317	-1.826	0.006	-1.163	0.624
gi_115497247_ref_NM_001076079.1_	536696	YAF2	YY1 associated factor 2	Nucleus	transcription regulator	H	1.109	0.315	-1.190	0.524	-1.253	0.359

gi_119911525_ref_XM_001252957.1_	786013	AATF	apoptosis antagonizing transcription factor	Nucleus	transcription regulator	VH	1.110	0.323	-1.153	0.661	-1.451	0.073
gi_73853780_ref_NM_001029845.2_	516979	IRF3	interferon regulatory factor 3	Nucleus	transcription regulator	VH	1.110	0.315	1.086	1.203	1.213	1.341
gi_119908788_ref_XM_612405.3_	540633	KDM5B	lysine (K)-specific demethylase 5B	Nucleus	transcription regulator	VH	1.110	0.316	1.165	1.248	1.227	1.344
gi_119890708_ref_XM_581455.3_	505204	ANKIB1	ankyrin repeat and IBR domain containing 1	Nucleus	transcription regulator	H	1.111	0.309	1.085	1.183	1.013	1.143
gi_119912680_ref_XM_583104.3_	506628	CBX2	chromobox homolog 2 (Pc class homolog, Drosophila)	Nucleus	transcription regulator	H	1.116	0.301	-2.465	0.000	-1.688	0.008
gi_119887162_ref_XM_596225.3_	518043	DIP2A	DIP2 disco-interacting protein 2 homolog A (Drosophila	Nucleus	transcription regulator	H	1.120	0.291	-1.072	0.804	1.253	1.342
gi_119331215_ref_NM_001079788.1_	615512	CCNH	cyclin H	Nucleus	transcription regulator	H	1.121	0.279	1.103	1.203	-1.254	0.326
gi_77735576_ref_NM_001034312.1_	508055	CNOT7	CCR4-NOT transcription complex, subunit 7	Nucleus	transcription regulator	VH	1.121	0.280	1.036	1.141	-1.077	0.917
gi_114051078_ref_NM_001046218.1_	516010	SMAD2	SMAD family member 2	Nucleus	transcription regulator	VH	1.125	0.278	1.115	1.235	1.092	1.272
gi_119919717_ref_XM_580654.3_	504514	ELF4	E74-like factor 4 (ets domain transcription factor)	Nucleus	transcription regulator	VH	1.127	0.272	-1.113	0.628	1.103	1.298
gi_115495532_ref_NM_001075161.1_	504400	RBCK1	RanBP-type and C3HC4-type zinc finger containing 1	Cytoplasm	transcription regulator	H	1.128	0.275	-1.003	1.052	1.493	0.857
gi_119906545_ref_XM_584905.3_	508162	NCOA2	nuclear receptor coactivator 2	Nucleus	transcription regulator	VH	1.128	0.274	-1.226	0.365	1.197	1.338
gi_115497589_ref_NM_001076137.1_	538961	ANKRD54	ankyrin repeat domain 54	Nucleus	transcription regulator	H	1.130	0.278	-1.138	0.703	-1.202	0.511
gi_156523141_ref_NM_001102515.1_	534582	NFE2L1	nuclear factor (erythroid-derived 2)-like 1	Nucleus	transcription regulator	VH	1.130	0.258	-1.140	0.603	1.092	1.269
gi_157427957_ref_NM_001105416.1_	537503	SMARCA5	SWI/SNF related, matrix associated, actin dependent r	Nucleus	transcription regulator	VH	1.130	0.265	-1.140	0.674	-1.138	0.695
gi_47564069_ref_NM_001001162.1_	407240	USF2	upstream transcription factor 2, c-fos interacting	Nucleus	transcription regulator	VH	1.131	0.278	1.131	1.243	1.078	1.287
gi_119917224_ref_XM_583735.3_	507169	TRRAP	transformation/transcription domain-associated protein	Nucleus	transcription regulator	H	1.135	0.261	-1.879	0.004	1.091	1.287
gi_119893060_ref_XM_612715.3_	540703	FOXJ2	forkhead box J2	Nucleus	transcription regulator	VH	1.136	0.251	1.224	1.254	1.204	1.343
gi_119915805_ref_XM_614932.3_	541184	E2F3	E2F transcription factor 3	Nucleus	transcription regulator	H	1.147	0.155	-1.192	0.276	-1.504	0.041
gi_119895278_ref_XM_604851.2_	526480	ZNF496	zinc finger protein 496	Nucleus	transcription regulator	H	1.147	0.235	-1.322	0.159	-1.150	0.641
gi_114051222_ref_NM_001046198.1_	515363	PHB2	prohibitin 2	Cytoplasm	transcription regulator	VH	1.149	0.233	-1.404	0.087	-1.303	0.252
gi_119919137_ref_XM_879600.2_	531109	TAF6L	TAF6-like RNA polymerase II, p300/CBP-associated fa	Nucleus	transcription regulator	H	1.152	0.227	-1.111	0.732	-1.041	0.997
gi_77736342_ref_NM_001034699.1_	540272	PA2G4	proliferation-associated 2G4, 38kDa	Nucleus	transcription regulator	VH	1.153	0.226	-1.099	0.843	-1.250	0.387
gi_84000346_ref_NM_001038187.1_	539760	ILF2 (includes EG	interleukin enhancer binding factor 2, 45kDa	Nucleus	transcription regulator	VH	1.156	0.211	-1.242	0.353	-1.372	0.147
gi_84370076_ref_NM_001038526.1_	511048	RUVBL2	RuvB-like 2 (E. coli)	Nucleus	transcription regulator	VH	1.156	0.221	-1.394	0.102	-1.476	0.057
gi_148224063_ref_NM_001098081.1_	534353	YY1	YY1 transcription factor	Nucleus	transcription regulator	H	1.161	0.210	-1.243	0.370	1.050	1.231
gi_119895592_ref_XM_868478.2_	404125	HDAC3	histone deacetylase 3	Nucleus	transcription regulator	VH	1.166	0.206	-1.064	0.913	-1.158	0.625
gi_119921496_ref_XM_587845.3_	510673	PTTG1	pituitary tumor-transforming 1	Nucleus	transcription regulator	VH	1.168	0.194	-1.207	0.448	-1.388	0.121
gi_114051408_ref_NM_001046142.1_	513231	GTF2B	general transcription factor IIB	Nucleus	transcription regulator	VH	1.171	0.193	1.066	1.170	-1.002	1.096
gi_156120816_ref_NM_001102085.1_	524920	MYEF2	myelin expression factor 2	Nucleus	transcription regulator	VH	1.183	0.175	-1.586	0.014	-1.436	0.084
gi_115496233_ref_NM_001075742.1_	516578	TBP	TATA box binding protein	Nucleus	transcription regulator	VH	1.186	0.173	-1.040	1.001	-1.207	0.486
gi_119894521_ref_XM_866768.2_	510465	CRTC1	CREB regulated transcription coactivator 1	Nucleus	transcription regulator	H	1.187	0.180	1.113	1.217	1.451	0.938
gi_119904185_ref_XM_586742.3_	509719	ASB6	ankyrin repeat and SOCS box-containing 6	Nucleus	transcription regulator	H	1.188	0.172	-1.884	0.000	-2.102	0.000
gi_77735588_ref_NM_001034318.1_	508312	SFRS2	splicing factor, arginine/serine-rich 2	Nucleus	transcription regulator	VH	1.188	0.156	-1.271	0.268	-1.247	0.364
gi_123959763_ref_NM_001080735.1_	531369	TTF2	transcription termination factor, RNA polymerase II	Nucleus	transcription regulator	VH	1.189	0.167	1.138	1.247	-1.092	0.883
gi_126723003_ref_NM_001082454.1_	540641	CCRN4L	CCR4 carbon catabolite repression 4-like (S. cerevisia	Nucleus	transcription regulator	H	1.190	0.170	-1.669	0.006	-1.847	0.002
gi_119894329_ref_XM_866758.2_	615059	HTT	huntingtin	Cytoplasm	transcription regulator	H	1.191	0.168	-1.673	0.009	-1.157	0.608
gi_157074101_ref_NM_001103293.1_	540294	MED20	mediator complex subunit 20	Nucleus	transcription regulator	H	1.191	0.167	-1.142	0.674	-1.192	0.534
gi_119889053_ref_XM_863928.2_	506759	IFI16	interferon, gamma-inducible protein 16	Nucleus	transcription regulator	VH	1.191	0.164	1.795	0.194	2.037	0.129
gi_114052195_ref_NM_001046364.1_	533977	BUD31 (includes I	BUD31 homolog (S. cerevisiae)	Nucleus	transcription regulator	VH	1.197	0.160	-1.169	0.589	-1.371	0.145
gi_114052215_ref_NM_001046362.1_	533951	PLRG1	pleiotropic regulator 1 (PRL1 homolog, Arabidopsis)	Nucleus	transcription regulator	VH	1.201	0.156	-1.079	0.897	-1.243	0.381
gi_119906375_ref_XM_592984.3_	515040	ZHX2	zinc fingers and homeoboxes 2	Nucleus	transcription regulator	H	1.204	0.145	1.012	0.972	1.363	1.114
gi_115497369_ref_NM_001075155.1_	504336	DAXX	death-domain associated protein	Nucleus	transcription regulator	VH	1.210	0.145	-1.216	0.408	-1.341	0.189
gi_118151325_ref_NM_001078128.1_	768313	SOX4	SRY (sex determining region Y)-box 4	Nucleus	transcription regulator	VH	1.214	0.145	-2.428	0.000	-1.245	0.345
gi_115496557_ref_NM_001075712.1_	515389	MTA2	metastasis associated 1 family, member 2	Nucleus	transcription regulator	VH	1.218	0.132	-1.116	0.743	-1.191	0.528
gi_155371882_ref_NM_001101076.1_	511475	RUVBL1	RuvB-like 1 (E. coli)	Nucleus	transcription regulator	VH	1.218	0.131	-1.468	0.050	-1.936	0.001
gi_62460609_ref_NM_001014956.1_	539584	NFYA	nuclear transcription factor Y, alpha	Nucleus	transcription regulator	H	1.220	0.136	-1.119	0.753	-1.064	0.932
gi_114052566_ref_NM_001045897.1_	504755	NAB2	NGFI-A binding protein 2 (EGR1 binding protein 2)	Nucleus	transcription regulator	H	1.224	0.131	-1.953	0.000	-1.162	0.583
gi_147904941_ref_NM_001011678.2_	497024	NFE2L2	nuclear factor (erythroid-derived 2)-like 2	Nucleus	transcription regulator	H	1.227	0.119	1.329	1.158	1.853	0.261
gi_157074203_ref_NM_001103347.1_	100125315	TEAD2	TEA domain family member 2	Nucleus	transcription regulator	H	1.230	0.123	-1.044	0.956	1.193	1.341
gi_116003842_ref_NM_001076809.1_	506235	HSF1	heat shock transcription factor 1	Nucleus	transcription regulator	VH	1.231	0.119	-1.555	0.021	-1.330	0.187
gi_62752036_ref_NM_001015525.1_	505638	TAF6	TAF6 RNA polymerase II, TATA box binding protein (TE	Nucleus	transcription regulator	VH	1.232	0.119	-1.088	0.856	1.052	1.250
gi_157074103_ref_NM_001103294.1_	540525	GTF2E1	general transcription factor IIE, polypeptide 1, alpha 56	Nucleus	transcription regulator	H	1.233	0.117	-1.102	0.835	-1.657	0.011
gi_118601827_ref_NM_001079618.1_	613879	MED30	mediator complex subunit 30	Nucleus	transcription regulator	H	1.234	0.110	-1.570	0.015	-1.943	0.001
gi_119912413_ref_XM_601299.3_	497012	UBTF	upstream binding transcription factor, RNA polymerase	Nucleus	transcription regulator	VH	1.234	0.110	1.152	1.246	1.213	1.343
gi_77735432_ref_NM_001034237.1_	505293	MED6	mediator complex subunit 6	Nucleus	transcription regulator	H	1.237	0.110	-1.048	0.961	-1.207	0.490
gi_66792729_ref_NM_001024506.1_	509855	IRF9	interferon regulatory factor 9	Nucleus	transcription regulator	H	1.238	0.108	1.527	0.589	2.828	0.008
gi_126723253_ref_NM_001080242.2_	508233	RELA	v-rel reticuloendotheliosis viral oncogene homolog A (a	Nucleus	transcription regulator	H	1.242	0.109	-1.327	0.188	-1.235	0.371
gi_134085614_ref_NM_001083439.1_	519808	SUPT5H	suppressor of Ty 5 homolog (S. cerevisiae)	Nucleus	transcription regulator	VH	1.246	0.097	-1.279	0.289	-1.186	0.547
gi_119920138_ref_XM_001249858.1_	767875	HMGN5	high-mobility group nucleosome binding domain 5	Nucleus	transcription regulator	H	1.256	0.092	-1.056	0.890	-1.528	0.034
gi_115497273_ref_NM_001075868.1_	525329	NCOA4	nuclear receptor coactivator 4	Nucleus	transcription regulator	VH	1.266	0.082	1.066	1.178	-1.093	0.848
gi_114052005_ref_NM_001046150.1_	513510	GTF2H1	general transcription factor IIH, polypeptide 1, 62kDa	Nucleus	transcription regulator	VH	1.267	0.083	-1.095	0.820	-1.225	0.409
gi_118601765_ref_NM_001079589.1_	511896	GTF2H3	general transcription factor IIH, polypeptide 3, 34kDa	Nucleus	transcription regulator	VH	1.267	0.083	-1.182	0.538	-1.372	0.143
gi_119914213_ref_XM_870485.2_	618161	BRF1	BRF1 homolog, subunit of RNA polymerase III transcrip	Nucleus	transcription regulator	H	1.276	0.082	-1.480	0.045	1.042	1.181
gi_110626168_ref_NM_176641.1_	338045	SQSTM1	sequestosome 1	Cytoplasm	transcription regulator	VH	1.277	0.079	1.197	1.258	1.275	1.326
gi_139947539_ref_NM_001083683.1_	513928	ATF6B	activating transcription factor 6 beta	Cytoplasm	transcription regulator	H	1.287	0.075	1.058	1.174	1.029	1.198
gi_115495660_ref_NM_001076161.1_	539431	MEN1	multiple endocrine neoplasia I	Nucleus	transcription regulator	VH	1.293	0.069	-1.056	0.944	-1.053	0.994
gi_139949051_ref_NM_001083684.1_	514216	TCF19	transcription factor 19	Nucleus	transcription regulator	VH	1.301	0.064	-1.260	0.318	-1.505	0.044
gi_119918919_ref_XM_864789.2_	514176	ZDHHC13	zinc finger, DHHC-type containing 13	Nucleus	transcription regulator	H	1.305	0.063	1.609	0.449	1.279	1.333
gi_124430747_ref_NM_001075811.2_	520800	TFE3	transcription factor binding to IGHM enhancer 3	Nucleus	transcription regulator	VH	1.306	0.061	-1.100	0.843	-1.113	0.802
gi_119917078_ref_XM_583422.3_	506908	CLIP2	CAP-GLY domain containing linker protein 2	Cytoplasm	transcription regulator	H	1.307	0.062	-1.048	0.926	1.079	1.281
gi_162287360_ref_NM_001012671.2_	508541	STAT3	signal transducer and activator of transcription 3 (acute	Nucleus	transcription regulator	VH	1.311	0.059	-1.059	0.946	1.558	0.697
gi_28372480_ref_NM_175712.1_	281730	CTBP2	C-terminal binding protein 2	Nucleus	transcription regulator	H	1.313	0.058	1.005	1.092	-1.067	0.935
gi_93205067_ref_NM_001001161.2_	407239	USF1	upstream transcription factor 1	Nucleus	transcription regulator	VH	1.314	0.060	-1.874	0.001	1.204	1.340

gi_119915224_ref_XM_001250850.1_	517805	FOXP4	forkhead box P4	Nucleus	transcription regulator	H	1.318	0.057	-1.419	0.083	-1.010	1.077
gi_88319935_ref_NM_174617.3_	282376	STAT5B	signal transducer and activator of transcription 5B	Nucleus	transcription regulator	H	1.318	0.054	1.062	1.166	1.292	1.301
gi_119910262_ref_XM_612960.3_	533526	CCNE1	cyclin E1	Nucleus	transcription regulator	H	1.319	0.053	-1.166	0.535	-1.875	0.002
gi_114051811_ref_NM_001046417.1_	536421	CYLD	cylindromatosis (turban tumor syndrome)	Nucleus	transcription regulator	H	1.322	0.054	1.302	1.193	1.326	1.233
gi_115497533_ref_NM_001075873.1_	525428	E2F4	E2F transcription factor 4, p107/p130-binding	Nucleus	transcription regulator	VH	1.333	0.049	-1.382	0.107	-1.447	0.069
gi_119910692_ref_XM_001256663.1_	790100	IRF2BP1	interferon regulatory factor 2 binding protein 1	Nucleus	transcription regulator	H	1.334	0.053	-2.899	0.000	-1.059	0.926
gi_6852489_gb_AW353499.1_AW35349	353105	STAT6	signal transducer and activator of transcription 6, interl	Nucleus	transcription regulator	VH	1.335	0.049	-1.169	0.544	1.202	1.341
gi_156121100_ref_NM_001102227.1_	539120	VEZF1	vascular endothelial zinc finger 1	Nucleus	transcription regulator	H	1.339	0.045	1.126	1.237	1.181	1.339
gi_119907798_ref_XM_585224.3_	508448	PSMC3	proteasome (prosome, macropain) 26S subunit, ATPas	Nucleus	transcription regulator	VH	1.339	0.048	-1.324	0.194	-1.507	0.043
gi_119907105_ref_XM_585092.3_	508330	MLL	myeloid/lymphoid or mixed-lineage leukemia (trithorax	Nucleus	transcription regulator	H	1.343	0.044	-1.283	0.072	1.289	1.321
gi_157280010_ref_NM_001105051.1_	100125778	RING1	ring finger protein 1	Nucleus	transcription regulator	VH	1.345	0.044	-1.632	0.010	-1.266	0.339
gi_119915255_ref_XM_612306.3_	533039	SRF	serum response factor (c-fos serum response element-	Nucleus	transcription regulator	VH	1.346	0.043	-1.461	0.054	-1.263	0.334
gi_156120882_ref_NM_001102118.1_	528433	DDX20	DEAD (Asp-Glu-Ala-Asp) box polypeptide 20	Nucleus	transcription regulator	VH	1.349	0.041	-1.069	0.905	-1.154	0.659
gi_119907824_ref_XM_583654.3_	507100	SPI1	spleen focus forming virus (SFFV) proviral integration (	Nucleus	transcription regulator	VH	1.349	0.042	1.111	1.209	1.256	1.335
gi_77736090_ref_NM_001034572.1_	530409	PHB (includes EG	prohibitin	Nucleus	transcription regulator	VH	1.350	0.041	-1.498	0.030	-1.757	0.005
gi_134085849_ref_NM_001083382.1_	507180	PHF5A	PHD finger protein 5A	Nucleus	transcription regulator	VH	1.352	0.041	-1.605	0.012	-1.945	0.001
gi_119919541_ref_XM_586187.3_	509260	DEAF1	deformed epidermal autoregulatory factor 1 (Drosophil	Nucleus	transcription regulator	H	1.355	0.043	-1.206	0.444	-1.103	0.732
gi_62751635_ref_NM_001015527.1_	505702	GTF2F1	general transcription factor IIF, polypeptide 1, 74kDa	Nucleus	transcription regulator	VH	1.355	0.040	-1.424	0.071	-1.553	0.025
gi_115497343_ref_NM_001075627.1_	513544	SAP30BP	SAP30 binding protein	Nucleus	transcription regulator	VH	1.360	0.039	-1.219	0.296	-1.442	0.072
gi_154707855_ref_NM_001099177.1_	618386	TCF25	transcription factor 25 (basic helix-loop-helix)	Nucleus	transcription regulator	VH	1.365	0.037	-1.075	0.865	1.026	1.193
gi_164448549_ref_NM_001024497.2_	507867	ANKZF1	ankyrin repeat and zinc finger domain containing 1	Nucleus	transcription regulator	VH	1.371	0.041	-1.629	0.010	-1.725	0.006
gi_119904250_ref_XM_001251674.1_	783869	GTF3C5	general transcription factor IIIC, polypeptide 5, 63kDa	Nucleus	transcription regulator	VH	1.372	0.036	-1.400	0.097	-1.390	0.124
gi_119879733_ref_XM_614853.3_	541166	ETV5	ets variant 5	Nucleus	transcription regulator	VH	1.388	0.032	-1.266	0.303	-1.796	0.004
gi_119884881_ref_XM_606317.3_	527910	SKIL	SKI-like oncogene	Nucleus	transcription regulator	H	1.394	0.028	2.352	0.012	2.431	0.029
gi_149642704_ref_NM_001099175.1_	618232	MED15	mediator complex subunit 15	Nucleus	transcription regulator	H	1.398	0.028	-1.943	0.000	-1.368	0.143
gi_77736577_ref_NM_001034800.1_	617277	TCEB2	transcription elongation factor B (SIII), polypeptide 2 (1	Nucleus	transcription regulator	VH	1.401	0.031	-1.468	0.048	-1.421	0.090
gi_119905767_ref_XM_606138.3_	527740	CBFA2T2	core-binding factor, runt domain, alpha subunit 2; trans	Nucleus	transcription regulator	H	1.402	0.032	1.114	1.186	1.269	1.330
gi_62751533_ref_NM_001015625.1_	520639	CLPB	ClpB caseinolytic peptidase B homolog (E. coli)	Nucleus	transcription regulator	H	1.405	0.030	-1.429	0.064	-1.847	0.003
gi_119911147_ref_XM_609413.3_	530932	TCEB2	transcription elongation factor B (SIII), polypeptide 2 (1	Nucleus	transcription regulator	VH	1.405	0.030	-1.507	0.030	-1.457	0.066
gi_114052113_ref_NM_001046375.1_	534228	PRPF6	PRP6 pre-mRNA processing factor 6 homolog (S. cere	Nucleus	transcription regulator	VH	1.415	0.026	-1.062	0.902	-1.072	0.915
gi_119915359_ref_XM_583214.3_	506727	NFKBIE	nuclear factor of kappa light polypeptide gene enhance	Nucleus	transcription regulator	H	1.419	0.028	-1.186	0.484	1.002	1.105
gi_119916184_ref_XM_596212.3_	518030	TAF4B	TAF4b RNA polymerase II, TATA box binding protein (T	Nucleus	transcription regulator	H	1.448	0.021	-1.169	0.541	-1.591	0.019
gi_165905558_ref_NM_001046125.2_	512562	MAGED1	melanoma antigen family D, 1	Plasma Membrane	transcription regulator	VH	1.452	0.018	1.158	1.252	1.181	1.341
gi_115496443_ref_NM_001075346.1_	508127	DMAP1	DNA methyltransferase 1 associated protein 1	Nucleus	transcription regulator	VH	1.458	0.018	-1.353	0.145	-1.244	0.351
gi_119915909_ref_XM_865312.2_	506141	IRF4	interferon regulatory factor 4	Nucleus	transcription regulator	H	1.470	0.018	-1.848	0.001	-2.502	0.000
gi_119911595_ref_XM_868906.2_	538671	SUPT6H	suppressor of Ty 6 homolog (S. cerevisiae)	Nucleus	transcription regulator	VH	1.504	0.013	-1.338	0.165	-1.077	0.908
gi_164420754_ref_NM_001076875.2_	525256	PREB	prolactin regulatory element binding	Nucleus	transcription regulator	H	1.507	0.013	-1.706	0.005	-1.772	0.004
gi_119911729_ref_XM_870907.2_	618576	MNT	MAX binding protein	Nucleus	transcription regulator	H	1.509	0.013	-2.683	0.000	1.009	1.092
gi_115497653_ref_NM_001075240.1_	506097	NFIL3	nuclear factor, interleukin 3 regulated	Nucleus	transcription regulator	H	1.511	0.011	1.026	1.137	-1.191	0.536
gi_84370155_ref_NM_001038565.1_	539291	MYCBP	c-myc binding protein	Nucleus	transcription regulator	H	1.513	0.012	-1.597	0.013	-2.021	0.000
gi_125630716_ref_NM_001081542.1_	541125	ELK3	ELK3, ETS-domain protein (SRF accessory protein 2)	Nucleus	transcription regulator	VH	1.517	0.011	-1.007	1.057	-1.079	0.887
gi_47564055_ref_NM_001001150.1_	407219	MITF	microphthalmia-associated transcription factor	Nucleus	transcription regulator	H	1.532	0.010	1.643	0.373	1.919	0.196
gi_114052756_ref_NM_001045875.1_	407125	EGR1	early growth response 1	Nucleus	transcription regulator	H	1.533	0.010	-1.221	0.400	1.800	0.314
gi_84000132_ref_NM_001038072.1_	510666	BMI1	BMI1 polycomb ring finger oncogene	Nucleus	transcription regulator	VH	1.542	0.009	1.093	1.217	-1.036	1.038
gi_119894776_ref_XR_028159.1_	784795	CARM1	coactivator-associated arginine methyltransferase 1	Nucleus	transcription regulator	VH	1.543	0.010	-1.542	0.024	-1.214	0.456
gi_115497489_ref_NM_001075872.1_	525408	NFKBIB	nuclear factor of kappa light polypeptide gene enhance	Nucleus	transcription regulator	H	1.545	0.008	-1.911	0.000	-2.075	0.000
gi_119894953_ref_XM_001255535.1_	788491	ZBTB7A	zinc finger and BTB domain containing 7A	Nucleus	transcription regulator	H	1.550	0.010	-2.271	0.000	-1.444	0.073
gi_78042545_ref_NM_001035098.1_	511280	NMI	N-myc (and STAT) interactor	Cytoplasm	transcription regulator	H	1.564	0.007	1.158	1.234	-1.206	0.457
gi_115495776_ref_NM_001075448.1_	510420	MYBL2	v-myb myeloblastosis viral oncogene homolog (avian)-	Nucleus	transcription regulator	VH	1.567	0.008	-1.877	0.000	-2.078	0.000
gi_119901277_ref_XM_615634.3_	535530	FOXO3	forkhead box O3	Nucleus	transcription regulator	VH	1.571	0.007	-1.003	0.925	1.823	0.287
gi_78369189_ref_NM_001035349.1_	524118	ARID5A	AT rich interactive domain 5A (MRF1-like)	Nucleus	transcription regulator	H	1.592	0.006	-2.243	0.000	-1.682	0.008
gi_115497487_ref_NM_001076141.1_	539003	CTNNB1	catenin (cadherin-associated protein), beta 1, 88kDa	Nucleus	transcription regulator	VH	1.680	0.004	1.025	1.137	-1.034	1.042
gi_116004218_ref_NM_001077001.1_	767928	RBPJ	recombination signal binding protein for immunoglobuli	Nucleus	transcription regulator	VH	1.689	0.004	1.042	1.151	-1.195	0.527
gi_119905866_ref_XM_615740.3_	535632	TGIF2	TGFB-induced factor homeobox 2	Nucleus	transcription regulator	H	1.704	0.004	-1.476	0.045	-1.058	0.891
gi_119917017_ref_XM_877865.2_	509874	GTF3C1	general transcription factor IIIC, polypeptide 1, alpha 2	Nucleus	transcription regulator	H	1.748	0.002	-1.615	0.013	-1.028	1.046
gi_76611568_ref_XM_869196.1_	617024	E2F2	E2F transcription factor 2	Nucleus	transcription regulator	H	1.780	0.002	-2.427	0.000	-2.359	0.000
gi_190360732_ref_NM_001128499.1_	510050	TGIF1	TGFB-induced factor homeobox 1	Nucleus	transcription regulator	H	1.793	0.002	1.089	1.179	-1.444	0.070
gi_118151389_ref_NM_001077900.1_	510814	STAT1	signal transducer and activator of transcription 1, 91kD	Nucleus	transcription regulator	VH	1.847	0.001	1.189	1.255	1.154	1.333
gi_119889242_ref_XM_585895.3_	509019	ZBTB7B	zinc finger and BTB domain containing 7B	Nucleus	transcription regulator	H	1.900	0.001	-2.238	0.000	1.707	0.419
gi_119904107_ref_XM_599033.3_	493639	PTGES2	prostaglandin E synthase 2	Cytoplasm	transcription regulator	H	1.917	0.001	-1.753	0.002	-1.995	0.001
gi_106880502_ref_NM_001034710.2_	540605	CREM	cAMP responsive element modulator	Nucleus	transcription regulator	H	1.919	0.001	-1.359	0.144	-1.403	0.103
gi_119911776_ref_XM_590665.3_	513045	MYBBP1A	MYB binding protein (P160) 1a	Nucleus	transcription regulator	VH	1.927	0.001	-2.545	0.000	-2.738	0.000
gi_154152114_ref_NM_001100336.1_	510923	TRIM25	tripartite motif-containing 25	Cytoplasm	transcription regulator	VH	1.957	0.001	-1.054	0.953	1.074	1.262
gi_119893790_ref_XM_608827.3_	530358	KLF3	Kruppel-like factor 3 (basic)	Nucleus	transcription regulator	H	2.005	0.001	1.040	1.146	1.263	1.337
gi_115497081_ref_NM_001075656.1_	514246	JUNB	jun B proto-oncogene	Nucleus	transcription regulator	H	2.028	0.001	-3.199	0.000	-2.049	0.000
gi_119916372_ref_XM_616030.3_	535916	SMAD7	SMAD family member 7	Nucleus	transcription regulator	H	2.067	0.000	1.297	1.200	2.153	0.082
gi_115497301_ref_NM_001076409.1_	616115	NFKB1	nuclear factor of kappa light polypeptide gene enhance	Nucleus	transcription regulator	VH	2.141	0.000	-1.398	0.093	-1.145	0.698
gi_60592797_ref_NM_001012673.1_	282375	STAT5A	signal transducer and activator of transcription 5A	Nucleus	transcription regulator	VH	2.254	0.000	-1.087	0.840	1.193	1.342
gi_77735604_ref_NM_001034326.1_	508638	NOC2L	nucleolar complex associated 2 homolog (S. cerevisiae	Nucleus	transcription regulator	VH	2.327	0.000	-1.498	0.032	-1.790	0.004
gi_77736398_ref_NM_001034727.1_	541236	XBP1	X-box binding protein 1	Nucleus	transcription regulator	H	2.404	0.000	-2.298	0.000	-1.747	0.006
gi_115495324_ref_NM_001075935.1_	530610	ATF6	activating transcription factor 6	Cytoplasm	transcription regulator	VH	2.521	0.000	-1.551	0.020	-1.693	0.008
gi_118601779_ref_NM_001079591.1_	513983	SCAND1	SCAN domain containing 1	Nucleus	transcription regulator	H	2.563	0.000	-1.869	0.000	-2.168	0.000
gi_164519007_ref_NM_001113302.1_	539361	SREBF1	sterol regulatory element binding transcription factor 1	Nucleus	transcription regulator	H	2.595	0.000	-7.844	0.000	-2.673	0.000
gi_77735420_ref_NM_001034231.1_	505025	ID2	inhibitor of DNA binding 2, dominant negative helix-loo	Nucleus	transcription regulator	VH	2.939	0.000	1.192	1.252	1.082	1.282

gi_119900643_ref_XM_604596.3_	526233	EGR3	early growth response 3	Nucleus	transcription regulator	H	3.238	0.000	-3.012	0.000	-1.776	0.004
gi_41386700_ref_NM_174726.1_	282713	NFKBIZ	nuclear factor of kappa light polypeptide gene enhance	Nucleus	transcription regulator	H	3.766	0.000	1.912	0.102	1.945	0.183
gi_67972412_ref_NM_001024929.1_	506945	BHLHE40	basic helix-loop-helix family, member e40	Nucleus	transcription regulator	VH	3.779	0.000	1.098	1.217	1.019	1.188
gi_110347584_ref_NM_182786.2_	280795	FOS	FBJ murine osteosarcoma viral oncogene homolog	Nucleus	transcription regulator	H	3.966	0.000	-1.263	0.279	-1.537	0.029
gi_119901924_ref_XM_001251809.1_	515125	SMAD3	SMAD family member 3	Nucleus	transcription regulator	H	4.105	0.000	1.177	1.249	1.416	1.060
gi_119901328_ref_XM_618588.3_	538384	PRDM1	PR domain containing 1, with ZNF domain	Nucleus	transcription regulator	H	4.186	0.000	-1.006	1.041	1.355	1.166
gi_115495012_ref_NM_001075819.1_	521378	CITED2	Cbp/p300-interacting transactivator, with Glu/Asp-rich c	Nucleus	transcription regulator	H	5.909	0.000	-2.431	0.000	-2.881	0.000
gi_119888839_ref_XM_869638.2_	617389	RUNX3	runt-related transcription factor 3	Nucleus	transcription regulator	H	8.600	0.000	-1.414	0.084	1.562	0.689
gi_156120848_ref_NM_001102101.1_	526392	NFKB2	nuclear factor of kappa light polypeptide gene enhance	Nucleus	transcription regulator	H	9.354	0.000	-2.838	0.000	-1.390	0.123
gi_119903790_ref_XM_864138.2_	509889	FOSL2	FOS-like antigen 2	Nucleus	transcription regulator	H	12.227	0.000	-1.035	0.922	1.401	1.087
