# Supplementary material for: A Bovine Lymphosarcoma Cell Line Infected with Theileria annulata Exhibits an Irreversible Reconfiguration of Host Cell Gene Expression
Source: PLoS One. 2013 Jun 26;8(6):e66833. doi: 10.1371/journal.pone.0066833 (PMC3694138; doi:10.1371/journal.pone.0066833)
Supplement: Table S9 — Transcription regulators predicted to be either activated or inhibited in untreated TBL20 (A) OR at 24 h (B) OR 48 h (C) following exposure to BW720c. (PDF) [file pone.0066833.s011.pdf]

Table S9: Transcription regulators predicted to be either activated or inhibited in untreated TBL20 (A) *OR* at 24h (B) *OR* 48h (C) following exposure to BW720c

A. Activated or Inhibited in TBL20 compared to BL20

Regulator	Predicted Activation Status in TBL20	z-score	p-value	Target gene ratio
STAT2	Activated	2.569	$1.15x10^{-4}$*	9/12
CREB1	Activated	2.508	$3.45x10^{-6}$*	24/51
CEBPB	Activated	2.450	$7.65x10^{-5}$*	30/59
STAT4	Activated	2.337	$5.84x10^{-10}$*	40/58
TP63	Activated	2.212	$5.02x10^{-6}$*	13/18
MTPN	Activated	2.122	$1.58x10^{-2}$	15/17
RUNX3	Inhibited	-2.038	$3.57x10^{-2}$	4/8
RUNX1	Inhibited	-2.123	$1.70x10^{-2}$	4/6
NKX2-1	Inhibited	-2.205	$7.66x10^{-3}$	4/31
KDM5B	Inhibited	-2.239	$6.91x10^{-3}$	18/23
POU2AF1	Inhibited	-2.261	$1.41x10^{-3}$	5/12
SOX2	Inhibited	-2.280	$1.87x10^{-2}$	7/13
TCF4	Inhibited	-2.360	$3.74x10^{-2}$	5/10
MYB	Inhibited	-2.419	$1.54x10^{-3}$	9/16
NR3C1	Inhibited	-2.487	$6.53x10^{-14}$*	33/131
EBF1	Inhibited	-2.653	$7.71x10^{-2}$	5/6
PAX5	Inhibited	-2.796	$2.68x10^{-4}$*	7/11
HDAC	Inhibited	-2.873	$8.60x10^{-6}$*	13/16

B. Activated or Inhibited in TBL20 at 24h BW720c compared to untreated TBL20 control

Regulator	Predicted Activation Status in TBL20 24h BW720c	z-score	p-value	Target gene ratio
CREBBP	Activated	2.425	$9.02x10^{-2}$	12/21
IRF9	Activated	2.383	$2.14x10^{-4}$*	6/9
CEBPD	Activated	2.123	$4.00x10^{-1}$	4/5
FLI1	Inhibited	-2.047	$1.64x10^{-1}$	5/6
SP1	Inhibited	-2.067	$9.35x10^{-3}$	18/51
ATF4	Inhibited	-2.188	$5.89x10^{-3}$	9/16
NR1H3	Inhibited	-2.320	$7.35x10^{-5}$*	12/17
IFI16	Inhibited	-2.391	$7.81x10^{-2}$	8/9
ETS1	Inhibited	-2.671	$8.69x10^{-3}$	10/23

C. Activated or Inhibited in TBL20 at 48h BW720c compared to TBL20 control

Regulator	Predicted Activation Status in TBL20 48h BW720c	z-score	p-value	Target gene ratio
CDKN2A	Activated	3.703	$1.32x10^{-5}$*	32/40
TP53 (includes EG:22059)	Activated	2.726	$9.09x10^{-9}$*	74/162
RB1	Activated	2.656	$7.48x10^{-4}$*	16/41
Rb	Activated	2.545	$1.59x10^{-1}$	8/8
NR4A2	Activated	2.469	$4.17x10^{-1}$	5/5
SIRT1	Activated	2.433	$5.26x10^{-3}$	10/14
SPI1 (includes EG:20375)	Activated	2.418	$9.37x10^{-8}$*	14/38
BCL3	Activated	2.412	$1.64x10^{-2}$	6/7
Stat3-Stat3	Activated	2.240	$1.76x10^{-1}$	4/4
MEF2A	Activated	2.200	$7.57x10^{-2}$	4/5
MYOG	Activated	2.185	$1.20x10^{-1}$	4/5
IRF1 (includes EG:16362)	Activated	2.137	$1.15x10^{-6}$*	19/32
SOX9	Activated	2.131	$3.94x10^{-1}$	4/5
MITF	Activated	2.119	$6.86x10^{-2}$	11/20
MEF2D	Activated	2.097	$1.61x10^{-3}$	4/6
AR	Activated	2.070	$1.58x10^{-2}$	16/34
TOB1	Activated	2.061	$8.84x10^{-2}$	7/8
RUNX2	Activated	2.038	$5.83x10^{-2}$	7/11
MEF2C	Activated	2.027	$1.50x10^{-1}$	5/7
MED1 (includes EG:19014)	Inhibited	-2.002	$4.28x10^{-1}$	5/7
DDIT3	Inhibited	-2.047	$2.49x10^{-2}$	5/7
CCNE1	Inhibited	-2.053	$1.95x10^{-2}$	5/5
NR1H3	Inhibited	-2.078	$4.94x10^{-4}$*	12/18
E2F2	Inhibited	-2.133	$5.93x10^{-4}$*	6/15
E2F3	Inhibited	-2.251	$4.54x10^{-5}$*	7/18
E2F1	Inhibited	-2.346	$1.64x10^{-2}$	26/48
PPARGC1A	Inhibited	-2.353	$2.55x10^{-2}$	16/21
IFI16	Inhibited	-2.570	$1.35x10^{-1}$	9/10
NFE2L2	Inhibited	-3.120	$1.11x10^{-5}$*	47/65
